# Biomimetic design of an α-ketoacylphosphonium-based light-activated oxygenation auxiliary†

**DOI:** 10.1039/d3sc03572g

**Published:** 2023-09-08

**Authors:** Ryoto Oya, Kenji Ota, Masaaki Fuki, Yasuhiro Kobori, Masahiro Higashi, Kazunori Nagao, Hirohisa Ohmiya

**Affiliations:** a Division of Pharmaceutical Sciences, Graduate School of Medical Sciences, Kanazawa University Kakuma-Machi Kanazawa 920-1192 Japan; b Institute for Chemical Research, Kyoto University Gokasho, Uji Kyoto 611-0011 Japan nagao.kazunori.4j@kyoto-u.ac.jp ohmiya@scl.kyoto-u.ac.jp; c Molecular Photoscience Research Center, Department of Chemistry, Graduate School of Science, Kobe University Kobe 657-8501 Japan; d Department of Molecular Engineering, Graduate School of Engineering, Kyoto University Kyoto 615-8510 Japan

## Abstract

The biomimetic design of a transition metal complex based on the iron(iv)-oxo porphyrin π-cation radical species in cytochrome P450 enzymes has been studied extensively. Herein, we translate the functions of this iron(iv)-oxo porphyrin π-cation radical species to an α-ketoacyl phosphonium species comprised of non-metal atoms and utilize it as a light-activated oxygenation auxiliary for *ortho*-selective oxygenation of anilines. Visible light irradiation converts the α-ketoacyl phosphonium species to the excited state, which acts as a transiently generated oxidant. The intramolecular nature of the process ensures high regioselectivity and chemoselectivity. The auxiliary is easily removable. A one-pot protocol is also described.

## Abstract 2

The biomimetic design of a transition metal complex based on the iron(iv)-oxo porphyrin π-cation radical species in cytochrome P450 enzymes has been studied extensively.

## Graphical abstract

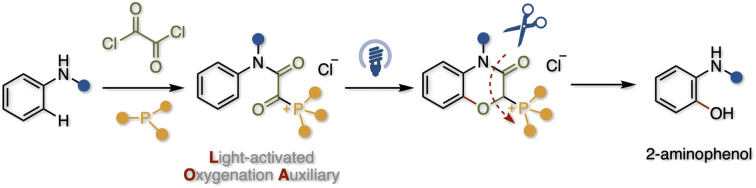

## Introduction

**Fig. 1:**
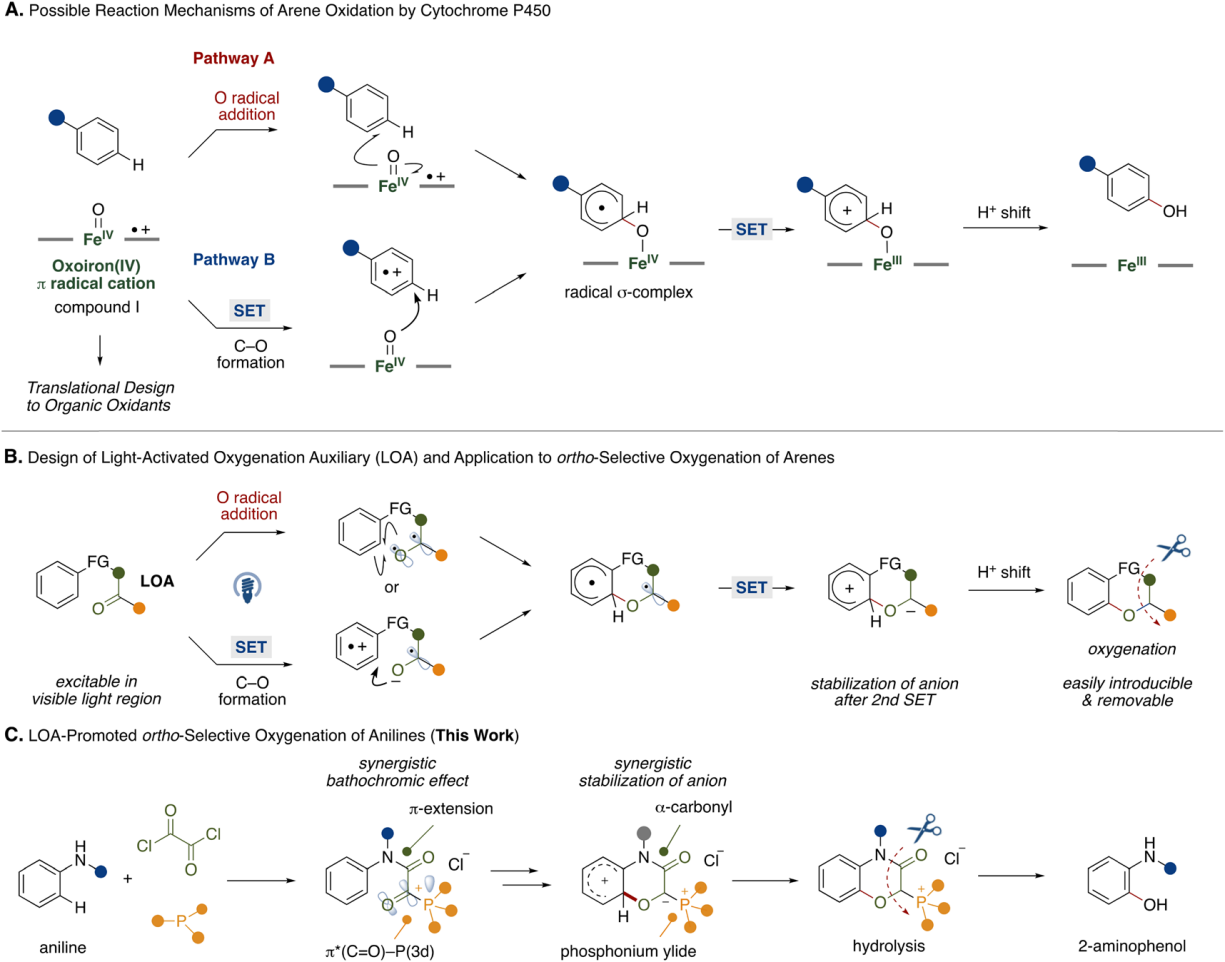
Biomimetic design of light-activated oxygenation auxiliary and its application.

Biomimetic reaction design provides ideal and direct solutions to synthetic problems. For example, heme iron centers are active sites in cytochrome P450 enzymes, playing a crucial role in oxygenation of arene-based xenobiotics including pharmaceutical drugs and simple hydrocarbons.^1^ In such arene oxygenation reactions by cytochrome P450, several investigations suggested the involvement of iron(iv)-oxo porphyrin π-cation radicals (compound I) generated by heme iron centers and molecular oxygen or other oxidants.^2^ Since the heme iron center is capable of oxidizing various arenes, the biomimetic design of iron(iv)-oxo porphyrin π-cation radicals with various combinations of transition metals and ligands has been the focus of development.^3^ Among some putative reaction pathways, two reaction pathways A and B, are shown in Fig. 1A. In pathway A, which is the target of many biomimetic systems, the reaction starts with addition of an oxygen atom from the iron(iv)-oxo porphyrin π-cation radicals to an arene in a radical manner. The resultant radical *σ*-complex undergoes intramolecular electron transfer followed by proton shift to form a phenol scaffold. Recently, Asaka and Fujii disclosed that a single electron transfer (SET) process might be the rate limiting step in arene C–H oxygenation by iron(iv)-oxo porphyrin π-cation radical species in an alternative reaction pathway (pathway B).^4^ The reaction involves the single electron oxidation of an arene substrate by iron(iv)-oxo porphyrin π-cation radicals to form an arene radical cation and iron(iv)-oxo intermediate in the solvent cage. Subsequently, the intermediate reacts with the arene radical cation to produce a radical *σ*-complex. In this reaction process, the iron(iv)-oxo porphyrin π-cation radicals function as two equivalents of single electron oxidants and an oxygen atom source. While CYP450-mediated oxidation provides a powerful tool for C–H oxygenation, the regioselectivity relies on the electronic nature of the arene substrates.

Inspired by these studies, we questioned whether we could mimic the functions of the iron(iv)-oxo porphyrin π-cation radicals with transition metal-free fragments and oxygenate the proximal position of an arene to achieve metal-free regioselective arene C–H oxygenation. For this strategy, we developed a light-activated oxygenation auxiliary (LOA) for *ortho*-selective oxygenation of arenes (Fig. 1B). The LOA contains a carbonyl group which is excitable in the visible light region and is covalently introduced to the substrate. The carbonyl group is assumed to adopt a biradical state (Fig. 1B, top) or become an acceptor moiety (Fig. 1B, bottom) upon photoexcitation, whereupon it can oxidize a proximal arene substituent through O radical addition of the biradical state or single electron transfer followed by C–O bond formation within the resultant radical ion pair. The biradical intermediate would then undergo rearomatization by single electron oxidation of the remaining carbon radical on the LOA group followed by a proton shift to lead to the oxygenated product. To make this hypothesis feasible, the requirements of the LOA are as follows: (1) must be excitable in the visible light region, (2) can stabilize the anion generated by the 2nd single electron transfer and (3) be easily introducible and removable.

To satisfy these requirements, we focused on an α-ketoacyl phosphonium salt consisting of a 1,2-dicarbonyl structure and acyl phosphonium^5^ species as the LOA for *ortho*-selective C–H oxygenation of anilines (Fig. 1C). While aliphatic and aromatic ketones require hazardous UV light for excitation,^6^ the 1,2-dicarbonyl structure allows for a red shift in the absorption wavelength.^7^ Furthermore, conjugation of a third-row heteroatom to the carbonyl group leads to a red shift in the absorption wavelength through interaction of a 3d orbital of the heteroatom and the π* orbital of the carbonyl group.^8^ This synergistic bathochromic effect might allow the LOA to be excited in the visible light region. Additionally, the synergistic electron-withdrawing natures of the carbonyl and phosphonium moieties would contribute to the stabilization of the anion generated by a second single electron oxidation of the arene radical.

With the above requirements in mind, we designed iron(iv)-oxo porphyrin π-cation radicals mimic with an α-ketoacyl phosphonium LOA for *ortho*-selective oxygenation of anilines, providing a synthetic route to 2-aminophenol derivatives that are present in natural products and pharmaceutical drugs and materials.^9^ Conventionally, electrophilic peroxide reagents have been utilized for *ortho*-selective oxidation of anilines.^10^ However, these protocols suffer from moderate regioselectivity and low chemoselectivity. To overcome such problems, directing group strategies that combine transition metal catalysts and suitable oxidants have been extensively studied.^11,12^ Our LOA-based strategy might add a tactically different and transition metal-free protocol to the synthetic toolbox for 2-aminophenols.

## Results and discussion

After screening reaction conditions, we found that acyl chloride 1a-1 derived from *N*-methylaniline 1a (0.2 mmol) and an excess amount of oxalyl chloride reacted with an equimolar amount of PCy_3_ in MeCN under visible light irradiation to afford a phosphonium salt bearing a 1,4-benzoxazin-3-one scaffold (2a) in 86% isolated yield (Table 1, entry 1). The effects of various nucleophiles and solvents were evaluated (Table 1, entries 2–10). While the less hindered PBu_3_ showed moderate reactivity, the reaction did not proceed at all with P^*t*^Bu_3_, PMe_3_ and PPh_3_ (entries 2–5). No reactivity of these phosphines would be attributed to the inefficient formation of an acyl phosphonium species due to the steric hindrance and the low nucleophilicity. Amine nucleophiles, such as DMAP (4-dimethylaminopyridine) and DABCO (1,4-diazabicyclo[2.2.2]octane), did not provide the product (entries 6 and 7). While PrCN and DCM were comparable with MeCN, other solvents exhibited lower reactivity than MeCN (entries 8–11). Control experiments revealed that both light irradiation and phosphine were necessary for this reaction (entries 12 and 13). This protocol permitted scale-up to 1.0 mmol without any modification of the reaction conditions (entry 14). In the conventional oxygenation of arene C–H bonds, competing oxygenation of weak C(sp^3^)–H bonds and overoxidation are problems. However, neither oxygenation of the methyl group on the nitrogen nor overoxidation were observed.

**Table 1:** Screening of reaction conditions^a^

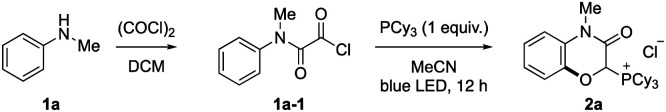		
Entry	Change from standard conditions	Yield of 2a^b^ (%)
1	None	94 (86)
2	PBu_3_ instead of PCy_3_	77
3	P^*t*^Bu_3_ instead of PCy_3_	0
4	PMe_3_ instead of PCy_3_	0
5	PPh_3_ instead of PCy_3_	0
6	DMAP instead of PCy_3_	0
7	DABCO instead of PCy_3_	0
8	PrCN instead of MeCN	90
9	DCM instead of MeCN	88
10	AcOEt instead of MeCN	48
11	Acetone instead of MeCN	30
12	Without blue LED	0
13	Without phosphine	0
14	1 mmol scale	83 (86)

a Oxalyl chloride protection of anilines was carried out with 1a (1.0 mmol) and oxalyl chloride (10.0 mmol) in dichloromethane (10 mL). Photoreaction was carried out with the oxalyl chloride-protected aniline (0.2 mmol) and tricyclohexylphosphine (0.2 mmol) in acetonitrile (2.0 mL) with blue LED irradiation for 12 h.

b ^1^H-NMR yield. The number in parenthesis is isolated yield.

**Fig. 2:**
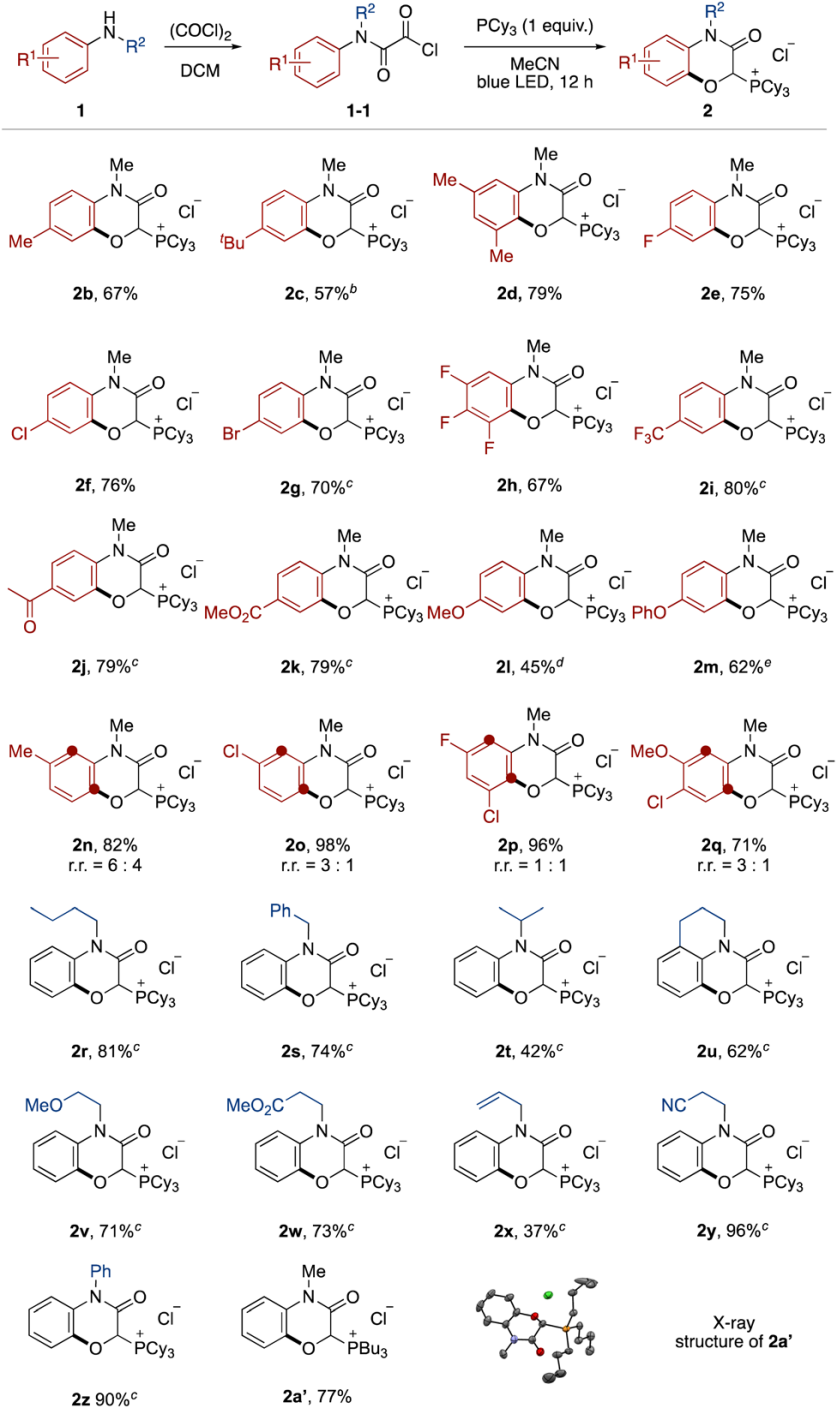
Substrate scope. ^*a a*^Oxalyl chloride protection of anilines was carried out with 1a (0.2 mmol) and oxalyl chloride (2.0 mmol) in dichloromethane. Photoreaction was carried out with the oxalyl chloride-protected aniline 1-1 and tricyclohexylphosphine (0.2 mmol) in acetonitrile (2.0 mL) with blue LED irradiation for 12 h. ^*b*^Light irradiation time was modified to 48 h ^*c*^at a 1.0 mmol scale. ^*d*^Light irradiation time was modified to 84 h. ^*e*^Light irradiation time was modified to 42 h.

The effect of the aromatic ring of the *N*-alkyl anilines 1 was investigated (Fig. 2). Anilines with alkyl substituents at the *para* and *meta* positions on the aromatic ring were tolerated (2b–2d). This transition-metal-free protocol was compatible with halogen substituents, which offer further functionalization by cross coupling (2e–2h). Electron-deficient anilines ensured high yields (2i–2k). Although it was necessary to prolong the reaction time, electron-rich anilines were also applicable (2l and 2m).^13^ In the case of *meta*-substituted anilines, a mixture of regioisomers was produced (2n–2q).

Next, the *N*-substituent groups on the anilines were evaluated. This protocol tolerated primary and secondary alkyl groups that contain weak C–H bonds (2r–2t). Tetrahydroisoquinoline could be converted to the corresponding tricyclic compound in good yield (2u). Functional groups, such as ether, ester, olefin, and cyano were compatible (2v–2y). Diphenylamine was also oxygenated with *ortho*- and mono-selectivity under the standard reaction conditions (2z). We also isolated the phosphonium salt 2a′ by use of tributylphosphine instead of tricyclohexylphosphine, and the structure could be confirmed by X-ray crystal structure analysis (see ESI†).

**Fig. 3:**
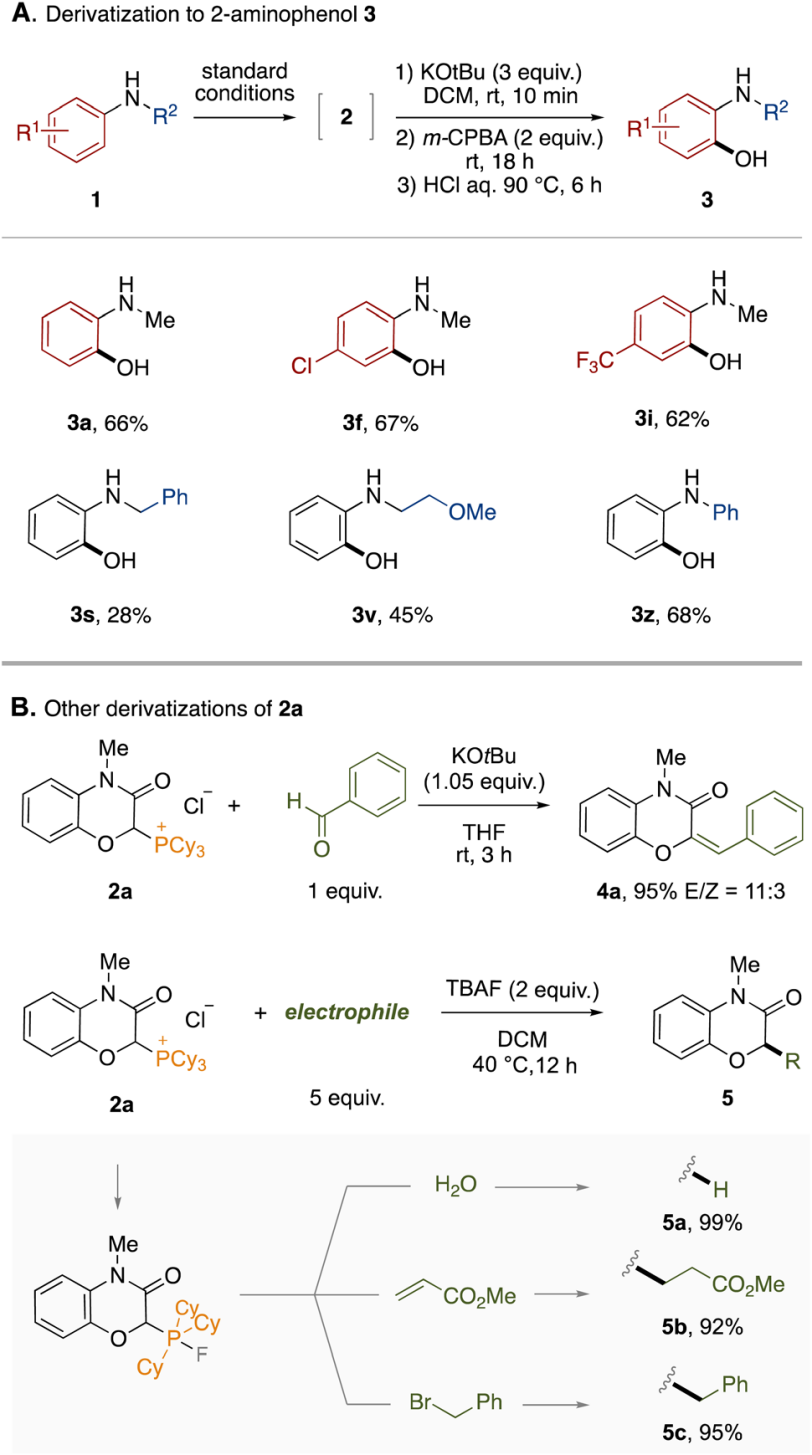
Derivatization.

To demonstrate the synthetic utility of this protocol, the derivatization of the obtained phosphonium salts was conducted. First, a one-pot derivatization of phosphonium salts 2 to 2-aminophenols 3 was examined (Fig. 3A). The phosphonium LOA was found to be removable by treatment with KO^*t*^Bu and *m*-CPBA followed by hydrolysis under acidic conditions. The entire process could be performed in a one-pot manner by simple combination, accomplishing the formal *ortho*-selective C–H oxygenation of anilines. This one-pot protocol was applicable to representative anilines shown in Fig. 3A (3a–3z). The hydrolysis under basic conditions is provided in the ESI.† Furthermore, the phosphonium salt 2a could lead to 2*H*-1,4-benzoxazin-3(4*H*)-one derivatives through various dephosphorylative functionalizations (Fig. 3B). For example, the Wittig reaction between 2a and benzaldehyde afforded the corresponding trisubstituted alkene 4a in high yield. In the presence of tetrabutylammonium fluoride (TBAF), 2a could react with electrophiles. Thus, dephosphorylative protonation of 2a with water occurred to give 2*H*-1,4-benzoxazin-3(4*H*)-one in quantitative yield. Dephosphorylative alkylation was also achieved by use of methyl acrylate or benzyl bromide. In this reaction, a fluoride anion might convert the alkylphosphonium moiety to the corresponding carbanion equivalent through formation of a phosphorane moiety.^14^ We applied the one-pot protocol of LOA-promoted oxygenation and dephosphorylative protonation to the synthesis of flumioxazin (see ESI†).^15^

**Fig. 4:**
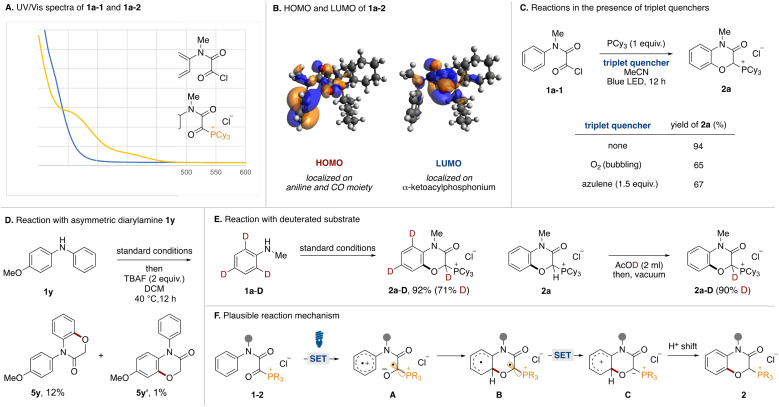
Mechanistic experiments.

Mechanistic experiments were performed to clarify the reaction mechanism. First, UV-vis absorption spectra of acyl chloride 1a-1 and α-ketoacyl phosphonium 1a-2 derived from *N*-methylaniline were measured (Fig. 4A). While the absorption band of 1a-1 did not reach into the visible light region, that of 1a-2 extended to around 450 nm. Therefore, the bathochromic effect by the phosphonium moiety was experimentally confirmed. The HOMO and LUMO orbitals of 1a were evaluated using DFT calculations. Two conformational isomers were found and the HOMO and LUMO of the more stable one are shown (Fig. 4B). The HOMO is comprised of the π orbital of aniline and the *n* orbitals of the two carbonyl groups. Natural Transition Orbitals (NTO) analysis for the S_1_ transition reveal that distribution of the *n* orbital in the HONTO is emphasized. The LUMO and LUNTO are comprised of the π* orbitals of the two carbonyl groups. To elucidate the involvement of a triplet state, the reactions were tested in the presence of triplet quenchers (Fig. 4C). Although the addition of O_2_ or azulene to the optimal reaction conditions diminished the product yield, the reactions were not completely inhibited. We tried to observe triplet species in the reaction by time-resolved electron paramagnetic resonance (TREPR) at 80 K, however, no signals were observed. Currently, we do not have any reasonable explanations based on experimental proof for decrease of yields in the presence of triplet quenchers. Although we could not observe any triplet species, the involvement of triplet species could not be ruled out completely. We conducted the reaction using an asymmetric diarylamine substrate 1y with phenyl and *p*-methoxyphenyl groups on the nitrogen atom (Fig. 4D). After running under the optimal conditions followed by dephosphorylative protonation, two oxygenated products 5y and 5y′ were obtained. The 10 : 1 ratio indicated that C(sp^2^)–O formation preferentially occurred on the simple phenyl group. In Siegel's previous report,^16^ the oxygen radical added preferentially to the electron-rich aromatic ring over the neutral one. This result and the observed low reactivity of 2l and 2m (Fig. 2) were not consistent with an oxygen radical-mediated process. Therefore, we postulated that the charge transfer from the aniline moiety to the α-ketoacyl phosphonium salt might be the major pathway in the excited state.^17^ To determine the origin of the hydrogen atom at the α-position of the phosphonium moiety of 2a, the reaction with *o*- and *p*-deuterated aniline 1a-D was carried out under standard conditions (Fig. 4E, left). The reaction proceeded to afford the deuterated product 2a-D in high yield (71% D incorporation). The moderate D incorporation could be rationalized by the acidic nature of the hydrogen (Fig. 4E, right).

Based on these experimental results, a plausible reaction mechanism is shown in Fig. 4F. Acyl phosphonium salt 1-2 formed from aniline 1, oxalyl chloride, and phosphine becomes excited by blue LED irradiation. In the singlet or triplet excited state of 1-2, intramolecular electron transfer from the aniline moiety to the α-ketoacyl phosphonium moiety occurs to generate the radical ion pair (A). The O anion of the resultant ketyl radical adds onto the *ortho* position of the aniline radical cation, resulting in the formation of the C(sp^2^)–O bond (B). Intramolecular electron transfer generates a phosphonium ylide (C), which is converted to phosphonium salt 2 by a proton shift.

## Conclusions

In summary, we demonstrated a biomimetic design of LOA comprising α-ketoacyl phosphonium-enabled *ortho*-selective C(sp^2^)–H oxygenation of anilines under visible light irradiation. This oxygenation protocol does not require transition metals, and can access synthetically useful 2-aminophenol scaffolds in a one-pot manner. Further applications of this LOA strategy for the oxygenation of other substrates are under investigation.

## Data availability

All experimental and characterization data, as well as pictures of NMR spectra are available in the ESI.†

## Author contributions

R. O., K. O., K. N., and H. O. designed, performed and analysed the experiments. M. F. and Y. K. performed the TREPR experiment. M. H. performed the DFT calculations. K. O., K. N., and H. O. co-wrote the paper. All authors contributed to discussions.

## Conflicts of interest

There are no conflicts to declare.

## Supplementary Material

SC-014-D3SC03572G-s001

SC-014-D3SC03572G-s002
