# Supplementary material for: Biomimetic design of an α-ketoacylphosphonium-based light-activated oxygenation auxiliary
Source: Chem Sci. 2023 Sep 8;14(38):10488–93. doi: 10.1039/d3sc03572g (PMC10548508; doi:10.1039/d3sc03572g)
Supplement: SC-014-D3SC03572G-s001 [file SC-014-D3SC03572G-s001.pdf]

Electronic Supplementary Material (ESI) for Chemical Science.
This journal is © The Royal Society of Chemistry 2023

Ortho-Selective Oxygenation of Anilines by α-Ketoacylphosphonium-Based Light-Activated Oxygenation Auxiliary

Ryoto Oya,[1] Kenji Ota,[2] Masaaki Fuki,[3] Yasuhiro Kobori,[3] Masahiro Higashi,[4] Kazunori Nagao,[2]* Hirohisa Ohmiya[2]*

[1]Division of Pharmaceutical Sciences, Graduate School of Medical Sciences, Kanazawa University, Kakuma-machi, Kanazawa 920-1192, Japan

[2] Institute for Chemical Research, Kyoto University, Gokasho, Uji, Kyoto 611-0011, Japan

[3] Molecular Photoscience Research Center and Department of Chemistry, Graduate School of Science, Kobe University, Kobe 657-8501, Japan

[4] Department of Molecular Engineering, Graduate School of Engineering, Kyoto University, Kyoto 615-8510, Japan

*E-mail: Kazunori Nagao : nagao.kazunori.4j@kyoto-u.ac.jp
Hirohisa Ohmiya: ohmiya@scl.kyoto-u.ac.jp

■ **Table of Contents** ■

1. Instrumentation and Chemicals S2
2. Procedures for Light-Driven Ortho-Selective Oxygenation of Anilines S3
3. Characterization Data for Oxygenated Products S4–S16
4. X-ray Diffraction Analysis for 2a' S17
5. Deprotection of LOA Group S18
6. Characterization of 2-Aminophenols S19–S20
7. Procedures for Dephosphorylative Functionalization S20–S22
8. UV-Vis Adsorption Spectra Experiments S22
9. NMR Spectra of Reaction Intermediates S23–S25
10. DFT Calculation S25–S30
11. Reactions with Labelled Substrate or Reagent S31
12. Reaction with Asymmetric Diarylamine S32
13. Synthesis of Oxazolidinone via Photoexcitation of α-Ketoacylphosphonium S33–S34
14. Reactions in the presence of Triplet Quenchers S34
15. Application to Synthesis of Flumioxazine S35–S37

■ **References** ■ S38
■ **NMR Spectra** ■ S39–S166

1. Instrumentation and Chemicals

NMR spectra were recorded on a Bruker AVANCE NEO 400N spectrometer, operating at 400 MHz for ^{1}H NMR, 100.6 MHz for ^{13}C NMR. Chemical shift values for ^{1}H and ^{13}C are referenced to Me_4Si and the residual solvent resonances, respectively. Chemical shifts were reported in δ ppm. Mass spectra were obtained with JMS-T100TD (DART). TLC analyses were performed on commercial glass plates bearing 0.25-mm layer of Merck Silica gel 60F_{254}. Silica gel (Wakosil® 60, 64~210 µm) was used for column chromatography. Biotage Selekt was used for purification. IR spectra were measured with a Thermo Scientific iD7 ATR Accessory for the Thermo Scientific Nicolet iS5 FT-IR Spectrometer. Melting points were measured on a Stanford Research Systems MPA100. UV-Vis absorption spectra were recorded on a Shimadzu UV-1900. Fluorescence spectra were recorded on a Shimadzu RF-6000. Kessil PR160L 440 nm (highest blue and intensity setting) was used as a light source.

All reactions were carried out under nitrogen or argon atmosphere. Materials were obtained from commercial suppliers or prepared according to standard procedures unless otherwise noted. Dichloromethane and acetonitrile were purchased from FUJIFILM Wako Pure Chemical Co., stored under nitrogen, and used as received. Tricyclohexylphosphine was purchased from BLD Pharmatech Ltd., stored under nitrogen, and used as received. Oxalyl chloride was purchased from Tokyo Chemical Industry Co., stored under nitrogen, and used as received. Anilines **1i**, **1r**, **1s**, **1t**, **1u**, **1y, 1z** and **S1** were purchased from Tokyo Chemical Industry Co., stored under nitrogen, and used as received. Aniline **1k** was purchased from Angene Chemical Co., stored under nitrogen, and used as received. Aniline **1g** was purchased from Oakwood Chemical Co., stored under nitrogen, and used as received. Aniline **1x** was purchased from Sigma-Aldrich Co., stored under nitrogen, and used as received. Anilines **1a-D**, **1b**, **1c**, **1d**, **1e**, **1f**, **1h**, **1j**, **1l**, **1n** and **1o** were prepared by the reported procedure.[1] Anilines **1v** was prepared by the reported procedure.[2] Anilines **1w** was prepared by the reported procedure.[3]

2. Procedures for Light-Driven Ortho-Selective Oxygenation of Anilines

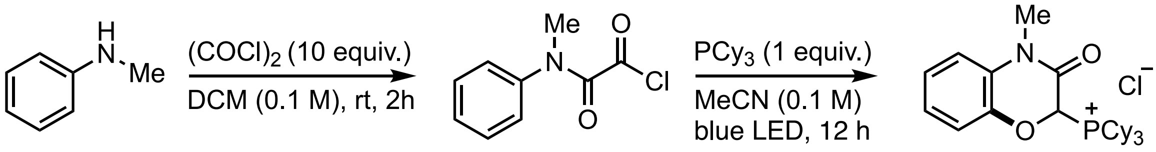

1a **1a-1** **2a**

Procedure A (1.0 mmol scale). An oven-dried vial (20 mL) with a stirring bar was placed under N_2 atmosphere by three cycles of evacuating and N_2 backfilling and charged with oxalyl chloride (857.6 mg, 1.0 mmol, 10.0 equiv.) in dry DCM (5.0 mL). Methylaniline **1a** (107.2 mg, 1.0 mmol, 1.0 equiv.) was added in dry DCM (5.0 mL) in one shot with vigorous stirring at room temperature. The mixture was stirred at room temperature for 2 hours and excess amounts of oxalyl chloride were removed by evacuation. To the reaction mixture was then added MeCN (10 mL) and tricyclohexylphosphine (280.4 mg, 1.0 mmol, 1.0 equiv.) in the glove box. The vial was brought outside the glove box and stirred for 1 h. The reaction was stirred and irradiated with 440 nm blue LED for 12 h with a cooling fan to keep the temperature at approximately 40 °C (Figure S1). The solvent was removed by evacuation. The resultant material was dissolved in a minimal quantity of THF. Hexane was added to the solution under sonication to form a precipitate. The precipitate was collected by filtration, washed with a small amount of THF, and dried under vacuum for 30 min to afford **2a** (411.2 mg, 0.86 mmol, 86% isolated yield) as a white solid.

Procedure B (0.2 mmol scale). α-Ketoacyl chloride was prepared according to the same procedure as above. In a glovebox, to an oven-dried vial with a stirring bar was added α-ketoacyl chloride (0.2 mmol, 1.0 equiv.), MeCN (2 mL) and tricyclohexylphosphine (56.1 mg, 0.2 mmol, 1.0 equiv.). The vial was brought outside the glove box and stirred for 1 h. The reaction was stirred and irradiated with 440 nm blue LED for 12 h with a cooling fan to keep the temperature at approximately 40 °C (Figure S1). The solvent was removed by evacuation. The resultant material was dissolved in a minimal quantity of THF. Hexane was added to the solution under sonication to form a precipitate. The precipitate was collected by filtration, washed with a small amount of THF, and dried under vacuum for 30 min to afford a white solid.

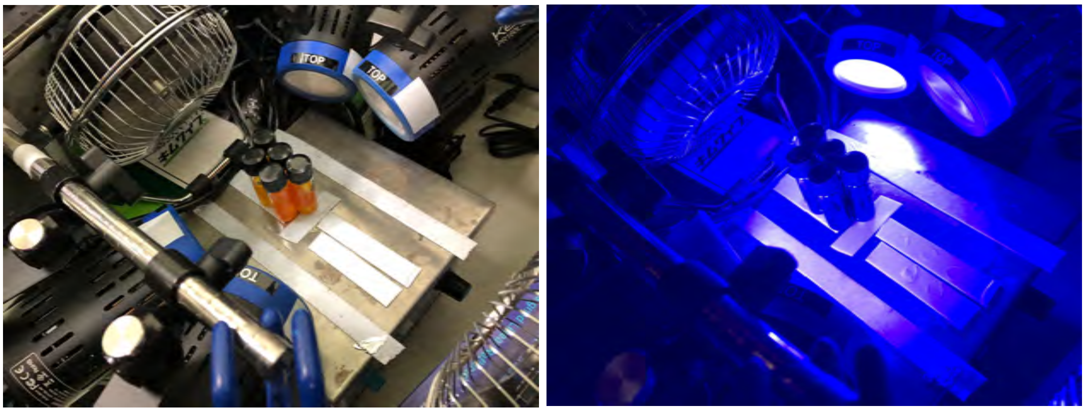

Figure S1. Light set up

3. Characterization Data for Oxygenated Products

Tricyclohexyl(4-methyl-3-oxo-3,4-dihydro-2*H*-benzo[*b*][1,4]oxazin-2-yl)phosphonium chloride (2a)

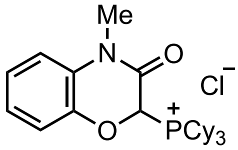

The reaction was carried out according to procedure A. The product **2a** was purified by recrystallization by sonication of a THF, hexane solution. (Fig. 2, 411.2 mg, 0.86 mmol, 86% isolated yield.). White solid. **M.p.** 237–243 °C (decomp.). **IR** (neat) 520, 765, 1056, 1318, 1473, 1502, 1665, 2752, 2851, 2920 cm $^{-1}$. **^{1}H NMR** (400 MHz, $CDCl_3$) δ 7.17 (m, 1H), 7.12–7.04 (m, 3H), 6.85 (d, J = 7.2 Hz, 1H), 3.45 (s, 3H), 3.18 (qt, J = 12.8, 2.0 Hz, 3H), 2.23–2.21 (m, 6H), 1.95–1.92 (m, 6H), 1.82 (d, J = 12.8 Hz, 3H), 1.78–1.64 (m, 6H), 1.49 (q, J = 12.8 Hz, 6H), 1.35–1.25 (m, 3H). **^{13}C NMR** (100.6 MHz, $CDCl_3$) δ 161.3, 143.6 (d, $J_{C–P}$ = 8.3 Hz), 128.4, 124.9, 124.7, 117.0, 115.7, 70.9 (d, $J_{C–P}$ = 59.8 Hz), 31.7 (d, $J_{C–P}$ = 35.3 Hz), 29.1, 27.7 (d, $J_{C–P}$ = 3.7 Hz), 27.7 (d, $J_{C–P}$ = 3.4 Hz), 26.7 (d, $J_{C–P}$= 12.3 Hz), 26.7 (d, $J_{C–P}$ = 12.3 Hz), 25.5. **^{31}P NMR** (162 MHz, $CDCl_3$) δ 36.5. **HRMS–ESI** (*m/z*): [M–Cl]$^+$ calcd for $C_{27}H_{41}NO_2P$; 442.2869, found 442.2870.

Tricyclohexyl(4,7-dimethyl-3-oxo-3,4-dihydro-2*H*-benzo[*b*][1,4]oxazin-2-yl)phosphonium chloride (2b)

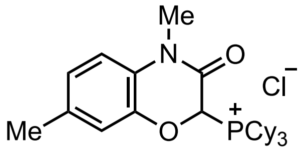

The reaction was carried out according to procedure B. The product **2b** was purified by recrystallization by sonication of a THF, hexane solution. (Fig. 2, entry 13; 65.9 mg, 0.13 mmol, 67% isolated yield.). White solid. **M.p.** 228–232 °C (decomp.). **IR** (neat) 753, 1171, 1298, 1387, 1417, 1511, 1670, 2838, 2853, 2929 cm $^{-1}$. **^{1}H NMR** (400 MHz, $CDCl_3$) δ 6.96 (s, 2H), 6.85 (s, 1H), 6.68 (d, J = 6.8 Hz, 1H), 3.42 (s, 3H), 3.17 (q, J = 12.8 Hz, 3H), 2.33 (s, 3H), 2.22–2.20 (m, 6H), 1.96–1.93 (m, 6H), 1.82 (d, J = 12.8 Hz, 3H), 1.83–1.64 (m, 6H), 1.50 (q, J = 12.8 Hz, 6H), 1.35–1.25 (m, 3H). **^{13}C NMR** (100.6 MHz, $CDCl_3$) δ 160.9, 143.2 (d, $J_{C–P}$ = 8.5 Hz), 135.3, 125.9, 125.2, 117.4, 115.5, 70.8 (d, $J_{C–P}$ = 60.0 Hz), 31.7 (d, $J_{C–P}$ = 35.5 Hz), 29.0, 27.7 (d, $J_{C–P}$ = 3.9 Hz), 27.6 (d, $J_{C–P}$ = 3.9 Hz), 26.7 (d, $J_{C–P}$= 12.4 Hz), 26.7 (d, $J_{C–P}$ = 12.4 Hz), 25.5, 20.8. **^{31}P NMR** (162 MHz, $CDCl_3$) δ 36.4. **HRMS–ESI** (*m/z*): [M–Cl]$^+$ calcd for $C_{28}H_{43}NO_2P$; 456.3026, found 456.3027.

{7-(*tert*-Butyl)-4-methyl-3-oxo-3,4-dihydro-2*H*-benzo[*b*][1,4]oxazin-2-yl}tricyclohexylphosphonium chloride (2c)

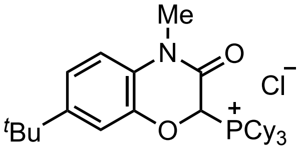

The reaction was carried out according to procedure B and the reaction time was modified to 48 h. The product **2c** was purified by recrystallization by sonication of a THF, Et_2O solution. (Fig. 2; 61.0 mg, 0.11 mmol, 57% isolated yield.). White solid. **M.p.** 216–225 °C (decomp.). **IR** (neat) 1061, 1078, 1268, 1290, 1402, 1444, 1485, 1666, 2848, 2931 cm $^{-1}$. **^{1}H NMR** (400 MHz, $CDCl_3$) δ 7.20 (d, J = 8.4 Hz, 1H), 7.07 (d, J = 8.4 Hz, 1H), 6.97 (s, 1H), 6.38 (d, J = 6.8 Hz, 1H), 3.45 (s, 3H), 3.14 (q, J = 12.0 Hz, 3H), 2.21–2.18 (m, 6H), 1.98–1.94 (m, 6H), 1.82 (d, J = 12.4 Hz, 3H), 1.83–1.67 (m, 6H), 1.50 (q, J = 12.4 Hz, 6H), 1.35–1.22 (m, 12H). **^{13}C NMR** (100.6 MHz, $CDCl_3$) δ 160.5, 148.8, 142.7 (d, $J_{C–P}$ = 8.0 Hz), 125.4, 121.6, 115.3, 113.6, 70.4 (d, $J_{C–P}$ = 60.0 Hz), 34.4, 31.5 (d, $J_{C–P}$ = 35.1 Hz), 31.0, 29.0, 27.5 (d, $J_{C–P}$ = 4.5 Hz), 27.5 (d, $J_{C–P}$ = 4.4 Hz), 26.6 (d, $J_{C–P}$= 12.3 Hz), 26.5 (d, $J_{C–P}$= 12.3 Hz), 25.3. **^{31}P NMR** (162 MHz, $CDCl_3$) δ 36.6. **HRMS–ESI** (*m/z*): $[M–Cl]^+$ calcd for $C_{31}H_{49}NO_2P$; 498.3495, found 498.3494.

Tricyclohexyl(4,6,8-trimethyl-3-oxo-3,4-dihydro-2*H*-benzo[*b*][1,4]oxazin-2-yl)phosphonium chloride (2d)

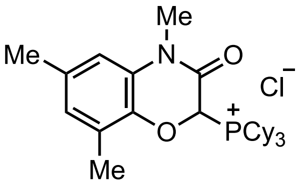

The reaction was carried out according to procedure B. The product **2d** was purified by recrystallization by sonication of a THF, hexane solution. (Fig. 2; 80.0 mg, 0.16 mmol, 79% isolated yield.). White solid. **M.p.** 232–236 °C (decomp.). **IR** (neat) 769, 1231, 1341, 1380, 1446, 1473, 1666, 2766, 2853, 2927 cm $^{-1}$. **^{1}H NMR** (400 MHz, $CDCl_3$) δ 6.77 (s, 1H), 6.72 (s, 1H), 6.54 (d, J = 6.8 Hz, 1H), 3.41 (s, 3H), 3.20 (q, J = 12.8 Hz, 3H), 2.31 (s, 3H), 2.29 (s, 3H), 2.26–2.19 (m, 6H), 1.96–1.93 (m, 6H), 1.83–1.70 (m, 9H), 1.50 (q, J = 12.8 Hz, 6H), 1.35–1.24 (m, 3H). **^{13}C NMR** (100.6 MHz, $CDCl_3$) δ 161.5, 140.0 (d, $J_{C–P}$ = 8.7 Hz), 133.8, 127.9, 127.3, 126.0, 113.9, 70.9 (d, $J_{C–P}$ = 60.9 Hz), 31.7 (d, $J_{C–P}$ = 35.6 Hz), 29.2, 27.6 (d, $J_{C–P}$ = 4.2 Hz), 26.7 (d, $J_{C–P}$= 12.3 Hz), 25.4, 21.0, 16.3. **^{31}P NMR** (162 MHz, $CDCl_3$) δ 36.5. **HRMS–ESI** (*m/z*): $[M–Cl]^+$ calcd for $C_{29}H_{45}NO_2P$; 470.3182, found 470.3180.

Tricyclohexyl(7-fluoro-4-methyl-3-oxo-3,4-dihydro-2*H*-benzo[*b*][1,4]oxazin-2-yl)phosphonium chloride (2e)

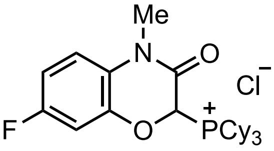

The reaction was carried out according to procedure A. The product **2e** was purified by recrystallization by sonication of a THF, hexane solution. (Fig. 2; 74.4mg, 0.15 mmol, 75% isolated yield.). White solid. **M.p.** 232–237 °C (decomp.). **IR** (neat) 742, 922, 1184, 1154, 1421, 1508, 1670, 2855, 2932 cm $^{-1}$. **^{1}H NMR** (400 MHz, $CDCl_3$) δ 7.33 (d, J = 7.2 Hz, 1H), 7.07 (m, 1H), 6.87 (m, 1H), 6.79 (m, 1H), 3.44 (s, 3H), 3.08 (q, J = 12.8 Hz, 3H), 2.22–2.20 (m, 6H), 1.96–1.94 (m, 6H),

1.82 (d, J = 12.0 Hz, 3H), 1.70 (q, J = 12.6 Hz, 6H), 1.46 (q, J = 12.8 Hz, 6H), 1.35–1.25 (m, 3H). **^{13}C NMR** (100.6 MHz, $CDCl_3$) δ 160.7, 159.0 (d, J_{C-F} = 246.0 Hz), 144.1 (dd, J = 11.6, 8.9 Hz), 124.9 (d, J_{C-F} = 3.2 Hz), 116.5 (d, J_{C-F} = 9.5 Hz), 111.0 (d, J_{C-F} = 22.8 Hz), 105.1 (d, J_{C-F} = 26.6 Hz), 71.0 (d, J_{C-P} = 60.4 Hz), 31.7 (d, J_{C-P} = 35.5 Hz), 29.2, 27.6 (d, J_{C-P} = 3.7 Hz, ×2C), 26.7 (d, J_{C-P}= 12.3 Hz), 26.7 (d, J_{C-P} = 12.3 Hz), 25.4. **^{19}F NMR** (376.5 MHz, $CDCl_3$) δ–116.1. **^{31}P NMR** (162 MHz, $CDCl_3$) δ 36.4. **HRMS–ESI** (*m/z*): $[M–Cl]^+$ calcd for $C_{27}H_{40}NO_2P$; 460.2775, found460.2778.

(7-Chloro-4-methyl-3-oxo-3,4-dihydro-2*H*-benzo[*b*][1,4]oxazin-2-yl)tricyclohexylphosphonium chloride (2f)

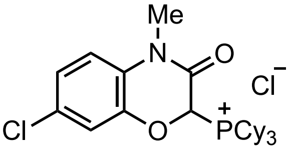

The reaction was carried out according to procedure B. The product **2f** was purified by recrystallization by sonication of a THF, hexane solution. (Fig. 2; 77.9 mg, 0.15 mmol, 76% isolated yield.). White solid. **M.p.** 224–232 °C (decomp.). **IR** (neat) 732, 890, 1139, 1286, 1382, 1444, 1500, 1668, 2852, 2929 cm $^{-1}$. **^{1}H NMR** (400 MHz, $CDCl_3$) δ 7.52 (m, 1H), 7.11 (dd, J = 8.4, 1.6 Hz, 1H), 7.03–7.01 (m, 2H), 3.43 (s, 3H), 3.07 (q, J = 12.4 Hz, 3H), 2.24–2.17 (m, 6H), 1.97–1.94 (m, 6H), 1.82 (d, J = 12.4 Hz, 3H), 1.70 (q, J = 12.4 Hz, 6H), 1.45 (q, J = 12.4 Hz, 6H), 1.35–1.23 (m, 3H). **^{13}C NMR** (100.6 MHz, $CDCl_3$) δ 161.0, 143.9 (d, J_{C-P} = 9.2 Hz), 129.6, 127.4, 124.4, 117.2, 116.5, 71.2 (d, J_{C-P} = 60.4 Hz), 31.7 (d, J_{C-P} = 35.6 Hz), 29.2, 27.7 (d, J_{C-P} = 3.4 Hz), 27.7 (d, J_{C-P} = 3.5 Hz), 26.8 (d, J_{C-P}= 12.4 Hz), 26.7 (d, J_{C-P} = 12.4 Hz), 25.5. **^{31}P NMR** (162 MHz, $CDCl_3$) δ 36.4. **HRMS–ESI** (*m/z*): $[M–Cl]^+$ calcd for $C_{27}H_{40}ClNO_2P$; 476.2480, found476.2473.

(7-Bromo-4-methyl-3-oxo-3,4-dihydro-2*H*-benzo[*b*][1,4]oxazin-2-yl)tricyclohexylphosphonium chloride (2g)

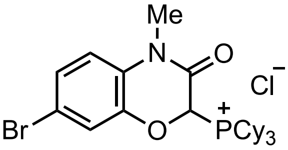

The reaction was carried out according to procedure A. The product **2g** was purified by recrystallization by sonication of a THF, hexane solution. (Fig. 2; 389.9 mg, 0.70 mmol, 70% isolated yield.). White solid. **M.p.** 248–250 °C (decomp.). **IR** (neat) 868, 1065, 1312, 1383, 1423, 1463, 1494, 1660, 1673, 2845, 2933 cm $^{-1}$. **^{1}H NMR** (400 MHz, $CDCl_3$) δ 7.47 (d, J = 6.8 Hz, 1H), 7.26 (dd, J = 8.4, 2.0 Hz, 1H), 7.16 (d, J = 2.0 Hz, 1H), 6.98 (d, J = 8.4 Hz, 1H), 3.42 (s, 3H), 3.07 (q, J = 12.8 Hz, 3H), 2.23–2.16 (m, 6H), 1.96–1.94 (m, 6H), 1.83–1.80 (m, 3H),1.70 (q, J = 12.8, 6H), 1.45 (q, J = 12.8 Hz, 6H), 1.35–1.25 (m, 3H). **^{13}C NMR** (100.6 MHz, $CDCl_3$) δ 161.0, 144.0 (d, J_{C-P} = 8.9 Hz), 127.8, 127.4, 119.9, 116.9, 116.7, 71.1 (d, J_{C-P} = 60.4 Hz), 31.7 (d, J_{C-P} = 35.6 Hz), 29.1, 27.7 (d, J_{C-P} = 3.4 Hz), 27.6 (d, J_{C-P} = 3.5 Hz), 26.8 (d, J_{C-P}= 12.3 Hz), 26.7 (d, J_{C-P} = 12.3 Hz), 25.4. **^{31}P NMR** (162 MHz, $CDCl_3$) δ 36.2. **HRMS–ESI** (*m/z*): $[M–Cl]^+$ calcd for $C_{27}H_{40}BrNO_2P$; 520.1975, found 520.1996.

Tricyclohexyl(6,7,8-trifluoro-4-methyl-3-oxo-3,4-dihydro-2*H*-benzo[*b*][1,4]oxazin-2-yl)phosphonium chloride (2h)

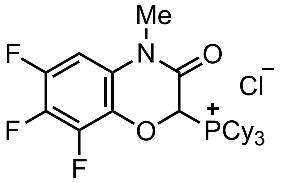

The reaction was carried out according to procedure A. The product **2h** was purified by recrystallization by sonication of a THF, hexane solution. (Fig. 2, entry 13; 357.9 mg, 0.67 mmol, 67% isolated yield.). White solid. **M.p.** 230–234 °C (decomp.). **IR** (neat) 772, 1103, 1361, 1500, 1523, 1679, 2791, 2850, 2938 cm $^{-1}$. **^{1}H NMR** (400 MHz, $CDCl_3$) δ 8.05 (m, 1H), 6.74 (m, 1H), 3.39 (s, 3H), 2.94 (q, J = 12.8 Hz, 3H), 2.29–2.15 (m, 6H), 1.97–1.95 (m, 6H), 1.82–1.68 (m, 9H), 1.45–1.26 (m, 9H). **^{13}C NMR** (100.6 MHz, $CDCl_3$) δ 161.2, 146.6 (ddd, J = 244.5, 10.6, 2.8 Hz), 140.7 (ddd, J = 250.3, 13.3, 4.8 Hz), 136.3 (ddd, J = 250.3, 16.3, 13.3 Hz), 129.6 (m), 125.5 (d, J = 9.6 Hz), 98.7 (d, J = 22.0 Hz), 71.1 (d, $J_{C\text{–}P}$ = 61.3 Hz), 31.4 (d, $J_{C\text{–}P}$ = 35.5 Hz), 29.4, 27.2, 26.3 (d, $J_{C\text{–}P}$ = 12.4 Hz), 24.9. **^{19}F NMR** (376.5 MHz, $CDCl_3$) δ –138.9 (d, J= 22.9 Hz), –153.0 (d, J= 22.9 Hz), –163.5 (t, J= 22.9 Hz). **^{31}P NMR** (162 MHz, $CDCl_3$) δ 37.1. **HRMS–ESI** (*m/z*): [M–Cl]$^+$ calcd for $C_{27}H_{38}F_3NO_2P$; 496.2587, found 496.2588.

Tricyclohexyl(4-methyl-3-oxo-7-(trifluoromethyl)-3,4-dihydro-2*H*-benzo[*b*][1,4]oxazin-2-yl)phosphonium chloride (2i)

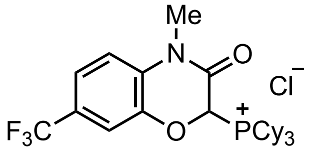

The reaction was carried out according to procedure A. The product **2i** was purified by recrystallization by sonication of a THF, hexane solution. (Fig. 2, entry 13; 436.8 mg, 0.80 mmol, 80% isolated yield.). White solid. **M.p.** 233–235 °C (decomp.). **IR** (neat) 904, 1123, 1144, 1167, 1332, 1440, 1671, 2745, 2853, 2920 cm $^{-1}$. **^{1}H NMR** (400 MHz, $CDCl_3$) δ 7.70 (d, J = 6.8 Hz, 1H), 7.41 (m, 1H), 7.22 (m, 2H), 3.48 (s, 3H), 3.03 (q, J= 12.8 Hz, 3H), 2.24–2.17 (m, 6H), 1.98–1.95 (m, 6H), 1.82 (d, J= 12.0 Hz, 3H), 1.75–1.66 (m, 6H), 1.44 (q, J= 12.8 Hz, 6H), 1.36–1.26 (m, 3H). **^{13}C NMR** (100.6 MHz, $CDCl_3$) δ 161.4, 143.2 (d, $J_{C\text{–}P}$ = 32.4 Hz), 131.5, 126.5 (q, $J_{C\text{–}F}$ = 33.6 Hz), 123.3 (q, $J_{C\text{–}F}$ = 271.9 Hz), 121.5 (q, $J_{C\text{–}F}$ = 3.7 Hz), 116.0, 113.9 (q, $J_{C\text{–}F}$ = 3.6 Hz), 71.0 (d, $J_{C\text{–}P}$ = 60.7 Hz), 31.7 (d, $J_{C\text{–}P}$ = 35.6 Hz), 29.2, 27.6 (d, $J_{C\text{–}P}$ = 4.3 Hz), 27.6 (d, $J_{C\text{–}P}$ = 4.3 Hz), 26.8 (d, $J_{C\text{–}P}$= 12.3 Hz), 26.7 (d, $J_{C\text{–}P}$ = 12.3 Hz), 25.4. **^{19}F NMR** (376.5 MHz, $CDCl_3$) δ –62.1. **^{31}P NMR** (162 MHz, $CDCl_3$) δ 36.5. **HRMS–ESI** (*m/z*): [M–Cl]$^+$ calcd for $C_{28}H_{40}F_3NO_2P$; 510.2743, found 510.2749.

(7-Acetyl-4-methyl-3-oxo-3,4-dihydro-2*H*-benzo[*b*][1,4]oxazin-2-yl)tricyclohexylphosphonium chloride (2j)

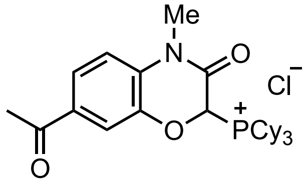

The reaction was carried out according to procedure A. The product **2j** was purified by recrystallization by sonication of a MeCN, hexane solution. (Fig. 2; 410.9 mg, 0.79 mmol, 79% isolated yield.). White solid. **M.p.** 226–229 °C (decomp.). **IR** (neat) 514, 895, 1284, 1307, 1424, 1442, 1667, 1667, 2850, 2940 cm $^{-1}$. **^{1}H NMR** (400 MHz, $CDCl_3$) δ 7.76–7.73 (m, 2H), 7.61 (s, 1H), 7.18 (d, J = 8.4 Hz, 1H), 3.49 (s, 3H), 3.03 (q, J = 12.8 Hz, 3H), 2.58 (s, 3H), 2.25–2.19 (m, 6H), 1.98–1.96 (m, 6H), 1.82 (d, J = 12.4 Hz, 3H), 1.72 (q, J = 11.6 Hz, 6H), 1.44 (q, J = 12.8 Hz, 6H), 1.35–1.26 (m, 3H). **^{13}C NMR** (100.6 MHz, $CDCl_3$) δ 196.2, 161.7, 143.5 (d, J_{C-P} = 8.6 Hz), 133.4, 132.6, 125.3, 116.2, 115.5, 70.9 (d, J_{C-P} = 59.3 Hz), 31.7 (d, J_{C-P} = 35.7 Hz), 29.4, 27.6 (d, J_{C-P} = 4.4 Hz), 27.6 (d, J_{C-P} = 4.3 Hz), 26.8 (d, J_{C-P}= 12.3 Hz), 26.8 (d, J_{C-P}= 12.3 Hz), 26.5, 25.4. **^{31}P NMR** (162 MHz, $CDCl_3$) δ 36.2. **HRMS–ESI** (*m/z*): $[M–Cl]^+$ calcd for $C_{29}H_{43}NO_3P$; 484.2975, found 484.2974.

Tricyclohexyl{7-(methoxycarbonyl)-4-methyl-3-oxo-3,4-dihydro-2*H*-benzo[*b*][1,4]oxazin-2-yl}phosphonium chloride (2k)

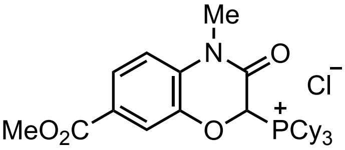

The reaction was carried out according to procedure A. The product **2k** was purified by recrystallization by sonication of a THF, hexane solution. (Fig. 2; 423.5 mg, 0.79 mmol, 79% isolated yield.). White solid. **M.p.** 187–192 °C (decomp.). **IR** (neat) 743, 1132, 1256, 1288, 1381, 1440, 1677, 1715, 2854, 2932 cm $^{-1}$. **^{1}H NMR** (400 MHz, $CDCl_3$) δ 7.83 (m, 1H), 7.70 (d, J = 6.8 Hz, 1H), 7.65 (d, J = 1.2 Hz, 1H), 7.12 (d, J = 8.4 Hz, 1H), 3.91 (s, 3H), 3.47 (s, 3H), 3.07 (q, J = 12.8 Hz, 3H), 2.27–2.17 (m, 6H), 1.97–1.95 (m, 6H), 1.82 (d, J = 12.8 Hz, 3H), 1.71 (q, J = 11.6 Hz, 6H), 1.45 (q, J = 12.8 Hz, 6H), 1.36–1.24 (m, 3H). **^{13}C NMR** (100.6 MHz, $CDCl_3$) δ 165.7, 161.8, 143.3 (d, J_{C-P} = 9.0 Hz), 132.5, 126.4, 126.1, 117.9, 115.4, 71.1 (d, J_{C-P} = 60.8 Hz), 52.3, 31.8 (d, J_{C-P} = 35.8 Hz), 29.3, 27.7 (d, J_{C-P} = 4.6 Hz), 27.7 (d, J_{C-P} = 4.8 Hz), 26.8 (d, J_{C-P}= 12.3 Hz), 26.8 (d, J_{C-P}= 12.3 Hz), 25.5. **^{31}P NMR** (162 MHz, $CDCl_3$) δ 36.3. **HRMS–ESI** (*m/z*): $[M–Cl]^+$ calcd for $C_{29}H_{43}NO_4P$; 500.2924, found 500.2921.

Tricyclohexyl(7-methoxy-4-methyl-3-oxo-3,4-dihydro-2*H*-benzo[*b*][1,4]oxazin-2-yl)phosphonium chloride (2l)

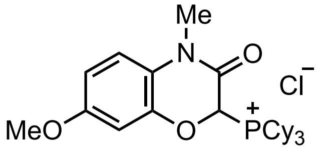

The reaction was carried out according to procedure B and the reaction time was modified to 84 h. The product **2l** was purified by recrystallization by sonication of a THF, Et_2O solution. (Fig. 2; 46.1 mg, 0.09 mmol, 45% isolated yield.). White solid. **M.p.** 230–237 °C (decomp.). **IR** (neat) 1131, 1165, 1300, 1395, 1447, 1513, 1666, 2855, 2934, 3391. cm $^{-1}$. **^{1}H NMR** (400 MHz, $CDCl_3$) δ 7.02 (d, J = 8.8 Hz, 1H), 6.71 (dd, J = 8.8, 2.8 Hz, 1H), 6.64 (d, J = 6.4 Hz, 1H), 6.62 (d, J = 2.8 Hz, 1H), 3.81 (s, 3H), 3.42 (s, 3H), 3.13 (q, J = 12.8 Hz, 3H), 2.20–2.18 (m, 6H), 1.96–1.93 (m, 6H), 1.82 (d, J = 12.4 Hz, 3H), 1.72 (quin, J = 12.0 Hz, 6H), 1.49 (q, J = 12.8 Hz, 6H), 1.35–1.25 (m, 3H). **^{13}C NMR** (100.6 MHz, $CDCl_3$) δ 160.3, 156.9, 144.1 (d, J_{C-P} = 8.7 Hz), 121.7, 116.3, 109.4, 103.6, 70.7 (d, J_{C-P} = 59.3 Hz), 55.8, 31.7 (d, J_{C-P} = 35.5 Hz), 29.1, 27.6 (×2C), 26.7 (d, J_{C-P}= 12.5 Hz), 26.7 (d, J_{C-P}= 12.5 Hz), 25.5. **^{31}P NMR** (162 MHz, $CDCl_3$) δ 37.1. **HRMS–ESI** (*m/z*): $[M–Cl]^+$ calcd for $C_{28}H_{43}NO_3P$; 472.2975, found 472.2974.

Tricyclohexyl(4-methyl-3-oxo-7-phenoxy-3,4-dihydro-2*H*-benzo[*b*][1,4]oxazin-2-yl)phosphonium chloride (2m)

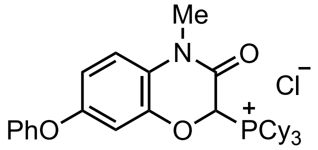

The reaction was carried out according to procedure B and the reaction time was modified to 42 h. The product **2m** was purified by recrystallization by sonication of a THF, Hexane solution. (Fig. 2; 70.7 mg, 0.12 mmol, 62% isolated yield.). White solid. **M.p.** 220–228 °C (decomp.). **IR** (neat) 742, 1210, 1447, 1506, 1669, 2854, 2932 cm $^{-1}$. **^{1}H NMR** (400 MHz, $CDCl_3$) δ 7.36 (t, J = 7.8 Hz, 2H), 7.14 (t, J = 7.2 Hz, 1H), 7.03–7.01 (m, 4H), 6.75 (dd, J = 8.4, 2,4 Hz, 1H), 6.71 (d, J = 2.4 Hz, 1H), 3.43 (s, 3H), 3.11 (q, J = 12.6 Hz, 3H), 2.21–2.20 (m, 6H), 1.94–1.92 (m, 6H), 1.80 (d, J = 12.6 Hz, 3H), 1.70–1.67 (m, 6H), 1.46 (q, J = 12.6 Hz, 6H), 1.31–1.25 (m, 3H). **^{13}C NMR** (100.6 MHz, $CDCl_3$) δ 160.6, 156.4, 154.2, 144.3 (d, J_{C-P} = 8.7 Hz), 129.9, 123.9, 123.9, 119.0, 116.4, 114.0, 107.9, 71.0 (d, J_{C-P} = 59.5 Hz), 31.7 (d, J_{C-P} = 35.5 Hz), 29.2, 27.7 (d, J_{C-P}= 4.8 Hz, ×2C), 26.7 (d, J_{C-P}= 12.5 Hz), 26.7 (d, J_{C-P}= 12.5 Hz), 25.5. **^{31}P NMR** (162 MHz, $CDCl_3$) δ 37.1. **HRMS–ESI** (*m/z*): $[M–Cl]^+$ calcd for $C_{33}H_{45}NO_3P$; 534.3132, found 534.3133.

Tricyclohexyl(4,6-dimethyl-3-oxo-3,4-dihydro-2*H*-benzo[*b*][1,4]oxazin-2-yl)phosphonium chloride (2n, major)
Tricyclohexyl(4,8-dimethyl-3-oxo-3,4-dihydro-2*H*-benzo[*b*][1,4]oxazin-2-yl)phosphonium chloride (2n, minor)

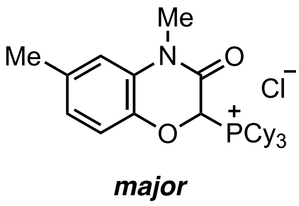

major

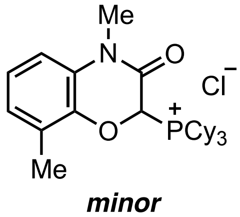

minor

The reaction was carried out according to procedure B. The product **2n** was purified by recrystallization by sonication of a THF, hexane solution. (Fig. 2; 80.7 mg, 0.16 mmol, 82% isolated

yield.). White solid. **M.p.** 185–242 °C (decomp.). **IR** (neat) 725, 923, 1229, 1375, 1446, 1482, 1668, 2854, 2930 cm $^{-1}$. Signals for two regioisomer (6:4) were given: **^{1}H NMR** (400 MHz, $CDCl_3$) **Major regioisomer:** δ 6.97–6.88 (m, 3H), 6.53 (d, J = 6.8 Hz, 1H), 3.44 (s, 3H), 3.26–3.08 (m, 3H), 2.36 (s, 3H), 2.20–2.18 (m, 6H), 1.96–1.94 (m, 6H), 1.83–1.64 (m, 9H), 1.49 (q, J = 12.8 Hz, 6H), 1.35–1.21 (m, 3H). **Minor regioisomer:** δ 7.07 (t, J = 8.0 Hz, 1H), 6.97–6.88 (m, 2H), 6.57 (d, J = 6.8 Hz, 1H), 3.44 (s, 3H), 3.26–3.08 (m, 3H), 2.33 (s, 3H), 2.20–2.18 (m, 6H), 1.96–1.94 (m, 6H), 1.83–1.64 (m, 9H), 1.49 (q, J = 12.8 Hz, 6H), 1.35–1.21 (m, 3H). Signals for two regioisomer were given: **^{13}C NMR** (100.6 MHz, $CDCl_3$) δ 161.3, 161.2, 141.9 (d, J_{C-P} = 8.1 Hz), 141.2 (d, J_{C-P} = 8.5 Hz), 134.6, 128.1, 127.9, 126.8, 126.4, 125.2, 124.0, 116.5, 116.2, 113.4, 70.7 (d, J_{C-P} = 60.7 Hz), 70.6 (d, J_{C-P} = 59.9 Hz), 31.7 (d, J_{C-P} = 35.5 Hz), 31.6 (d, J_{C-P} = 35.4 Hz), 29.2, 29.0, 27.6 (d, J_{C-P} = 4.3 Hz), 27.6 (d, J_{C-P} = 4.3 Hz), 26.6 (d, J_{C-P} = 12.4 Hz), 26.6 (d, J_{C-P} = 12.3 Hz), 25.4, 25.4, 21.1, 16.3. Signals for the minor rotamer were enclosed in parenthesis: **^{31}P NMR** (162 MHz, $CDCl_3$) δ (36.7), 36.4. **HRMS–ESI** (*m/z*): $[M–Cl]^+$ calcd for $C_{28}H_{43}NO_2P$; 456.3026, found 456.3026.

(6-Chloro-4-methyl-3-oxo-3,4-dihydro-2*H*-benzo[*b*][1,4]oxazin-2-yl)tricyclohexylphosphonium chloride (2o, major)
(8-Chloro-4-methyl-3-oxo-3,4-dihydro-2*H*-benzo[*b*][1,4]oxazin-2-yl)tricyclohexylphosphonium chloride (2o, minor)

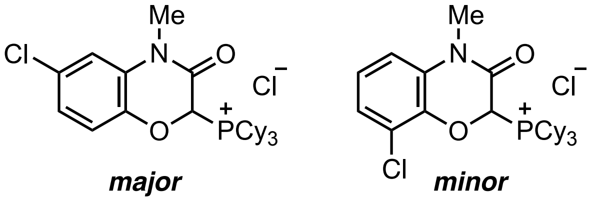

The reaction was carried out according to procedure B. The product **2o** was purified by recrystallization by sonication of a THF, hexane solution. (Fig. 2; 100.5 mg, 0.20 mmol, 98% isolated yield.). White solid. **M.p.** 232–236 °C (decomp.). **IR** (neat) 721, 953, 1229, 1368, 1473, 1497, 1675, 2854, 2932 cm $^{-1}$. Signals for two regioisomer (7:3) were given: **^{1}H NMR** (400 MHz, $CDCl_3$) **Major regioisomer:** δ 7.44 (d, J = 7.2 Hz, 1H), 7.04–6.98 (m, 3H), 3.41 (s, 3H), 3.07 (q, J = 12.8, 3H), 2.21–2.17 (m, 6H), 1.97–1.94 (m, 6H), 1.83–1.65 (m, 9H), 1.50–1.41 (m, 6H), 1.37–1.25 (m, 3H). **Minor regioisomer:** δ 7.21 (d, J = 6.8 Hz, 1H), 7.15–7.08 (m, 2H), 7.01 (m,1H), 3.46 (s, 3H), 3.07 (q, J = 12.8 Hz, 3H), 2.21–2.17 (m, 6H), 1.97–1.94 (m, 6H), 1.83–1.65 (m, 9H), 1.50–1.41 (m, 6H), 1.37–1.25 (m, 3H). Signals for two regioisomer were given: **^{13}C NMR** (100.6 MHz, $CDCl_3$) δ 161.4, 161.2, 142.2 (d, J_{C-P} = 8.8 Hz), 140.2 (d, J_{C-P} = 8.6 Hz), 130.1, 129.6, 129.5, 125.4, 124.6, 124.3, 122.1, 117.9, 115.8, 114.3, 71.2 (d, J_{C-P} = 61.0 Hz), 71.0 (d, J_{C-P} = 60.3 Hz), 31.9 (d, J_{C-P} = 35.5 Hz), 31.5 (d, J_{C-P} = 35.5 Hz), 29.5, 29.1, 27.7 (d, J_{C-P} = 3.5 Hz), 27.6 (d, J_{C-P} = 2.8 Hz), 27.5 (d, J_{C-P} = 4.3 Hz), 27.5 (d, J_{C-P} = 6.3 Hz), 26.7 (d, J_{C-P} = 12.3 Hz, ×2C), 26.7 (d, J_{C-P} = 12.3 Hz, ×2C), 25.4, 25.3. Signal for the minor isomer were enclosed in parenthesis: **^{31}P NMR** (162 MHz, $CDCl_3$) δ (36.9), 36.4. **HRMS–ESI** (*m/z*): $[M–Cl]^+$ calcd for $C_{27}H_{40}ClNO_2P$; 476.2480, found 476.2473.

(8-Chloro-6-fluoro-4-methyl-3-oxo-3,4-dihydro-2*H*-benzo[*b*][1,4]oxazin-2-yl)tricyclohexylphosphonium chloride (2p)
(8-Chloro-6-fluoro-4-methyl-3-oxo-3,4-dihydro-2*H*-benzo[*b*][1,4]oxazin-2-yl)tricyclohexylphosphonium chloride (2p)

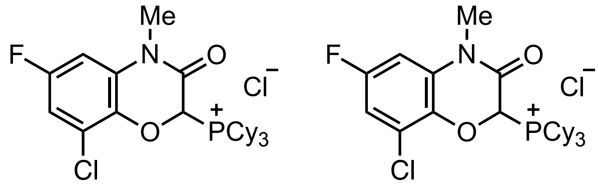

The reaction was carried out according to procedure A. The product **2p** was purified by recrystallization by sonication of a THF, Et_2O solution. (Fig. 2; 500.9 mg, 0.96 mmol, 96% isolated yield.). White solid. **M.p.** 232–242 °C (decomp.). **IR** (neat) 1171, 1272, 1335, 1371, 1446, 1485, 1503, 1606, 1685, 2853, 2926 cm $^{-1}$. Peaks for two regioisomers (1:1) were given: **^{1}H NMR** (400 MHz, $CDCl_3$) δ 7.69 (d, J = 6.8 Hz, 1H), 7.44 (d, J = 6.8 Hz, 1H), 6.92–6.80 (m, 4H), 3.44 (s, 3H), 3.43 (s, 3H), 3.07–2.96 (m, 6H), 2.23–2.17 (m, 12H), 1.96–1.95 (m, 12H), 1.82–1.59 (m, 18H), 1.48–1.26 (m, 18H). Peaks for two regioisomers were given: **^{13}C NMR** (162.0 MHz, $CDCl_3$) δ 161.3, 161.3, 137.0 (d, J = 245.6 Hz), 150.5 (d, J = 250.4 Hz), 136.6 (dd, J = 9.6, 3.8 Hz), 131.2 (d, J = 2.9 Hz), 131.0 (dd, J = 15.3, 9.6 Hz), 130.8 (d, J = 11.6 Hz), 128.7 (d, J = 10.6 Hz), 122.2 (d, J = 12.5 Hz), 112.3 (d, J = 21.6 Hz), 111.5 (d, J = 25.9 Hz), 111.2 (d, J = 2.8 Hz), 102.3 (d, J = 27.9 Hz), 71.2 (d, J = 61.4 Hz), 71.0 (d, J = 61.4 Hz), 31.5 (d, J = 35.4 Hz), 31.4 (d, J = 35.5 Hz), 29.4, 29.2, 27.3 (m), 27.3 (m), 26.5 (d, J = 12.5 Hz), 26.5 (d, J = 11.5 Hz), 25.1. Peaks for two regioisomers were given: **^{19}F NMR** (376.5 MHz, $CDCl_3$) δ –115.0, –131.8. Peaks for two regioisomers were given: **^{31}P NMR** (162.0 MHz, $CDCl_3$) δ 37.5, 37.3. **HRMS–ESI** (*m/z*): $[M–Cl]^+$ calcd for $C_{27}H_{39}Cl_2NO_2P$; 494.2385, found 494.2386.

(7-Chloro-6-methoxy-4-methyl-3-oxo-3,4-dihydro-2*H*-benzo[*b*][1,4]oxazin-2-yl)tricyclohexylphosphonium chloride (2q, major)
(7-Chloro-8-methoxy-4-methyl-3-oxo-3,4-dihydro-2*H*-benzo[*b*][1,4]oxazin-2-yl)tricyclohexylphosphonium chloride (2q, minor)

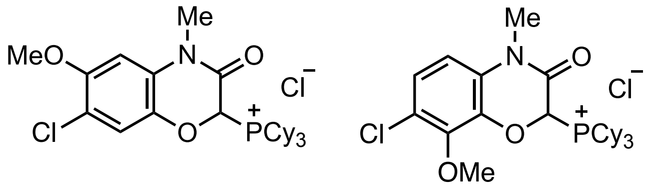

major ***minor***

The reaction was carried out according to procedure B. The product **2q** was purified by recrystallization by sonication of a THF, Et_2O solution. (Fig. 2; 76.6 mg, 0.14 mmol, 71% isolated yield.). White solid. **M.p.** 200–206 °C (decomp.). **IR** (neat) 919, 1066, 1292, 1344, 1426, 1447, 1504, 1671, 2850, 2929 cm $^{-1}$. Signals for two regioisomer (7:3) were given: **^{1}H NMR** (600 MHz, $CDCl_3$) **Major regioisomer:** δ 7.03 (s, 1H), 6.92 (d, J = 6.6 Hz, 1H), 6.80 (s, 1H), 3.96 (s, 3H), 3.52 (s, 3H), 3.03 (q, J = 12.6 Hz, 3H), 2.18–2.11 (m, 6H), 1.96–1.95 (m, 6H), 1.82 (d, J = 12.6 Hz, 3H), 1.75–1.65 (m, 6H), 1.49–1.42 (m, 6H), 1.34–1.28 (m, 3H). **Minor regioisomer:** δ 7.64 (d, J = 7.2 Hz, 1H),

7.11 (d, J = 8.4 Hz, 1H), 6.81 (d, J = 8.4 Hz, 1H), 4.01 (s, 3H), 3.41 (s, 3H), 3.03 (q, J = 12.6 Hz, 3H), 2.18–2.11 (m, 6H), 1.96–1.95 (m, 6H), 1.82 (d, J = 12.6 Hz, 3H), 1.75–1.65 (m, 6H), 1.49–1.42 (m, 6H), 1.34–1.28 (m, 3H). Signals for two regioisomer were given: **^{13}C NMR** (150.9 MHz, $CDCl_3$) **Major regioisomer:** δ 161.2, 152.0, 137.0 (d, $J_{C\text{–}P}$ = 8.8 Hz), 127.9, 124.4, 118.1, 101.0, 71.0 (d, $J_{C\text{–}P}$ = 61.4 Hz), 57.2, 31.7 (d, $J_{C\text{–}P}$ = 35.7 Hz), 29.5, 27.6 (d, $J_{C\text{–}P}$ = 4.1 Hz), 26.7 (m), 25.4. **Minor regioisomer:** δ 161.1, 145.1, 138.2 (d, $J_{C\text{–}P}$ = 8.8 Hz), 128.4, 124.3, 117.3, 111.0, 71.1 (d, $J_{C\text{–}P}$ = 61.0 Hz), 61.8, 31.9 (d, $J_{C\text{–}P}$ = 35.2 Hz), 29.2, 27.5 (d, $J_{C\text{–}P}$ = 2.1 Hz), 26.7 (m), 25.3. Signal for the minor isomer was enclosed in parenthesis: **^{31}P NMR** (243 MHz, $CDCl_3$) δ (37.6), 36.8. **HRMS–ESI** (*m/z*): $[M–Cl]^+$ calcd for $C_{28}H_{42}ClNO_3P$; 506.2585, found 506.2584.

(4-Butyl-3-oxo-3,4-dihydro-2*H*-benzo[*b*][1,4]oxazin-2-yl)tricyclohexylphosphonium chloride (2r)

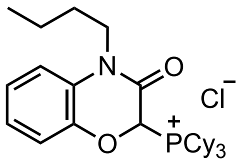

The reaction was carried out according to procedure A. The product **2r** was purified by recrystallization by sonication of a THF, hexane solution. (Fig. 2; 421.3 mg, 0.81 mmol, 81% isolated yield.). White solid. **M.p.** 224–228 °C (decomp.). **IR** (neat) 756, 1234, 1277, 1402, 1448, 1499, 1668, 2855, 2932, 3390 cm $^{-1}$. **^{1}H NMR** (400 MHz, $CDCl_3$) δ 7.17 (m, 1H), 7.13–7.05 (m, 3H), 6.51 (d, J = 6.8 Hz, 1H), 4.18 (m, 1H), 3.84 (m, 1H), 3.18 (q, J = 12.8 Hz, 3H), 2.21–2.20 (m, 6H), 1.95–1.92 (m, 6H), 1.83–1.65 (m, 11H), 1.50 (q, J = 12.8 Hz, 6H), 1.40 (q, J = 7.6 Hz, 2H), 1.35–1.23 (m, 3H), 0.97 (t, J = 7.6 Hz, 3H),. **^{13}C NMR** (100.6 MHz, $CDCl_3$) δ 161.1, 143.9 (d, $J_{C\text{–}P}$ = 8.6 Hz), 127.1, 124.9, 124.6, 117.4, 115.7, 70.9 (d, $J_{C\text{–}P}$ = 60.2 Hz), 41.6, 31.7 (d, $J_{C\text{–}P}$ = 35.4 Hz), 28.8, 27.8 (d, $J_{C\text{–}P}$ = 4.3 Hz), 27.7 (d, $J_{C\text{–}P}$ = 4.2 Hz), 26.7 (d, $J_{C\text{–}P}$= 12.4 Hz, ×2C), 25.5, 20.0, 13.7. **^{31}P NMR** (162 MHz, $CDCl_3$) δ 36.6. **HRMS–ESI** (*m/z*): $[M–Cl]^+$ calcd for $C_{30}H_{47}NO_2P$; 484.3339, found 484.3340.

(4-Benzyl-3-oxo-3,4-dihydro-2*H*-benzo[*b*][1,4]oxazin-2-yl)tricyclohexylphosphonium chloride (2s)

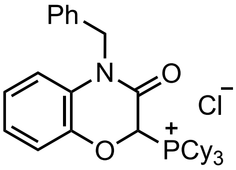

The reaction was carried out according to procedure A. The product **2s** was purified by recrystallization by sonication of a THF, hexane solution. (Fig. 2; 410.1 mg, 0.74 mmol, 74% isolated yield.). White solid. **M.p.** 210–220 °C (decomp.). **IR** (neat) 720, 754, 927, 1128, 1315, 1442, 1498, 1667, 2855, 2935 cm $^{-1}$. **^{1}H NMR** (400 MHz, $CDCl_3$) δ 7.34–7.27 (m, 5H), 7.08–7.06 (m, 4H), 6.96 (d, J = 6.8 Hz, 1H), 5.37 (d, J = 16.0 Hz, 1H), 5.09 (d, J = 16.0 Hz, 1H), 3.20 (q, J = 12.0 Hz, 3H), 2.24–2.21 (m, 6H), 1.95–1.92 (m, 6H), 1.82–1.68 (m, 9H), 1.52–1.44 (m, 6H), 1.33–1.23 (m, 3H). **^{13}C NMR** (100.6 MHz, $CDCl_3$) δ 161.7, 143.8 (d, $J_{C\text{–}P}$ = 8.8 Hz), 134.9, 129.0, 127.9, 127.5, 127.0, 125.0, 124.6, 117.3, 116.4, 71.2 (d, $J_{C\text{–}P}$ = 60.8 Hz), 45.3, 31.7 (d, $J_{C\text{–}P}$ = 35.4 Hz), 27.8 (d, $J_{C\text{–}P}$ = 4.4

Hz), 27.7 (d, J_{C-P} = 4.2 Hz), 26.7 (d, J_{C-P}= 12.4 Hz), 26.7 (d, J_{C-P}= 12.3 Hz), 25.5. **^{31}P NMR** (162 MHz, $CDCl_3$) δ 36.7. **HRMS–ESI** (*m/z*): $[M–Cl]^+$ calcd for $C_{33}H_{45}NO_2P$; 518.3182, found 518.3182.

Tricyclohexyl(4-isopropyl-3-oxo-3,4-dihydro-2*H*-benzo[*b*][1,4]oxazin-2-yl)phosphonium chloride (2t)

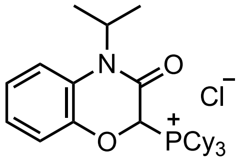

The reaction was carried out according to procedure A. The product **2t** was purified by recrystallization by sonication of a THF, Et_2O solution. (Fig. 2; 212.6 mg, 0.42 mmol, 42% isolated yield.). White solid. **M.p.** 216–225 °C (decomp.). **IR** (neat) 1269, 1305, 1353, 1365, 1379, 1448, 1500, 1668, 2850, 2936 cm $^{-1}$. **^{1}H NMR** (600 MHz, $CDCl_3$) δ 7.23 (d, *J* = 7.8 Hz, 1H), 7.19 (t, *J* = 7.2 Hz, 1H), 7.12 (t, *J* = 7.2 Hz, 1H), 7.08 (d, *J* = 7.8 Hz, 1H), 6.06 (d, *J* = 6.6 Hz, 1H), 4.58 (quin, *J* = 6.6 Hz, 1H), 3.18 (q, *J* = 12.6 Hz, 3H), 2.21–2.17 (m, 6H), 1.95–1.94 (m, 6H), 1.82 (d, *J* = 12.6 Hz, 3H), 1.75–1.68 (m, 6H), 1.63 (d, *J* = 6.6 Hz, 3H), 1.60 (d, *J* = 6.6 Hz, 3H), 1.52 (q, *J* = 12.8 Hz, 6H), 1.34–1.28 (m, 3H). **^{13}C NMR** (150.9 MHz, $CDCl_3$) δ 162.5, 144.4 (d, J_{C-P} = 8.3 Hz), 128.5, 125.1, 124.9, 117.4, 116.5, 71.5 (d, J_{C-P} = 59.1 Hz), 50.2, 31.8 (d, J_{C-P} = 35.4 Hz), 27.7 (d, J_{C-P} = 3.9 Hz), 27.6 (d, J_{C-P} = 3.9 Hz), 26.5 (d, J_{C-P}= 12.3 Hz), 25.4, 19.9, 19.1. **^{31}P NMR** (162 MHz, $CDCl_3$) δ 38.0. **HRMS–ESI** (*m/z*): $[M–Cl]^+$ calcd for $C_{29}H_{45}NO_2P$; 470.3182, found 470.3183.

Tricyclohexyl(3-oxo-2,3,6,7-tetrahydro-5*H*-[1,4]oxazino[2,3,4-*ij*]quinolin-2-yl)phosphonium chloride (2u)

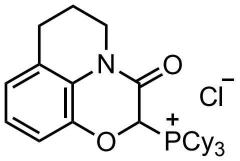

The reaction was carried out according to procedure B. The product **2u** was purified by recrystallization by sonication of a THF, hexane solution. (Fig. 2; 62.5 mg, 0.12 mmol, 62% isolated yield.). White solid. **M.p.** 225–234 °C (decomp.). **IR** (neat) 849, 917, 1063, 1261, 1328, 1389, 1519, 1661, 2851, 2933 cm $^{-1}$. **^{1}H NMR** (400 MHz, $CDCl_3$) δ 6.98 (m, 1H), 6.90 (m, 1H), 6.85 (m, 1H), 6.69 (d, *J* = 6.8 Hz, 1H), 4.18 (m, 1H), 3.65 (m, 1H), 3.13 (q, *J* = 12.8 Hz, 3H), 2.95–2.80 (m, 2H), 2.22–2.19 (m, 6H), 2.15–2.00 (m, 2H) 1.95–1.93 (m, 6H), 1.81 (d, *J* = 12.4 Hz, 3H), 1.76–1.63 (m, 6H), 1.48 (q, *J* = 12.8 Hz, 6H), 1.34–1.25 (m, 3H). **^{13}C NMR** (100.6 MHz, $CDCl_3$) δ 159.8, 142.6 (d, J_{C-P} = 7.5 Hz), 126.7, 124.5, 124.2, 123.7, 114.6, 70.2 (d, J_{C-P} = 58.7 Hz), 40.8, 31.6 (d, J_{C-P} = 35.2 Hz), 27.6 (d, J_{C-P} = 1.8 Hz), 27.6 (d, J_{C-P} = 1.4 Hz), 26.7 (d, J_{C-P}= 12.4 Hz), 26.7 (d, J_{C-P} = 12.3 Hz), 26.1, 25.5, 20.5. **^{31}P NMR** (162 MHz, $CDCl_3$) δ 36.4. **HRMS–ESI** (*m/z*): $[M–Cl]^+$ calcd for $C_{29}H_{43}NO_2P$; 468.3026, found 468.3017.

Tricyclohexyl(4-(2-methoxyethyl)-3-oxo-3,4-dihydro-2*H*-benzo[*b*][1,4]oxazin-2-yl)phosphonium chloride (2v)

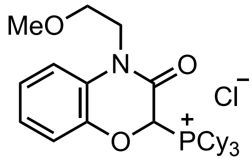

The reaction was carried out according to procedure A. The product **2v** was purified by recrystallization by sonication of a THF, hexane solution. (Fig. 2; 370.7 mg, 0.71 mmol, 71% isolated yield.). White solid. **M.p.** 218–224 °C (decomp.). **IR** (neat) 760, 851, 1063, 1119, 1242, 1404, 1499, 1664, 2848, 2930 cm $^{-1}$. **^{1}H NMR** (400 MHz, $CDCl_3$) δ 7.25 (dd, J = 8.0, 1.6 Hz, 1H), 7.19–7.04 (m, 3H), 6.55 (d, J = 6.8 Hz, 1H), 4.49 (m, 1H), 4.02 (dt, J = 14.8, 4.0 Hz, 1H), 3.71–3.61 (m, 2H), 3.32 (s, 3H), 3.15 (q, J = 12.4 Hz, 3H), 2.22–2.14 (m, 6H), 1.95–1.93 (m, 6H), 1.83–1.65 (m, 9H), 1.49 (q, J = 12.4 Hz, 6H), 1.35–1.26 (m, 3H). **^{13}C NMR** (100.6 MHz, $CDCl_3$) δ 161.3, 143.7 (d, $J_{C\text{–}P}$ = 8.5 Hz), 127.2, 125.0, 124.6, 117.2, 116.4, 70.7 (d, $J_{C\text{–}P}$ = 60.3 Hz), 68.6, 58.9, 41.7, 31.6 (d, $J_{C\text{–}P}$ = 35.5 Hz), 27.6 (d, $J_{C\text{–}P}$ = 4.4 Hz), 27.5 (d, $J_{C\text{–}P}$ = 4.3 Hz), 26.6 (d, $J_{C\text{–}P}$= 12.3 Hz), 26.6 (d, $J_{C\text{–}P}$= 12.4 Hz), 25.4. **^{31}P NMR** (162 MHz, $CDCl_3$) δ 36.4. **HRMS–ESI** (*m/z*): [M–Cl]$^{+}$ calcd for $C_{29}H_{45}NO_3P$; 486.3132, found 486.3147.

Tricyclohexyl(4-(3-methoxy-3-oxopropyl)-3-oxo-3,4-dihydro-2*H*-benzo[*b*][1,4]oxazin-2-yl)phosphonium chloride (2w)

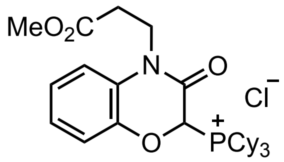

The reaction was carried out according to procedure A. The product **2w** was purified by recrystallization by sonication of a THF, hexane solution. (Fig. 2; 401.6 mg, 0.73 mmol, 73% isolated yield.). White solid. **M.p.** 190–196 °C (decomp.). **IR** (neat) 742, 1209, 1314, 1373, 1401, 1502, 1664, 1731, 2855, 2940 cm $^{-1}$. **^{1}H NMR** (400 MHz, $CDCl_3$) δ 7.19–7.05 (m, 4H), 6.91 (d, J = 6.8 Hz, 1H), 4.39 (m, 1H), 4.24 (m, 1H), 3.66(s, 3H), 3.15 (q, J = 12.8 Hz, 3H), 2.85–2.70 (m, 2H), 2.22–2.21 (m, 6H), 1.97–1.94 (m, 6H), 1.83–1.69 (m, 9H), 1.49 (q, J = 12.8 Hz, 6H), 1.36–1.26 (m, 3H). **^{13}C NMR** (100.6 MHz, $CDCl_3$) δ 171.1, 161.5, 143.9 (d, $J_{C\text{–}P}$ = 8.8 Hz), 127.0, 125.0, 124.7, 117.4, 115.4, 71.0 (d, $J_{C\text{–}P}$ = 60.7 Hz), 51.9, 37.9, 31.7 (d, $J_{C\text{–}P}$ = 35.5 Hz), 31.3, 27.7 (d, $J_{C\text{–}P}$ = 3.7 Hz), 27.6 (d, $J_{C\text{–}P}$ = 3.4 Hz), 26.7 (d, $J_{C\text{–}P}$= 12.3 Hz), 26.7 (d, $J_{C\text{–}P}$= 12.3 Hz), 25.4. **^{31}P NMR** (162 MHz, $CDCl_3$) δ 36.4. **HRMS–ESI** (*m/z*): [M–Cl]$^{+}$ calcd for $C_{30}H_{45}NO_4P$; 514.3081, found 514.3088.

(4-Allyl-3-oxo-3,4-dihydro-2*H*-benzo[*b*][1,4]oxazin-2-yl)tricyclohexylphosphonium chloride (2x)

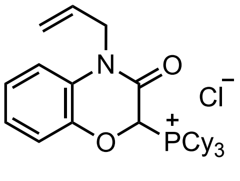

The reaction was carried out according to procedure A. The product **2x** was purified by recrystallization by sonication of a THF, hexane solution. (Fig. 2; 186.5 mg, 0.37 mmol, 37% isolated

yield.). White solid. **M.p.** 238–242 °C (decomp.). **IR** (neat) 756, 1064, 1240, 1325, 1408, 1434, 1469, 1670, 2851, 2930 cm $^{-1}$. **^{1}H NMR** (400 MHz, $CDCl_3$) δ 7.15–7.04 (m, 4H), 6.99 (d, J = 6.8 Hz, 1H), 5.88 (m, 1H), 5.35–5.28 (m, 2H), 4.68–4.58 (m, 2H), 3.18 (q, J = 12.8 Hz, 3H), 2.23–2.17 (m, 6H), 1.95–1.93 (m, 6H), 1.83–1.66 (m, 9H), 1.49 (q, J = 12.8 Hz, 6H), 1.34–1.24 (m, 3H). **^{13}C NMR** (100.6 MHz, $CDCl_3$) δ 161.2, 143.6 (d, $J_{C–P}$ = 8.4 Hz), 130.3, 127.4, 125.0, 124.5, 118.5, 117.2, 116.1, 70.9 (d, $J_{C–P}$ = 59.9 Hz), 44.3, 31.7 (d, $J_{C–P}$ = 35.4 Hz), 27.7 (d, $J_{C–P}$ = 4.5 Hz), 27.7 (d, $J_{C–P}$ = 4.5 Hz), 26.7 (d, $J_{C–P}$= 12.4 Hz), 26.7 (d, $J_{C–P}$= 12.4 Hz), 25.5. **^{31}P NMR** (162 MHz, $CDCl_3$) δ 36.8. **HRMS–ESI** (*m/z*): $[M–Cl]^+$ calcd for $C_{29}H_{43}NO_2P$; 468.3026, found 468.3006.

(4-(2-Cyanoethyl)-3-oxo-3,4-dihydro-2*H*-benzo[*b*][1,4]oxazin-2-yl)tricyclohexylphosphonium chloride (2y)

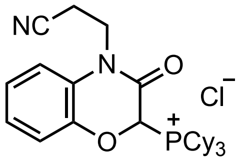

The reaction was carried out according to procedure A. The product **2y** was purified by recrystallization by sonication of a THF, hexane solution. (Fig. 2; 496.4 mg, 0.96 mmol, 96% isolated yield.). White solid. **M.p.** 247–253 °C (decomp.). **IR** (neat) 925, 1243, 1300, 1366, 1396, 1501, 1674, 2179, 2855, 2936 cm $^{-1}$. **^{1}H NMR** (400 MHz, $CDCl_3$) δ 7.44 (d, J = 7.2 Hz, 1H), 7.28 (m, 1H), 7.16 (t, J = 7.2 Hz, 1H), 7.10 (t, J = 7.2 Hz, 1H), 7.04 (m, 1H), 4.47 (m, 1H), 4.28 (m, 1H), 3.05–2.86 (m, 5H), 2.22–2.17 (m, 6H), 1.98–1.96 (m, 6H), 1.84–1.73 (m, 9H), 1.48–1.22 (m, 9H). **^{13}C NMR** (100.6 MHz, $CDCl_3$) δ 162.1, 144.2 (d, $J_{C–P}$ = 9.4 Hz), 126.7, 125.1, 125.0, 117.4, 117.4, 115.8, 71.1 (d, $J_{C–P}$ = 62.4 Hz), 37.4, 31.8 (d, $J_{C–P}$ = 35.7 Hz), 27.7 (d, $J_{C–P}$ = 4.9 Hz), 27.7 (d, $J_{C–P}$ = 4.9 Hz), 26.8 (d, $J_{C–P}$= 12.3 Hz), 26.8 (d, $J_{C–P}$= 12.3 Hz), 25.4, 15.9. **^{31}P NMR** (162 MHz, $CDCl_3$) δ 35.7. **HRMS–ESI** (*m/z*): $[M–Cl]^+$ calcd for $C_{29}H_{42}NO_2P$; 481.2978, found 481.2974.

Tricyclohexyl(3-oxo-4-phenyl-3,4-dihydro-2*H*-benzo[*b*][1,4]oxazin-2-yl)phosphonium chloride (2z)

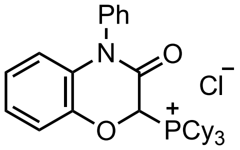

The reaction was carried out according to procedure A. The product **2z** was purified by recrystallization by sonication of a THF, hexane solution. (Fig. 2; 486.1 mg, 0.90 mmol, 90% isolated yield.). White solid. **M.p.** 217–222 °C (decomp.). **IR** (neat) 771, 1060, 1299, 1319, 1372, 1500, 1686, 2763, 2840, 2934 cm $^{-1}$. **^{1}H NMR** (400 MHz, $CDCl_3$) δ 7.60–7.56 (m, 2H), 7.52 (m, 1H), 7.41 (d, J = 6.8 Hz, 3H), 7.10–7.04 (m, 2H), 6.94 (m, 1H), 6.46 (d, J = 8.0 Hz, 1H), 3.13 (q, J = 12.8 Hz, 3H), 2.29–2.23 (m, 6H), 1.97–1.94 (m, 6H), 1.83–1.71 (m, 9H), 1.50–1.42 (m, 6H), 1.36–1.26 (m, 3H). **^{13}C NMR** (100.6 MHz, $CDCl_3$) δ 161.5, 143.0 (d, $J_{C–P}$ = 8.7 Hz), 134.8, 130.3, 129.5, 129.4, 128.6 (broad), 124.9, 124.2, 117.4, 116.9, 71.4 (d, $J_{C–P}$ = 60.3 Hz), 31.7 (d, $J_{C–P}$ = 35.3 Hz), 27.7 (d, $J_{C–P}$ =

1.9 Hz), 27.7 (d, J_{C-P} = 1.9 Hz), 26.7 (d, J_{C-P}= 12.4 Hz), 26.7 (d, J_{C-P}= 12.4 Hz), 25.5. ^{31}**P NMR** (162 MHz, $CDCl_3$) δ36.9. **HRMS–ESI** (*m/z*): $[M–Cl]^+$ calcd for $C_{32}H_{43}NO_2P$; 504.3026, found 504.3041.

Tributyl(4-methyl-3-oxo-3,4-dihydro-2*H*-benzo[*b*][1,4]oxazin-2-yl)phosphonium chloride (2a')

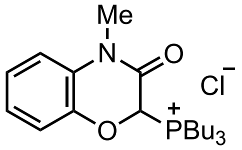

The reaction was carried out according to procedure A. (PBu_3 was used instead of PCy_3.). The product **2a'** was purified by recrystallization by sonication of a THF, hexane solution. (Fig. 2; 308.0 mg, 0.77 mmol, 77% isolated yield.). White solid. **M.p.** 207–212 °C (decomp.). **IR** (neat) 1226, 1316, 1380, 1464, 1500, 1672, 2755, 2871, 2931, 2958 cm $^{-1}$. ^{1}**H NMR** (400 MHz, $CDCl_3$) δ7.15 (m, 1H), 7.11–7.04 (m, 3H), 6.97 (d, *J* = 9.2 Hz, 1H), 3.40 (s, 3H), 2.90–2.71 (m, 6H), 1.72–1.60 (m, 6H), 1.59–1.48 (m, 6H), 0.97 (t, *J* = 7.2 Hz, 9H). ^{13}**C NMR** (100.6 MHz, $CDCl_3$) δ161.0, 143.6 (d, J_{C-P} = 8.7 Hz), 128.3, 124.8, 124.4, 117.1, 115.4, 71.5 (d, J_{C-P} = 68.1 Hz), 28.7, 24.0 (d, J_{C-P} = 4.8 Hz), 23.9 (d, J_{C-P} = 16.3 Hz), 18.5 (d, J_{C-P} = 44.2 Hz), 13.3. ^{31}**P NMR** (162 MHz, $CDCl_3$) δ39.5. **HRMS–ESI** (*m/z*): $[M–Cl]^+$ calcd for $C_{21}H_{35}NO_2P$; 364.2400, found 364.2400.

4. X-ray Diffraction Analysis for 2a’

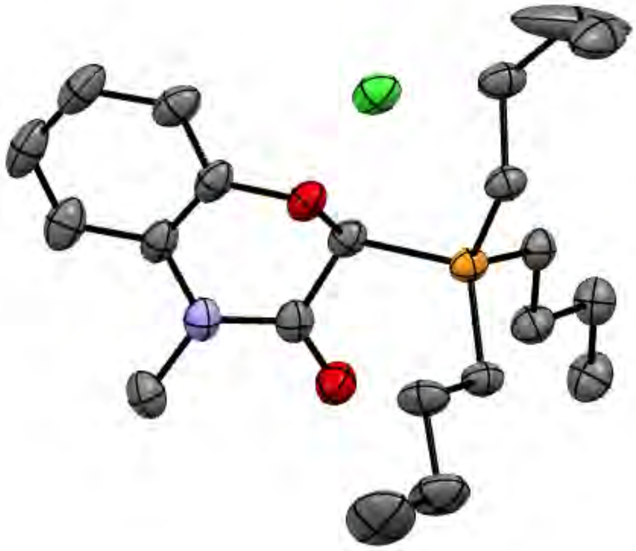

Figure S2. ORTEP diagram of **2a**’ (CCDC 2266573). Ellipsoids are drawn at 50% probability. A single crystal used for the analysis was obtained by recrystallization from n-hexane and tetrahydrofuran. Hydrogen atoms are omitted for clarity.

Bond precision: C-C = 0.0062 A Wavelength=0.71075

Cell:	a=9.5728(6)	b=12.9468(9)	c=18.7278(10)
	alpha=90	beta=90	gamma=90
Temperature:	296 K		

	Calculated	Reported
Volume	2321.1(2)	2321.1(3)
Space group	P 21 21 21	P 21 21 21
Hall group	P 2ac 2ab	P 2ac 2ab
Moiety formula	C21 H35 N O2 P, Cl	C21 H35 Cl N O2 P
Sum formula	C21 H35 Cl N O2 P	C21 H35 Cl N O2 P
Mr	399.92	399.94
Dx,g cm-3	1.144	1.144
Z	4	4
Mu (mm-1)	0.247	0.247
F000	864.0	864.0
F000’	865.29	
h,k,lmax	12,16,24	12,16,24
Nref	5325[3011]	5299
Tmin,Tmax	0.821,0.841	0.578,0.841
Tmin’	0.821	

Correction method= # Reported T Limits: Tmin=0.578 Tmax=0.841
AbsCorr = MULTI-SCAN

Data completeness= 1.76/1.00 Theta(max)= 27.484

R(reflections)= 0.0502(4695) wR2(reflections)= 0.1350(5299)

S = 1.046 Npar= 235

5. Deprotection of LOA Group

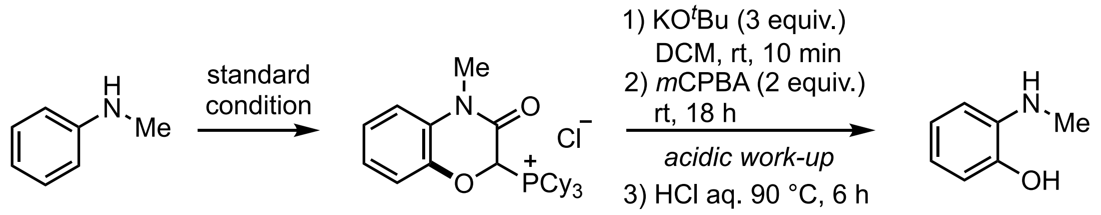

The reaction with 1a is representative. 2a was prepared from **1a** according to Procedure A and utilized for deprotection without purification. Then, **2a** (~ 1 mmol) was dissolved with DCM (10 ml). To the reaction mixture was added KO^{t}Bu (336.6 mg, 3.0 mmol, 3 equiv.) slowly in a glove box. After stirring for 1 hour, mCPBA contains 30% water (345.1 mg, 1.4 mmol, 1.4 equiv.) was slowly added at room temperature. After stirring for 18 hours, the solvent was removed by a rotary evaporator. Then concentrated hydrochloric acid (12 M, 2 mL) was added at room temperature and stirred at 90 °C for 6 h. After quenching with 6 N NaOH (12 mL), extracted with EtOAc three times and the combined organic layer was dried over Na_2SO_4 and filtered. After volatiles were removed under reduced pressure, purification by flash column chromatography on silica gel (95:5–75:25, hexane/EtOAc) gave **3a** in 66% yield based on **1a**.

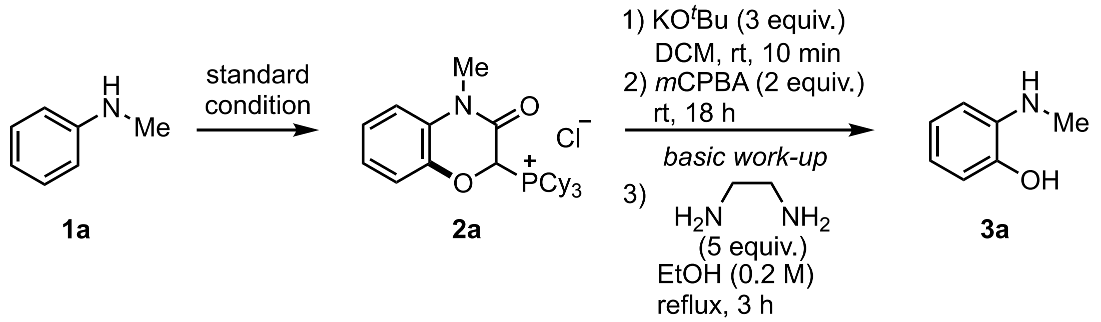

The reaction with 1a is representative. 2a was prepared from **1a** according to Procedure A and utilized for deprotection without purification. Then, **2a** (~ 1 mmol) was dissolved with DCM (10 ml). To the reaction mixture was added KO^{t}Bu (336.6 mg, 3.0 mmol, 3 equiv.) slowly in a glove box. After stirring for 1 hour, mCPBA contains 30% water (345.1 mg, 1.4 mmol, 1.4 equiv.) was slowly added at room temperature. After stirring for 18 hours, the solvent was removed by a rotary evaporator. Then, the residue was dissolved with EtOH (0.2 M, 5 ml). To the reaction mixture was added ethylenediamine (300.5 mg, 5.0 mmol, 5 equiv.) slowly at room temperature. After 3 h stirring at 80 °C, the solvent was removed by evacuation. And the residue was purified by silica gel chromatography on silica gel (95:5–75:25, hexane/EtOAc) gave **3a** in 52% yield based on **1a**.

6. Characterization of 2-Aminophenols

2-(Methylamino)phenol (3a)

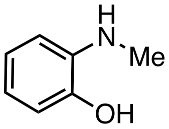

The product **3a** was purified by flash chromatography on silica gel (95:5–75:25, hexane/EtOAc) (Fig. 3A; 81.3 mg, 0.66 mmol, 66% isolated yield. Basic work-up; 64.1 mg, 0.52 mmol, 52% isolated yield.). Dark green solid. The spectrum data of product was consistent with the literature.[4]

5-Chloro-2-(methylamino)phenol (3f)

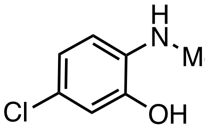

The product **3f** was purified by flash chromatography on silica gel (95:5–75:25, hexane/EtOAc) (Fig. 3A; 105.6 mg, 0.67 mmol, 67% isolated yield.). Reddish purple solid. **M.p.** 93–103 °C. **IR** (neat) 852, 1129, 1152, 1260, 1450, 1472, 1589, 2993, 3349 cm $^{-1}$. **^{1}H NMR** (400 MHz, CD_3CN) δ 6.78 (dd, J = 8.4, 2.4 Hz, 1H), 6.71 (d, J = 2.4 Hz, 1H), 6.49 (d, J = 8.4 Hz, 1H), 2.78 (s, 3H). **^{13}C NMR** (100.6 MHz, CD_3CN) δ 145.2, 138.9, 121.1, 120.3, 114.3, 110.9, 30.4. **HRMS–DART** (*m/z*): $[M+H]^+$ calcd for C_7H_9ClNO; 158.0367, found 158.0364.

2-(Methylamino)-5-(trifluoromethyl)phenol (3i)

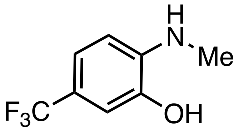

The product **3i** was purified by flash chromatography on silica gel (95:4:1–75:24:1, hexane/EtOAc/NEt_3) (Fig. 3A; 118.5 mg, 0.62 mmol, 62% isolated yield.). Yellowish brown solid. **M.p.** 77–80 °C. **IR** (neat) 912, 1106, 1153, 1264, 1334, 1454, 1484, 1613, 3076, 3379 cm $^{-1}$. **^{1}H NMR** (400 MHz, $CDCl_3$) δ 7.26 (d, J = 8.0 Hz, 1H), 6.89 (s, 1H), 6.60 (d, J = 8.0 Hz, 1H), 4.64 (broad, 2H), 2.91 (s, 3H). **^{13}C NMR** (100.6 MHz, $CDCl_3$) δ 142.0, 141.2, 124.7 (q, $J_{C–F}$ = 270.0 Hz), 120.7, 118.0 (q, $J_{C–F}$ = 32.4 Hz), 110.6, 109.2, 30.1. **^{19}F NMR** (376.5 MHz, $CDCl_3$) δ –60.8. **HRMS–DART** (*m/z*): $[M+H]^+$ calcd for $C_8H_9F_3NO$; 192.0631, found 192.0632.

2-(Benzylamino)phenol (3s)

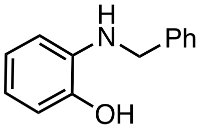

The product **3s** was purified by flash chromatography on silica gel (95:5–75:25, hexane/EtOAc) (Fig. 3A; 55.6 mg, 0.28 mmol, 28% isolated yield). Dark green solid. The spectrum data of **3a** was consistent with the literature.[4]

2-((2-Methoxyethyl)amino)phenol (3v)

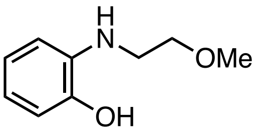

The product **3v** was purified by flash chromatography on silica gel (95:4:1–75:24:1, hexane/EtOAc/NEt_3) (Fig. 3A; 75.2 mg, 0.45 mmol, 45% isolated yield.). Brown solid. **M.p.** 64–74 °C. **IR** (neat) 1099, 1118, 1127, 1244, 1273, 1389, 1460, 1510, 2910, 3341 cm $^{-1}$. **^{1}H NMR** (400 MHz, $CDCl_3$) δ 6.85 (m, 1H), 6.72–6.63 (m, 3H), 5.06 (broad, 2H), 3.65 (t, J = 5.2 Hz, 2H), 3.43 (s, 3H), 3.30 (t, J = 5.2 Hz, 2H). **^{13}C NMR** (100.6 MHz, $CDCl_3$) δ 144.7, 136.7, 121.2, 118.5, 114.5, 113.3, 71.3, 58.7, 44.2. **HRMS–DART** (*m/z*): $[M+H]^+$ calcd for $C_9H_{14}NO_2$; 168.1019, found 168.1019.

2-(Phenylamino)phenol (3z)

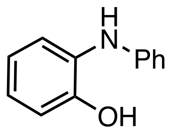

The product **3z** was purified by flash chromatography on silica gel (95:5–75:25, hexane/EtOAc) (Fig. 3A; 125.9 mg, 0.68 mmol, 68% isolated yield). Thin yellow solid. The spectrum data of product was consistent with the literature.[5]

7. Procedures for Dephosphorylative Functionalization

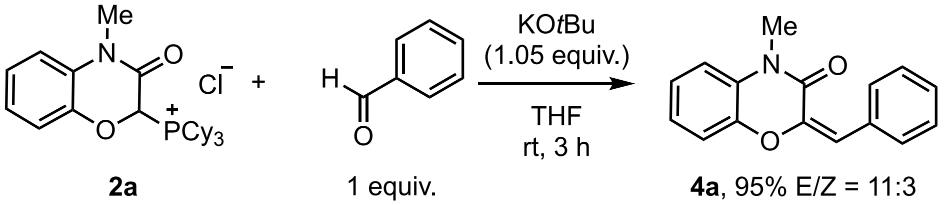

Wittig reaction. In a glovebox, to an oven-dried vial with a stirring bar was added 1,4-benzoxazinone phosphonium salt **2a** (95.6 mg, 0.2 mmol, 1.0 equiv.) and THF (1 mL). To the reaction mixture was added KOtBu (23.6 mg, 0.21 mmol, 1.05 equiv.) slowly. After stirring for 30 minutes, benzaldehyde (20.3 µL, 0.2 mmol, 1 equiv.) was slowly added at room temperature. The reaction was stirred at room temperature for 3 hours and quenched with water. The resultant reaction solution was extracted with ethyl acetate three times and the combined organic layer was dried over Na_2SO_4 and filtered. After volatiles were removed under reduced pressure, purification by flash column chromatography on silica gel gave the Wittig reaction product **4a** (47.5 mg, 0.19 mmol, 95% isolated yield).

(*E*)-2-Benzylidene-4-methyl-2*H*-benzo[*b*][1,4]oxazin-3(4*H*)-one (4a, major)
(*Z*)-2-Benzylidene-4-methyl-2*H*-benzo[*b*][1,4]oxazin-3(4*H*)-one (4a, minor)

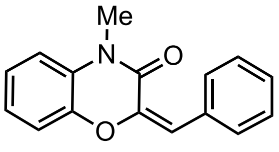

The product **4a** was purified by flash chromatography on silica gel (95:5–85:15, hexane/EtOAc) (Fig. 3B; 47.5 mg, 0.19 mmol, 95% isolated yield). Thin yellow solid. Signals for two regioisomer (11:3) were given: The spectrum data of product was consistent with the literature.[6]

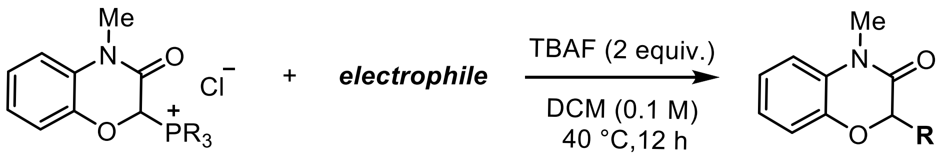

Dephosphorylative protonation. In a glovebox, to an oven-dried vial with a stirring bar was added 1,4-benzoxazinone phosphonium salt **2a** (47.8 mg, 0.1 mmol, 1.0 equiv.) and DCM (1 mL). Then, TBAF (1.0 mol/L THF, 200 µL, 2.0 equiv.) and H_2O (9 µL, 0.5 mmol) was sequentially and slowly added at the room temperature. The reaction was stirred at 40 °C for 12 hours and quenched with water. The resultant reaction solution was extracted with ethyl acetate three times and the combined organic layer was dried over Na_2SO_4 and filtered. After volatiles were removed under reduced pressure, purification by flash column chromatography on silica gel gave the dephosphorylated product **5a** (16.2 mg, 0.099 mmol, 99% isolated yield.).

Dephosphorylative alkylation. In a glovebox, to an oven-dried vial with a stirring bar was added 1,4-benzoxazinone phosphonium salt **2a** (47.8 mg, 0.1 mmol, 1.0 equiv.) and DCM (1 mL). Then, slowly add 3 Å molecular sieve (50 mg), TBAF (1.0 mol/L THF, 200 µL, 2.0 equiv.) and methyl acrylate (for alkylation, 44.8 µL, 0.5 mmol) or benzyl bromide (for benzylation, 59.4 µL, 0.5 mmol) was sequentially and slowly added at the room temperature. The reaction was stirred at 40 °C for 12 hours and quenched with water. The resultant reaction solution was extracted with ethyl acetate three times and the combined organic layer was dried over Na_2SO_4 and filtered. After volatiles were removed under reduced pressure, purification by flash column chromatography on silica gel gave the dephosphorylated product.

4-Methyl-2*H*-benzo[*b*][1,4]oxazin-3(4*H*)-one (5a)

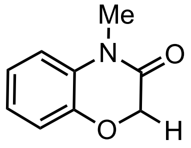

The product **5a** was purified by flash chromatography on silica gel (100:0–90:10, hexane/EtOAc) (Fig. 3B; 16.2 mg, 0.099 mmol, 99% isolated yield.). Colorless crystal. **M.p.** 232–236 °C. **IR** (neat) 750, 1054, 1226, 1280, 1386, 1478, 1503, 1678 cm $^{-1}$. ^{1}**H NMR** (400 MHz, $CDCl_3$) δ 7.26–6.97 (m, 4H), 4.62 (s, 2H), 3.37 (s, 3H). ^{13}**C NMR** (100.6 MHz, $CDCl_3$) δ 164.6, 145.1, 129.5, 123.8, 122.7, 116.8, 114.7, 67.6, 28.0. **HRMS–DART** (*m/z*): $[M+H]^+$ calcd for $C_9H_{10}NO_2$; 164.0706, found 164.0707.

Methyl 3-(4-Methyl-3-oxo-3,4-dihydro-2*H*-benzo[*b*][1,4]oxazin-2-yl)propanoate (5b)

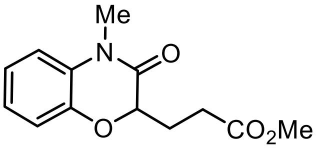

The product **5b** was purified by flash chromatography on silica gel (95:5–85:15, hexane/EtOAc) (Fig. 3B; 22.9 mg, 0.092 mmol, 92% isolated yield.). Pale yellow oil. **IR** (neat) 747, 1125, 1196, 1236, 1386, 1419, 1477, 1502, 1675, 1734 cm $^{-1}$. **^{1}H NMR** (400 MHz, $CDCl_3$) δ 7.07–6.95 (m, 4H), 4.58 (dd, *J* = 8.4, 4.4 Hz, 1H), 3.68 (s, 3H), 3.36 (s, 3H), 2.60–2.56 (m, 2H), 2.32 (m, 1H), 2.18 (m,1H). **^{13}C NMR** (100.6 MHz, $CDCl_3$) δ 173.1, 165.7, 143.9, 129.6, 123.9, 122.8, 117.1, 114.6, 75.9, 51.7, 29.4, 28.4, 25.7. **HRMS–DART** (*m/z*): $[M+H]^+$ calcd for $C_{13}H_{16}NO_2$; 250.1074, found 250.1076.

2-Benzyl-4-methyl-2*H*-benzo[*b*][1,4]oxazin-3(4*H*)-one (5c)

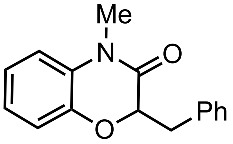

The product **5c** was purified by flash chromatography on silica gel (90:10–70:30, hexane/EtOAc) (Fig. 3B; 24.1 mg, 0.095 mmol, 95% isolated yield.). Colorless oil. **IR** (neat) 697, 744, 1079, 1264, 1277, 1383, 1453, 1475, 1500, 1674 cm $^{-1}$. **^{1}H NMR** (400 MHz, $CDCl_3$) δ 7.31–7.21 (m, 5H), 7.04–6.91 (m, 4H), 4.79 (dd, *J* = 9.6, 3.2 Hz, 1H), 3.36 (s, 3H), 3.23 (m, 1H), 3.06 (m, 1H). **^{13}C NMR** (100.6 MHz, $CDCl_3$) δ 165.8, 143.6, 136.6, 129.5, 129.5, 128.3, 126.7, 123.9, 122.5, 117.4, 114.5, 78.0, 36.7, 28.3. **HRMS–DART** (*m/z*): $[M+H]^+$ calcd for $C_{16}H_{16}NO_2$; 254.1176, found 254.1170.

8. UV-Vis Adsorption Spectra Experiments

UV-Vis absorption spectra of α-ketoacylchloride **1a-1** and α-ketoacylphosphonium **1a-2** were measured with 0.8 mM MeCN solution.

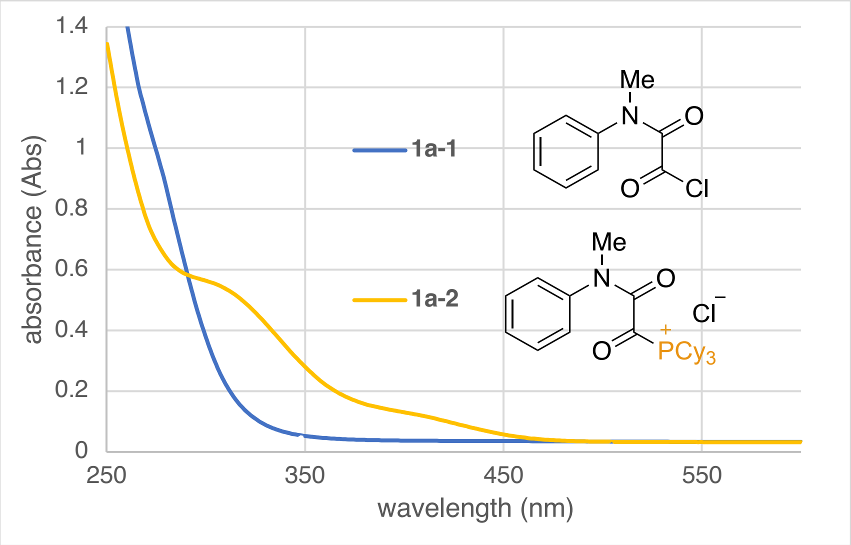

Figure S3. UV-Vis absorption spectra of α-ketoacylchloride and α-ketoacylphosphonium

9. NMR Spectra of Reaction Intermediates

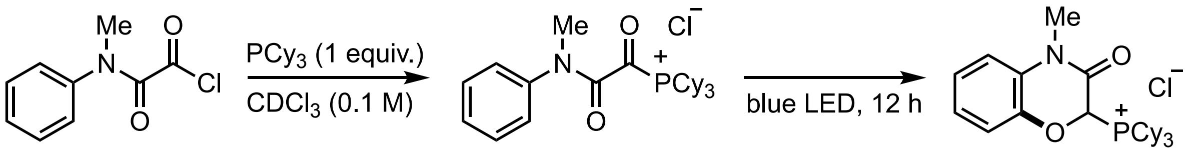

1a-1 **1a-2** **2a**

$^1H/^{31}P$-NMR $^1H/^{31}P$-NMR

Procedure. An oven-dried vial (4 mL) with a stirring bar was placed under N_2 atmosphere by three cycles of evacuating and N_2 backfilling and charged with **1a-1** (19.8 mg, 0.1 mmol, 1.0 equiv.) and 1,1,2,2-tetrachloroethane (11.2 mg, 0.067 mmol) as an internal standard in dry $CDCl_3$ (1.0 mL). Then, tricyclohexylphosphine (28.0 mg, 0.1 mmol, 1.0 equiv.) was added to the reaction mixture in the glove box. After stirring for 30 min, the aliquot was utilized for $^1H/^{31}P$-NMR analysis of **1a-2**. The vial was brought outside the glove box and irradiated with 440 nm blue LED with a cooling fan to keep the temperature around 40 °C for 12 h (Figure S1). Then the aliquot was utilized for $^1H/^{31}P$-NMR analysis of **2a**.

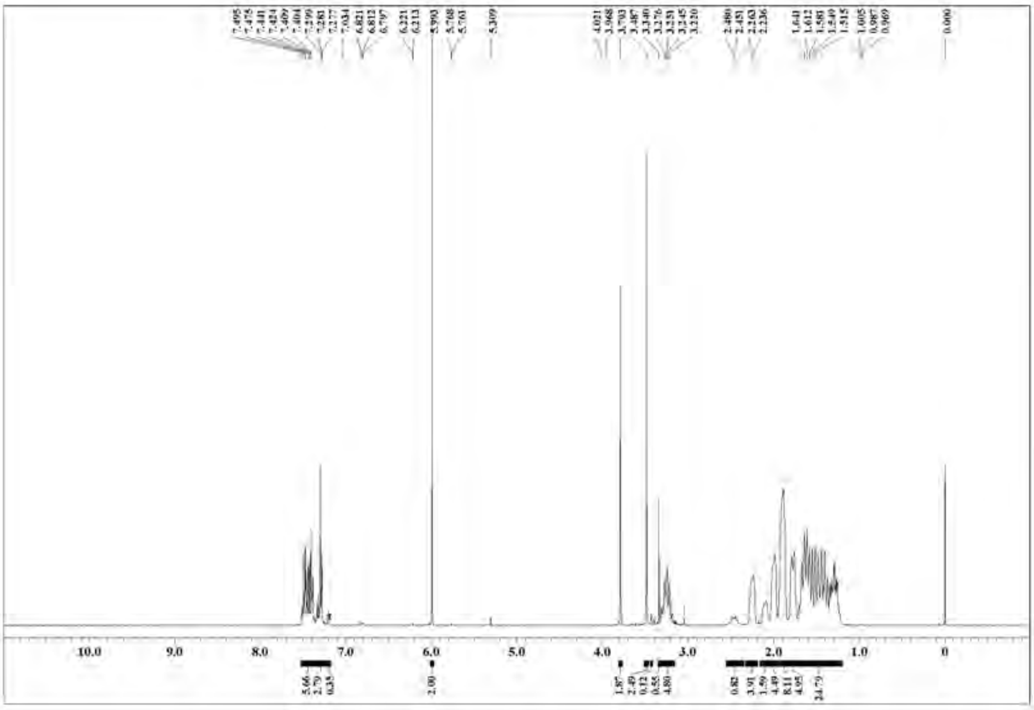

^1H-NMR (400 MHz, $CDCl_3$) spectrum of **1a-2**

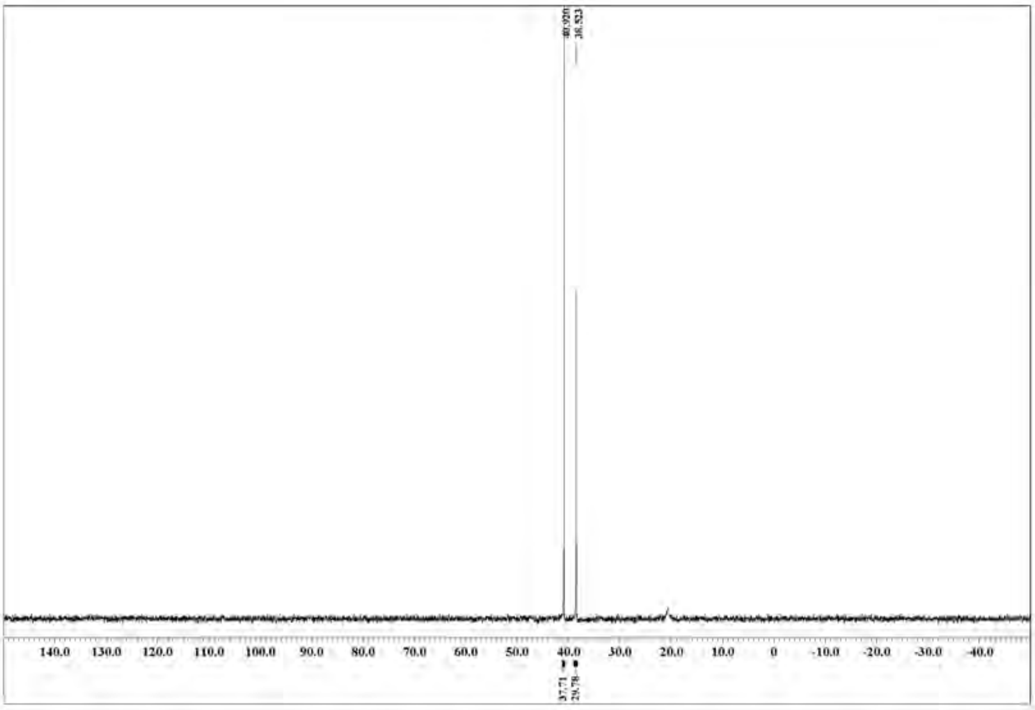

^{31}P-NMR (162 MHz, $CDCl_3$) spectrum of **1a-2**

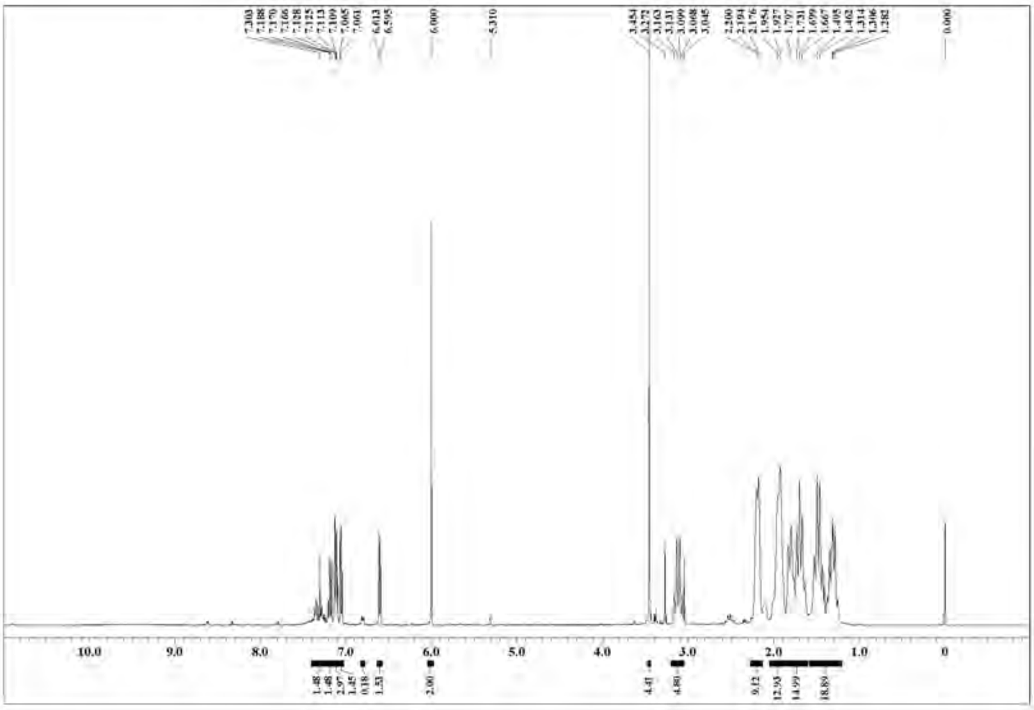

^{1}H-NMR (400 MHz, $CDCl_3$) spectrum of **2a**

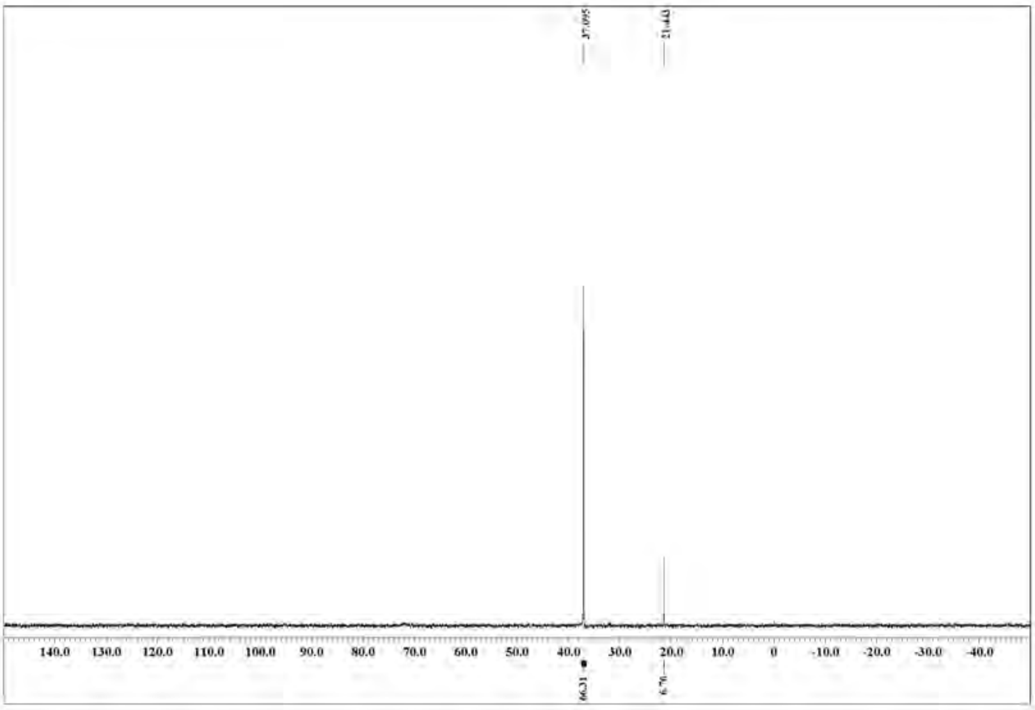

^{31}P-NMR (162 MHz, $CDCl_3$) spectrum of **2a**

10. DFT Calculation

All density functional theory (DFT) calculations were performed with the Gaussian 16 package.[7] The method B3LYP functional with the 6-31G(d) basis set was used for the geometry optimization and normal vibrational mode analysis. TD-DFT calculations were performed with the same level.

Conformational isomer **1a-2-C1**

Sum of electronic and thermal Free Energies= –1599.362060 (hartree)

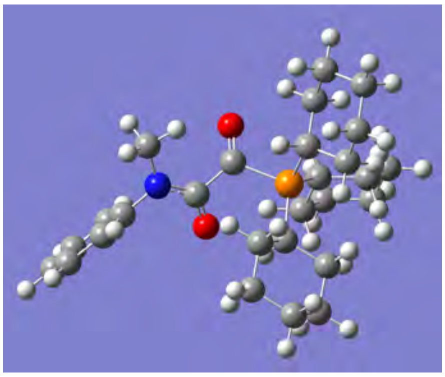

1 1

C	0.31250000	0.46875001	0.00000000
O	0.36583800	1.39725801	0.81034400
C	-1.09959400	0.26922601	-0.55774100
O	-1.36676300	0.34534201	-1.73440400
P	-2.56920100	0.27286701	0.68836200
C	-3.54811500	-1.23007299	0.14803300

C	-4.60483800	-1.70435899	1.17695300
C	-4.17803400	-1.06401099	-1.25888500
H	-2.77671300	-2.01320599	0.08347400
C	-5.27112500	-3.00883399	0.70067200
H	-5.37146300	-0.93093399	1.30767900
H	-4.14879400	-1.87042699	2.15695100
C	-4.84262400	-2.37719399	-1.70940800
H	-4.93760500	-0.27237799	-1.22601200
H	-3.42630900	-0.75453199	-1.98878500
C	-5.88544500	-2.86676299	-0.69721500
H	-6.03399700	-3.30325799	1.43040800
H	-4.52053600	-3.81218599	0.69279400
H	-5.30128300	-2.22219699	-2.69251300
H	-4.06891500	-3.14655299	-1.84457200
H	-6.30768500	-3.82444199	-1.02127200
H	-6.72132000	-2.15369399	-0.65922600
C	-3.42550600	1.88051801	0.30190700
C	-4.88992600	1.98109901	0.79181400
C	-2.59089000	3.09817401	0.77038200
H	-3.43281400	1.87059701	-0.79831700
C	-5.51853800	3.30141501	0.30648400
H	-4.92217900	1.94572501	1.88744000
H	-5.48459800	1.13816801	0.42655200
C	-3.23952900	4.40576701	0.28363800
H	-2.54338800	3.10626001	1.86748700
H	-1.55833000	3.02751201	0.41528800
C	-4.69900100	4.52301501	0.74240600
H	-6.54359600	3.36771701	0.68870600
H	-5.59410200	3.28032901	-0.78992000
H	-2.65052900	5.25320801	0.65208000
H	-3.19201200	4.44548401	-0.81378000
H	-5.14875200	5.43814501	0.34150100
H	-4.73162200	4.61294601	1.83769000
C	-1.91564800	0.13192701	2.42228700
C	-2.93004800	0.45551001	3.54597900
C	-1.22309700	-1.23465499	2.65312500
H	-1.13853100	0.90511501	2.43481100
C	-2.22484500	0.41168801	4.91490200
H	-3.75744300	-0.26323799	3.54058100
H	-3.36656600	1.44786001	3.39421400
C	-0.53670300	-1.25835599	4.03065700

H	-1.96274300	-2.04420799	2.60495000
H	-0.48047700	-1.43513299	1.87033800
C	-1.52451600	-0.93170299	5.15886400
H	-2.96190000	0.60930701	5.70147600
H	-1.48733500	1.22480201	4.96439600
H	-0.08391700	-2.24389499	4.18902800
H	0.28449100	-0.52872299	4.03304700
H	-1.00101300	-0.91236099	6.12111900
H	-2.27709600	-1.73029299	5.22916100
N	1.34486400	-0.27043099	-0.44864700
C	2.68297900	0.06118801	-0.00357200
C	3.41482900	-0.88650299	0.71556000
C	3.23875300	1.30179001	-0.32258900
C	4.71769100	-0.58923499	1.11458700
H	2.96832700	-1.84461299	0.96721500
C	4.54017000	1.59259901	0.08542400
H	2.65849100	2.02924801	-0.88068100
C	5.28081500	0.64926601	0.80042000
H	5.28935200	-1.32385499	1.67362500
H	4.97587100	2.55623101	-0.16048600
H	6.29525400	0.87867701	1.11235900
C	1.22537600	-1.31436199	-1.47836200
H	0.19114000	-1.63966099	-1.58378100
H	1.58069400	-0.93858999	-2.44292300
H	1.83657100	-2.17027699	-1.18618900

Conformational isomer **1a-2-C2** (more stable isomer)

Sum of electronic and thermal Free Energies= –1599.366068 (hartree)

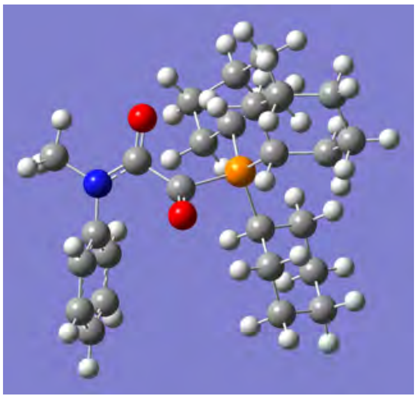

1 1

C	-0.07056452	0.36794355	0.00000000
O	0.74702848	1.27750955	0.15491700
C	0.56020348	-1.02499845	0.09892200

O	0.29339048	-1.80780545	0.97899700
P	2.11408348	-1.41479245	-0.96615300
C	1.59059048	-2.95674945	-1.88644400
C	2.52726148	-3.35003545	-3.05568600
C	1.34667848	-4.15927345	-0.93976300
H	0.62035548	-2.66454445	-2.31735000
C	1.95174748	-4.55674345	-3.82039000
H	3.52073748	-3.60551845	-2.66703200
H	2.66096948	-2.51392545	-3.74825700
C	0.77831348	-5.35385245	-1.72622600
H	2.29621948	-4.45491445	-0.47498200
H	0.66601448	-3.88742145	-0.12905100
C	1.68631948	-5.75184445	-2.89660300
H	2.64722048	-4.83372745	-4.62088500
H	1.01494448	-4.25581845	-4.31098800
H	0.64181748	-6.19704145	-1.03967600
H	-0.22096152	-5.09375645	-2.10356300
H	1.23450148	-6.57266345	-3.46467300
H	2.64123048	-6.13133445	-2.50582000
C	3.38697948	-1.72420045	0.35255100
C	4.64087248	-2.51339045	-0.09055800
C	3.77118148	-0.40802245	1.07411800
H	2.82151248	-2.34828945	1.06013200
C	5.54268848	-2.79330645	1.12702000
H	5.20509148	-1.93881145	-0.83502600
H	4.36035348	-3.46050245	-0.56260900
C	4.67682848	-0.70987845	2.28075000
H	4.30887148	0.24819155	0.37698800
H	2.87866648	0.14013855	1.39435100
C	5.92316248	-1.50550445	1.86984200
H	6.44060948	-3.32368845	0.78992300
H	5.01698048	-3.47232345	1.81312500
H	4.96245848	0.23472555	2.75712100
H	4.10463448	-1.27713345	3.02817800
H	6.52485448	-1.74679945	2.75312100
H	6.55637948	-0.88313645	1.22133100
C	2.50035648	0.01681655	-2.08680500
C	3.91842948	0.00151055	-2.70819000
C	1.40996048	0.20735755	-3.16999600
H	2.43778048	0.86883655	-1.39877200
C	4.15296448	1.29322755	-3.51379800

H	4.04025948	-0.86569345	-3.36777100
H	4.67987448	-0.08160545	-1.92642600
C	1.66914248	1.49815055	-3.96735800
H	1.41125648	-0.64800345	-3.85785300
H	0.41213448	0.25005655	-2.71682900
C	3.07443548	1.50823555	-4.58385400
H	5.14708348	1.25095855	-3.97307200
H	4.16216748	2.14878955	-2.82420600
H	0.90591548	1.59791055	-4.74773700
H	1.55147748	2.36113155	-3.29779700
H	3.24777148	2.45540255	-5.10649800
H	3.14695148	0.71543655	-5.34228200
N	-1.40166852	0.48764955	-0.15471400
C	-2.25867752	-0.65303045	-0.38371200
C	-2.90261052	-1.26769445	0.69303600
C	-2.47048352	-1.10117145	-1.69093300
C	-3.75520852	-2.34552645	0.45691400
H	-2.72373252	-0.91081045	1.70251500
C	-3.32413852	-2.18287945	-1.91945800
H	-1.98999652	-0.59048045	-2.52140400
C	-3.96561052	-2.80381245	-0.84652200
H	-4.25422252	-2.82834845	1.29159300
H	-3.49599452	-2.52960245	-2.93419200
H	-4.63406952	-3.64075245	-1.02508700
C	-2.02170852	1.82071655	-0.09457600
H	-1.23655252	2.56398455	0.04284800
H	-2.56640952	2.01566655	-1.02269100
H	-2.72196552	1.86340855	0.74493700

HOMO and LUMO of Conformational isomer **1a-2-C2**

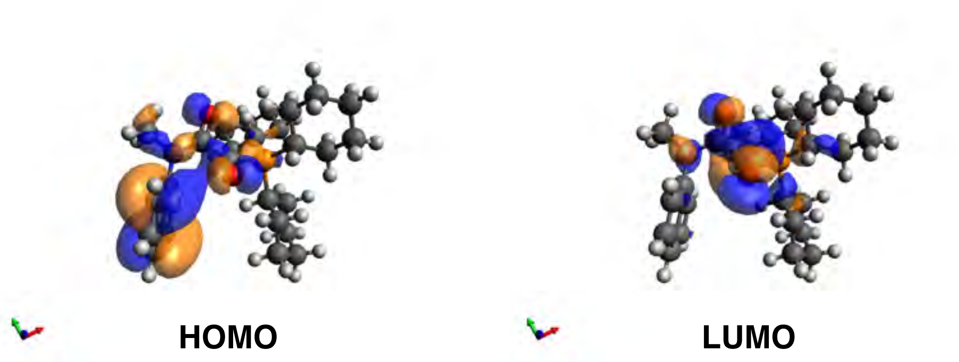

HONTO and LUNTO of Conformational isomer **1a-2-C2** [Natural Transition Orbital (NTO) analysis for the S_1 transition]

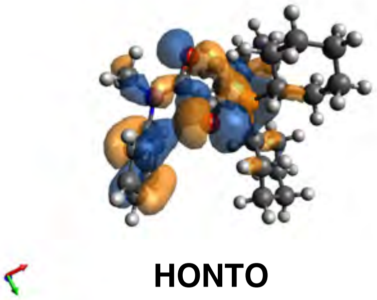

HONTO

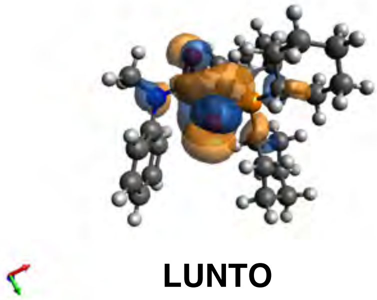

LUNTO

Comments on a red-colored species observed during time-resolved electron paramagnetic resonance (TREPR) of 1a-2

In the measurement of TREPR of **1a-2**, the originally yellowed sample turned red at 80K after a laser irradiation. When the sample was brought to room temperature, the sample returned to yellow. We estimated the red-colored species would be singlet species, such as **B** shown in Fig. 4F. Although the TD-DFT calculations of **1a-2(-C2)** and **B** (derived from **1a**) were performed, the calculation for **B** was unsuccessful. Therefore, we have not identified the red-colored species yet.

Result of TD-DFT calculation of Conformational isomer **1a-2-C2**

Excited State 1:	Singlet-A	3.2329 eV	383.51 nm	f=0.0110	<S**2>=0.000
107 ->121	0.10218				
117 ->121	0.27595				
118 ->121	-0.30261				
120 ->121	0.55239				

The above wavelength of the excited state 1 (383.51 nm) well explains the red-shfted band around 400 nm in Fig. 4A, strongly supporting the SET mechanism via A in Fig. 4F.

11. Reactions with Labelled Substrate or Reagent

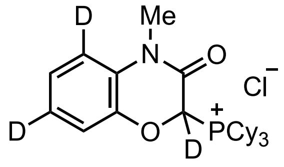

71%D

Fig. 4E, left. The reaction was carried out according to procedure A using **1a-D**. The product **2a-D** was purified by recrystallization by sonication of a THF, hexane solution. (Fig 4E; 414.5 mg, 0.86 mmol, 86% isolated yield, 71%D). White solid. **M.p.** 238–243 °C (decomp.). **IR** (neat) 557, 894, 1072, 1381, 1441, 1468, 1664, 2851, 2920 cm $^{-1}$. The ratio of deuterium incorporation was determined by ^{1}H-NMR analysis. **^{1}H NMR** (400 MHz, $CDCl_3$) δ 7.05 (d, J = 1.2 Hz, 1H), 7.16 (d, J = 1.2 Hz, 1H), 6.86 (d, J = 6.8 Hz, 0.29H), 3.44 (s, 3H), 3.18 (q, J = 12.8 Hz, 3H), 2.24–2.17 (m, 6H), 1.96–1.92 (m, 6H), 1.81 (d, J = 12.8 Hz, 3H), 1.73–1.65 (m, 6H), 1.49 (q, J = 12.8 Hz, 6H), 1.35–1.24 (m, 3H). **^{13}C NMR** (100.6 MHz, $CDCl_3$) δ 161.3 (d, $J_{C–D}$ = 5.9 Hz), 143.5 (d, $J_{C–P}$ = 9.0 Hz), 128.4 (d, $J_{C–D}$ = 3.3 Hz), 124.7 (m), 124.4, 116.9, 115.7 (t, $J_{C–D}$ = 25.9 Hz), 70.9 (d, $J_{C–P}$ = 59.8 Hz), 31.7 (dd, J = 35.4, 2.2 Hz), 29.1, 27.7 (d, $J_{C–P}$ = 3.7 Hz), 27.7 (d, $J_{C–P}$ = 3.2 Hz), 26.7 (d, $J_{C–P}$= 12.3 Hz), 26.7 (d, $J_{C–P}$ = 12.3 Hz), 25.5. **^{31}P NMR** (162 MHz, $CDCl_3$) δ 36.5. **HRMS–ESI** (*m/z*): $[M–Cl]^+$ calcd for $C_{27}H_{38}D_3NO_2P$; 445.3058, found 445.3067.

Fig. 4E, right. 1,4-Benzoxazinone phosphonium salt **2a** (239 mg, 0.5 mmol) was dissolved into AcOD (2 mL) and evacuate the solvent at 40°C. Then, The ration of deuterium incorporation was determined by ^{1}H-NMR analysis. The shift of peaks was observed due to the residual acetic acid. **^{1}H NMR** (400 MHz, $CDCl_3$) δ 7.18 (m, 1H), 7.13–7.10 (m, 2H), 7.06 (m, 1H), 6.32 (d, J = 6.8 Hz, 0.1H), 3.46 (s, 3H), 2.97 (q, J = 12.4 Hz, 3H), 2.14–2.09 (m, 6H), 1.95–1.92 (m, 6H), 1.81 (d, J = 12.4 Hz, 3H), 1.73–1.67 (m, 6H), 1.44 (q, J = 12.4 Hz, 6H), 1.34–1.30 (m, 3H).

12. Reaction with Asymmetric Diarylamine

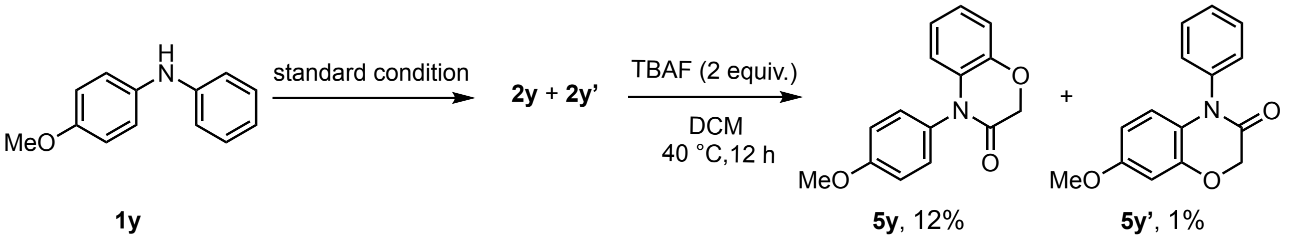

Fig. 4D. 2y was prepared according to Procedure A (48 h) and utilized for deprotection without purification. Then, **2y** (1 mmol) was dissolved with DCM (10 ml). Then, TBAF (1.0 mol/L THF, 2 mL, 2.0 equiv.) and H_2O (90 µL, 5 mmol) was sequentially and slowly added at the room temperature. The reaction was stirred at 40 °C for 12 hours and quenched with water. The resultant reaction solution was extracted with ethyl acetate three times and the combined organic layer was dried over Na_2SO_4 and filtered. After volatiles were removed under reduced pressure, purification by flash column chromatography on silica gel gave the dephosphorylated product (33.3 mg, 13% isolated yield).

4-(4-Methoxyphenyl)-2*H*-benzo[*b*][1,4]oxazin-3(4*H*)-one (5y, major)
7-Methoxy-4-phenyl-2*H*-benzo[b][1,4]oxazin-3(4*H*)-one (5y, minor)

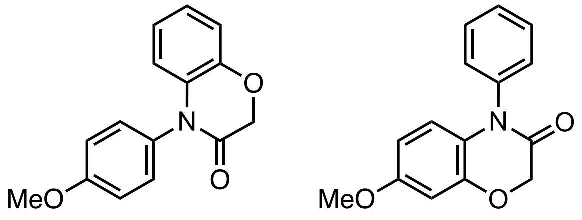

major (5y) **minor (5y')**

The product was purified by flash chromatography on silica gel (100:0–90:10, hexane/EtOAc) (Fig. 4D; 33.3 mg, 0.13 mmol, 13% isolated yield.). Thin orange solid. **M.p.** 114–120 °C. **IR** (neat) 1047, 1221, 1245, 1295, 1375, 1493, 1508, 1590, 1608, 1671, 1687 cm $^{-1}$. Signals for two regioisomer (9:1) were given: **^{1}H NMR** (400 MHz, $CDCl_3$) **Major regioisomer** (**5y**), the spectrum data of **S2** was consistent with the literature.[8]**:** δ 7.22–7.19 (m, 2H), 7.06–7.03 (m, 3H), 6.99 (td, J = 7.2, 1.8 Hz, 1H), 6.86 (m, 1H), 6.63 (dd, J = 5.6, 1.2 Hz, 1H), 4.78 (s, 2H), 3.87 (s, 3H). **Minor regioisomer** (**5y'**)**:** δ 7.53 (m, 2H), 7.45 (m, 1H), 7.30–7.29 (m, 2H), 6.63 (d, J = 3.0 Hz, 1H), 6.41 (dd, J = 9.0, 3.0 Hz, 1H), 6.35 (d, J = 9.0 Hz, 1H), 4.76 (s, 2H), 3.77 (s, 3H). Signals for two regioisomer were given: **^{13}C NMR** (100.6 MHz, $CDCl_3$) δ 164.5, 159.7, 144.9, 130.8, 129.9, 129.8, 128.6, 128.1, 124.0, 122.5, 117.5, 116.9, 116.8, 115.3, 68.3, 68.2, 55.6, 55.5 (only observed peaks). **HRMS–DART** (*m/z*): $[M+H]^+$ calcd for $C_{15}H_{14}NO_2$; 256.0968, found 256.0966.

13. Synthesis of Oxazolidinone via Photoexcitation of α-Ketoacylphosphonium

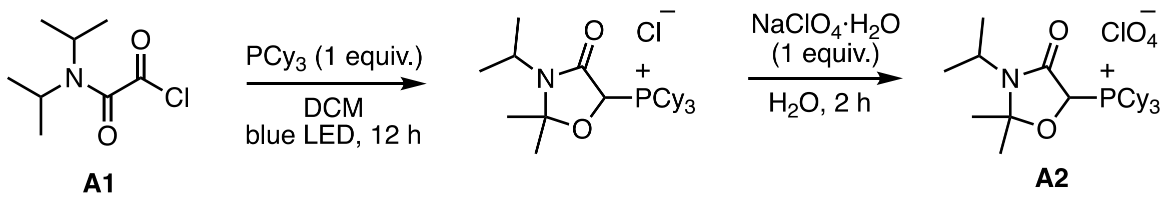

Procedure. In a glovebox, to an oven-dried vial with a stirring bar was added 2-(diisopropylamino)-2-oxoacetyl chloride **A1** (39.3 mg, 0.2 mmol, 1.0 equiv.) and DCM (2 mL). Then tricyclohexylphosphine (56.1 mg, 0.2 mmol, 1.0 equiv.) was slowly added to the solution. Then, the vial was sealed and brought outside the glove box. After stirring for 1 hour, the reaction was stirred and irradiated with 440 nm blue LED with a cooling fan to keep the temperature around 40 °C. After 12 hours of stirring, the solvent was removed using a rotary evaporator. To the crude material was added solution of $NaClO_4·H_2O$ (28.1 mg, 0.2 mmol, 1.0 equiv.) in 1 mL of H_2O and stirred for 2 hours. The resultant solution was extracted with DCM three times and the combined organic layer was dried over Na_2SO_4 and filtered. After volatiles were removed under reduced pressure, the obtained solid was dissolved in THF. Addition of hexane to the solution under sonication gave the white precipitate. The precipitate was collected by filtration and washed with small amount of THF. After drying for 30 min under vacuum, **A2** (72.9 mg, 0.14 mmol, 68% isolated yield.). was obtained as a white solid.

Tricyclohexyl(3-isopropyl-2,2-dimethyl-4-oxooxazolidin-5-yl)phosphonium perchlorate (A2)

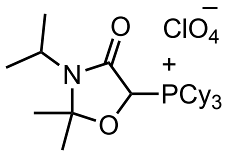

White solid. **M.p.** 273–290 °C (decomp.). **IR** (neat) 1086, 1262, 1348, 1430, 1701, 2860, 2933 cm $^{-1}$. **^{1}H NMR** (600 MHz, $CDCl_3$) δ 5.48 (s, 1H), 3.47 (sec, J = 6.6 Hz, 1H), 2.82 (qt, J = 12.6, 3 Hz, 3H), 2.15–2.07 (m, 6H), 1.96–1.94 (m, 6H), 1.81 (d, J = 12.6 Hz, 3H), 1.72–1.61 (m, 6H), 1.63 (s, 3H), 1.59 (s, 3H), 1.47 (q, J = 6.6 Hz, 3H), 1.46–1.39 (m, 6H), 1.43 (d, J = 6.6 Hz, 3H), 1.34–1.27 (m, 3H) . **^{13}C NMR** (100.6 MHz, $CDCl_3$) δ 163.9, 98.4 (d, J_{C-P} = 4.7 Hz), 67.6 (d, J_{C-P} = 58.4 Hz), 47.0, 30.3 (d, J_{C-P} = 35.5 Hz), 27.0 (d, J_{C-P} = 4.7 Hz), 27.0 (d, J_{C-P} = 3.8 Hz), 26.5 (d, J_{C-P}= 11.5 Hz), 26.4 (d, J_{C-P}= 11.5 Hz), 25.4, 25.3, 20.0, 19.4. **^{31}P NMR** (162 MHz, $CDCl_3$) δ 30.7. **HRMS–ESI** (*m/z*): $[M–ClO_4]^+$ calcd for $C_{26}H_{47}NO_2P$; 436.3339, found 436.3333.

Plausible mechanism for formation of A2. As with the aniline substrates, the phosphonium salt **A1-1** from **A1** becomes an excited state by photoirradiation. The excited state would undergo a charge tranfer from amine to a-ketoacylphosphonium (**A1-2**) followed by deprotonation in the radical ion pair[9] or 1,5-HAT on a biradical state (**A1-2'**), producing **A1-3**. The electron transfer from α-amino radical to α-phosphonium radical to give **A1-4**. The cyclization followed by proton shift affords **A2'**.

Plausible mechanism

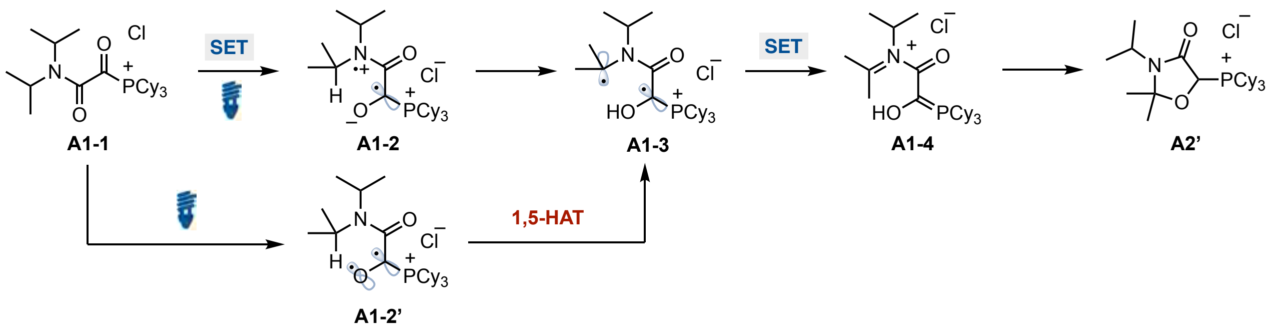

14. Reactions in the presence of Triplet Quenchers

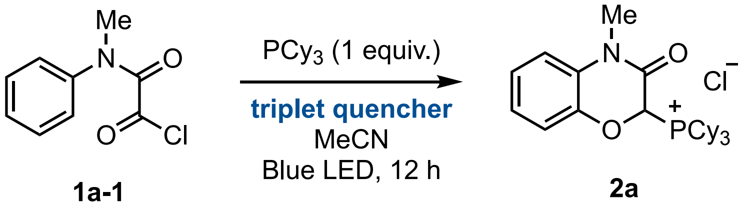

triplet quencher	yield of **2a** (%)
none	94
O_2 (bubbling)	65
azulene (1.5 equiv.)	67

Procedure (Fig. 4C). In a glovebox, to an oven-dried vial with a stirring bar was added α-ketoacyl chloride **1a-1** (0.2 mmol, 1.0 equiv.), MeCN (2 mL) and tricyclohexylphosphine (56.1 mg, 0.2 mmol, 1.0 equiv.). When azulene was used as a triplet quencher, azulene (38.5 mg, 0.3 mmol) was added in the glove box. Then the vial was sealed with a Teflon®-coated silicon rubber septum and brought outside the glove box and stirred for 1 h. When O_2 was used as a triplet quencher, the reaction mixture was bubbled with oxygen gas for 2 min. The reaction was stirred and irradiated with 440 nm blue LED with a cooling fan to keep the temperature around 40 °C (Fig. S1). After stirring for 12 h, the solvent was removed by evacuation. The yield of **2a** was determined by ^{1}H-NMR analysis using 1,1,2,2-tetrachloroethane.

15. Application to Synthesis of Flumioxazine

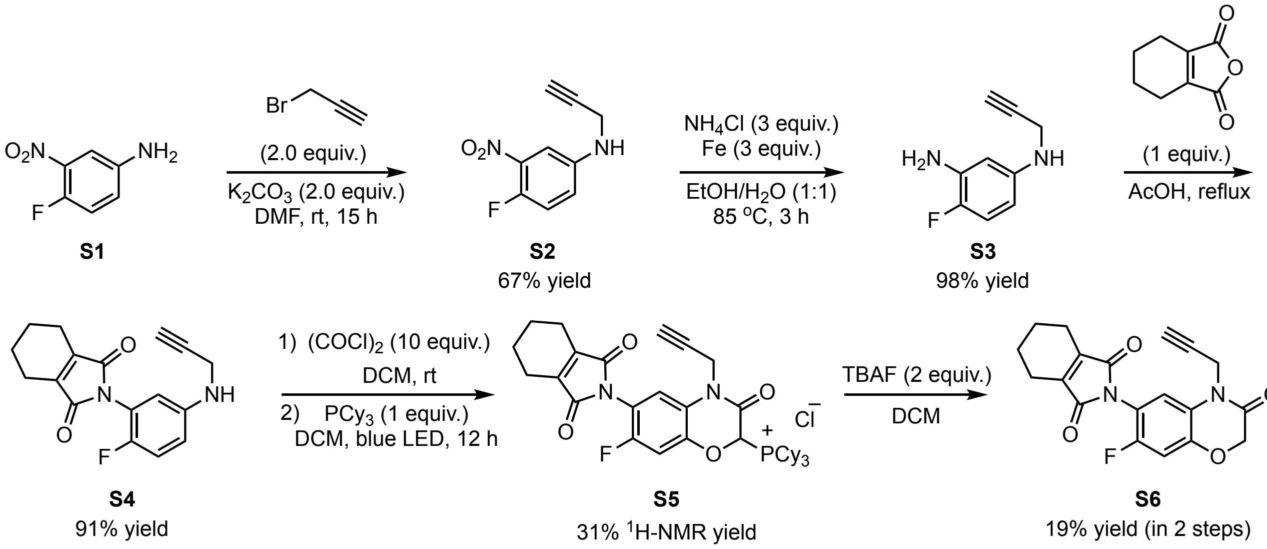

Propargylation. To a stirred solution of anhydrous K_2CO_3 (8.29 g, 80 mmol) in anhydrous DMF (60 mL) was added 4-Fluoro-3-nitroaniline **S1** (6.24 g, 40 mmol) and propargyl bromide (9.52 g, 6.02 mL, 80 mmol). This reaction mixture was stirred overnight at room temperature. The solvent was evaporated under reduced pressure and the residue was purified by silica gel chromatography (95:5–85:15, hexane/EtOAc) to give desired product **S2** (5.20 g, 67%).[10]

4-Fluoro-3-nitro-*N*-(prop-2-yn-1-yl)aniline (S2)

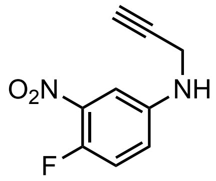

The product **S2** was purified by flash chromatography on silica gel (95:5–85:15, hexane/EtOAc) (5.20 g, 26.8 mmol, 67% isolated yield). Orange solid. The spectrum data of **S2** was consistent with the literature.[10]

Reduction of nitro group. To ammonium chloride (4.09 g, 76.5 mmol, 3 equiv.) in water was added iron powder (4.27 g, 76.5 mmol, 3 equiv.). To the resulting mixture was added 4-Fluoro-3-nitro-N-2-propyn-1-ylbenzenamine (4.96 g, 25.5 mmol, 1 equiv.) in H_2O/ethanol = 1:1 (100 mL). The resulting mixture was stirred vigorously at 85 °C for 3 hours. The reaction was quenched in water/ethyl acetate under stirring. The resulting 2 phase system was filtered and the layers were separated. The water layer was subsequently extracted with ethyl acetate. The combined organic phases were washed with brine, dried with Na_2SO_4 and the solvent was evaporated under reduced pressure and the residue was purified by silica gel chromatography (5-30% ethyl acetate in hexane) to give desired product (4.10 g, 98%).[11]

4-Fluoro-N^1-(prop-2-yn-1-yl)benzene-1,3-diamine (S3)

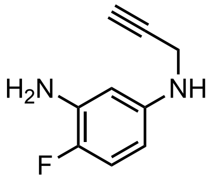

The product **S3** was purified by flash chromatography on silica gel (95:5–70:30, hexane/EtOAc) (4.10 g, 25.0 mmol, 98% isolated yield.). Light orange solid. **M.p.** 66–69 °C. **IR** (neat) 814, 1223, 1503, 1532, 1623, 3284, 3349, 3423 cm $^{-1}$. **^{1}H NMR** (400 MHz, $CDCl_3$) δ 6.83 (dd, J = 11.2, 8.0 Hz, 1H), 6.15 (dd, J = 7.6, 3.2 Hz, 1H), 6.01 (t, J = 8.0, 3.2 Hz, 1H), 3.85 (d, J = 2.4 Hz, 1H), 3.65 (broad, 1H), 2.21 (t, J = 2.4 Hz, 1H). **^{13}C NMR** (150.9 MHz, $CDCl_3$) δ 145.9 (d, $J_{C–F}$ = 229.7 Hz), 143.7 (d, $J_{C–F}$ = 1.8 Hz), 134.9 (d, $J_{C–F}$ = 14.0 Hz), 115.5 (d, $J_{C–F}$ = 19.5 Hz), 103.5 (d, $J_{C–F}$ = 6.5 Hz), 102.1 (d, $J_{C–F}$ = 2.6 Hz), 81.1, 71.2, 34.1. **^{19}F NMR** (376.5 MHz, $CDCl_3$) δ –147.4. **HRMS–DART** (*m/z*): $[M+H]^+$ calcd for $C_9H_{10}FN_2$; 165.0823, found 165.0823.

Condensation. A mixture of **D** (1.97 g, 12 mmol, 1 equiv.) and 3,4,5,6-Tetrahydrophthalic anhydride (1.83 g, 12 mmol, 1 equiv.) in acetic acid (72 mL) was heated under reflux for 1 h. The solvent was evaporated under reduced pressure and the residue was purified by silica gel chromatography (5-30% ethyl acetate in hexane) to give desired product (3.25 g, 91%).[12]

2-(2-Fluoro-5-(prop-2-yn-1-ylamino)phenyl)-4,5,6,7-tetrahydro-1*H*-isoindole-1,3(2*H*)-dione (S4)

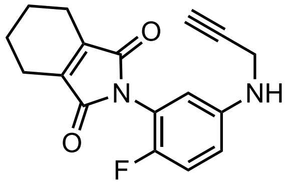

The product **S4** was purified by flash chromatography on silica gel (95:5–70:30, hexane/EtOAc) (3.25 g, 10.9 mmol, 91% isolated yield.). Light orange solid. **M.p.** 106–109 °C. **IR** (neat) 921, 1134, 1313, 1413, 1516, 1710, 2955, 3238, 3410 cm $^{-1}$. **^{1}H NMR** (400 MHz, $CDCl_3$) δ 7.03 (t, J = 9.2 Hz, 1H), 6.63 (m, 1H), 6.49 (m, 1H), 4.02 (broad, 1H), 3.84 (d, J = 2.0 Hz, 2H), 2.41 (s, 4H), 2.23 (t, J = 2.0 Hz, 1H), 1.80 (s, 4H). **^{13}C NMR** (150.9 MHz, $CDCl_3$) δ 169.1, 151.0 (d, $J_{C–F}$ = 241.7 Hz), 143.5, 142,1, 119.4 (d, $J_{C–F}$ = 14.5 Hz), 116.7 (d, $J_{C–F}$ = 21.0 Hz), 114.5 (d, $J_{C–F}$ = 7.2 Hz), 113.7, 80.4, 71.5, 33.7, 21.1, 20.1. **^{19}F NMR** (376.5 MHz, $CDCl_3$) δ–133.1. **HRMS–DART** (*m/z*): $[M+H]^+$ calcd for $C_{17}H_{16}FN_2O_2$; 299.1190, found 299.1192.

***o*-Oxidation and Dephosphorylation**. An oven-dried vial with a stirring bar was placed under N_2 atmosphere by three cycles of evacuating and N_2 backfilling and charged with oxalyl chloride (857.6 mg, 1.0 mmol, 10.0 equiv.) in dry DCM (5.0 mL). Then, **S4** (107.2 mg, 1.0 mmol, 1.0 equiv.) in dry DCM (5.0 mL) was added to the reaction mixture in shots with vigorous stirring at room temperature. And the mixture was stirred at room temperature for 2 hours and excess of oxalyl chloride was removed on a rotary evaporator. Then, the vial was brought to the glove box. To the reaction mixture was added DCM (10 mL) and tricyclohexylphosphine (280.4 mg, 1.0 mmol, 1.0 equiv.). After stirring

for 1 hour, the reaction was stirred and irradiated with 440 nm blue LED with a cooling fan to keep the temperature around 40 ℃. After stirring for 18 h, the solvent was removed using a rotary evaporator. Then the residual material (**S5**, 31% yield based on ^{1}H-NMR analysis) was dissolved with DCM (10 ml) in a glove box and treated with TBAF (1.0 mol/L THF, 2 mL, 2.0 equiv.) at room temperture. Then, water (5 equiv.) was added slowly. The reaction was stirred at 40℃ for 18 h. The solvent was evaporated under reduced pressure and the residue was purified by silica gel chromatography (95:5–85:15, hexane/EtOAc) gave **Flumioxazine (S6)** (66.5 mg, mmol, 19% yield in 2 steps).

Flumioxazine (S6)

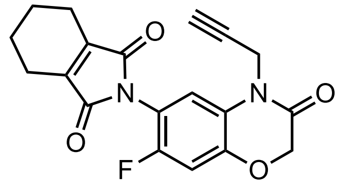

The product S6 was purified by flash chromatography on silica gel (95:5–85:15, hexane/EtOAc) (33.7 mg, 0.095 mmol, 10% isolated yield.). Off-white solid. **M.p.** 198–203 °C. **IR** (neat) 876, 1049, 1102, 1157, 1285, 1386, 1421, 1517, 1707, 3257 cm $^{-1}$. **^{1}H NMR** (600 MHz, C_6D_6) δ 7.09 (d, J = 7.2 Hz, 1H), 6.54 (d, J = 10.2 Hz, 1H), 4.11 (d, J = 2.4 Hz, 2H), 3.89 (S, 2H), 1.91 (quin, J = 3.6 Hz, 4H), 1.76 (t, J = 2.4 Hz, 1H), 1.08 (quin, J = 3.6 Hz, 4H). **^{1}H NMR** (400 MHz, $CDCl_3$) δ 7.05 (d, J = 7.2 Hz, 1H), 6.90 (d, J = 10.0 Hz, 1H), 4.68 (s, 2H), 4.66 (d, J = 2.4 Hz, 2H), 2.45 (quin, J = 3.6 Hz, 4H), 2.30 (t, J = 2.4 Hz, 1H), 1.84 (quin, J = 3.6 Hz, 4H). **^{13}C NMR** (150.9 MHz, C_6D_6) δ 169.0, 162.3, 154.6 (d, $J_{C–F}$ = 248.2 Hz), 146.1, 142,2, 125.4, 116.2, 114.4 (d, $J_{C–F}$ = 14.9 Hz), 105.9 (d, $J_{C–F}$ = 24.6 Hz), 77.6, 73.2, 67.3, 30.6, 21.1, 20.1. **^{19}F NMR** (376.5 MHz, $CDCl_3$) δ –122.3. **HRMS–DART** (*m/z*): $[M+H]^+$ calcd for $C_{19}H_{16}FN_2O_2$; 355.1089, found 355.1089.

■ **References** ■

(1) Jiao, J.; Zhang, X. R.; Chang, N. H.; Wang, J.; Wei, J. F.; Shi, X. Y.; Chen, Z. G. *J. Org. Chem.* **2011**, *76*, 1180−1183.

(2) Kwong, F. Y.; Klapars, A.; Buchwald, S. L. *Org. Lett.* **2002**, *4*, 581−584.

(3) Lad, U. P.; Kulkarni, M. A.; Desai, U. V.; Wadgaonkar, P. P. *C. R. Chim.* **2011**, *14*, 1059−1064.

(4) Noshita, M.; Shimizu, Y.; Morimoto, H.; Ohshima, T. *Organic Letters* **2016**, *18*, 6062–6065.

(5) Li, Y.; Wang, H.; Jiang, L.; Sun, F.; Fu, X.; Duan, C. *Eur. J. Org. Chem.* **2010**, *2010*, 6967–6973.

(6) Nakamura, A.; Yamamoto, K.; Murakami, R.; Kawashita, N.; Matsumoto, K.; Maegawa, T. *Synthesis* **2021**, *53*, 3862–3868.

(7) Gaussian 16, Revision C.01, Frisch, M. J.; Trucks, G. W.; Schlegel, H. B.; Scuseria, G. E.; Robb, M. A.; Cheeseman, J. R.; Scalmani, G.; Barone, V.; Petersson, G. A.; Nakatsuji, H.; Li, X.; Caricato, M.; Marenich, A. V.; Bloino, J.; Janesko, B. G.; Gomperts, R.; Mennucci, B.; Hratchian, H. P.; Ortiz, J. V.; Izmaylov, A. F.; Sonnenberg, J. L.; Williams-Young, D.; Ding, F.; Lipparini, F.; Egidi, F.; Goings, J.; Peng, B.; Petrone, A.; Henderson, T.; Ranasinghe, D.; Zakrzewski, V. G.; Gao, J.; Rega, N.; Zheng, G.; Liang, W.; Hada, M.; Ehara, M.; Toyota, K.; Fukuda, R.; Hasegawa, J.; Ishida, M.; Nakajima, T.; Honda, Y.; Kitao, O.; Nakai, H.; Vreven, T.; Throssell, K.; Montgomery, J. A., Jr.; Peralta, J. E.; Ogliaro, F.; Bearpark, M. J.; Heyd, J. J.; Brothers, E. N.; Kudin, K. N.; Staroverov, V. N.; Keith, T. A.; Kobayashi, R.; Normand, J.; Raghavachari, K.; Rendell, A. P.; Burant, J. C.; Iyengar, S. S.; Tomasi, J.; Cossi, M.; Millam, J. M.; Klene, M.; Adamo, C.; Cammi, R.; Ochterski, J. W.; Martin, R. L.; Morokuma, K.; Farkas, O.; Foresman, J. B.; Fox, D. J. Gaussian, Inc., Wallingford CT, 2016.

(8) Feng, E.; Huang, H.; Zhou, Y.; Ye, D.; Jiang, H.; Liu, H. *J. Org. Chem.* **2009**, *74*, 2846–2849.

(9) Chesta, C. A.; Whitten, D. G. *J. Am. Chem. Soc.* **1992**, *114*, 2188–2197.

(10) Kumar, A. R.; Wang, T.; Veedu, R. N.; Kumar, S. *Nucleosides Nucleotides Nucleic Acids* **2022**, *41*, 343–360.

(11) Wang, D.-W.; Zhang, H.; Yu, S.-Y.; Zhang, R.-B.; Liang, L.; Wang, X.; Yang, H.-Z.; Xi, Z. *J. Agric. Food Chem.* **2021**, *69*, 14115–14125.

(12) Huang, M.-Z.; Huang, K.-L.; Ren, Y.-G.; Lei, M.-X.; Huang, L.; Hou, Z.-K.; Liu, A.-P.; Ou, X.-M. *J. Agric. Food Chem.* **2005**, *53*, 7908–7914.

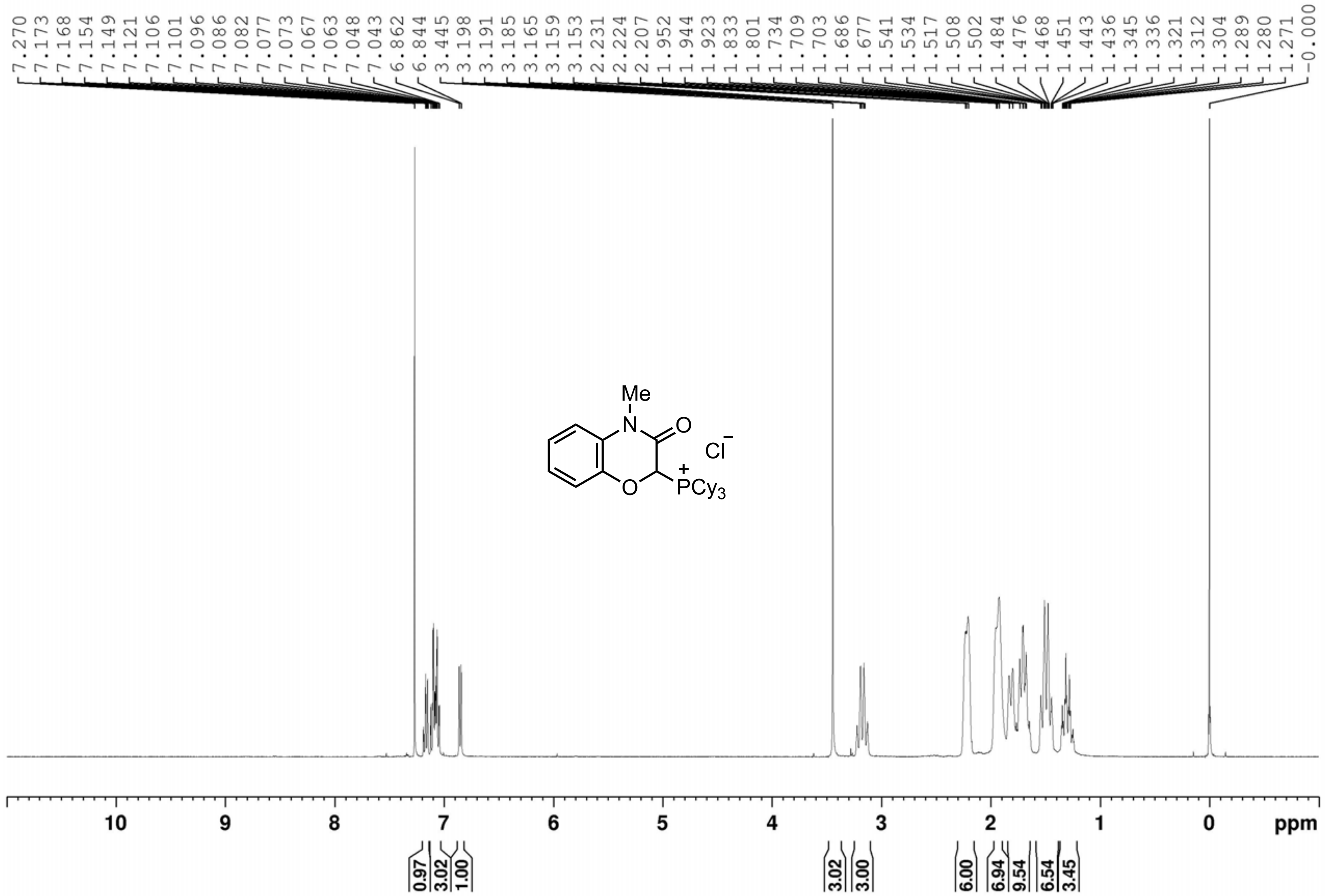

^{1}H NMR (400 MHz, $CDCl_3$) spectrum of **2a**

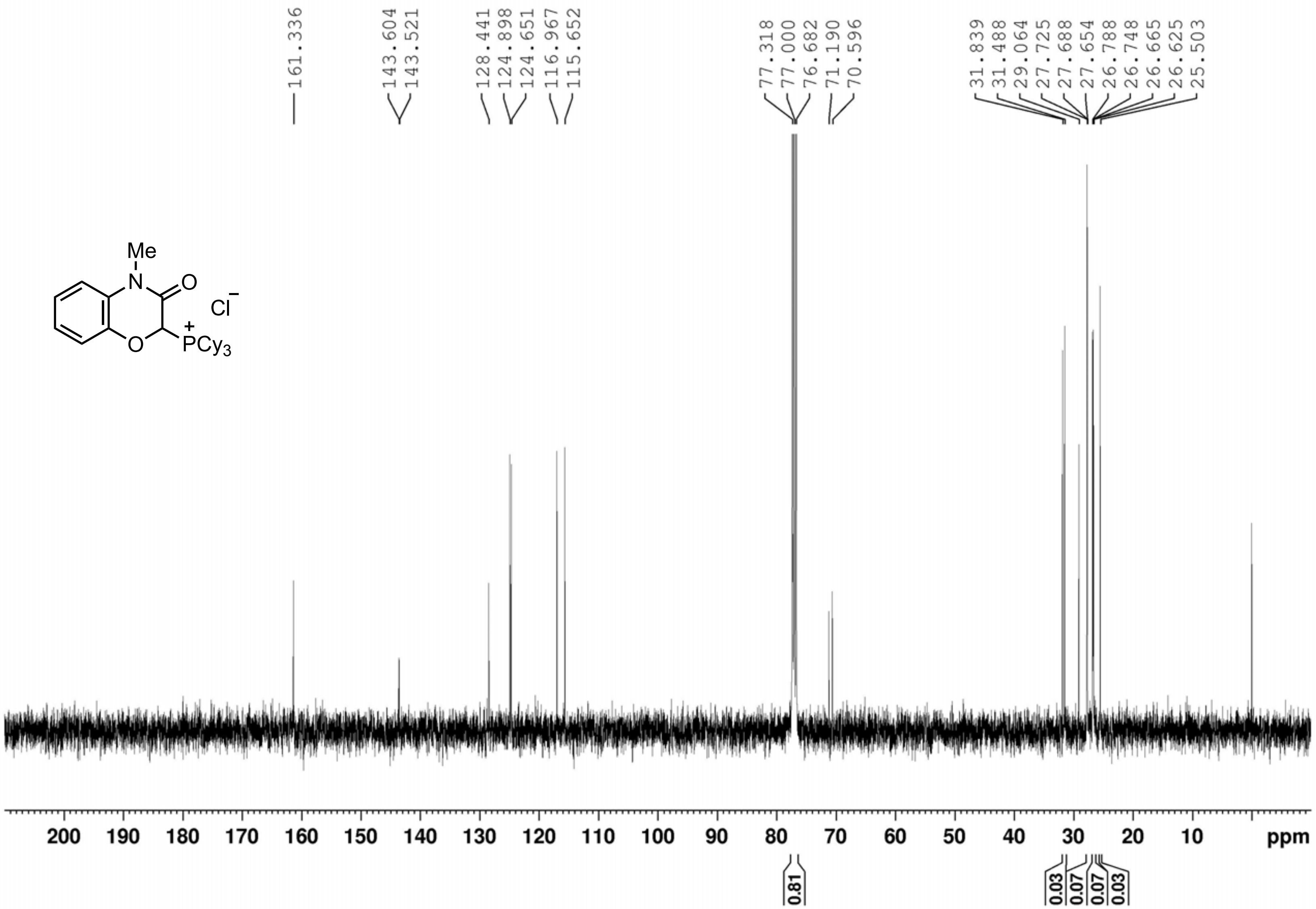

^{13}C NMR (100.6 MHz, $CDCl_3$) spectrum of **2a**

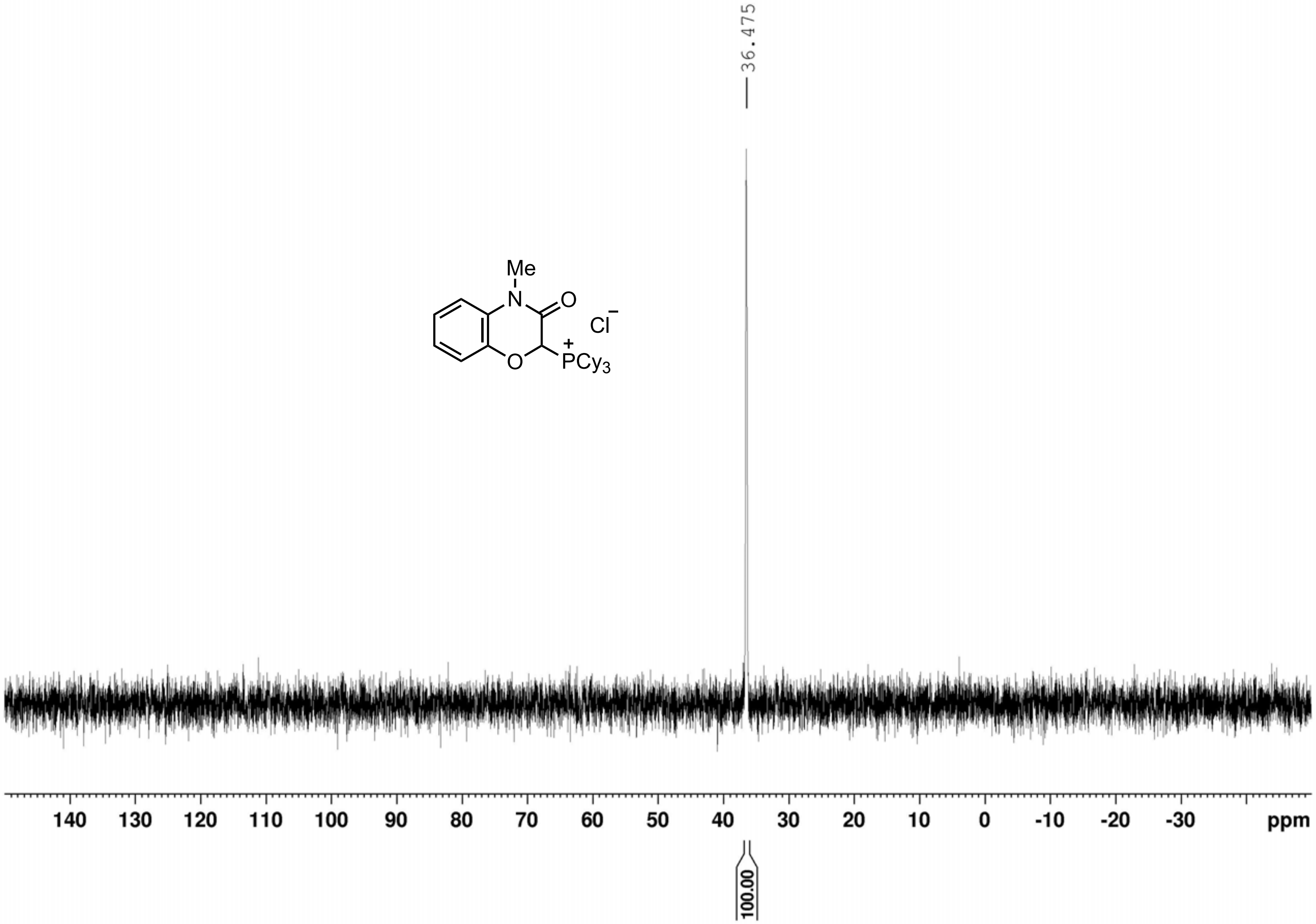

^{31}P NMR (162 MHz, $CDCl_3$) spectrum of **2a**

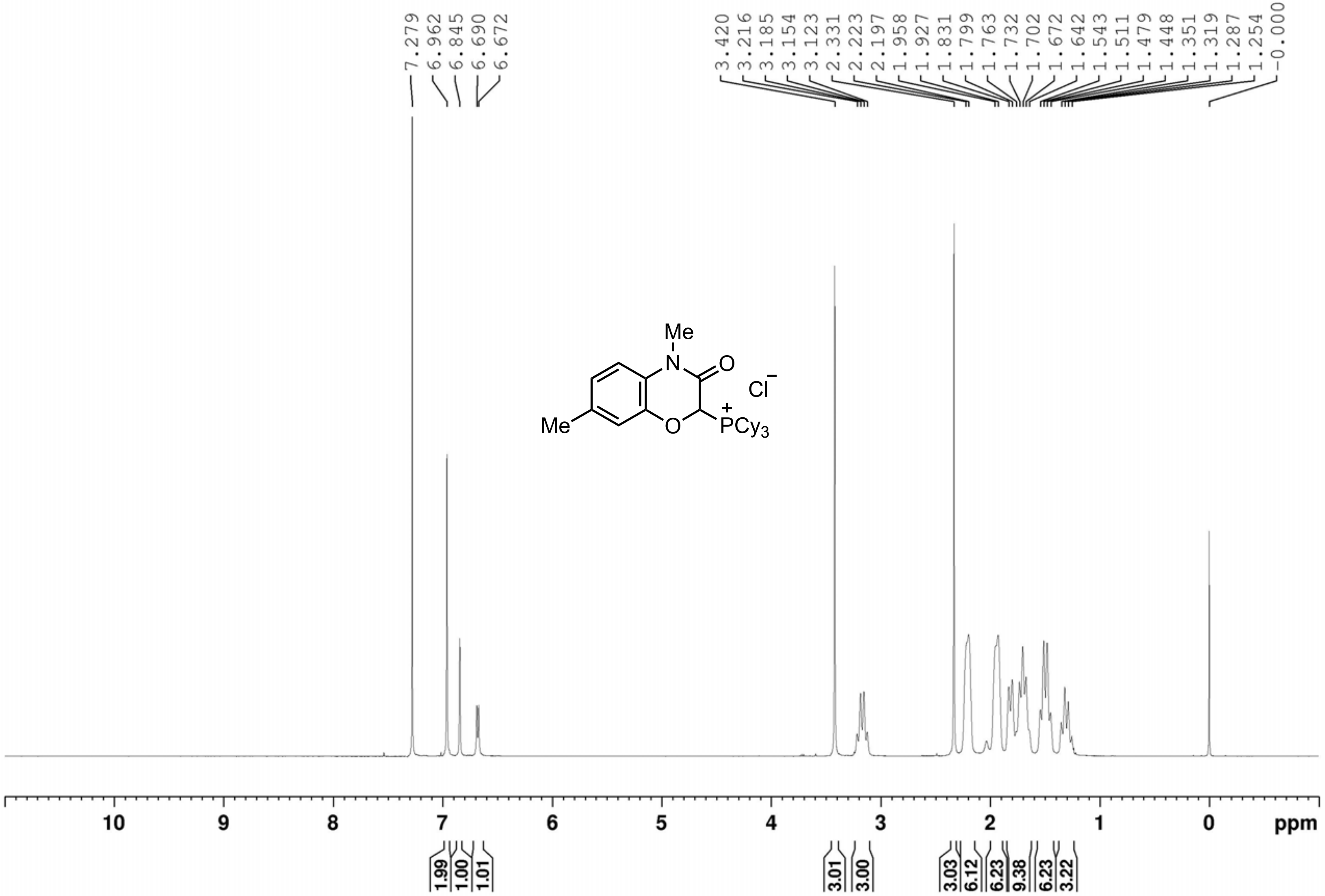

^{1}H NMR (400 MHz, $CDCl_3$) spectrum of **2b**

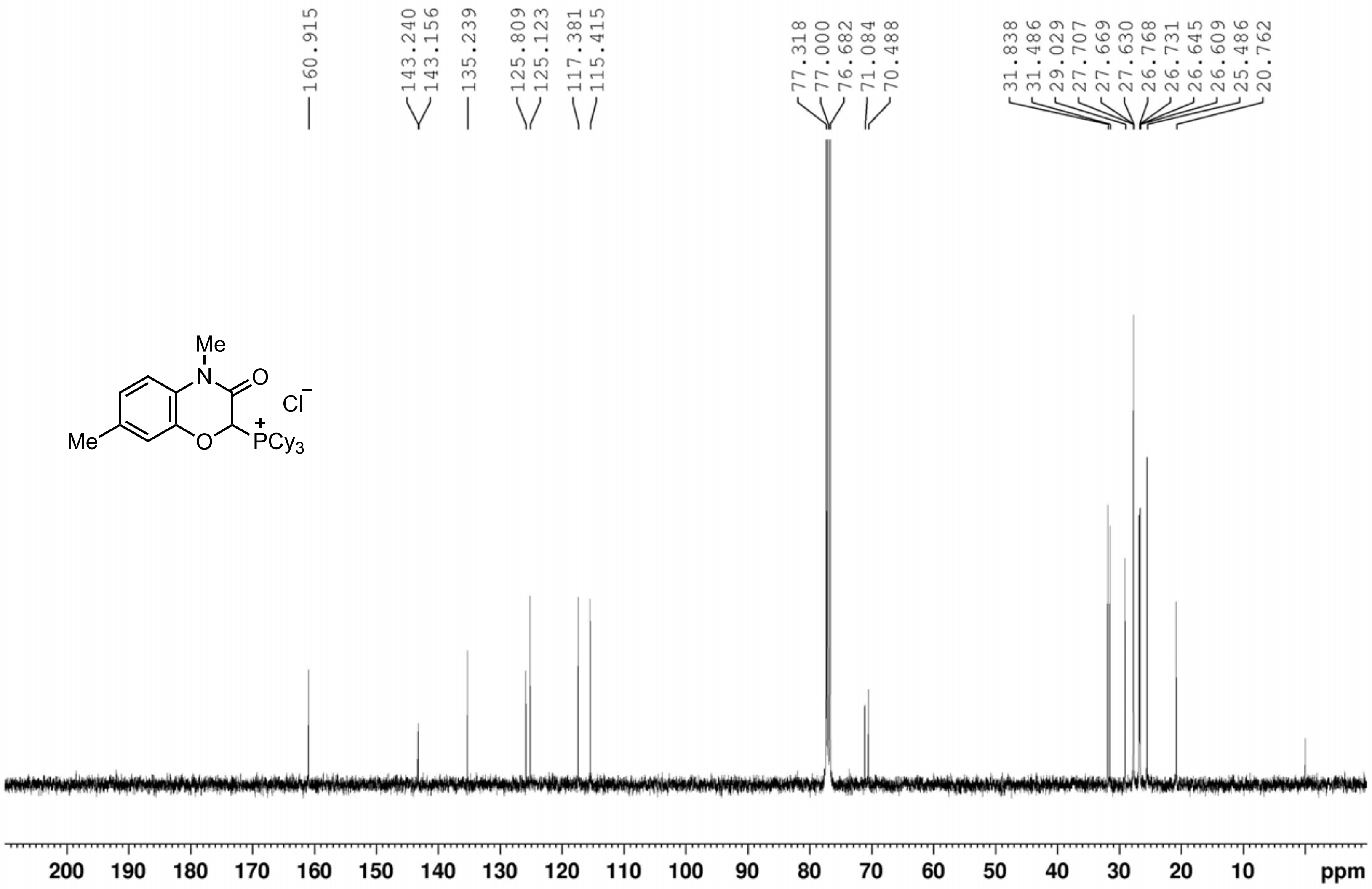

^{13}C NMR (100.6 MHz, $CDCl_3$) spectrum of **2b**

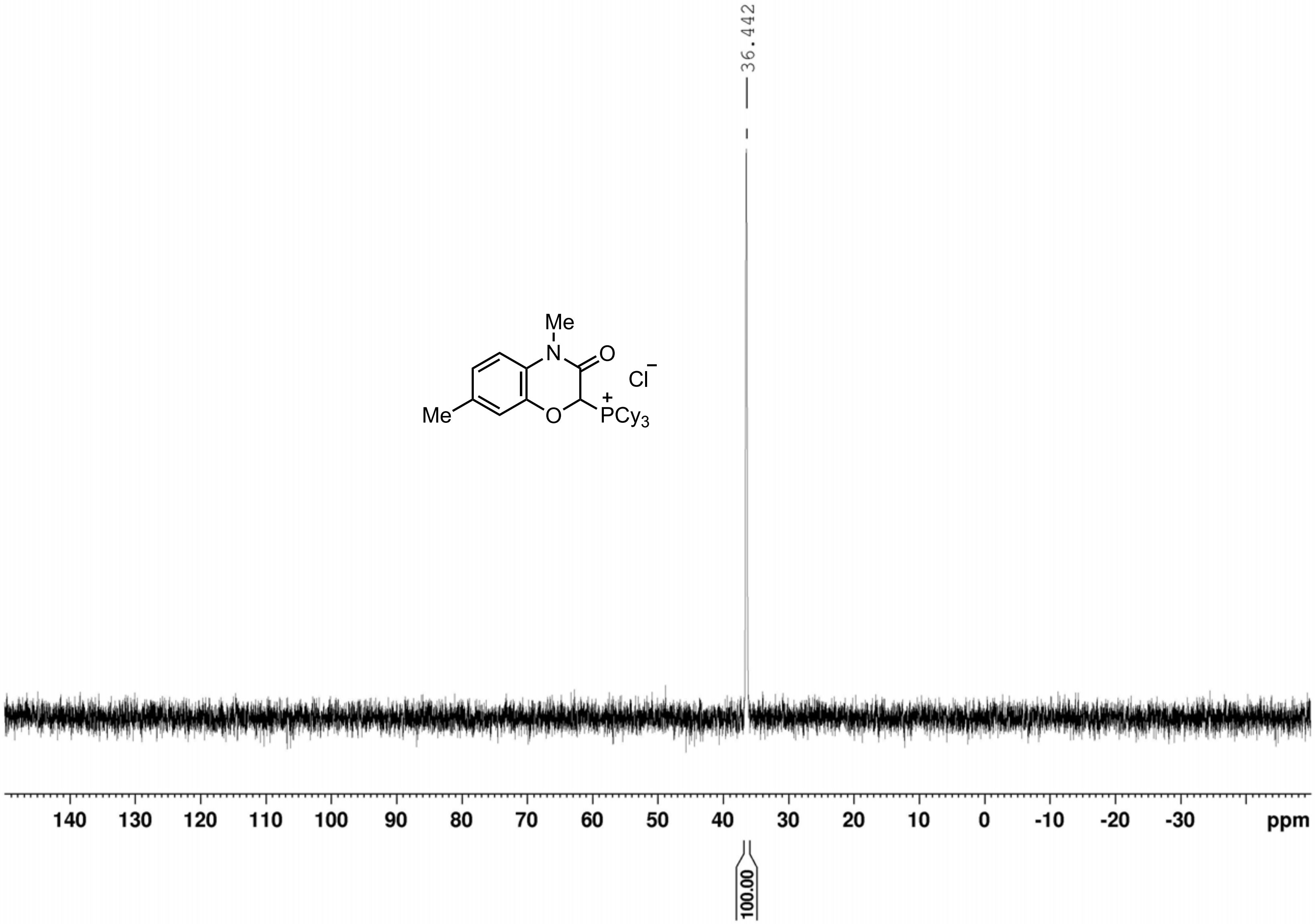

^{31}P NMR (162 MHz, $CDCl_3$) spectrum of **2b**

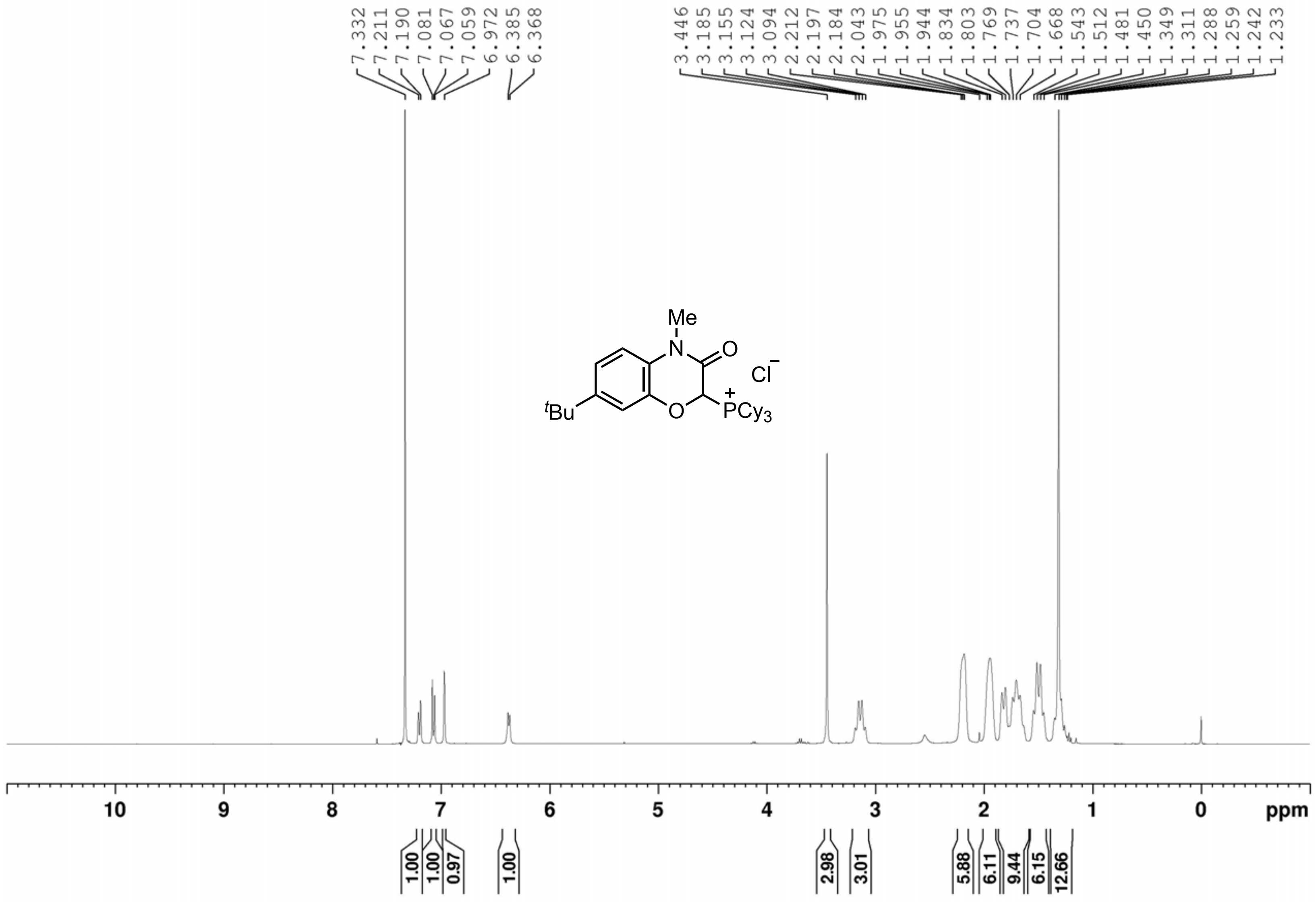

^{1}H NMR (400 MHz, $CDCl_3$) spectrum of **2c**

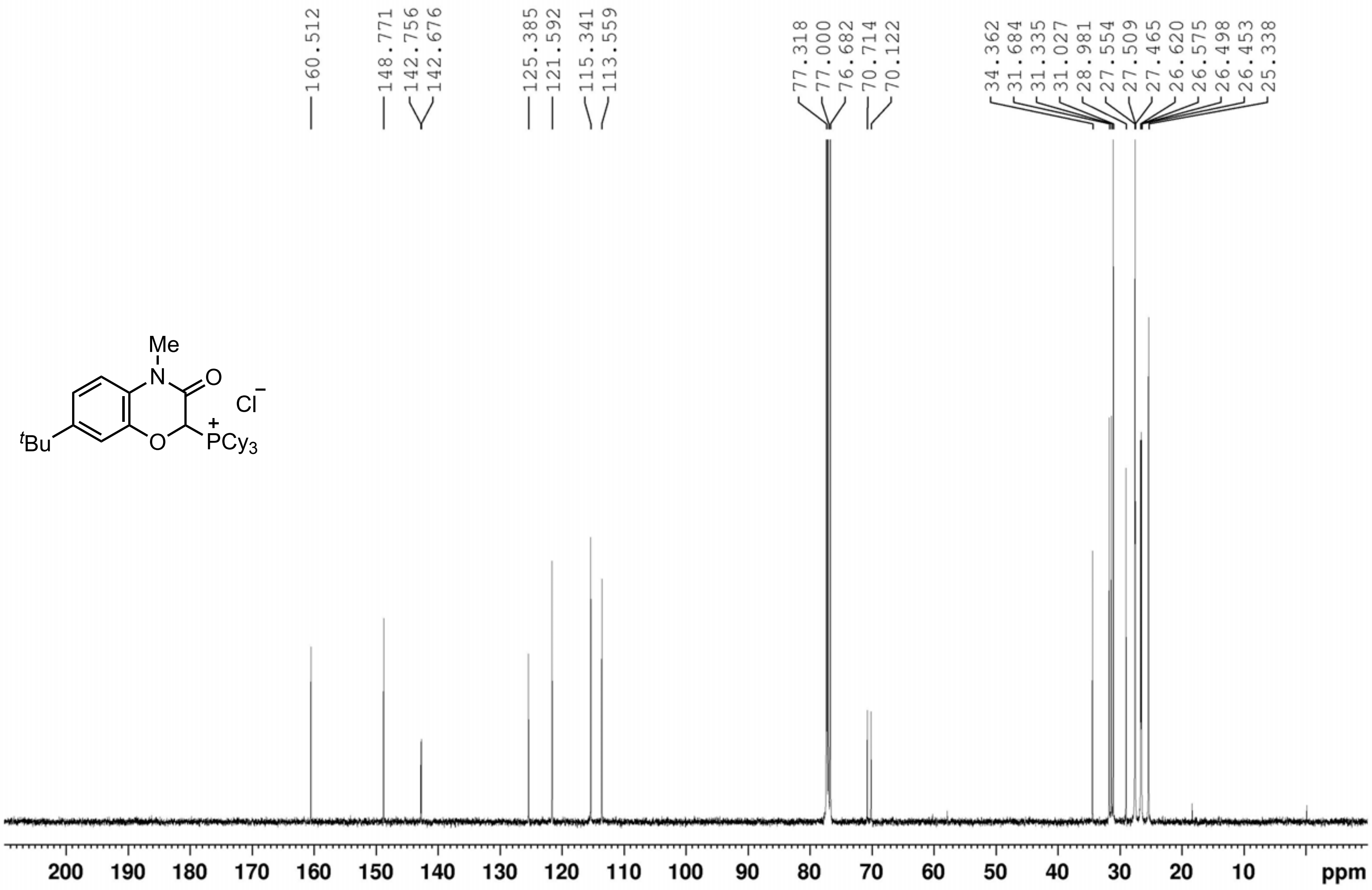

^{13}C NMR (100.6 MHz, $CDCl_3$) spectrum of **2c**

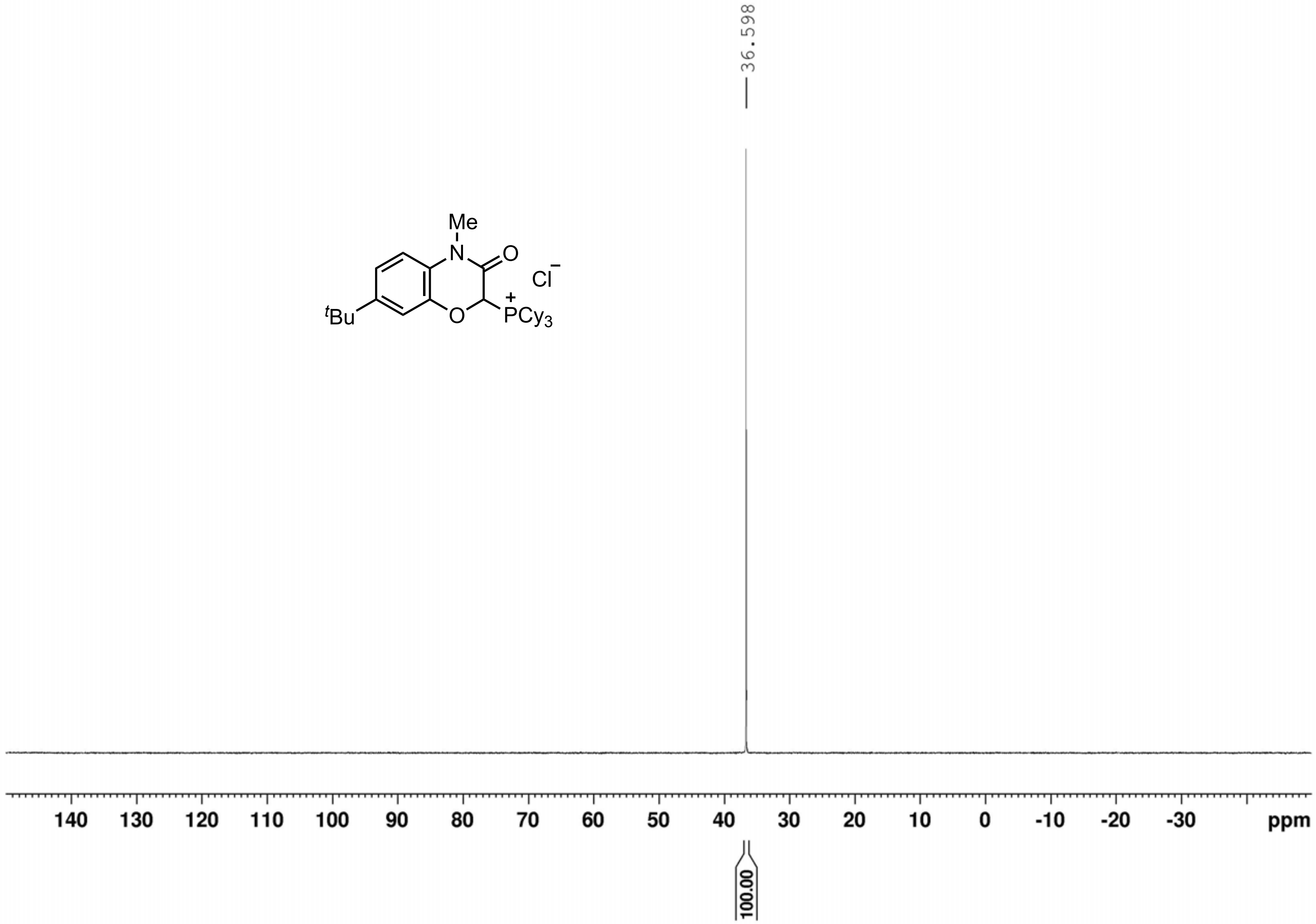

^{31}P NMR (162 MHz, $CDCl_3$) spectrum of **2c**

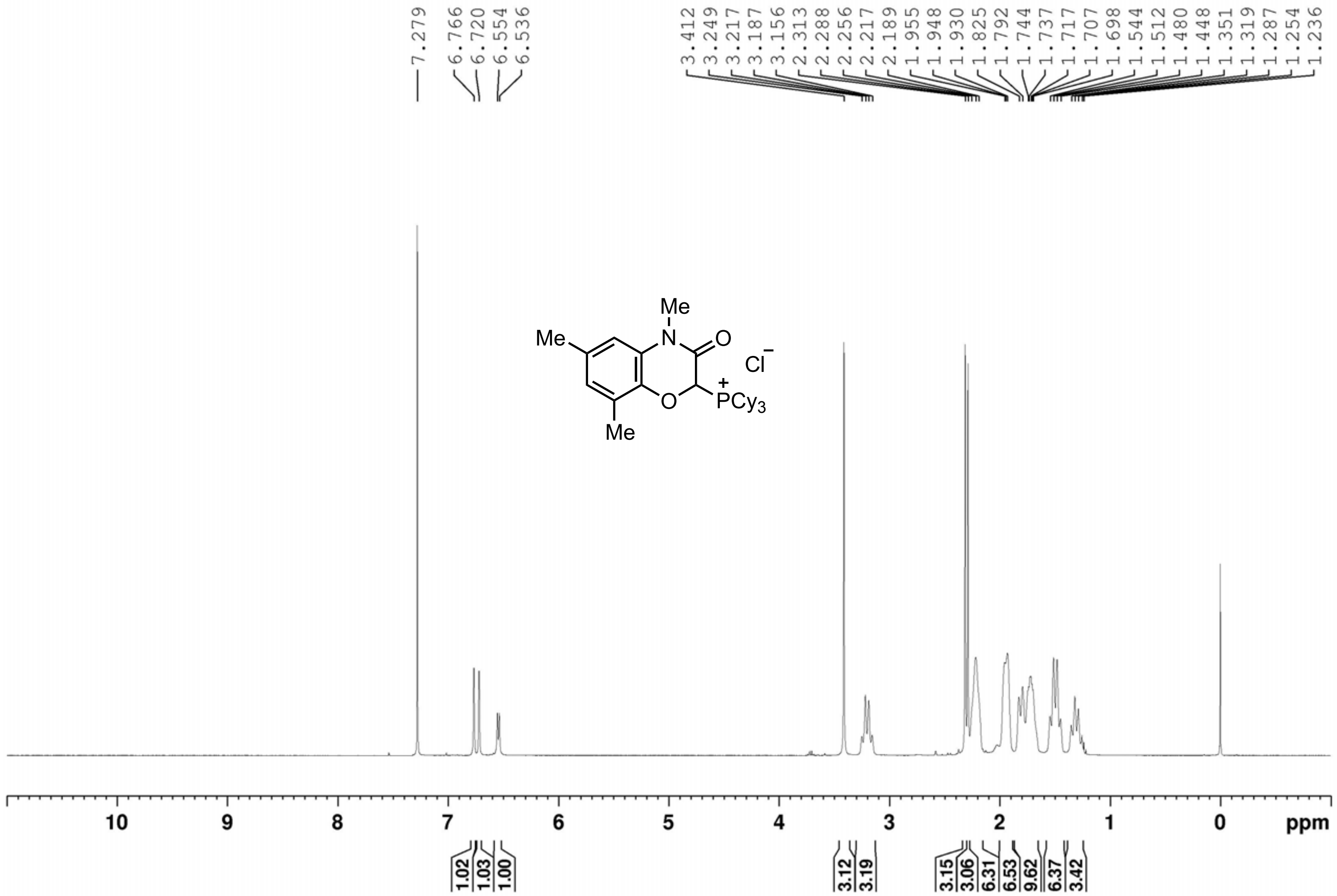

^{1}H NMR (400 MHz, $CDCl_3$) spectrum of **2d**

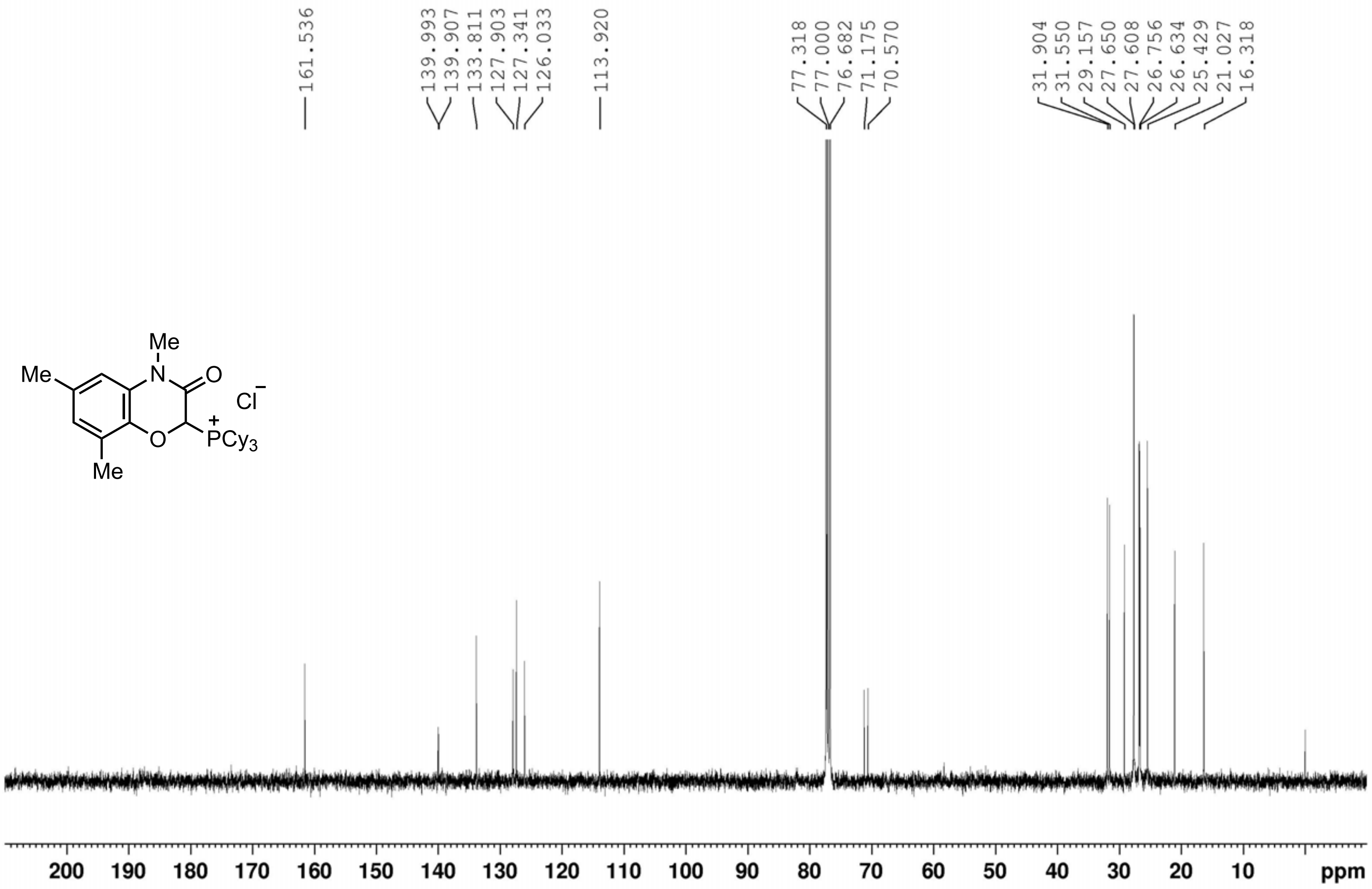

^{13}C NMR (100.6 MHz, $CDCl_3$) spectrum of **2d**

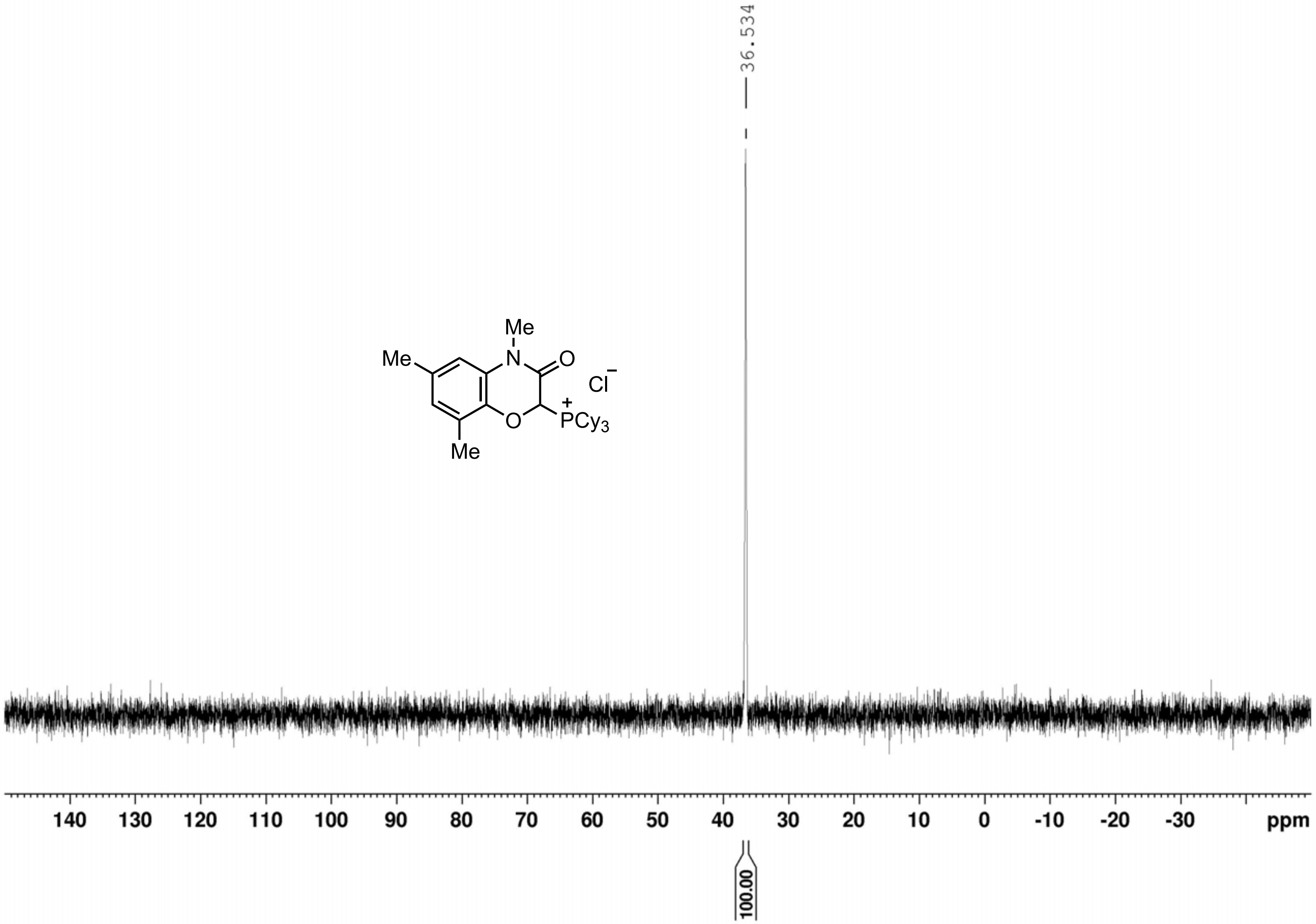

^{31}P NMR (162 MHz, $CDCl_3$) spectrum of **2d**

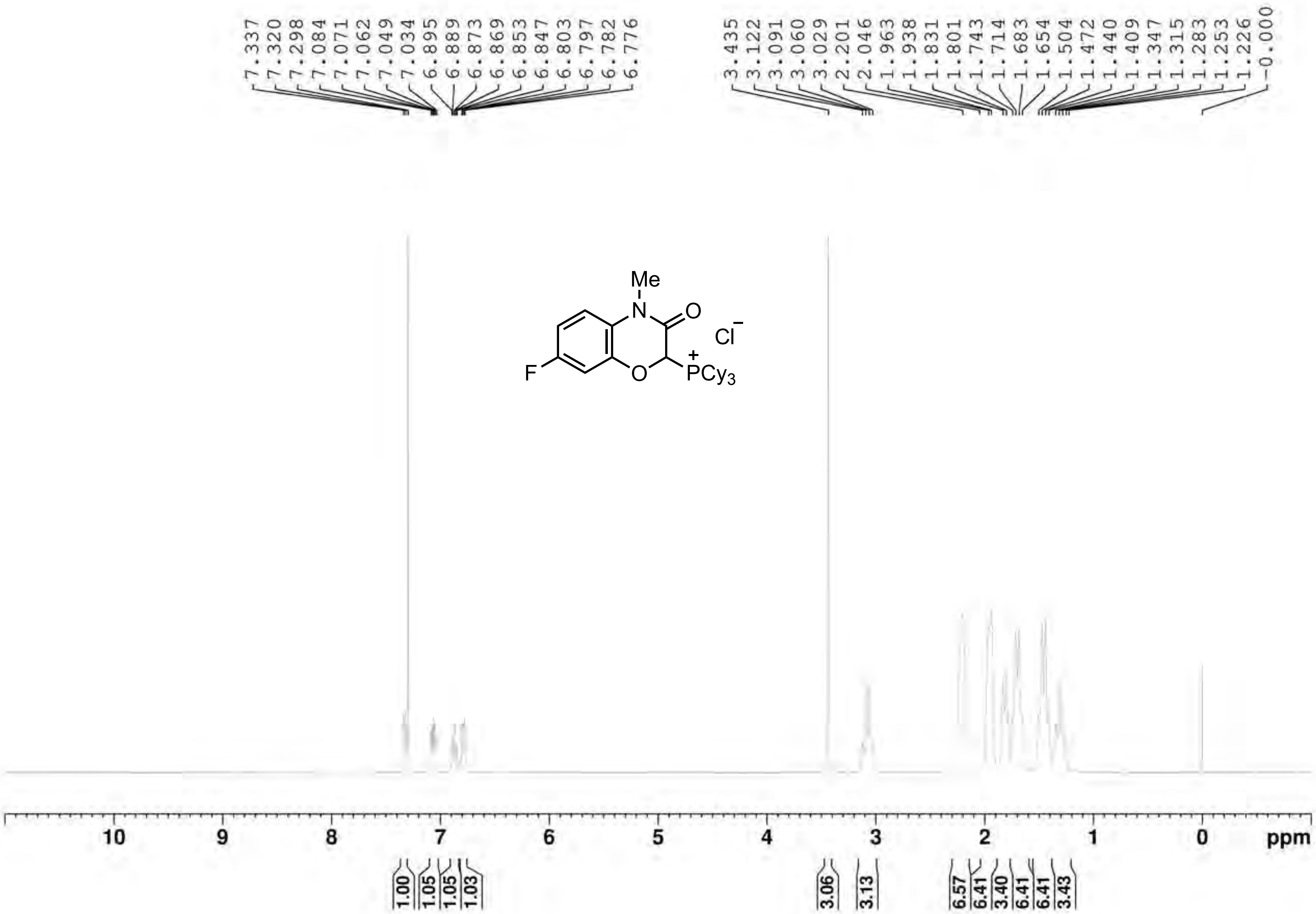

^{1}H NMR (400 MHz, $CDCl_3$) spectrum of **2e**

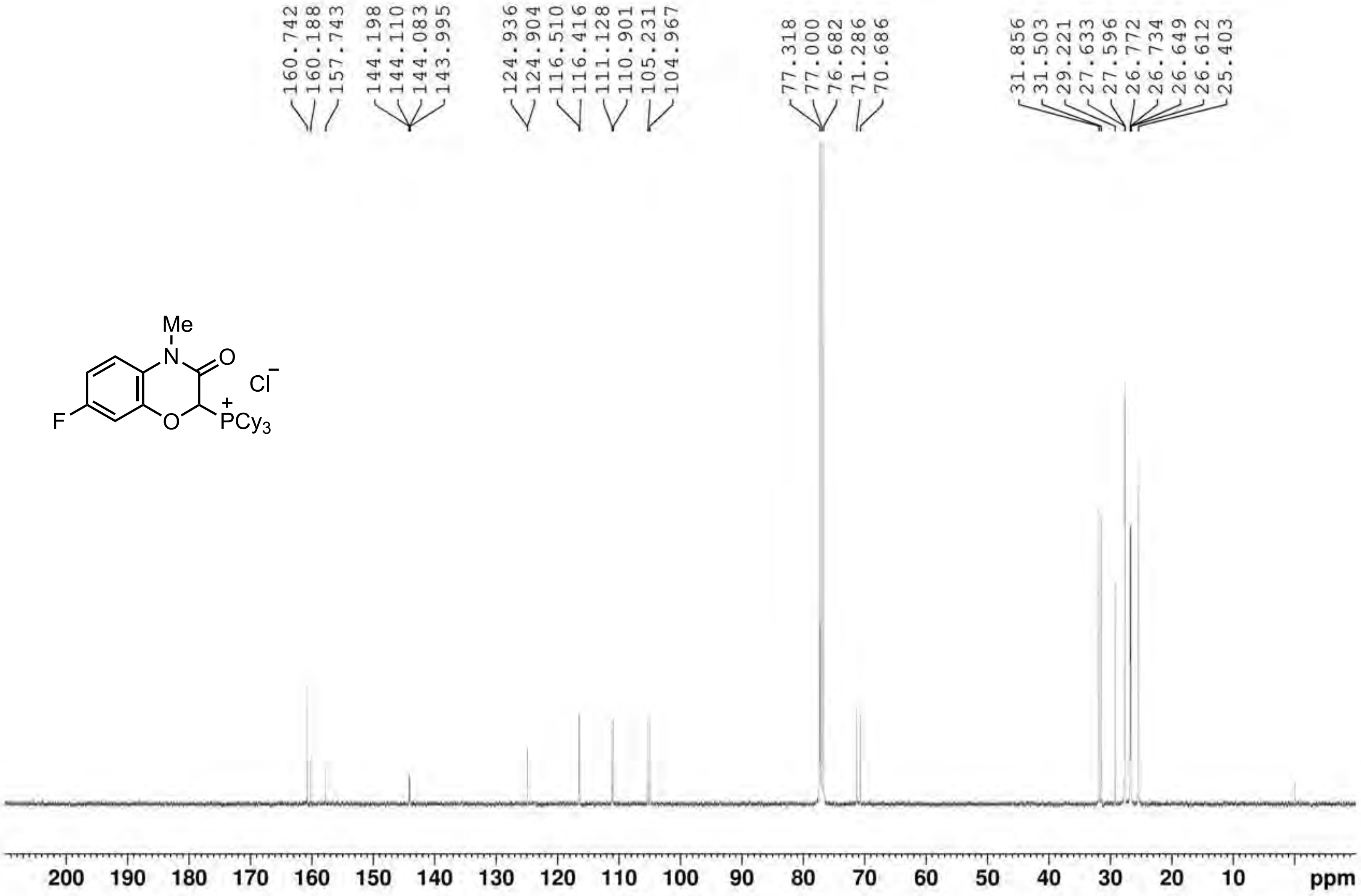

^{13}C NMR (100.6 MHz, $CDCl_3$) spectrum of **2e**

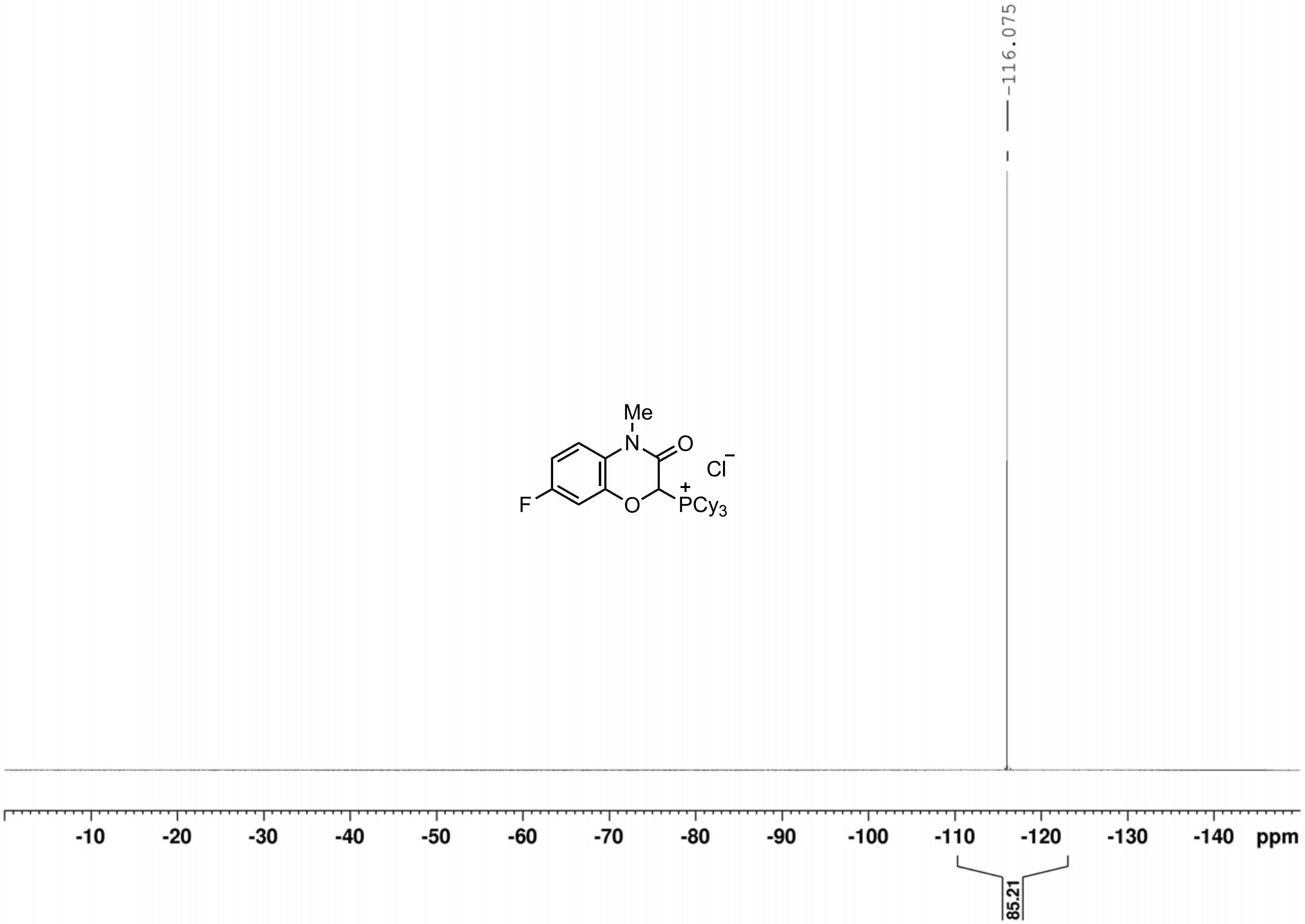

^{19}F NMR (376.5 MHz, $CDCl_3$) spectrum of **2e**

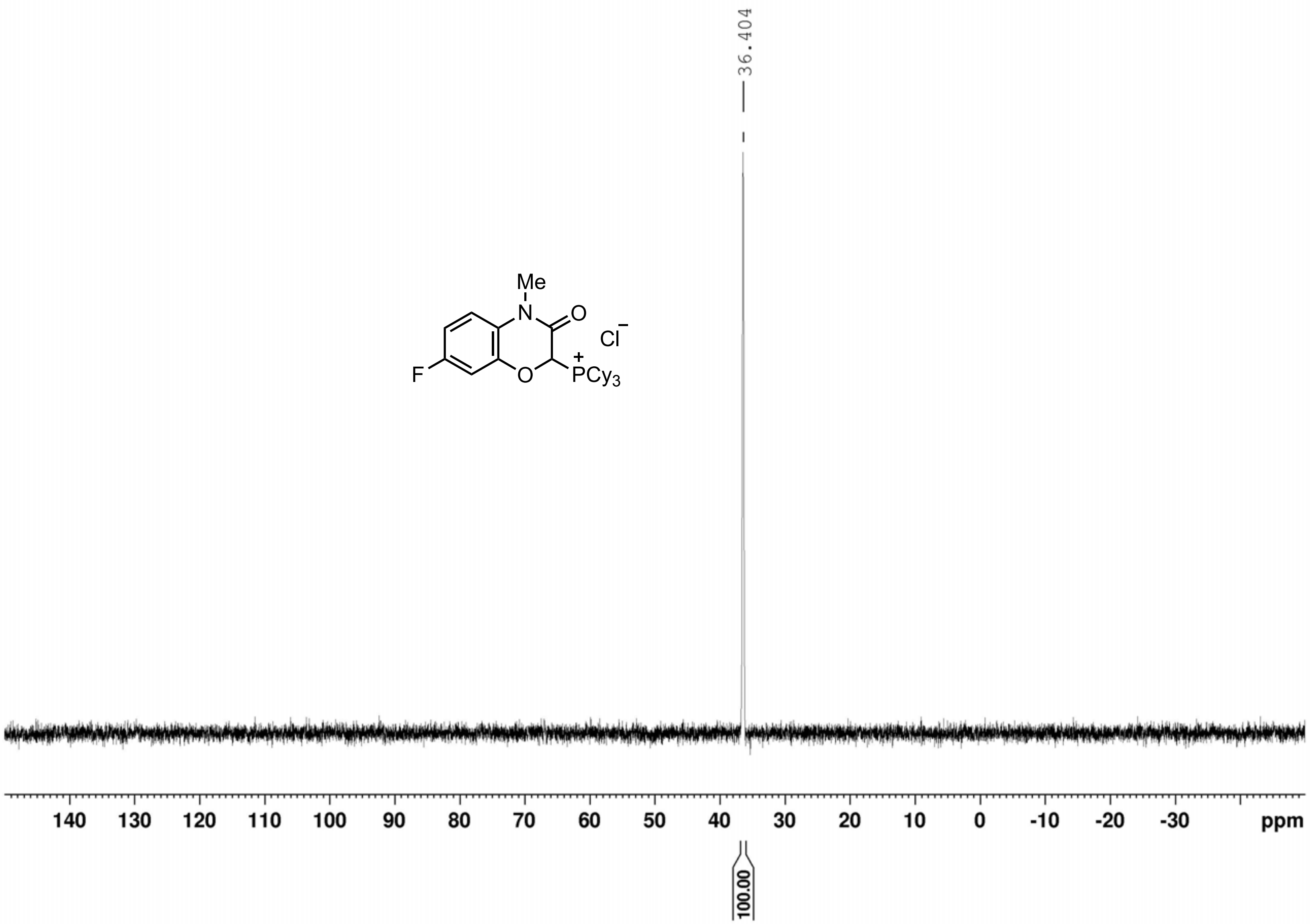

^{31}P NMR (162 MHz, $CDCl_3$) spectrum of **2e**

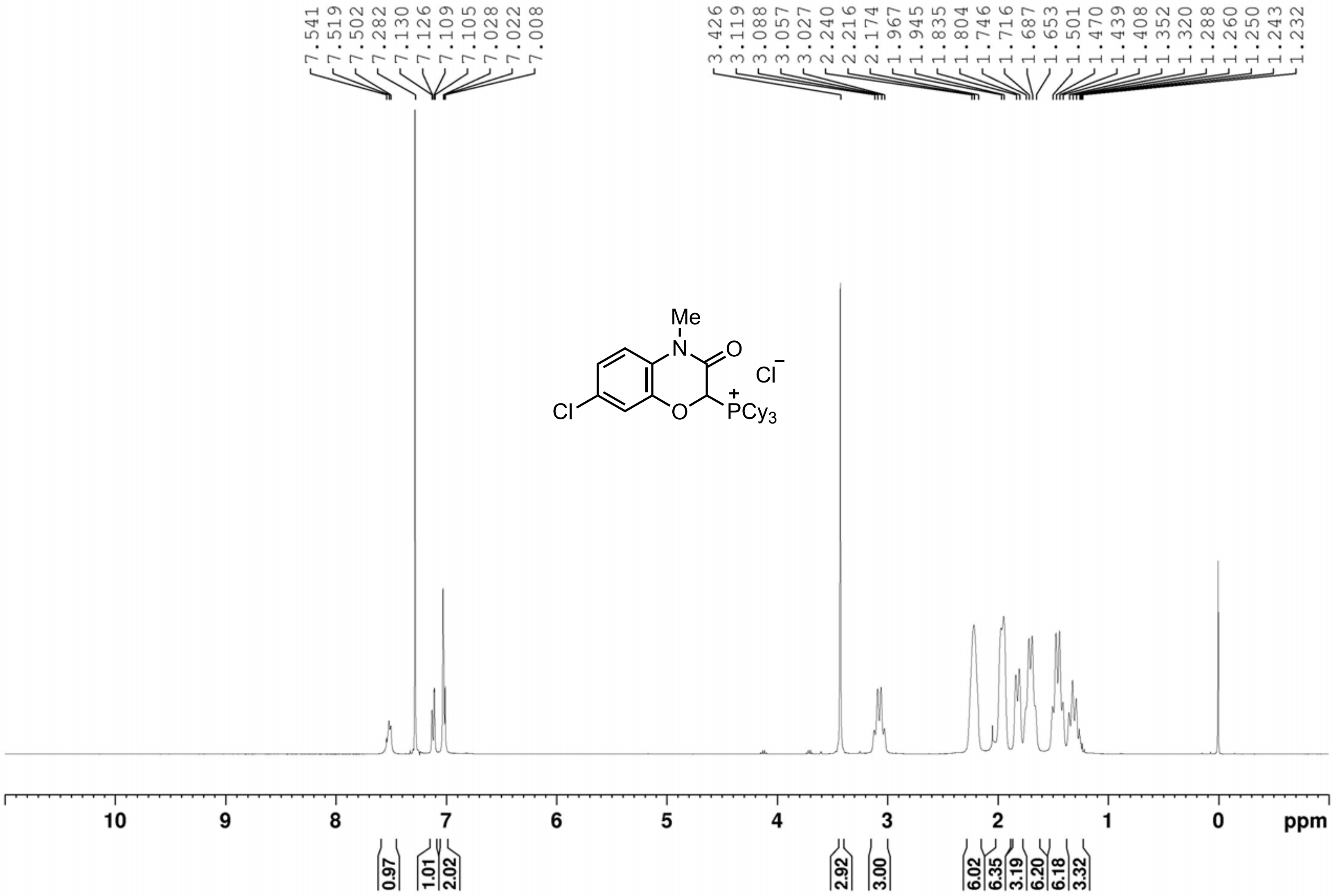

^{1}H NMR (400 MHz, $CDCl_3$) spectrum of **2f**

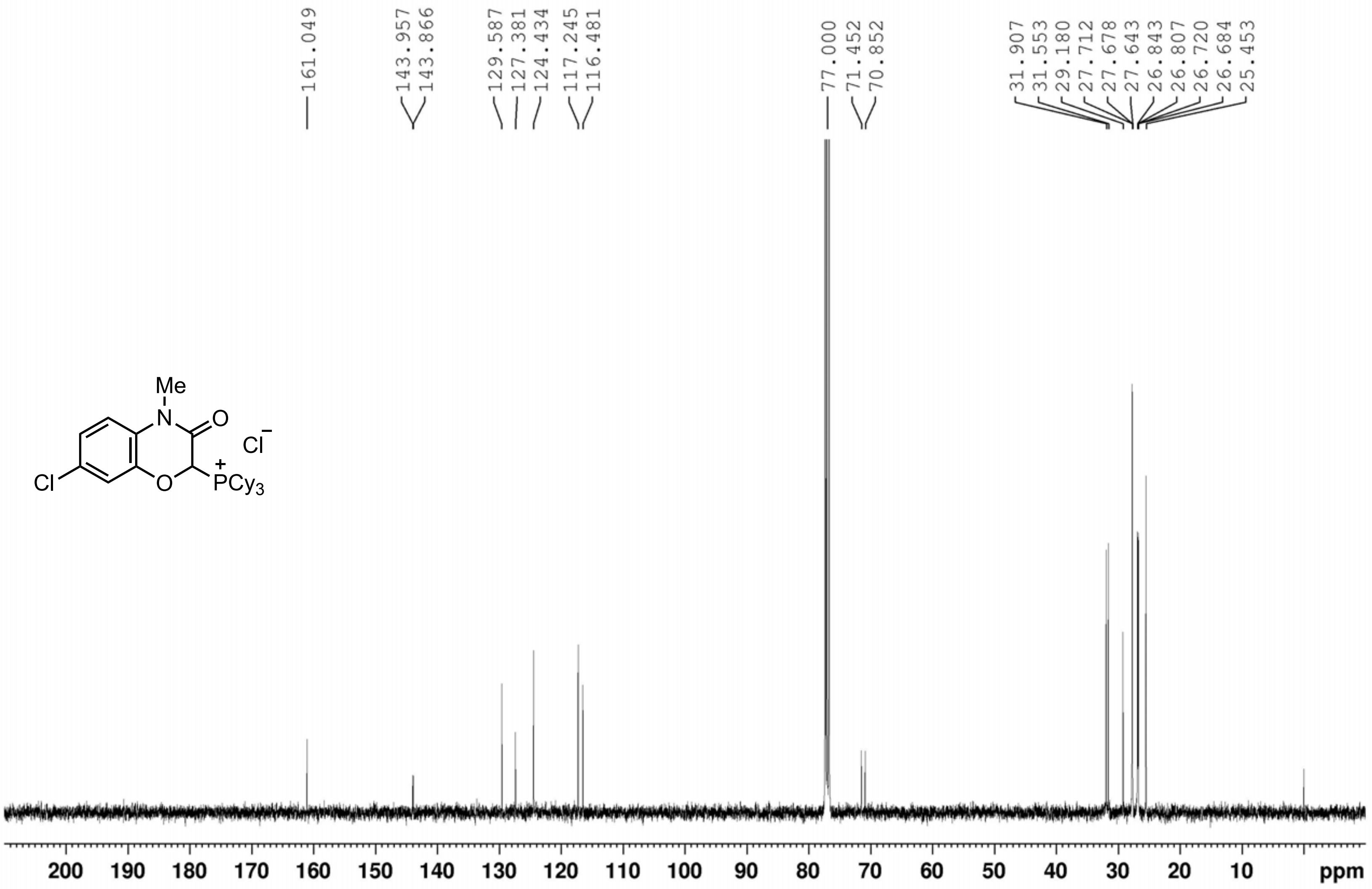

^{13}C NMR (100.6 MHz, $CDCl_3$) spectrum of **2f**

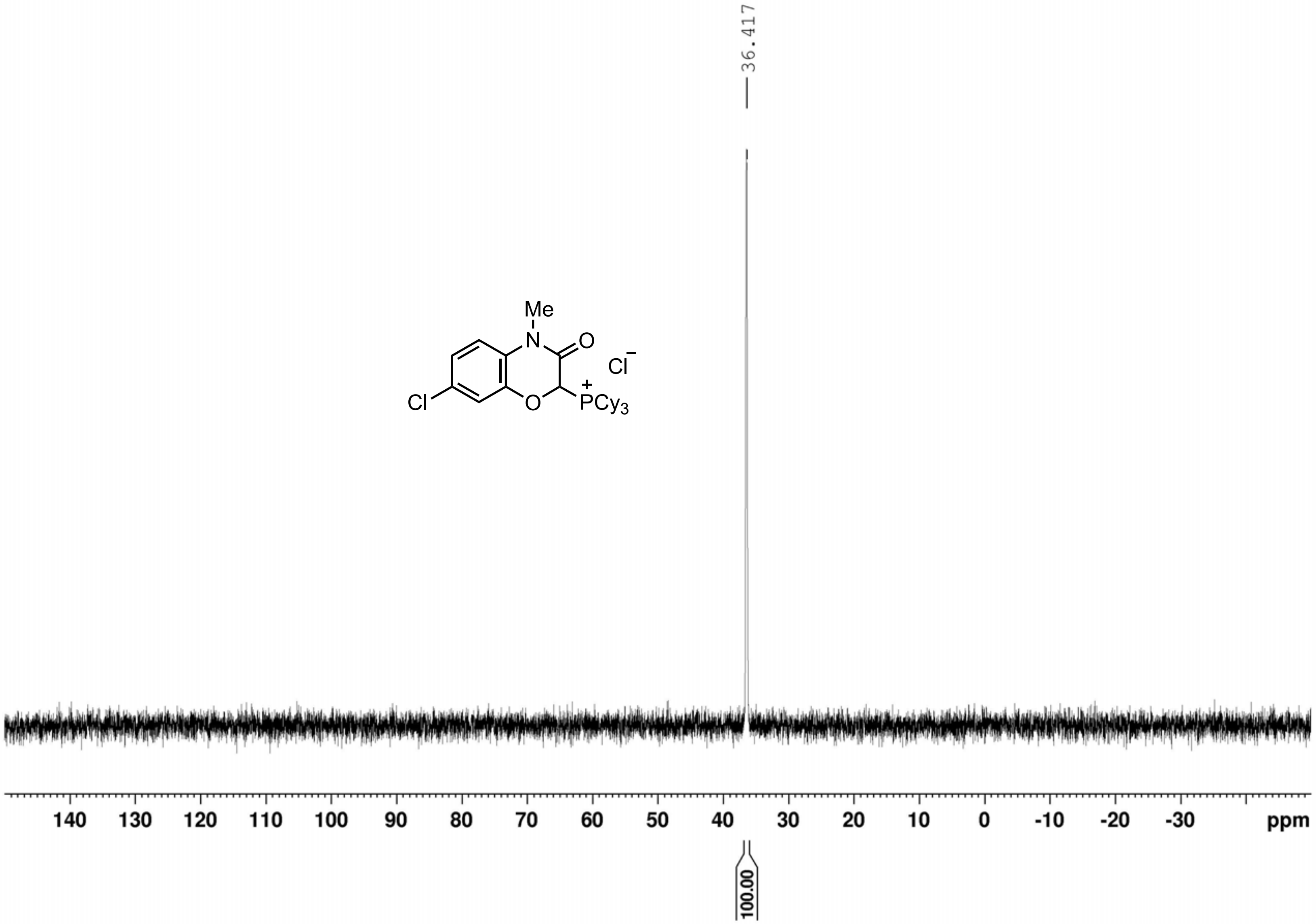

^{31}P NMR (162 MHz, $CDCl_3$) spectrum of **2f**

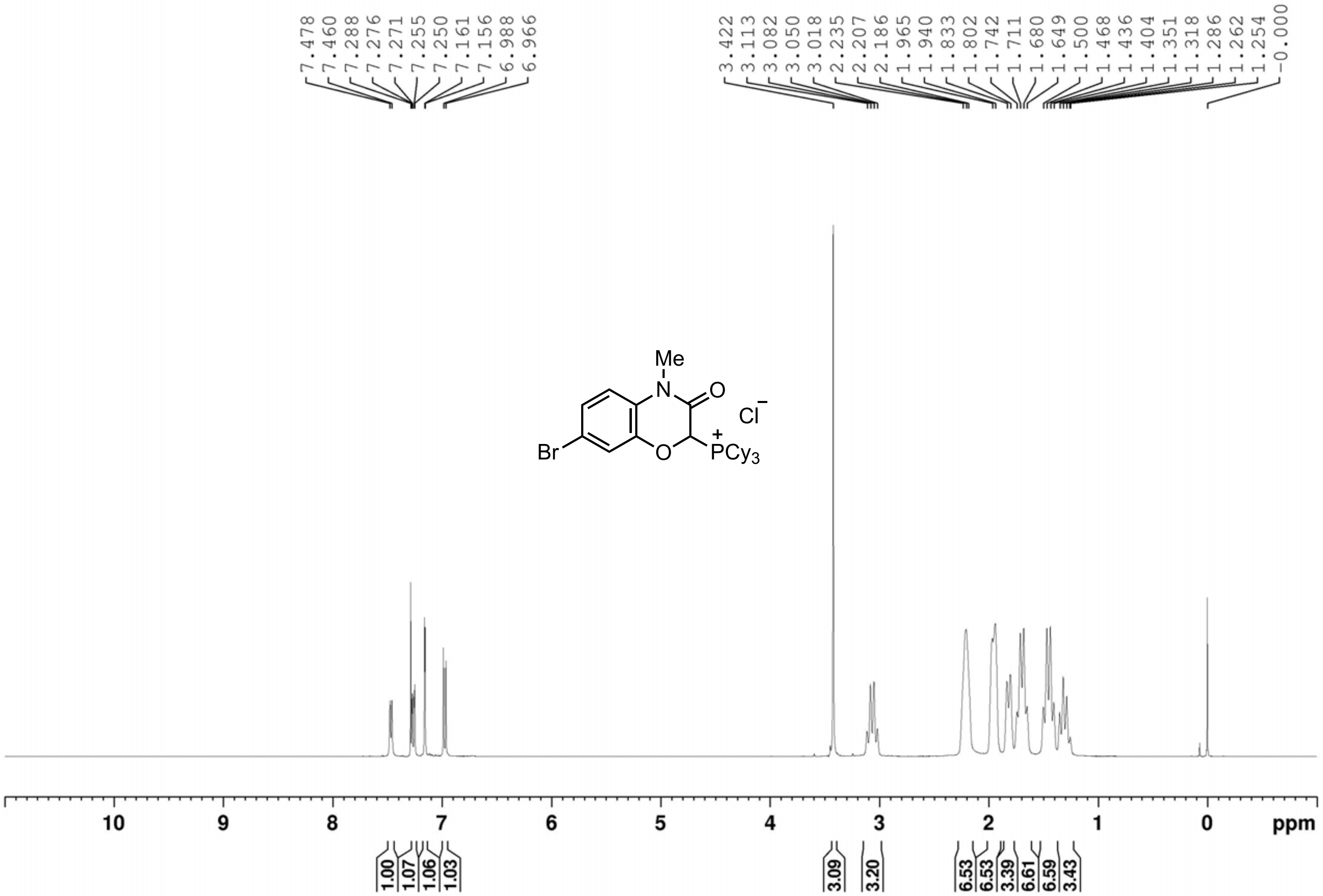

^{1}H NMR (400 MHz, $CDCl_3$) spectrum of **2g**

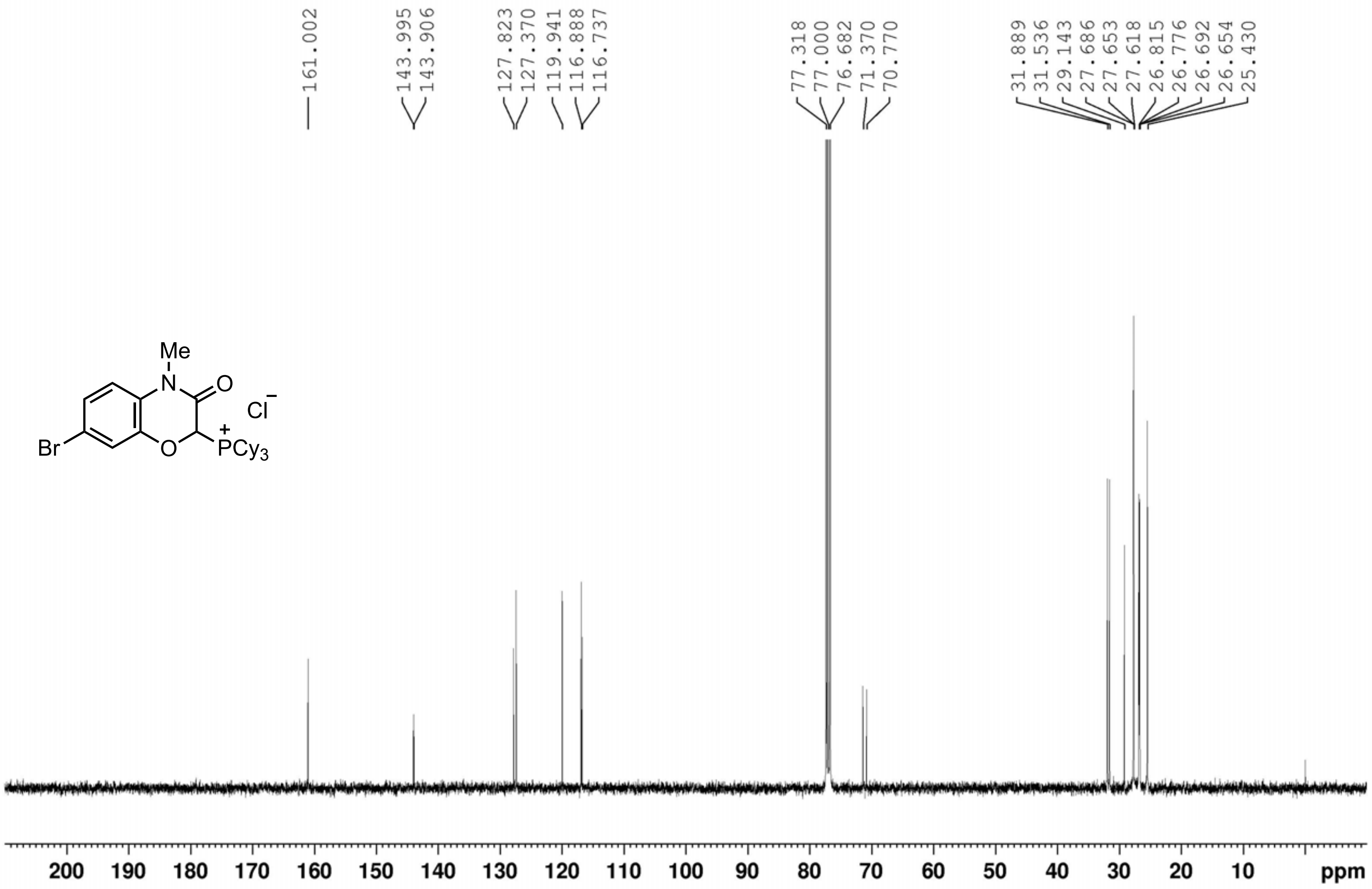

^{13}C NMR (100.6 MHz, $CDCl_3$) spectrum of **2g**

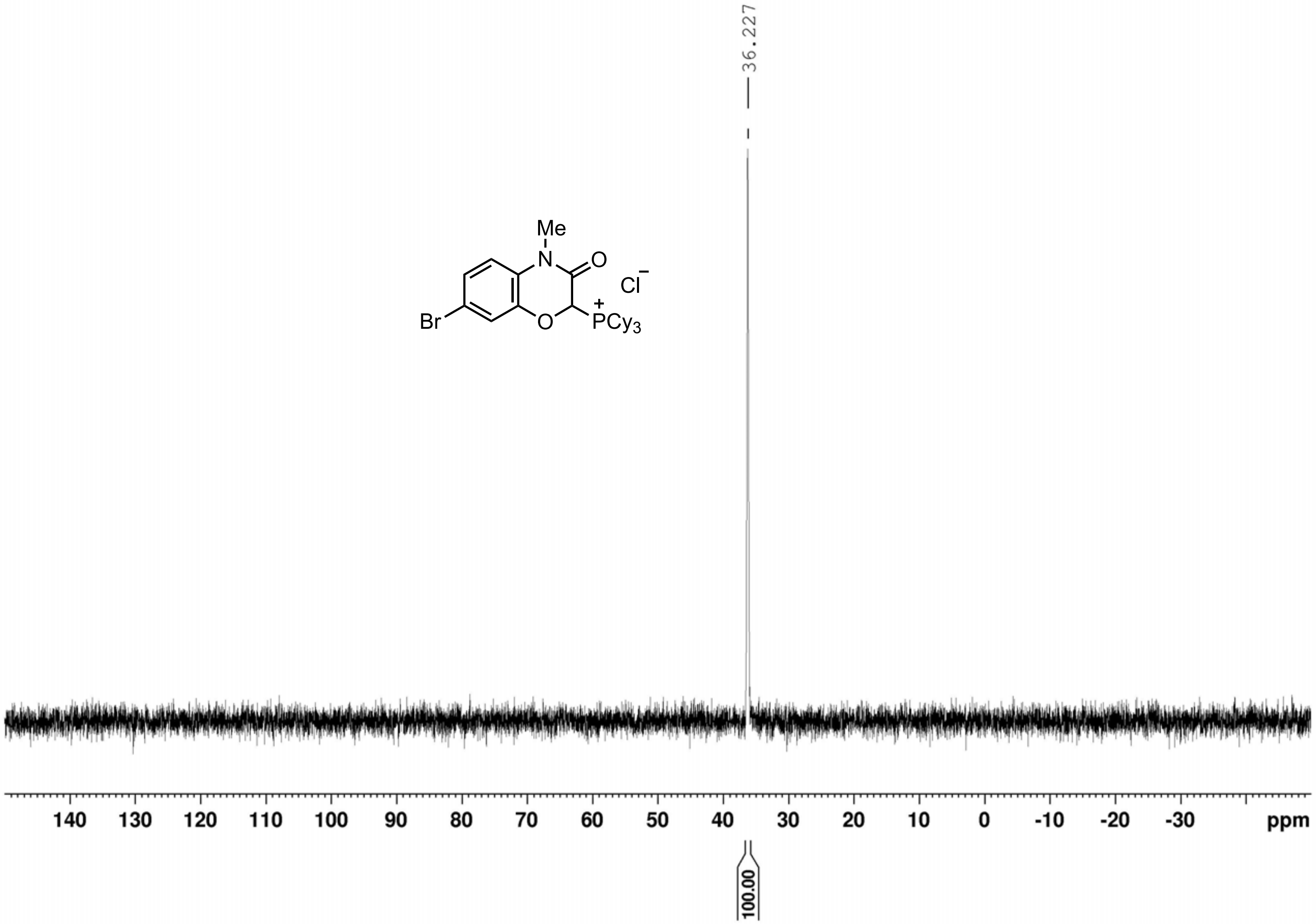

^{31}P NMR (162 MHz, $CDCl_3$) spectrum of **2g**

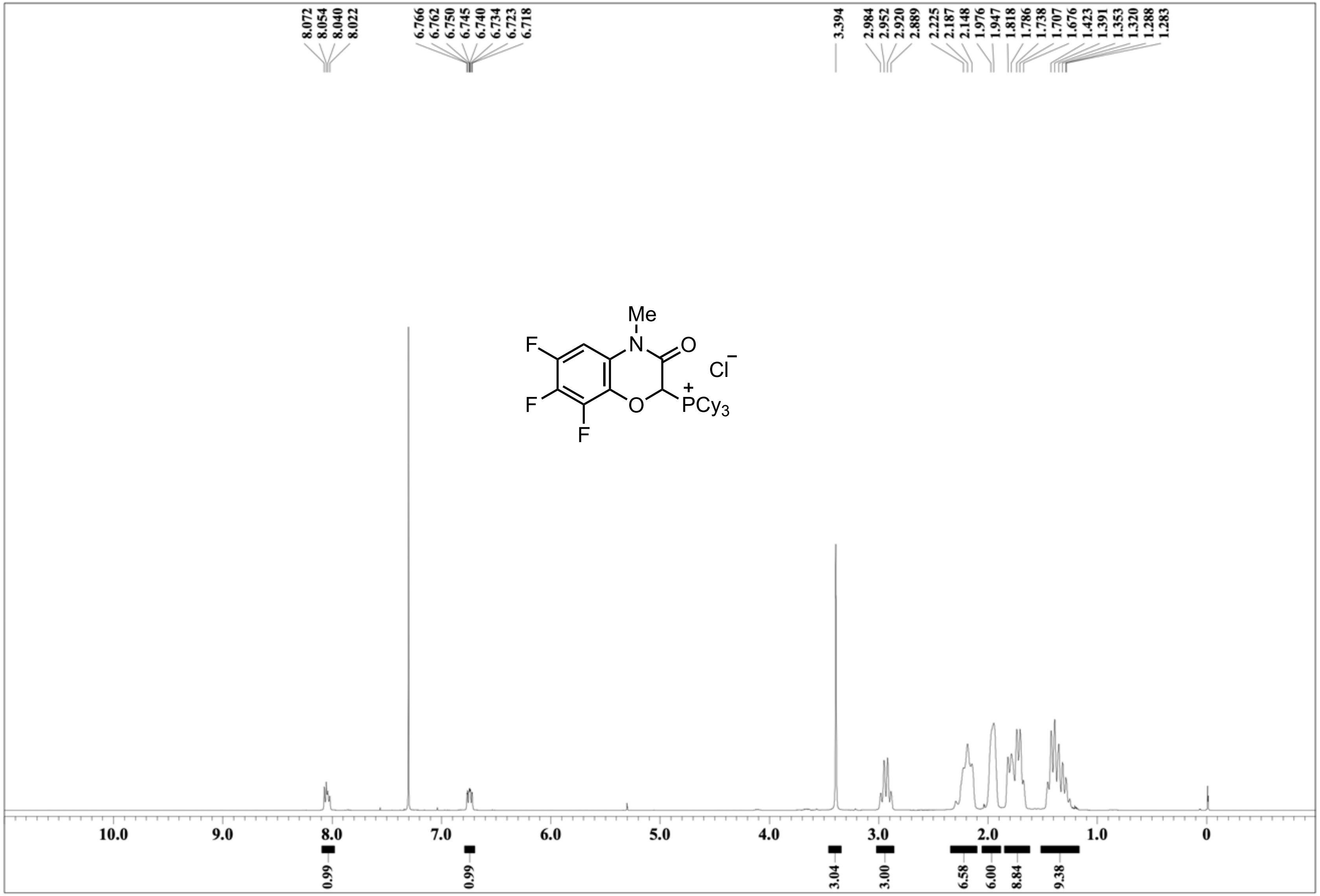

^{1}H NMR (400 MHz, $CDCl_3$) spectrum of **2h**

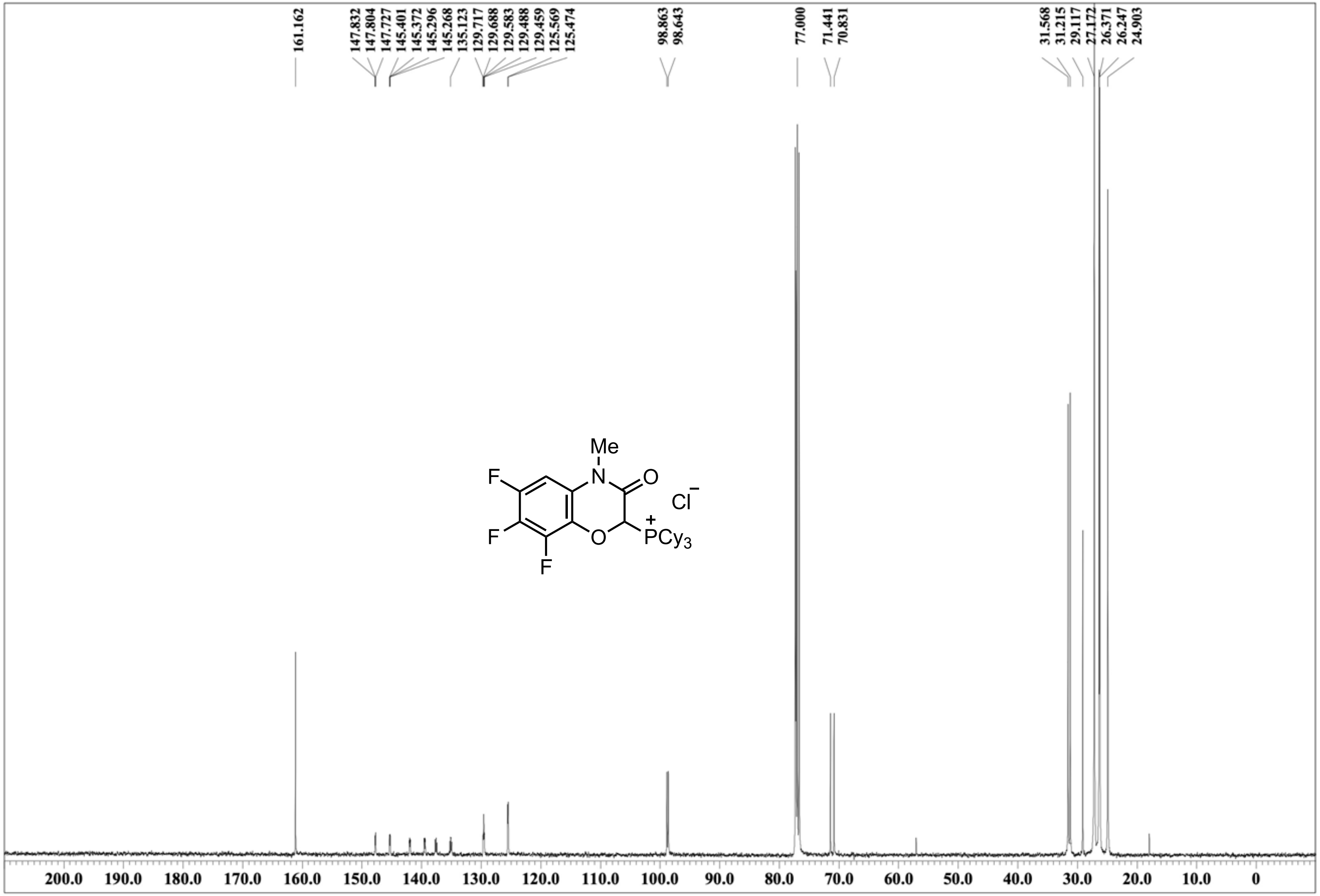

^{13}C NMR (100.6 MHz, $CDCl_3$) spectrum of **2h**

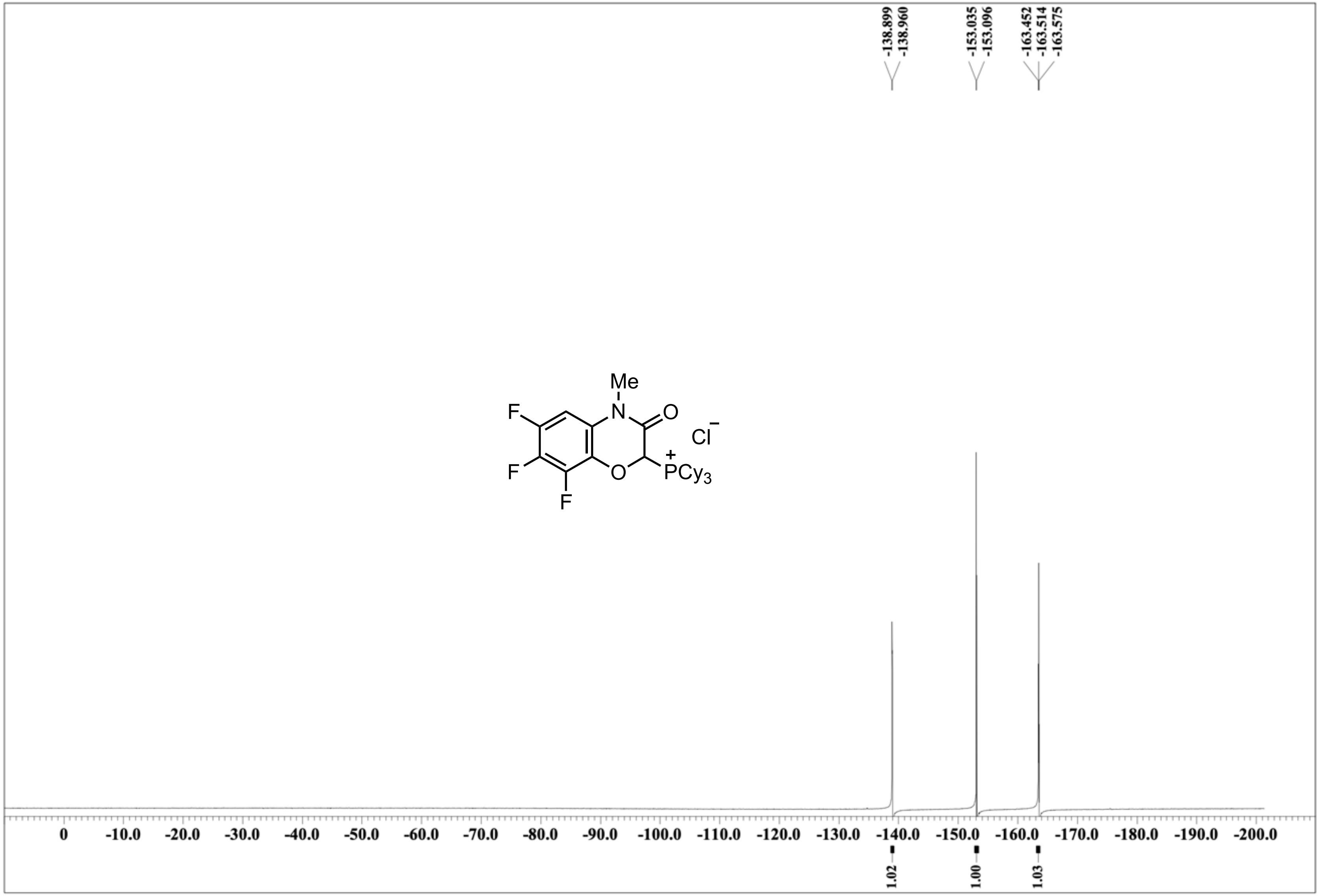

^{19}F NMR (376.5 MHz, $CDCl_3$) spectrum of **2h**

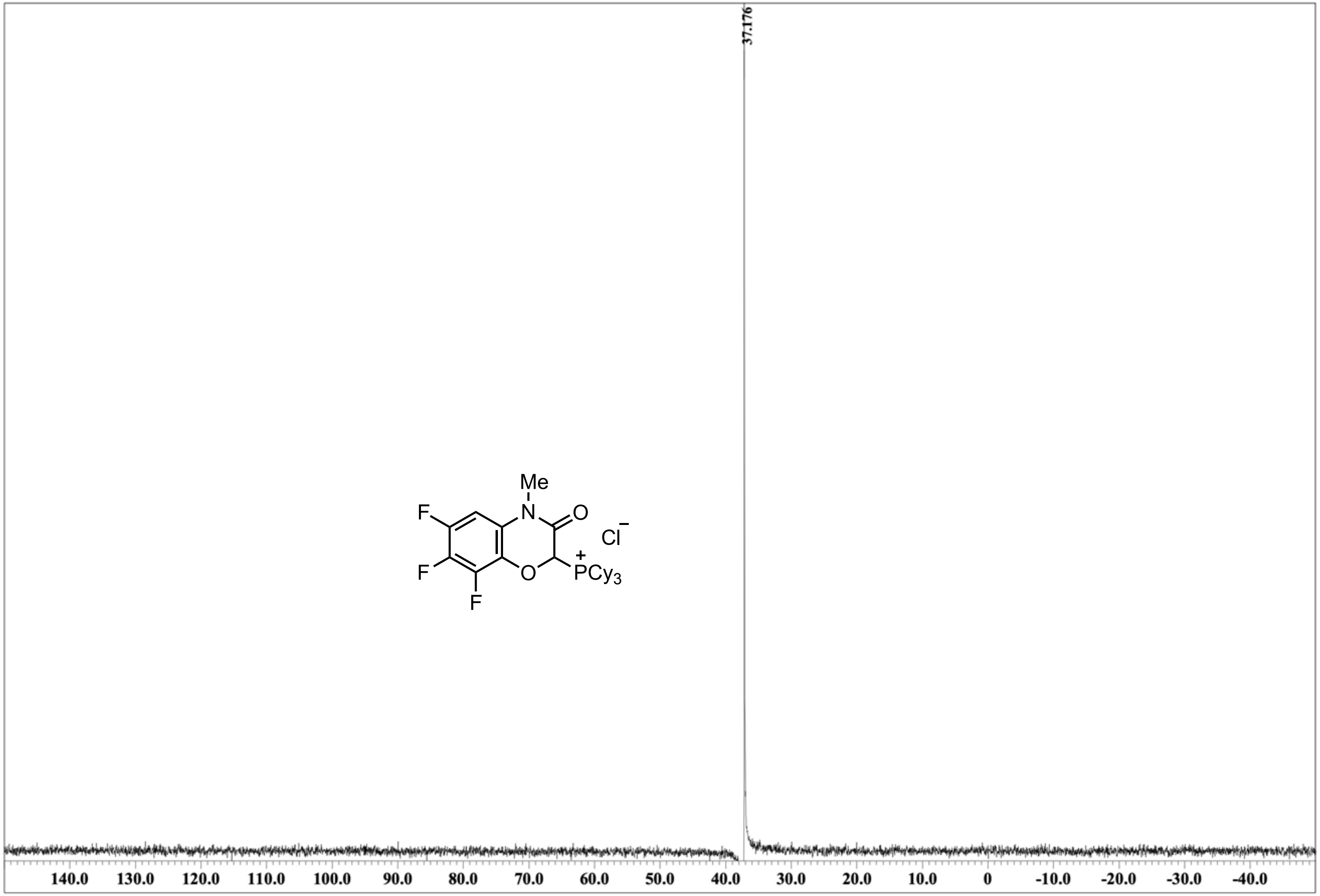

^{31}P NMR (162 MHz, $CDCl_3$) spectrum of **2h**

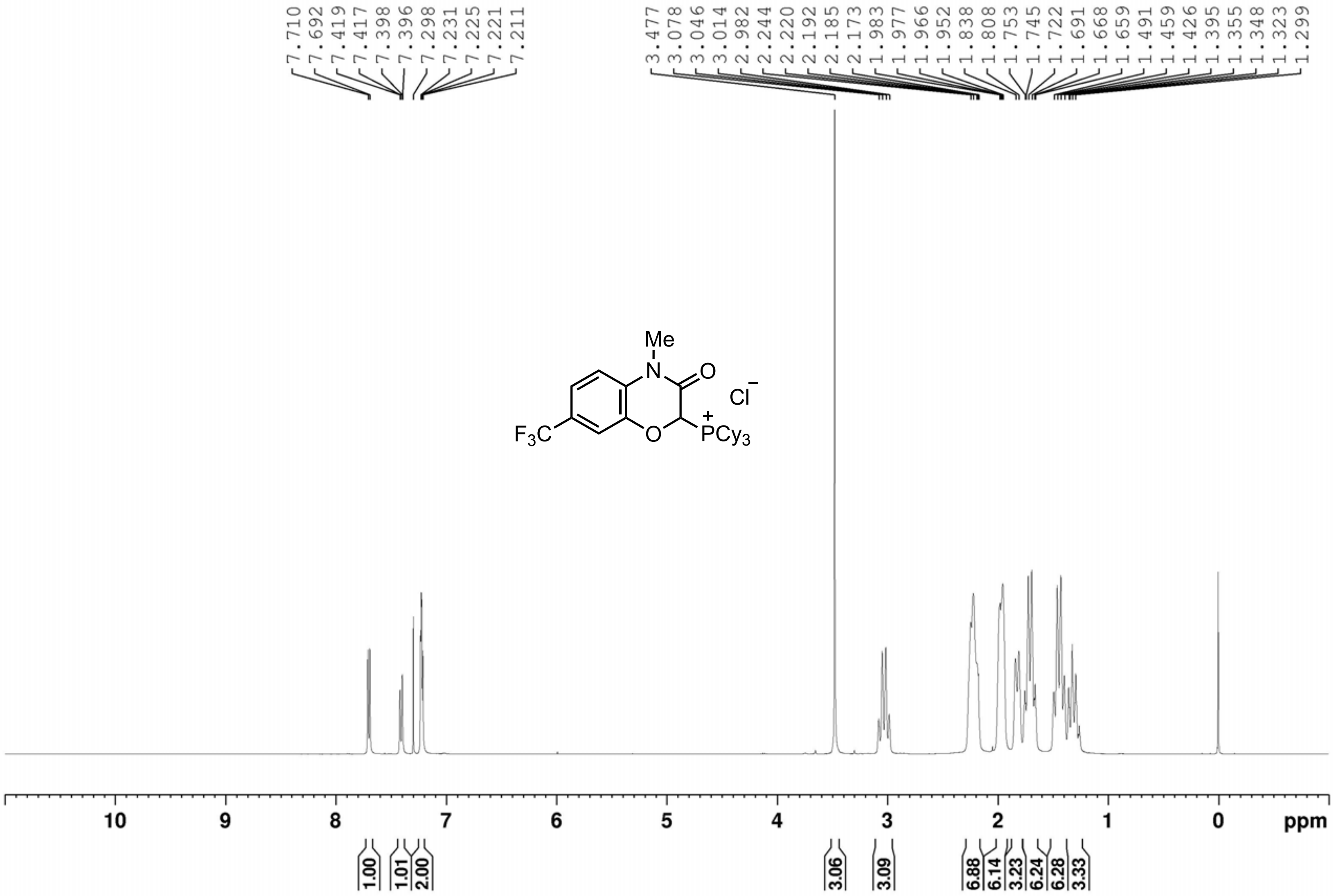

^{1}H NMR (400 MHz, $CDCl_3$) spectrum of **2i**

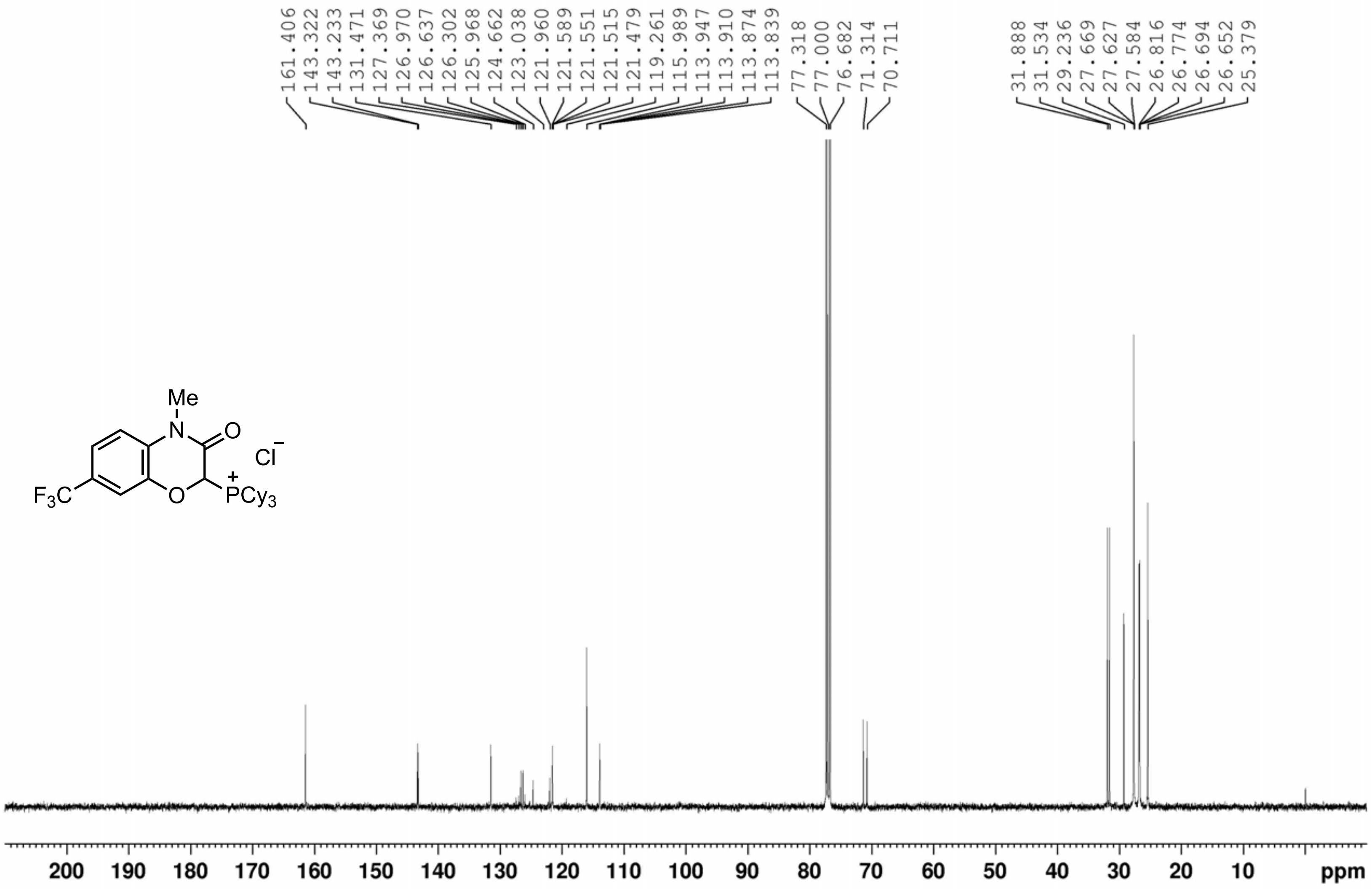

^{13}C NMR (100.6 MHz, $CDCl_3$) spectrum of **2i**

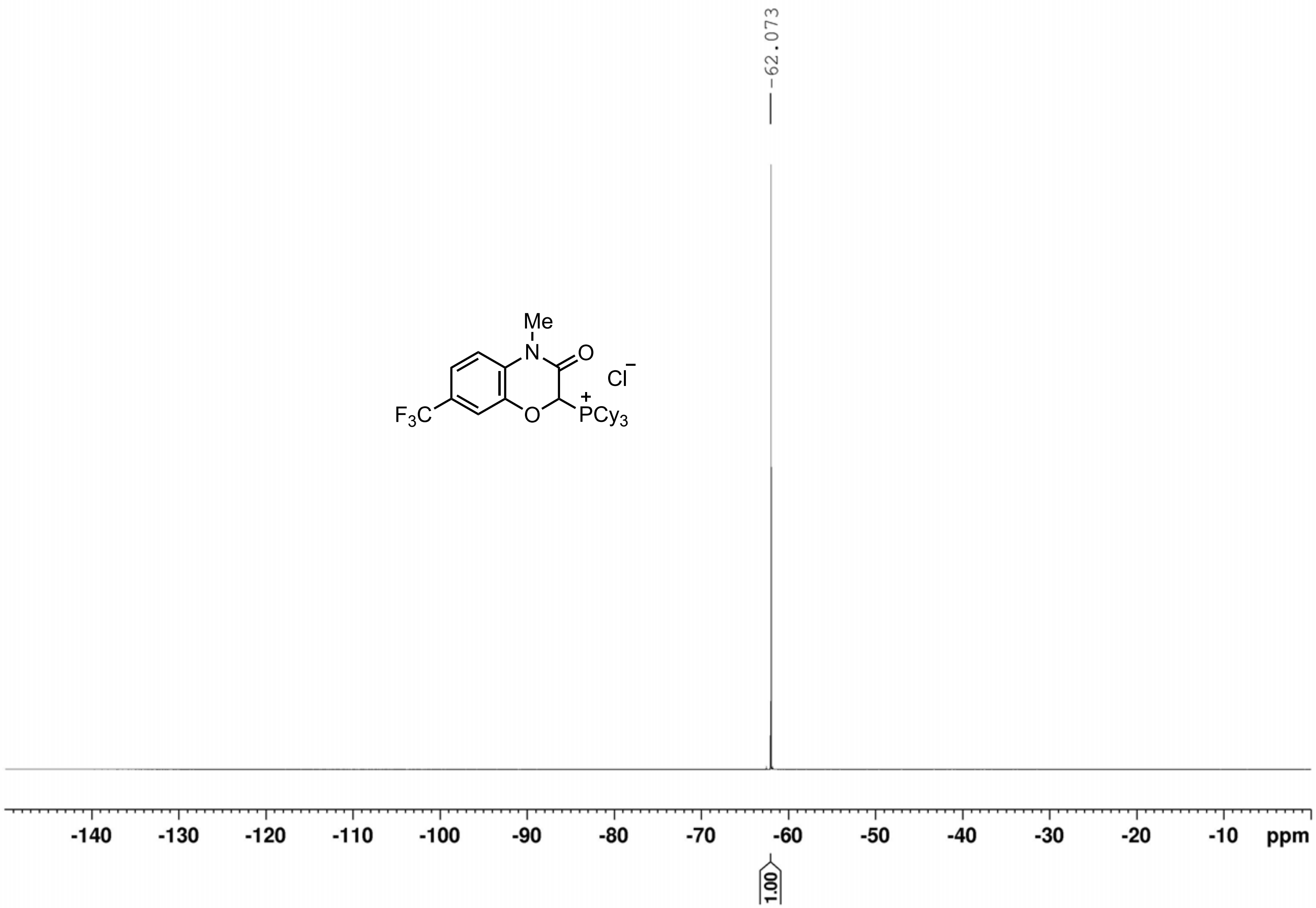

^{19}F NMR (376.5 MHz, $CDCl_3$) spectrum of **2i**

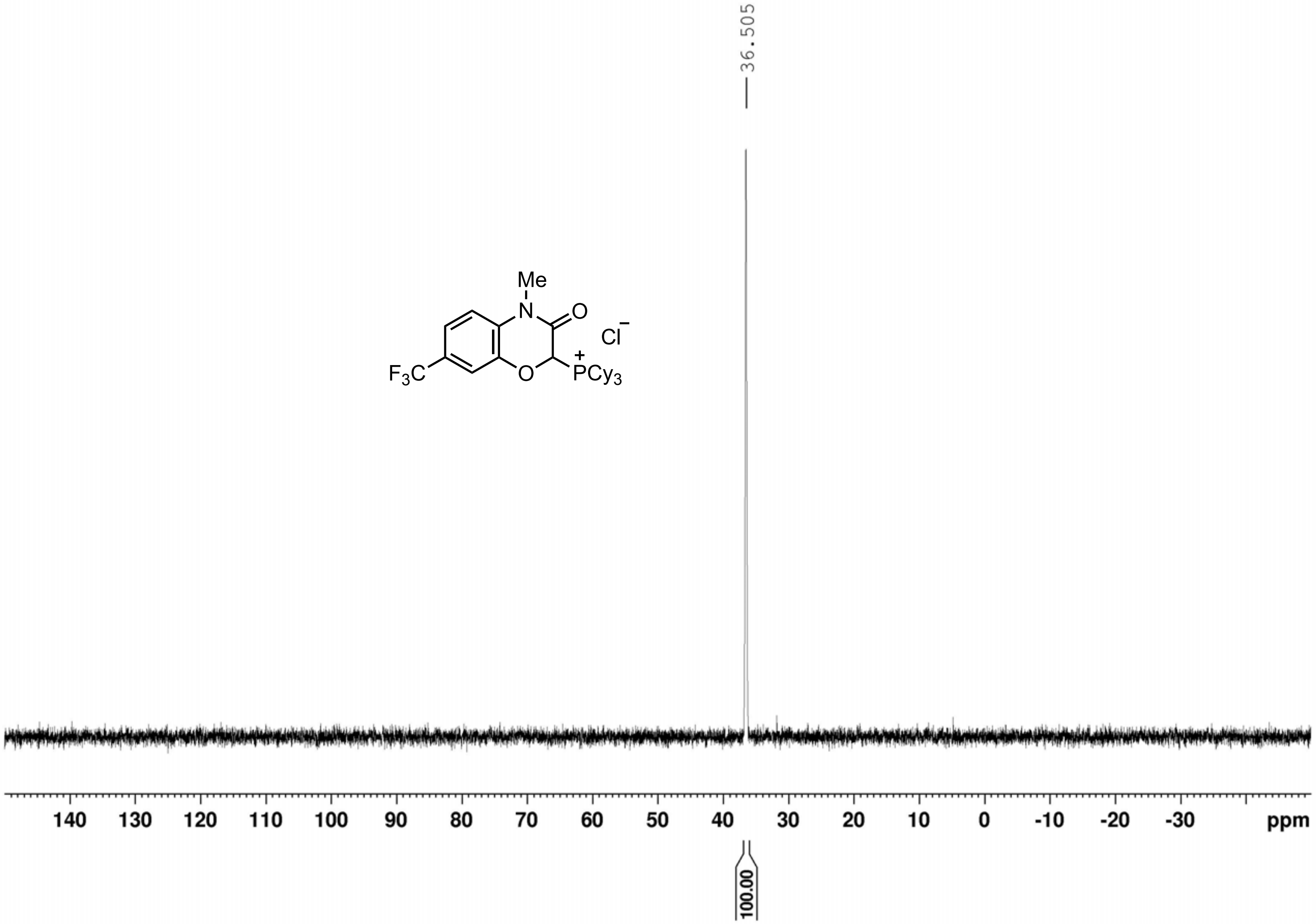

^{31}P NMR (162 MHz, $CDCl_3$) spectrum of **2i**

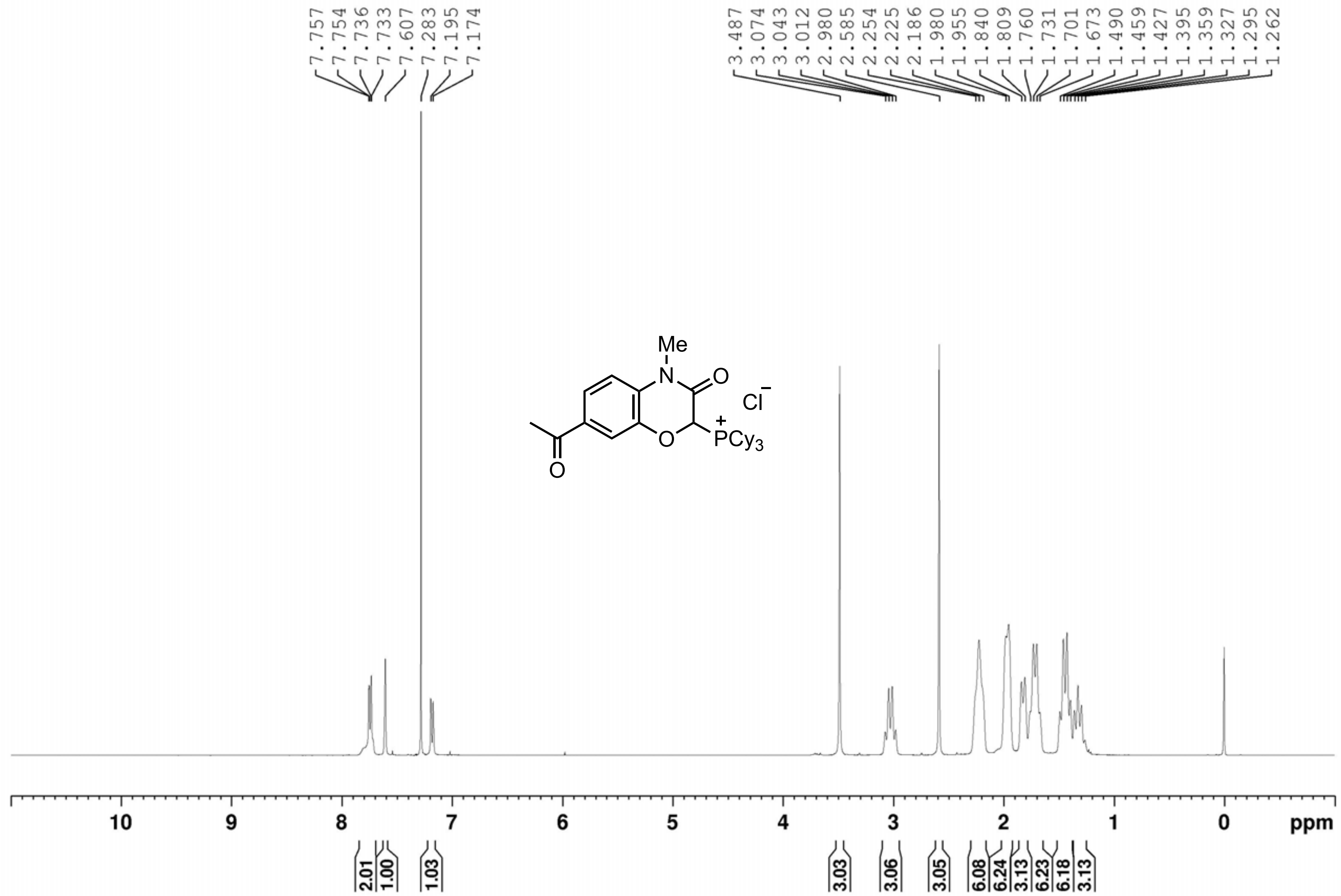

^{1}H NMR (400 MHz, $CDCl_3$) spectrum of **2j**

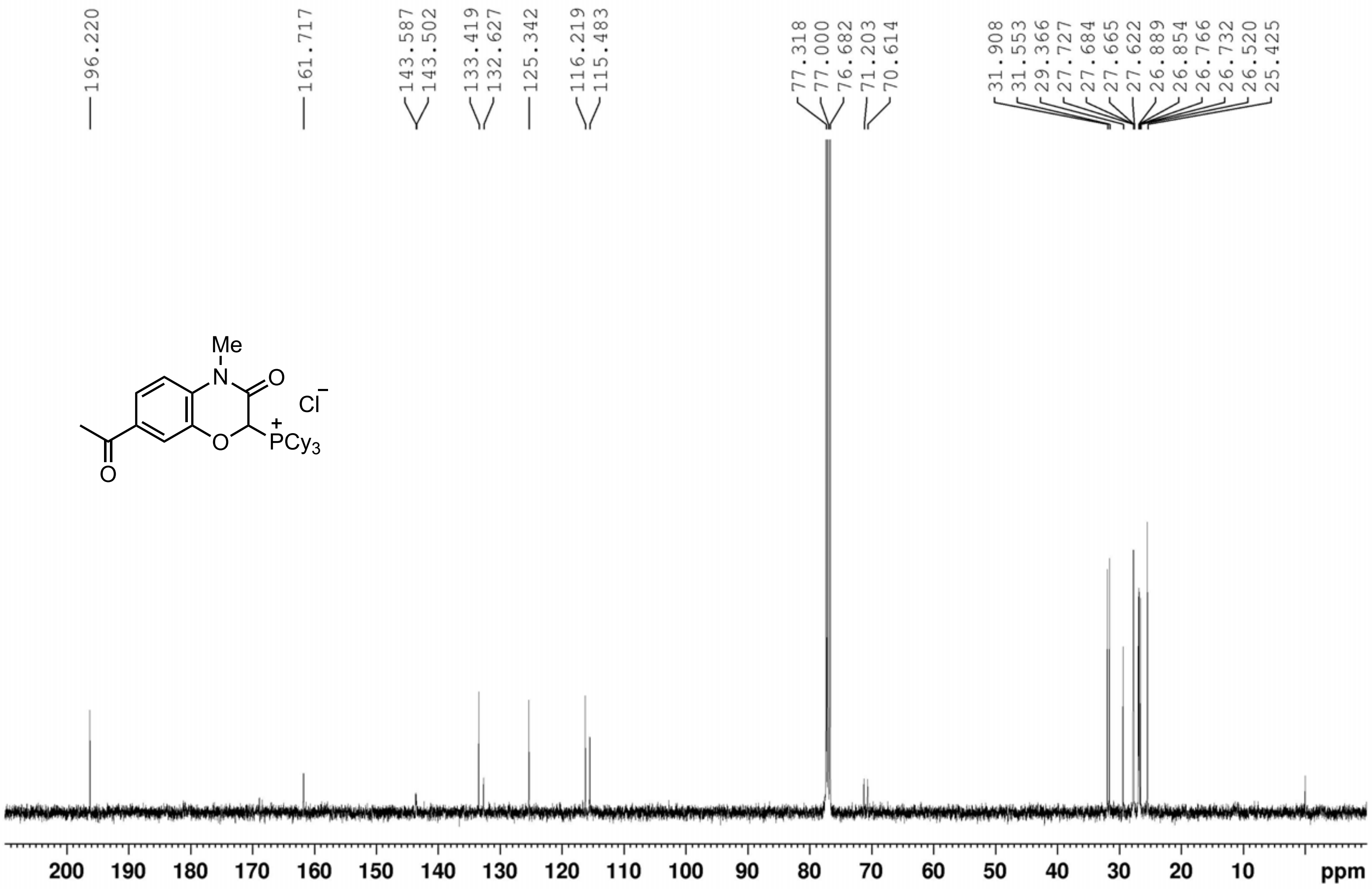

^{13}C NMR (100.6 MHz, $CDCl_3$) spectrum of **2j**

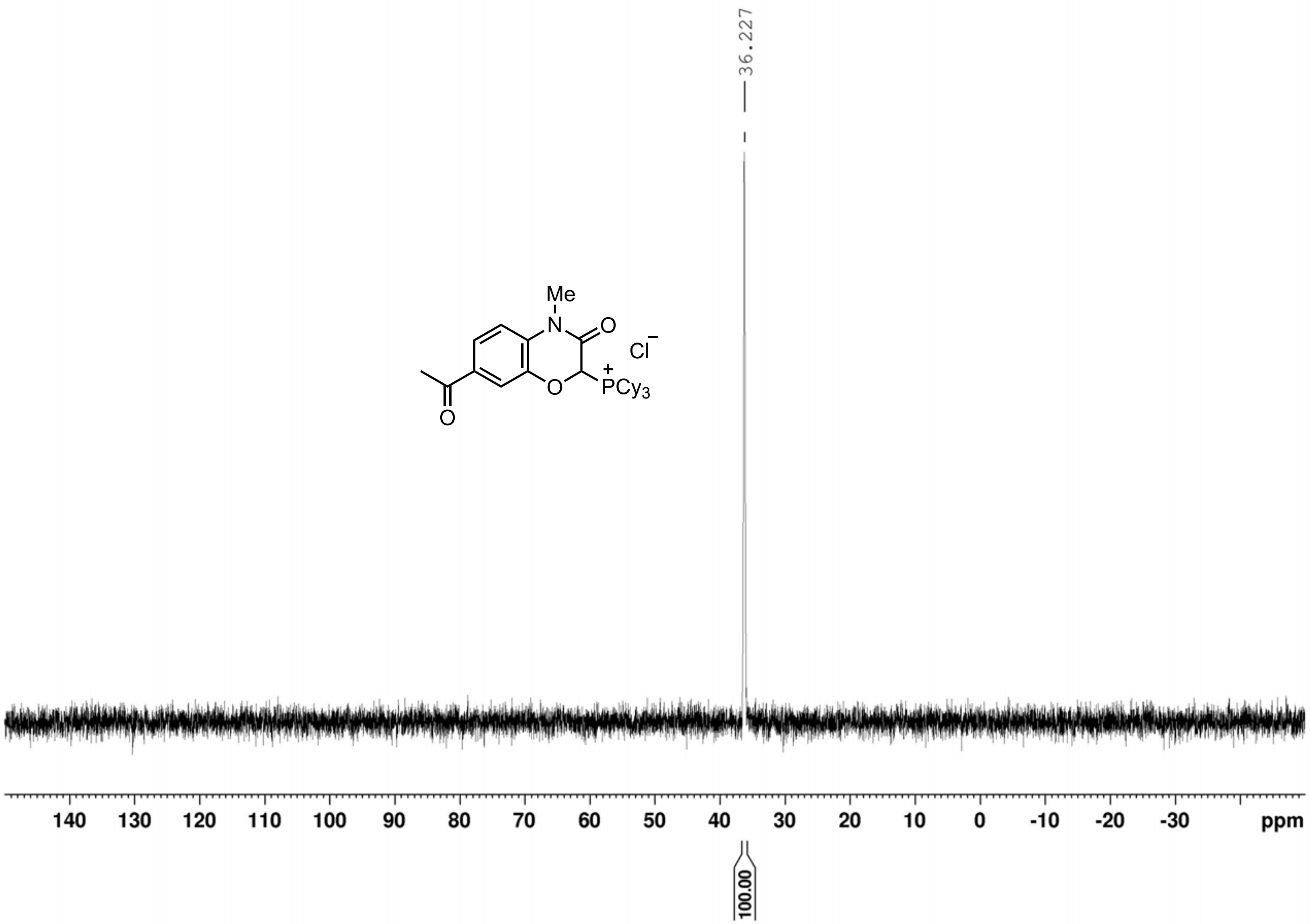

^{31}P NMR (162 MHz, $CDCl_3$) spectrum of **2j**

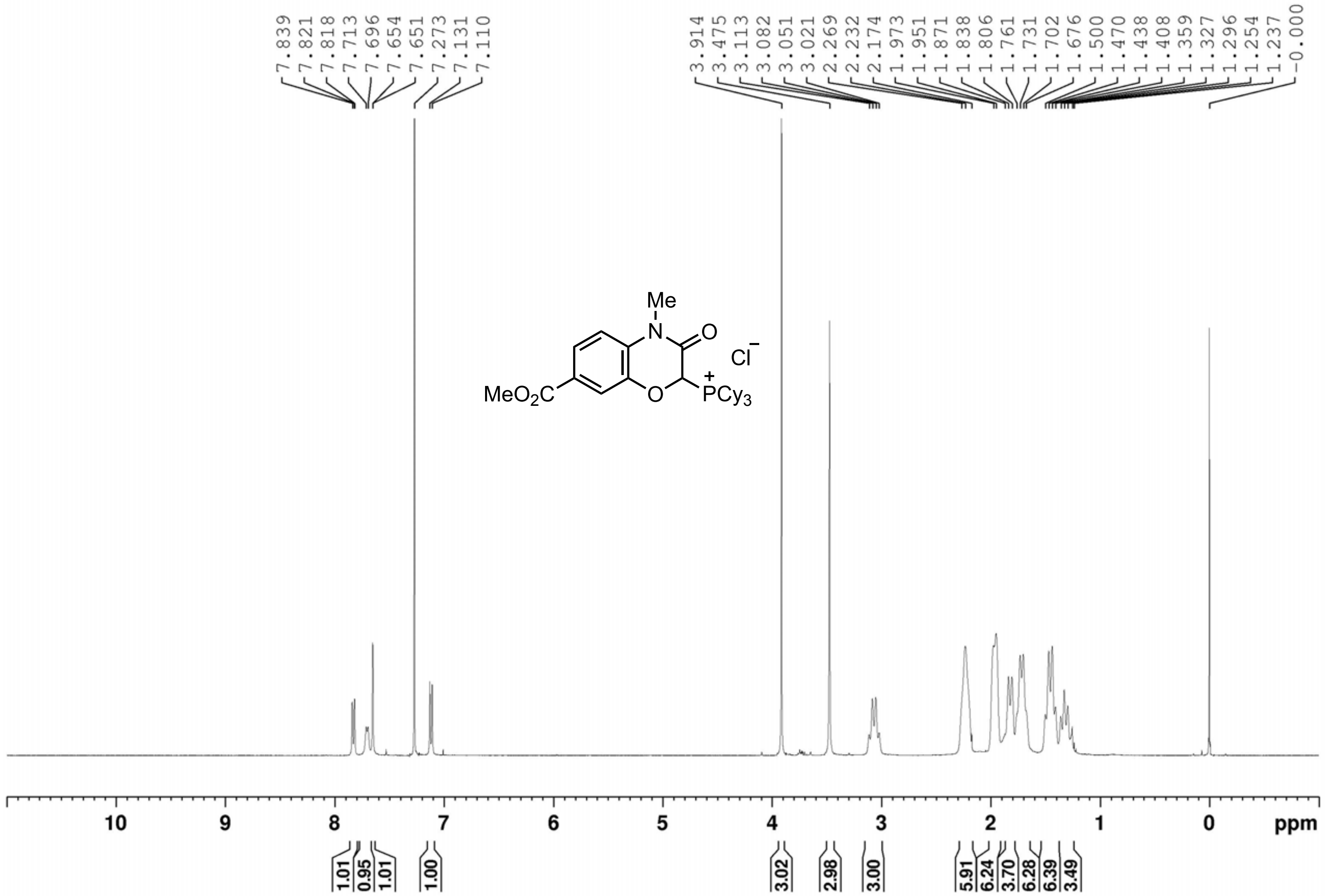

^{1}H NMR (400 MHz, $CDCl_3$) spectrum of **2k**

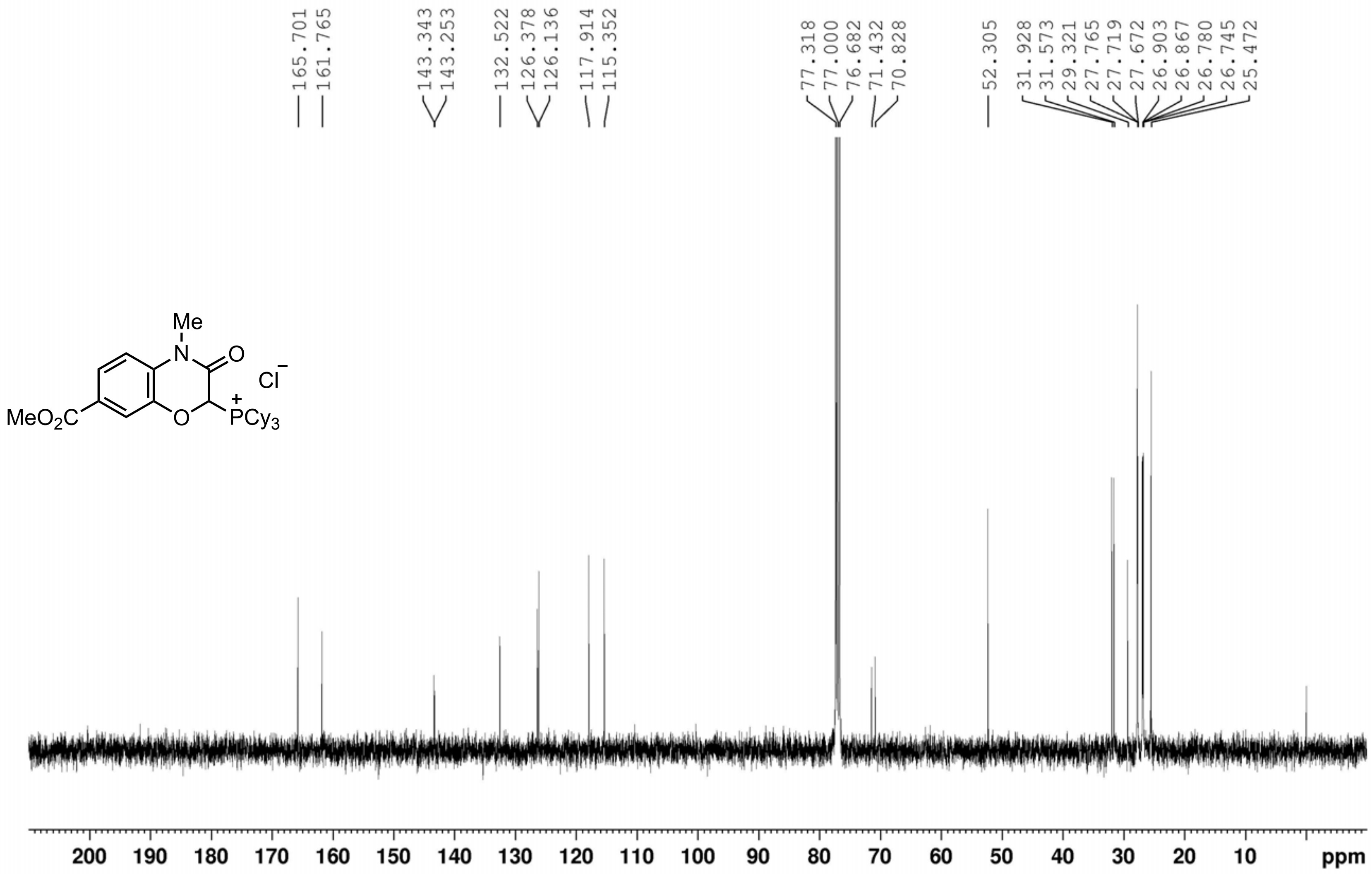

^{13}C NMR (100.6 MHz, $CDCl_3$) spectrum of **2k**

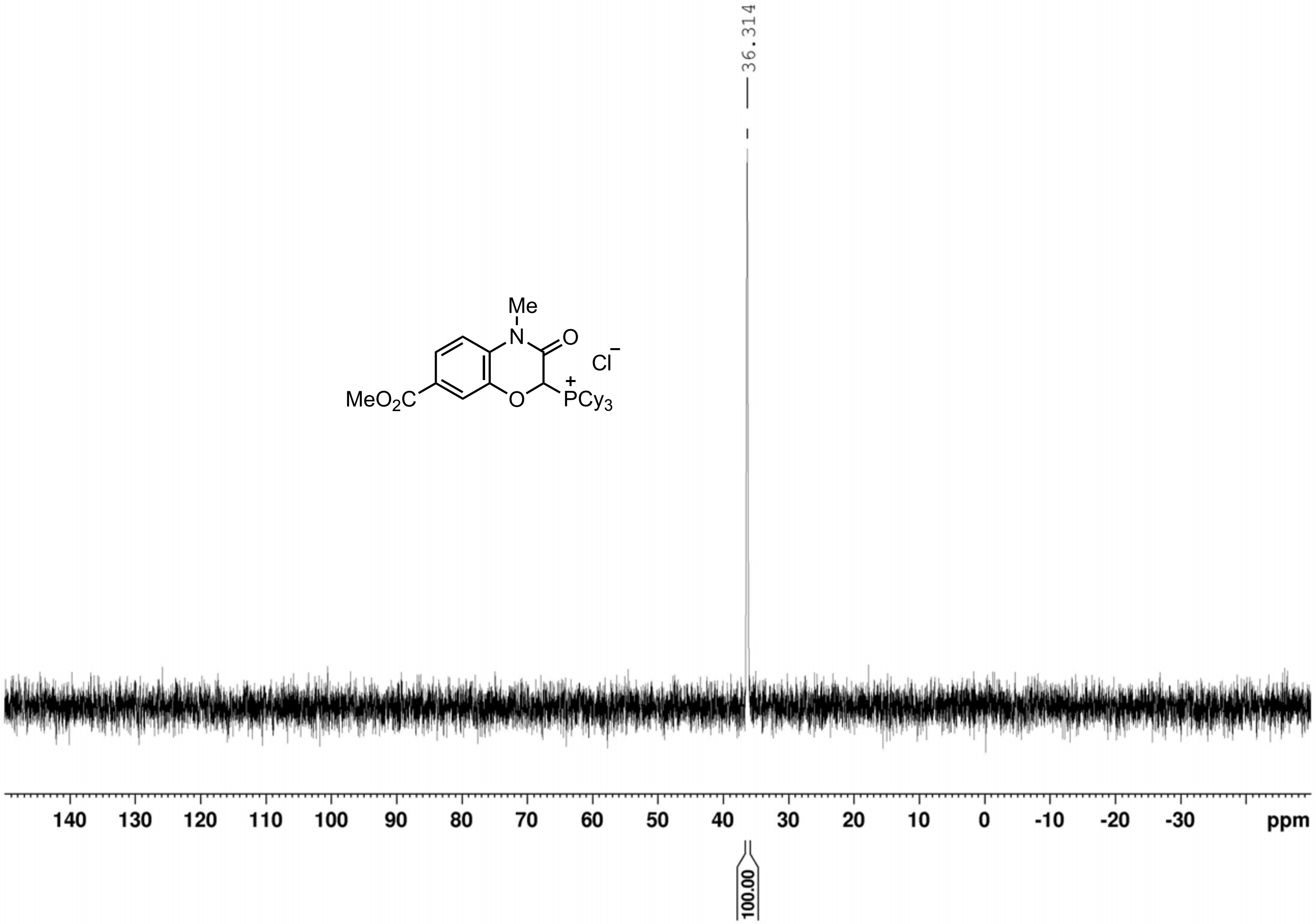

^{31}P NMR (162 MHz, $CDCl_3$) spectrum of **2k**

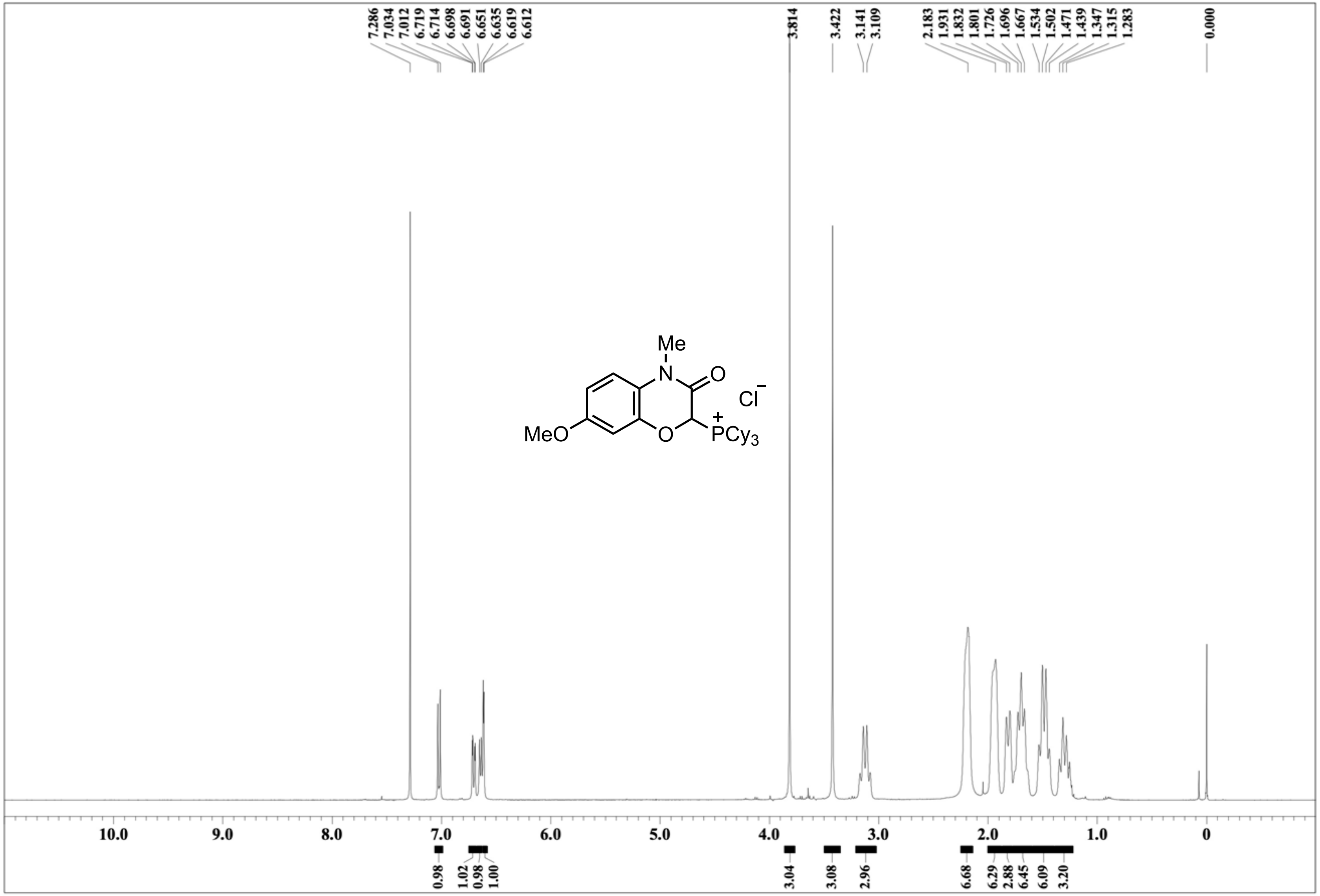

^{1}H NMR (400 MHz, $CDCl_3$) spectrum of **2l**

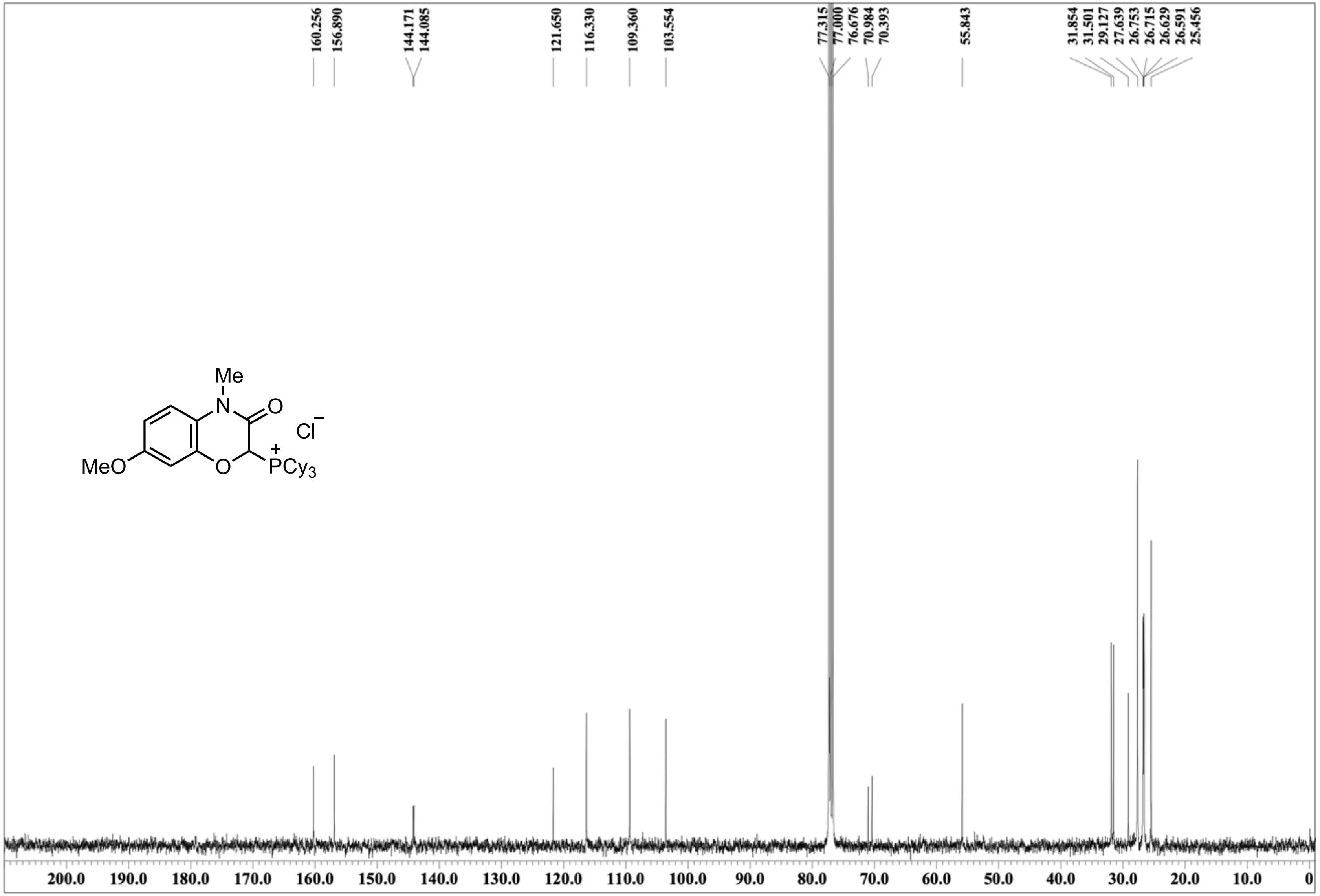

^{13}C NMR (100.6 MHz, $CDCl_3$) spectrum of **2l**

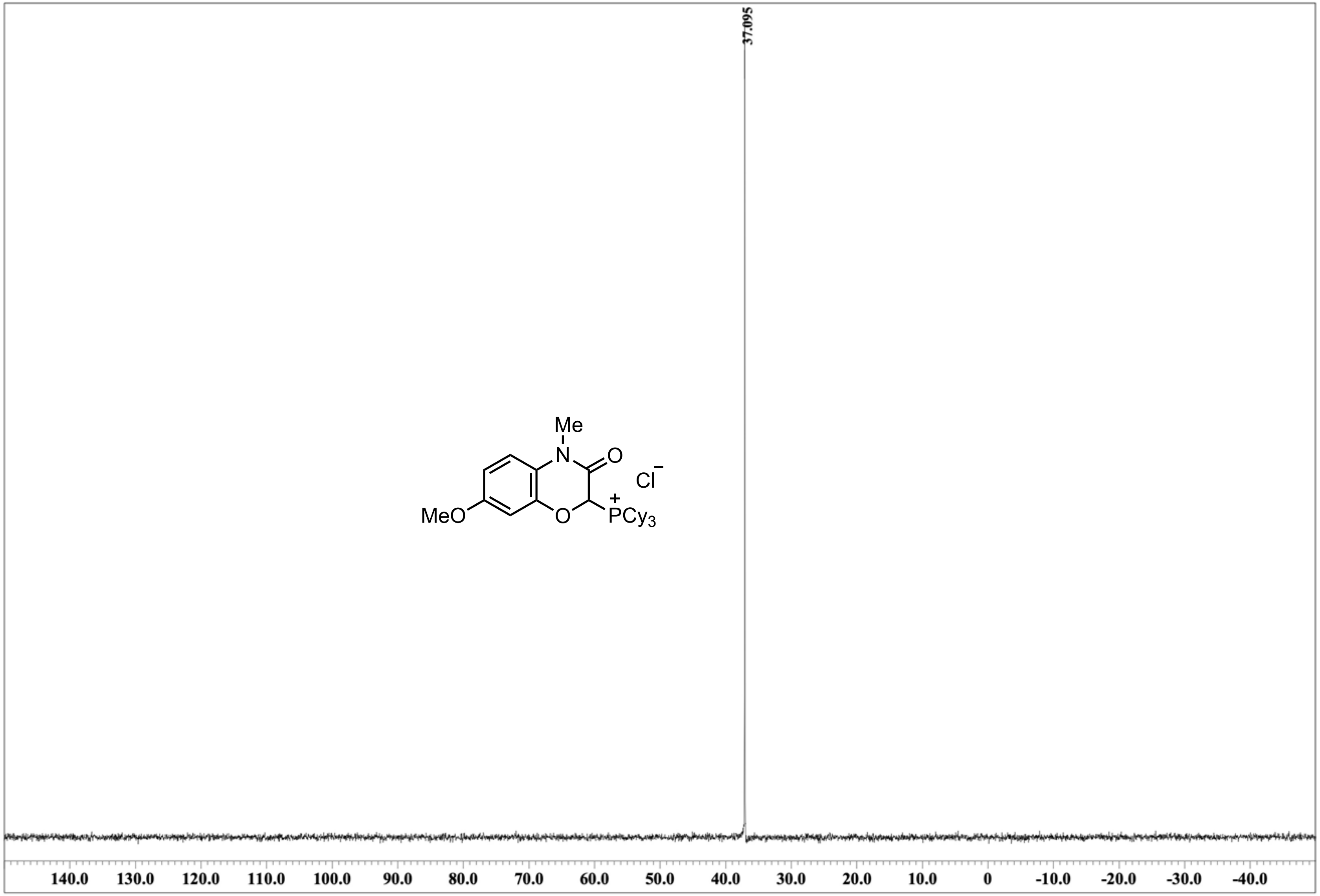

^{31}P NMR (162 MHz, $CDCl_3$) spectrum of **2l**

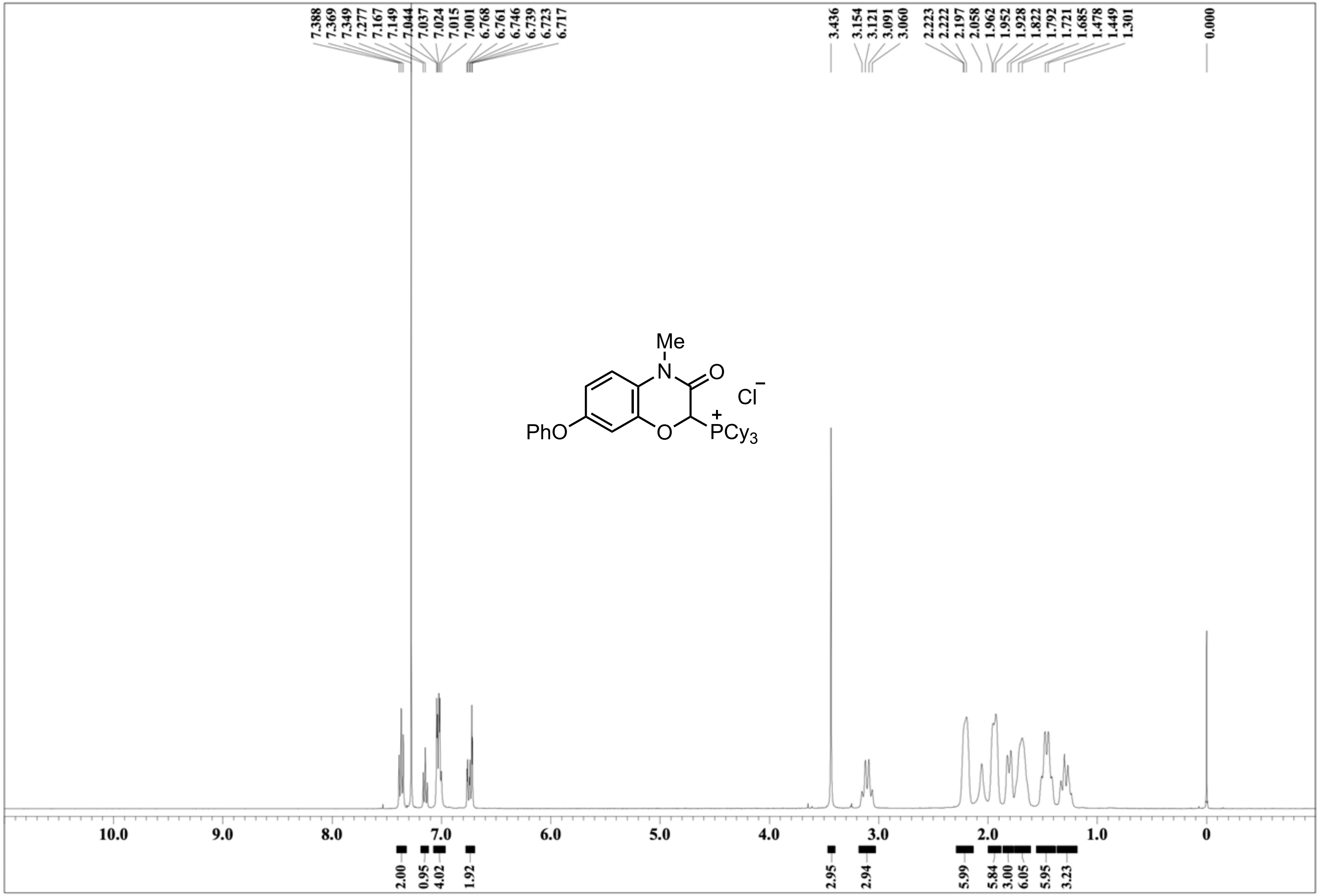

^{1}H NMR (400 MHz, $CDCl_3$) spectrum of **2m**

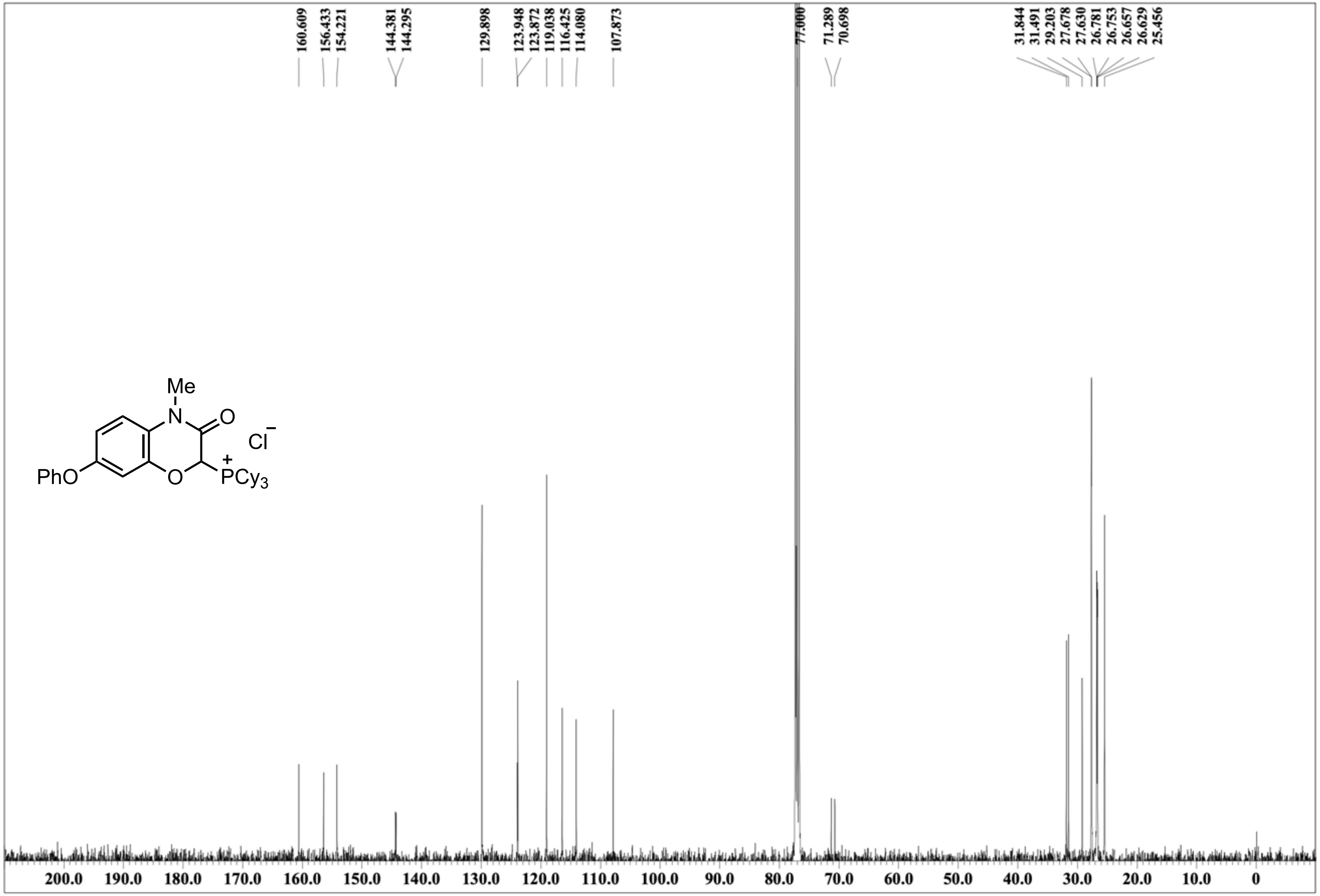

^{13}C NMR (100.6 MHz, $CDCl_3$) spectrum of **2m**

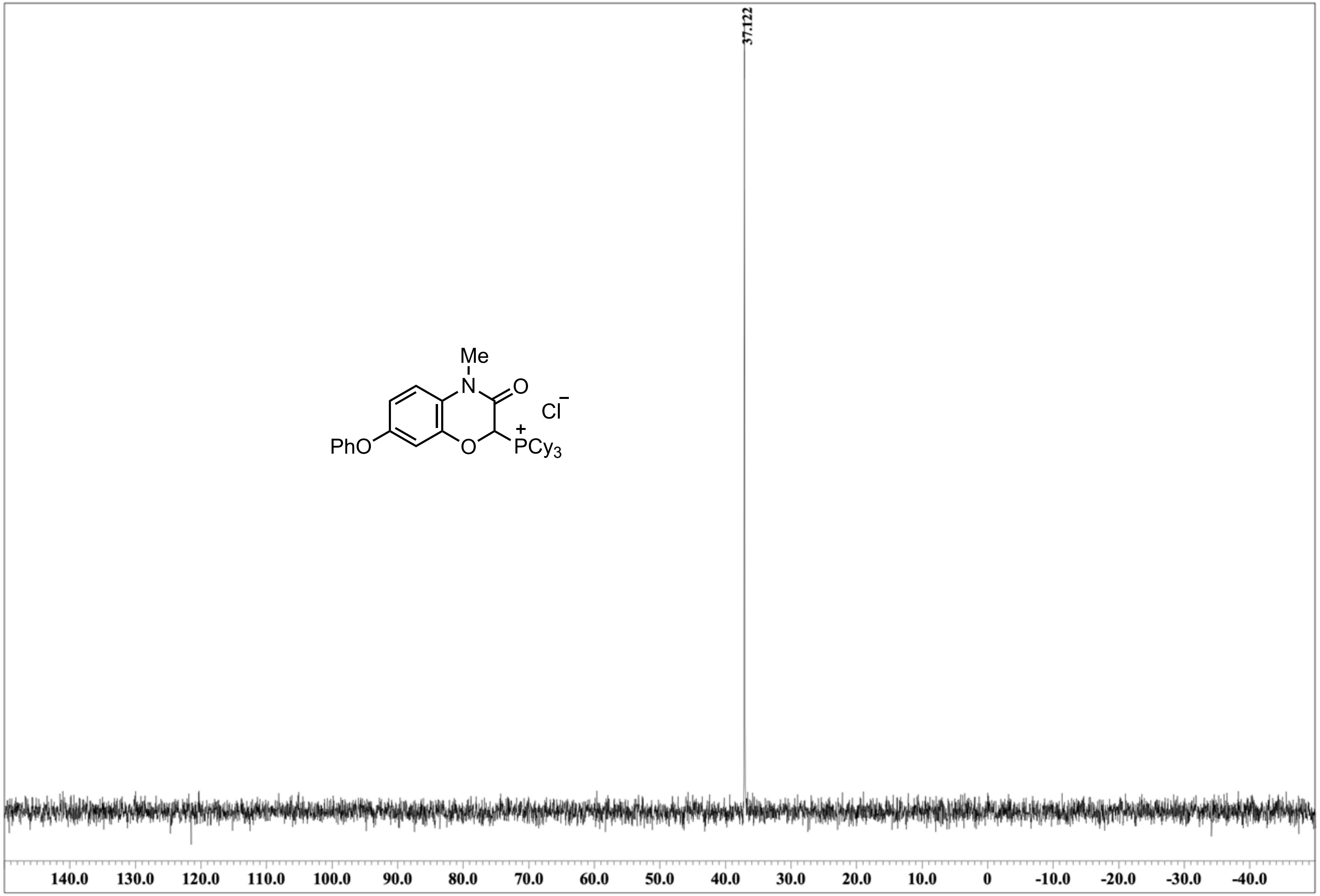

^{31}P NMR (162 MHz, $CDCl_3$) spectrum of **2m**

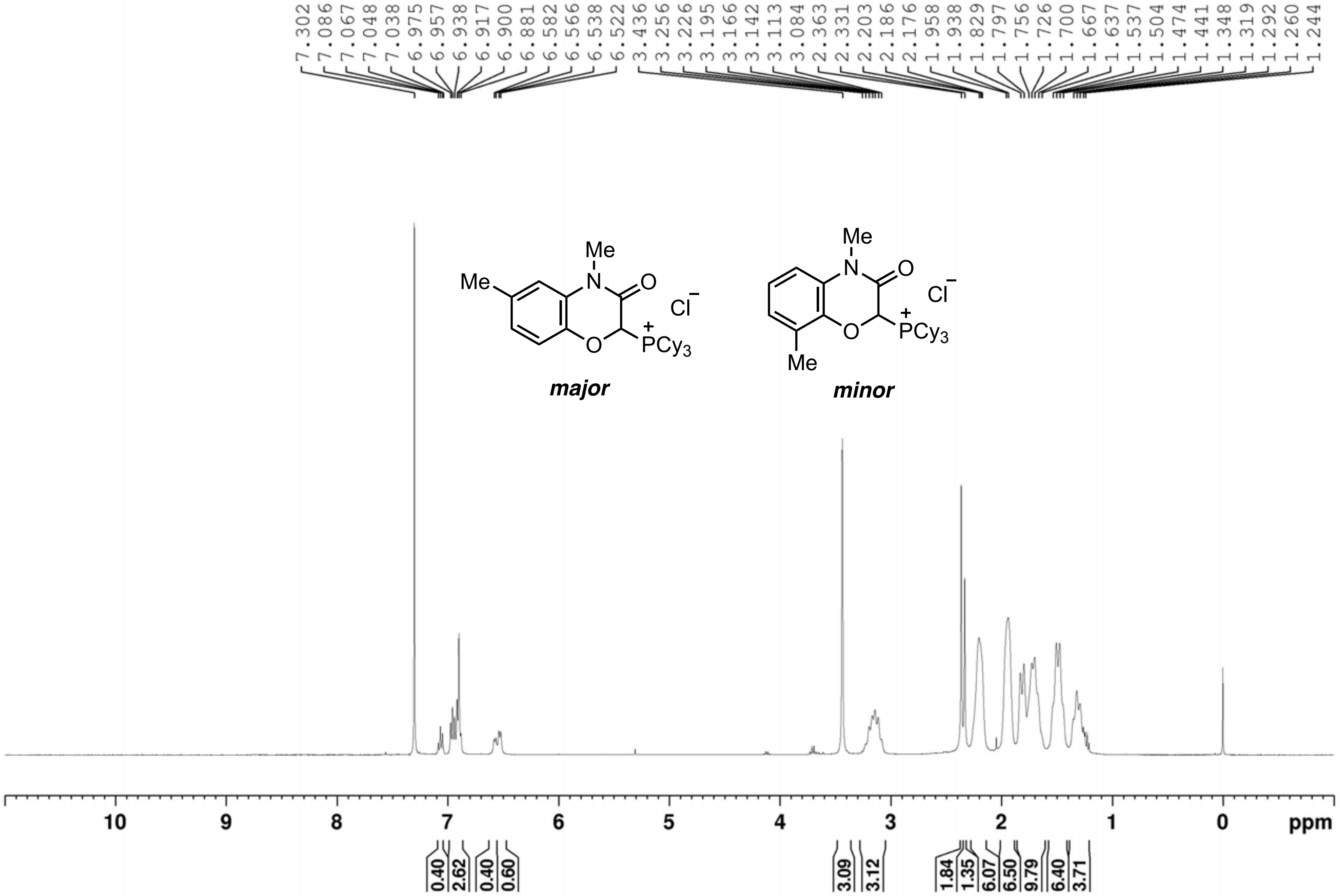

^1H NMR (400 MHz, $CDCl_3$) spectrum of **2n**

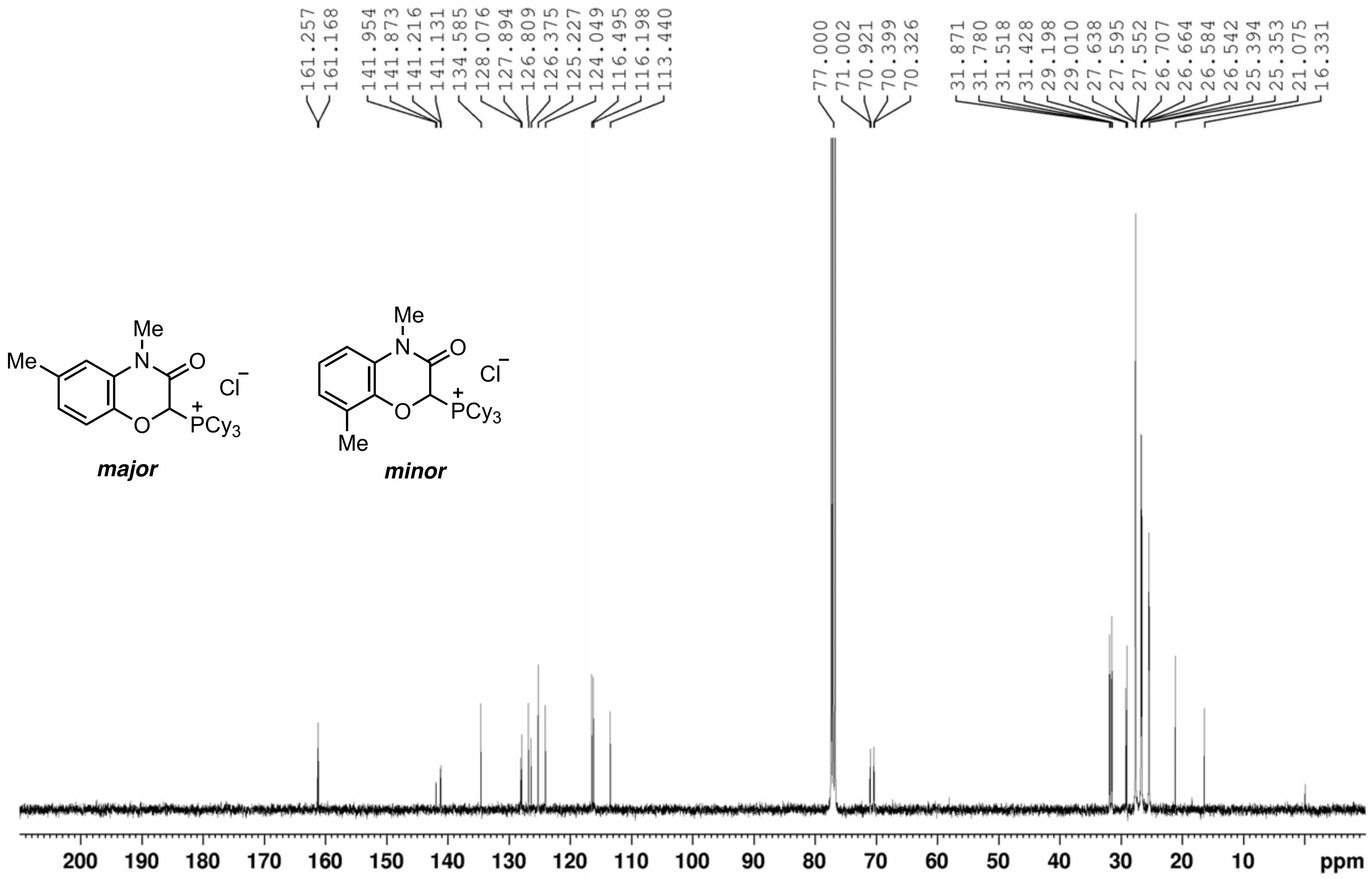

^{13}C NMR (100.6 MHz, $CDCl_3$) spectrum of **2n**

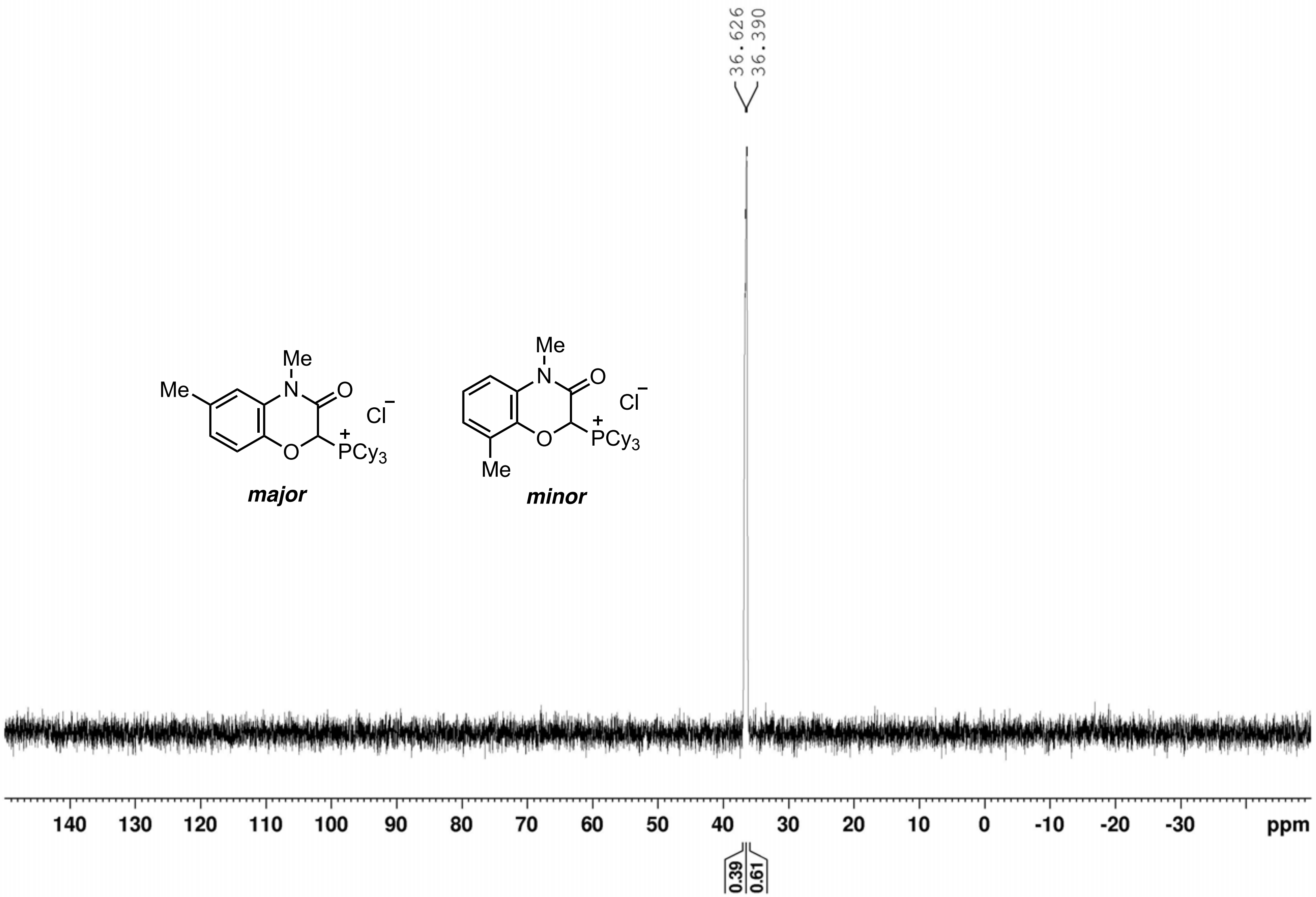

^{31}P NMR (162 MHz, $CDCl_3$) spectrum of **2n**

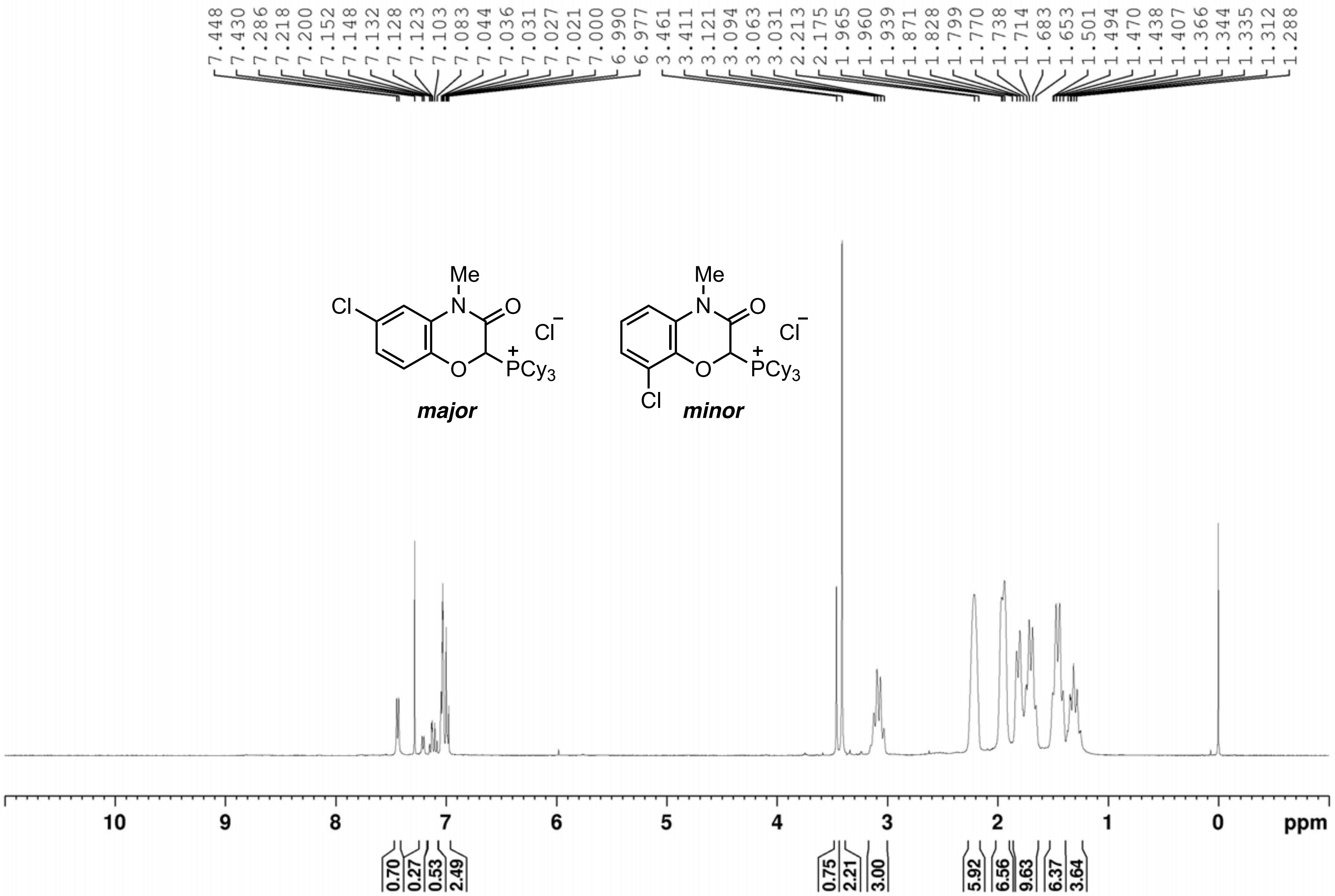

^{1}H NMR (400 MHz, $CDCl_3$) spectrum of **2o**

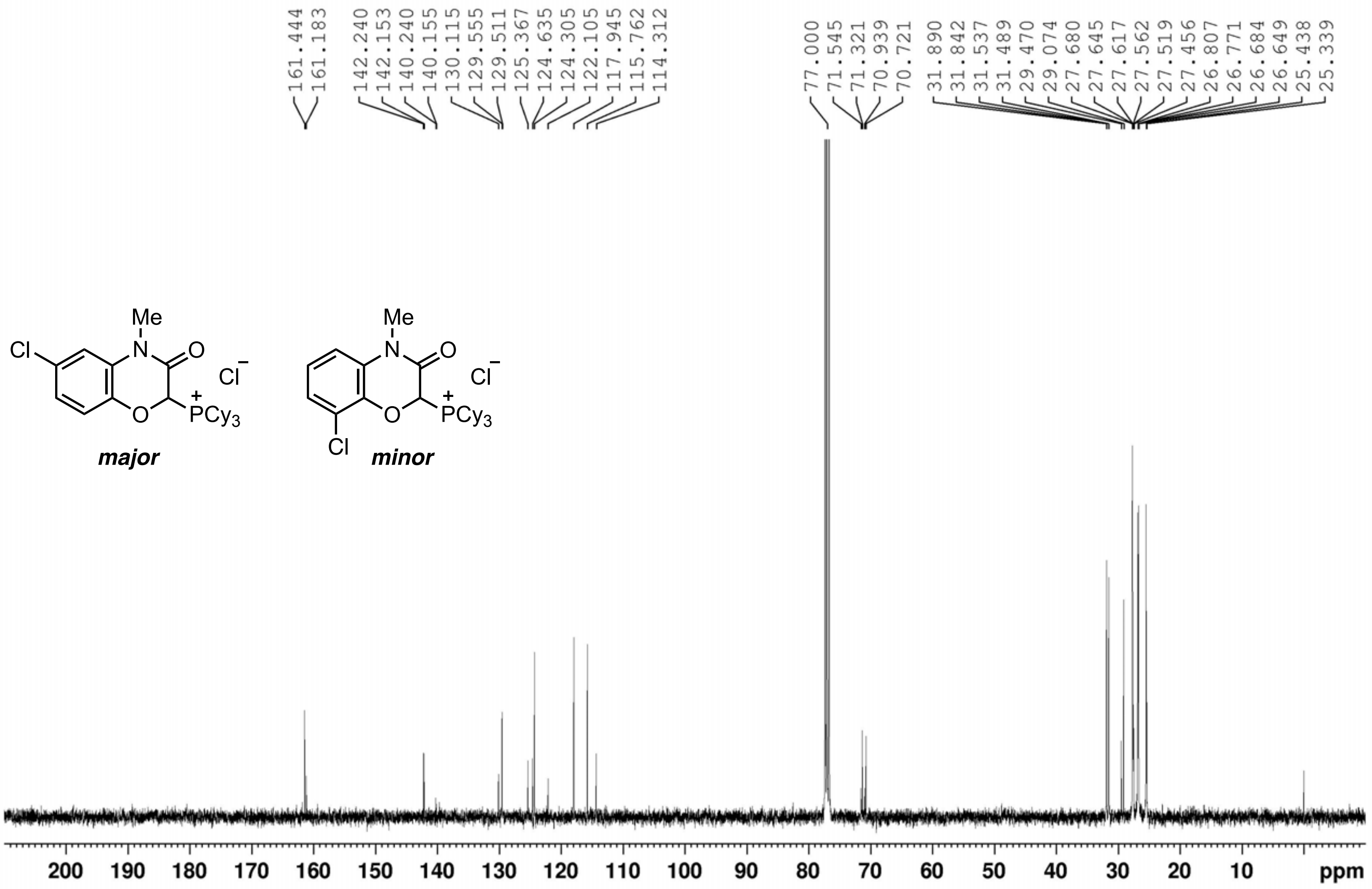

^{13}C NMR (100.6 MHz, $CDCl_3$) spectrum of **2o**

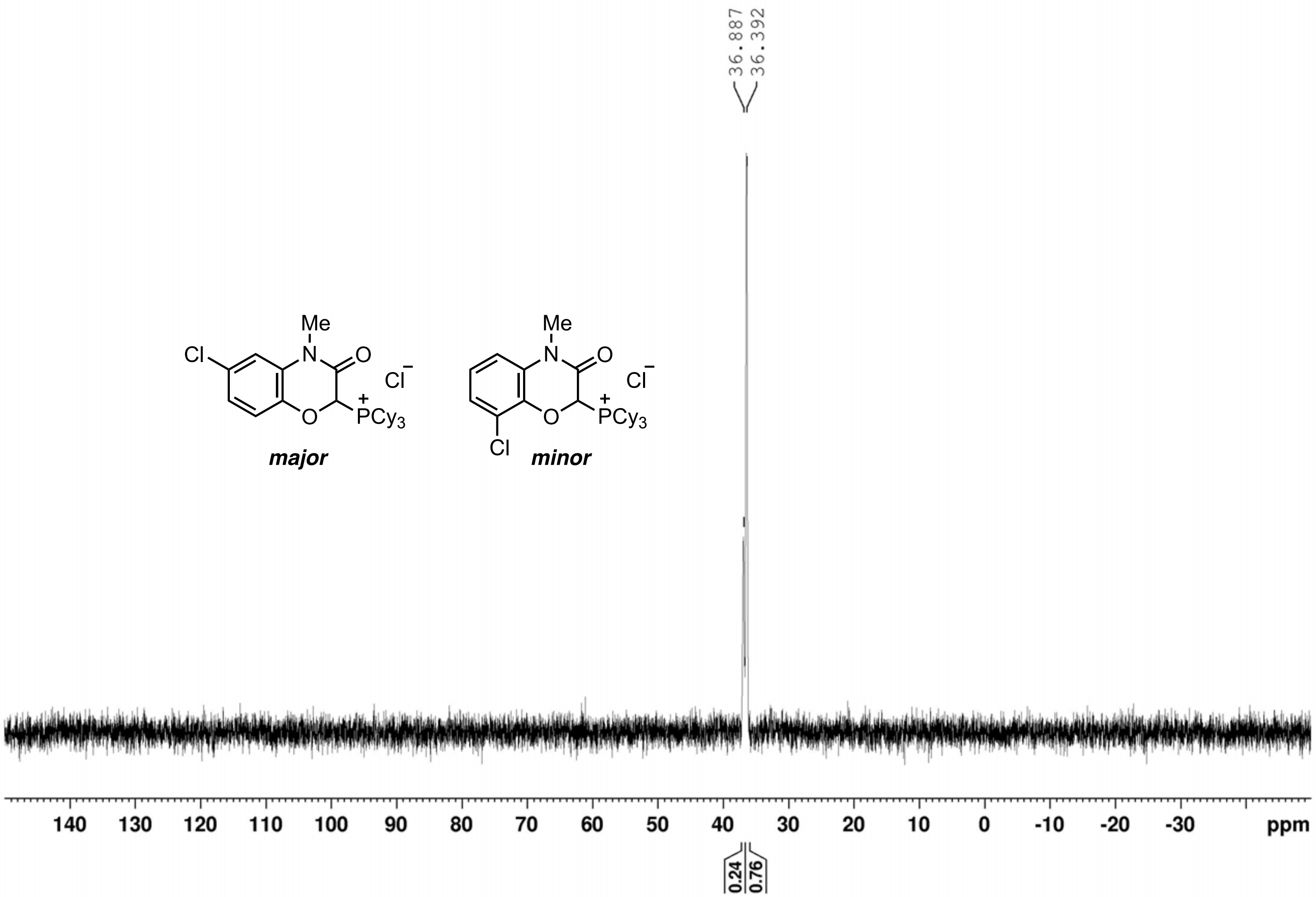

^{31}P NMR (162 MHz, $CDCl_3$) spectrum of **2o**

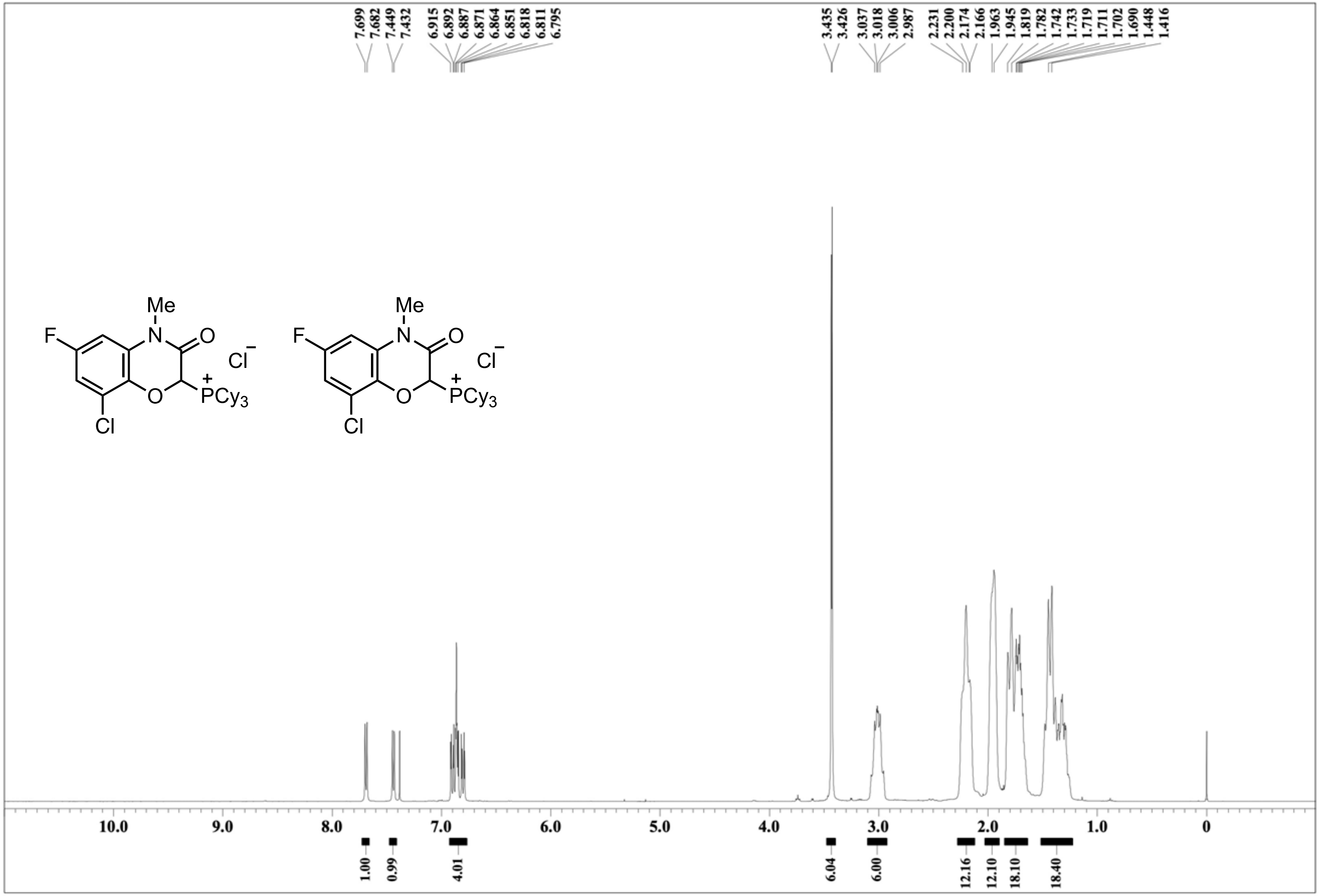

^{1}H NMR (400 MHz, $CDCl_3$) spectrum of **2p**

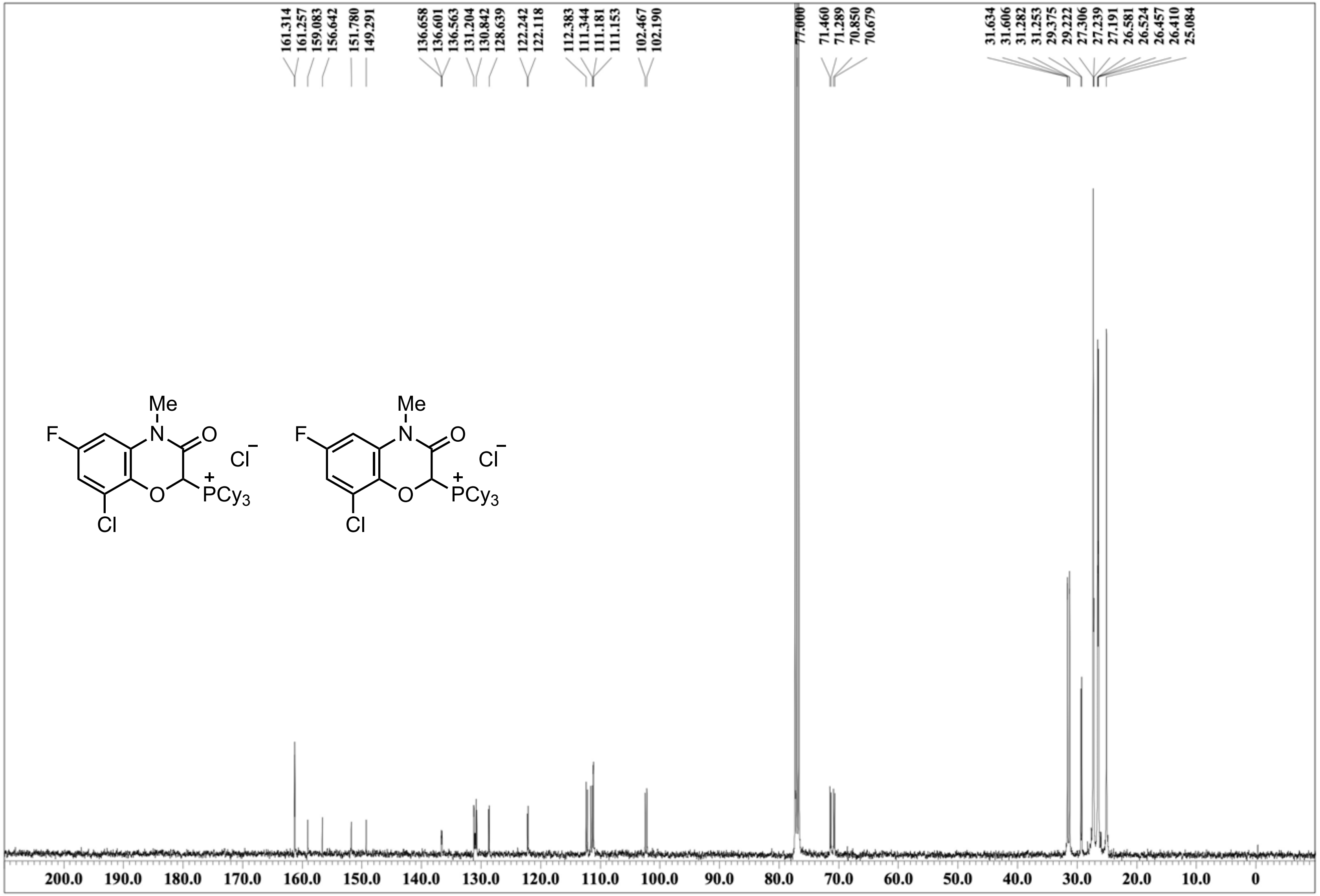

^{13}C NMR (100.6 MHz, $CDCl_3$) spectrum of **2p**

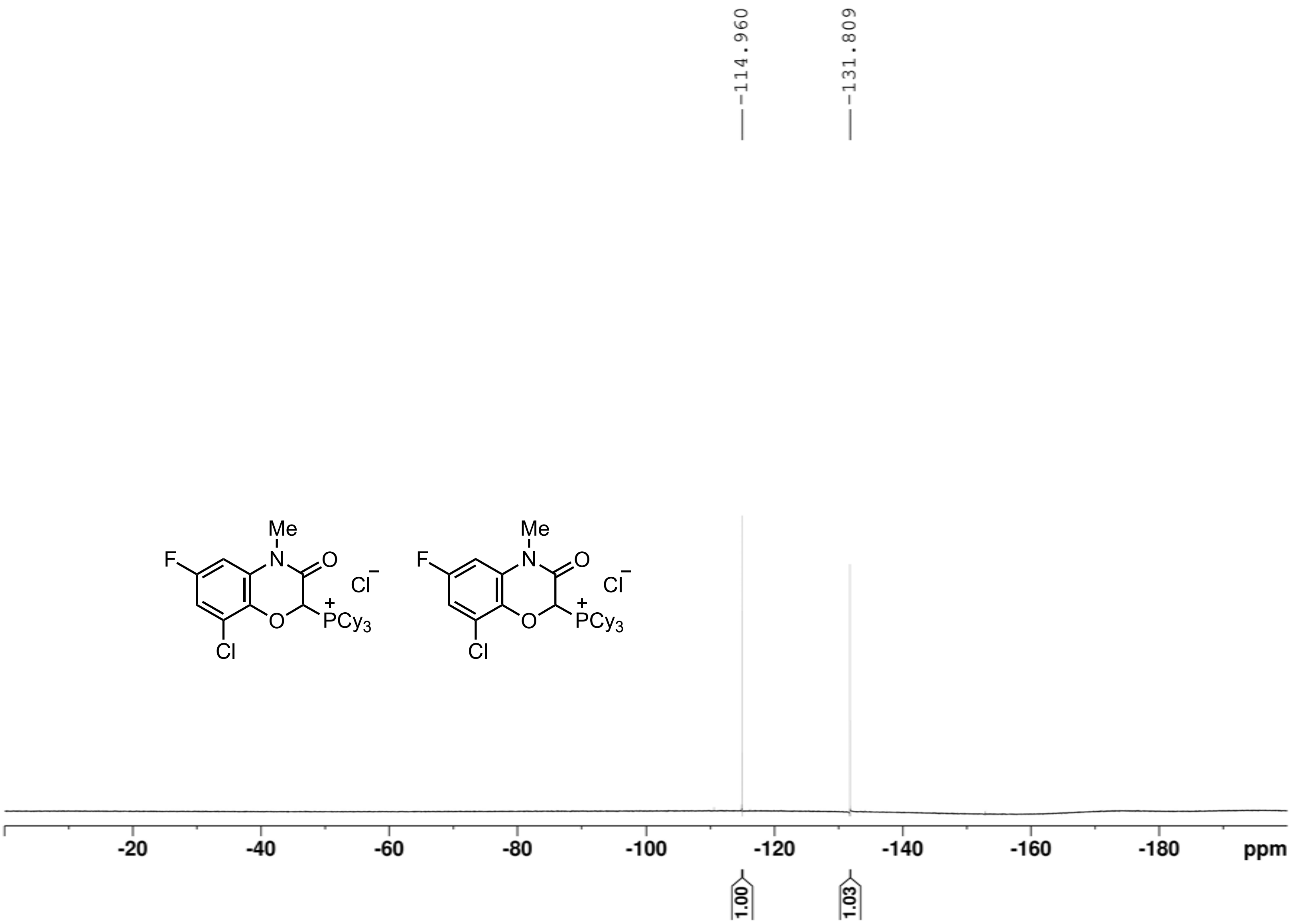

^{19}F NMR (376.5 MHz, $CDCl_3$) spectrum of **2p**

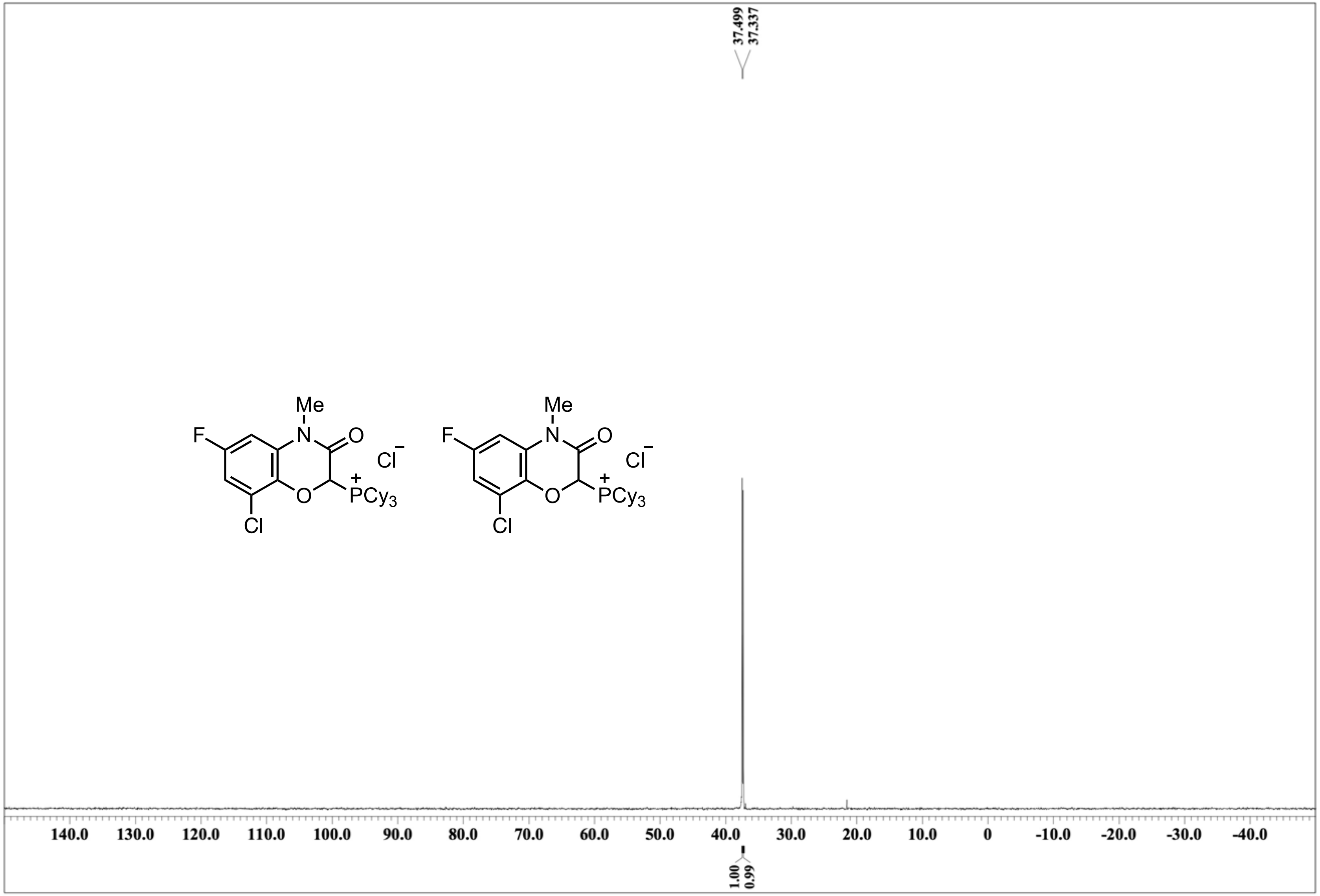

^{31}P NMR (162 MHz, $CDCl_3$) spectrum of **2p**

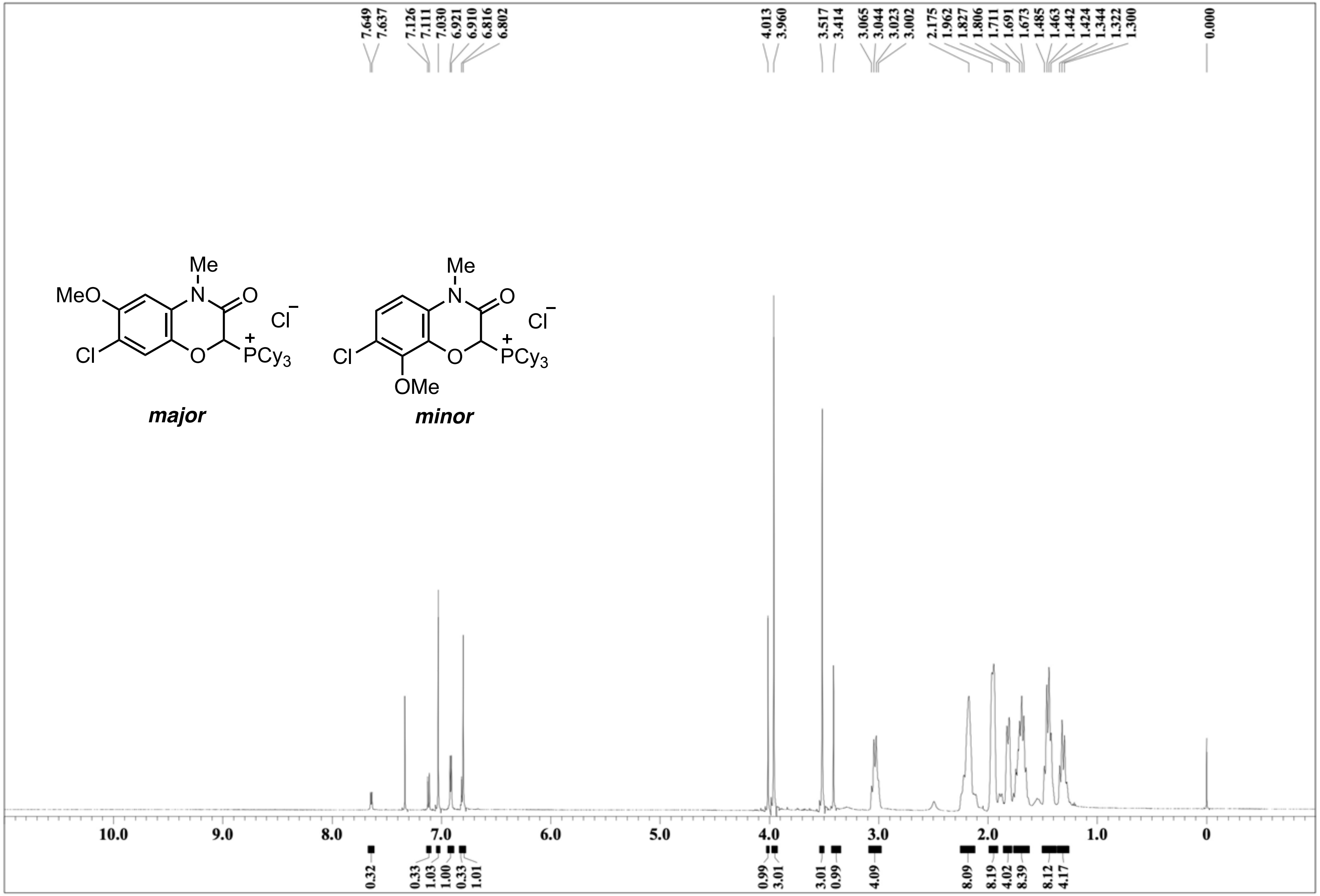

^{1}H NMR (400 MHz, $CDCl_3$) spectrum of **2q**

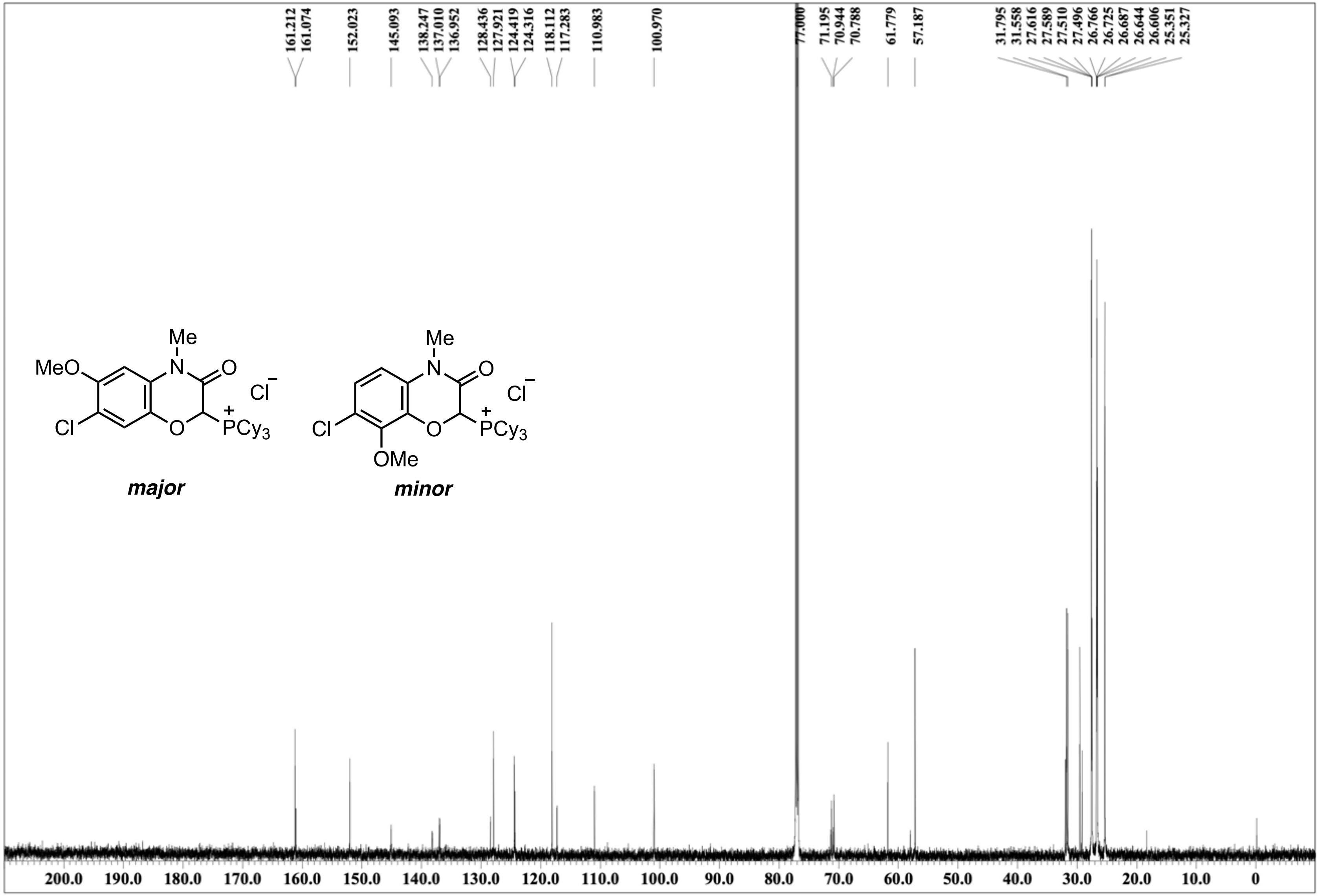

^{13}C NMR (100.6 MHz, $CDCl_3$) spectrum of **2q**

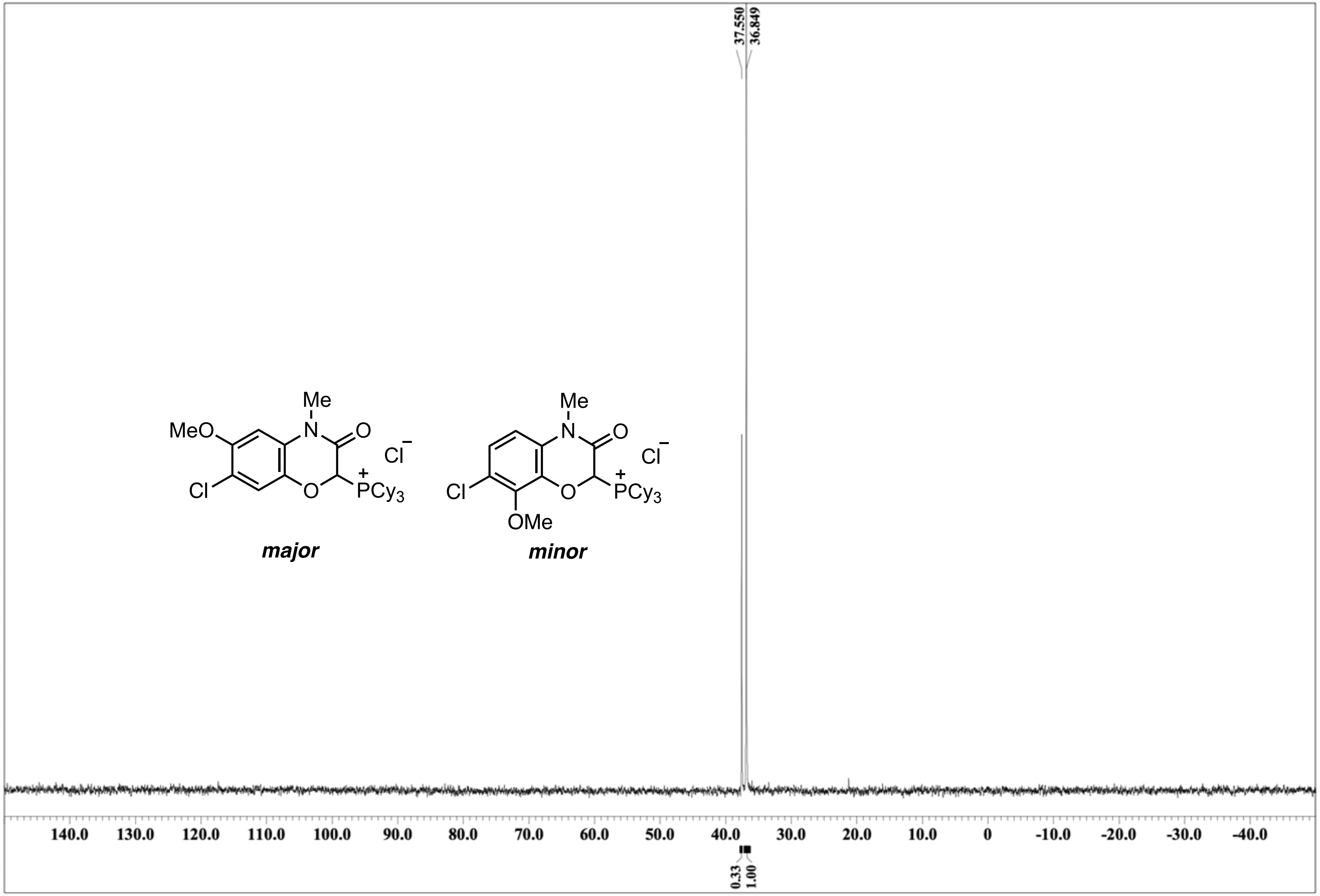

^{31}P NMR (162 MHz, $CDCl_3$) spectrum of **2q**

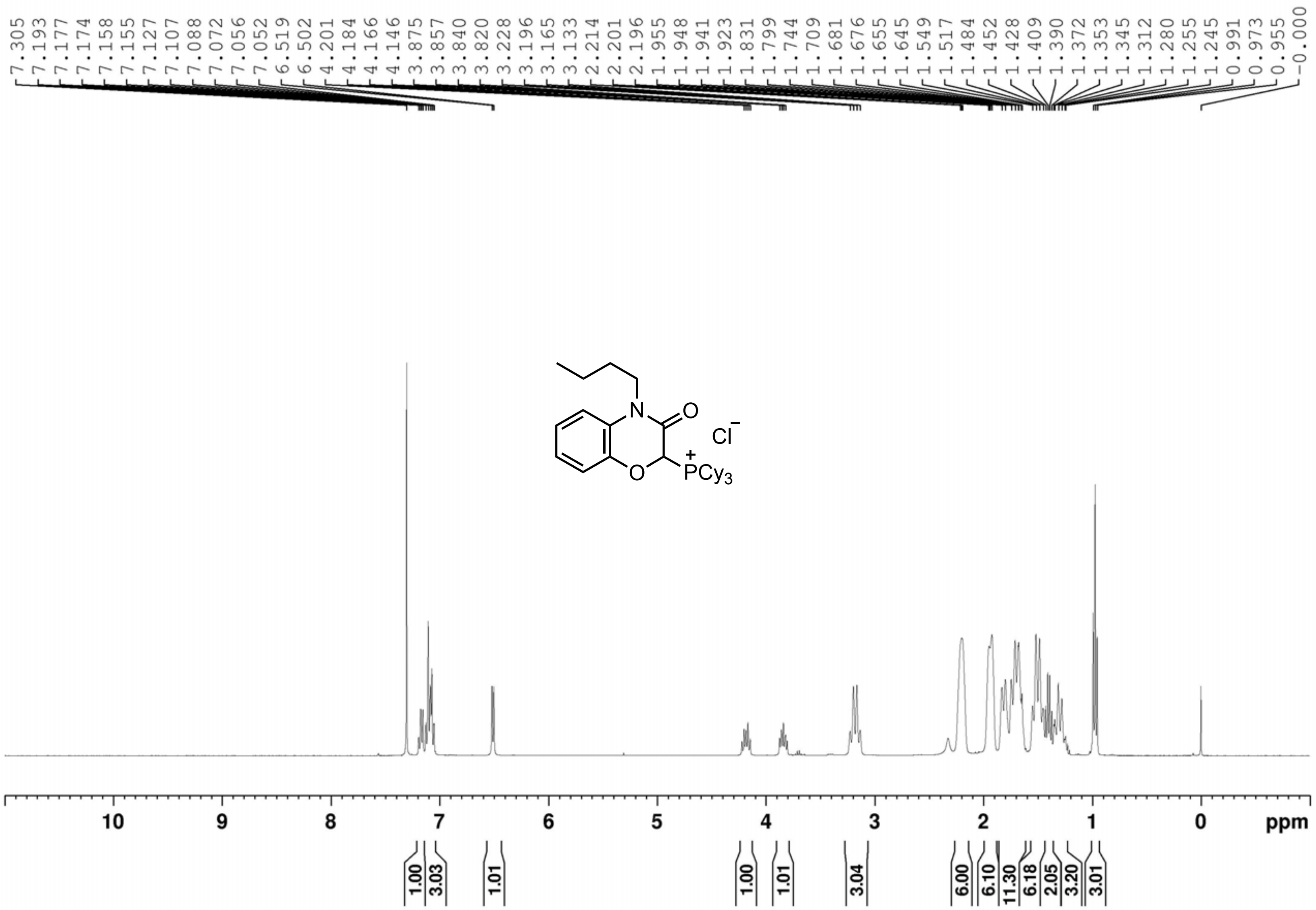

^{1}H NMR (400 MHz, $CDCl_3$) spectrum of **2r**

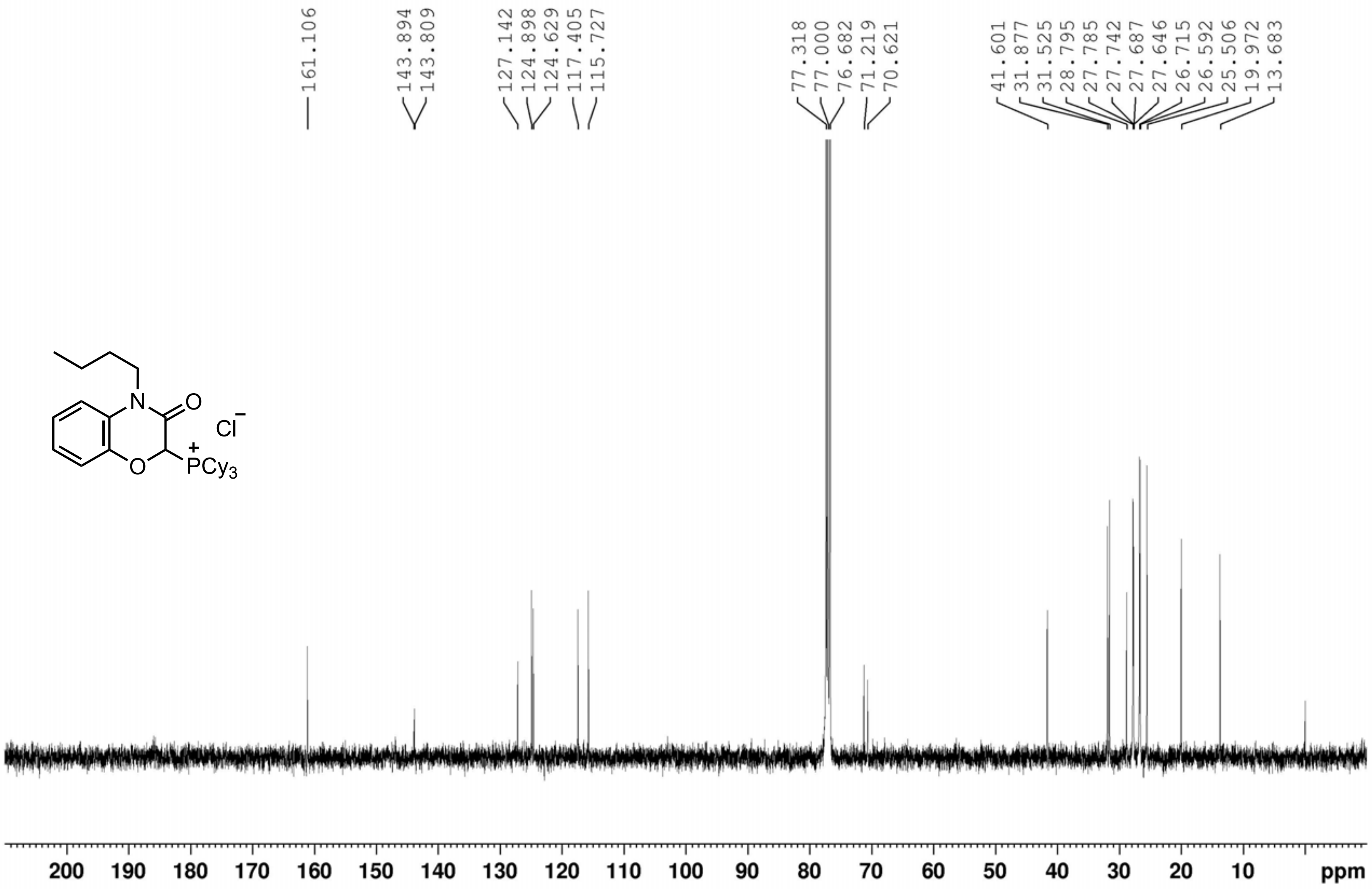

^{13}C NMR (100.6 MHz, $CDCl_3$) spectrum of **2r**

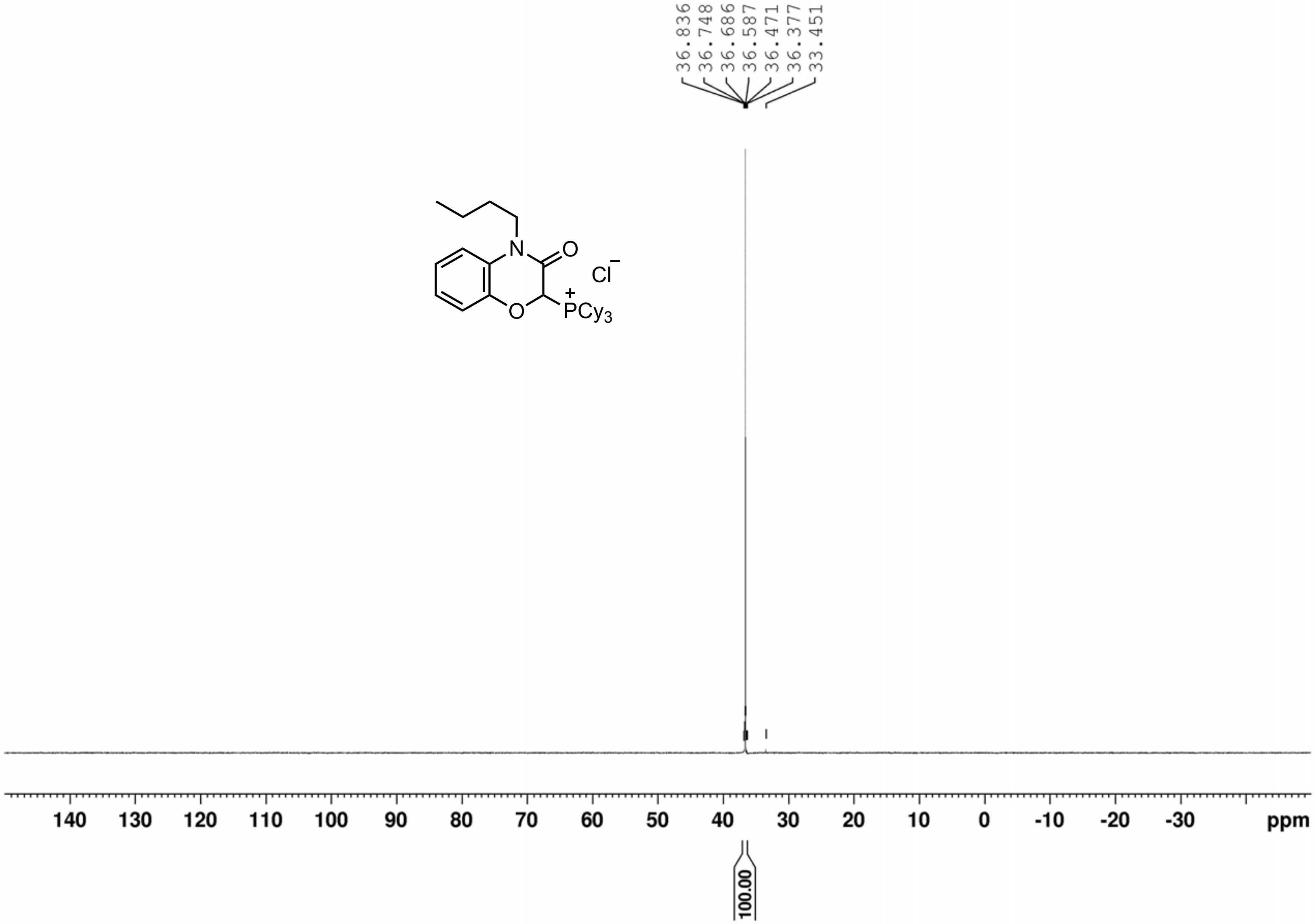

^{31}P NMR (162 MHz, $CDCl_3$) spectrum of **2r**

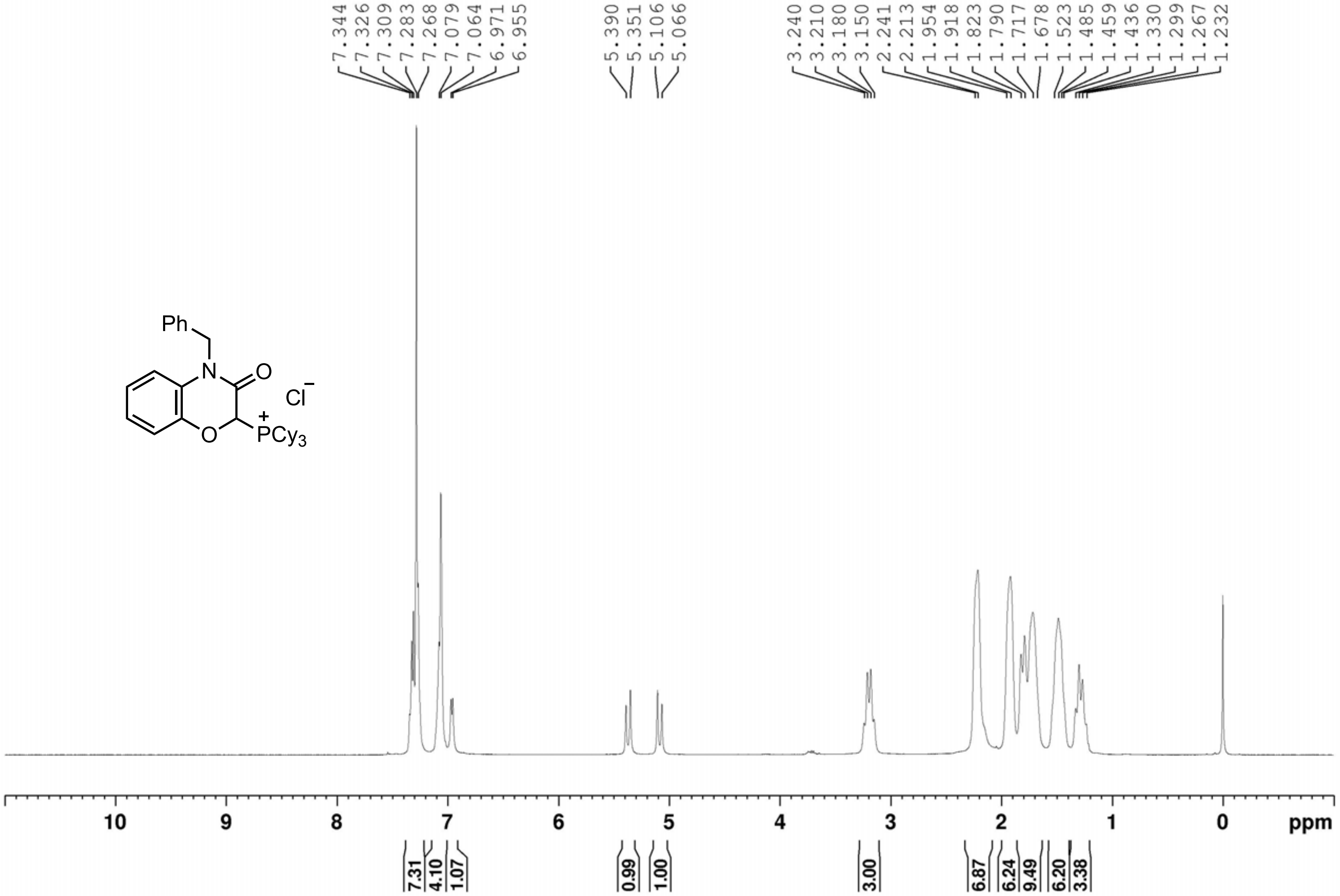

^{1}H NMR (400 MHz, $CDCl_3$) spectrum of **2s**

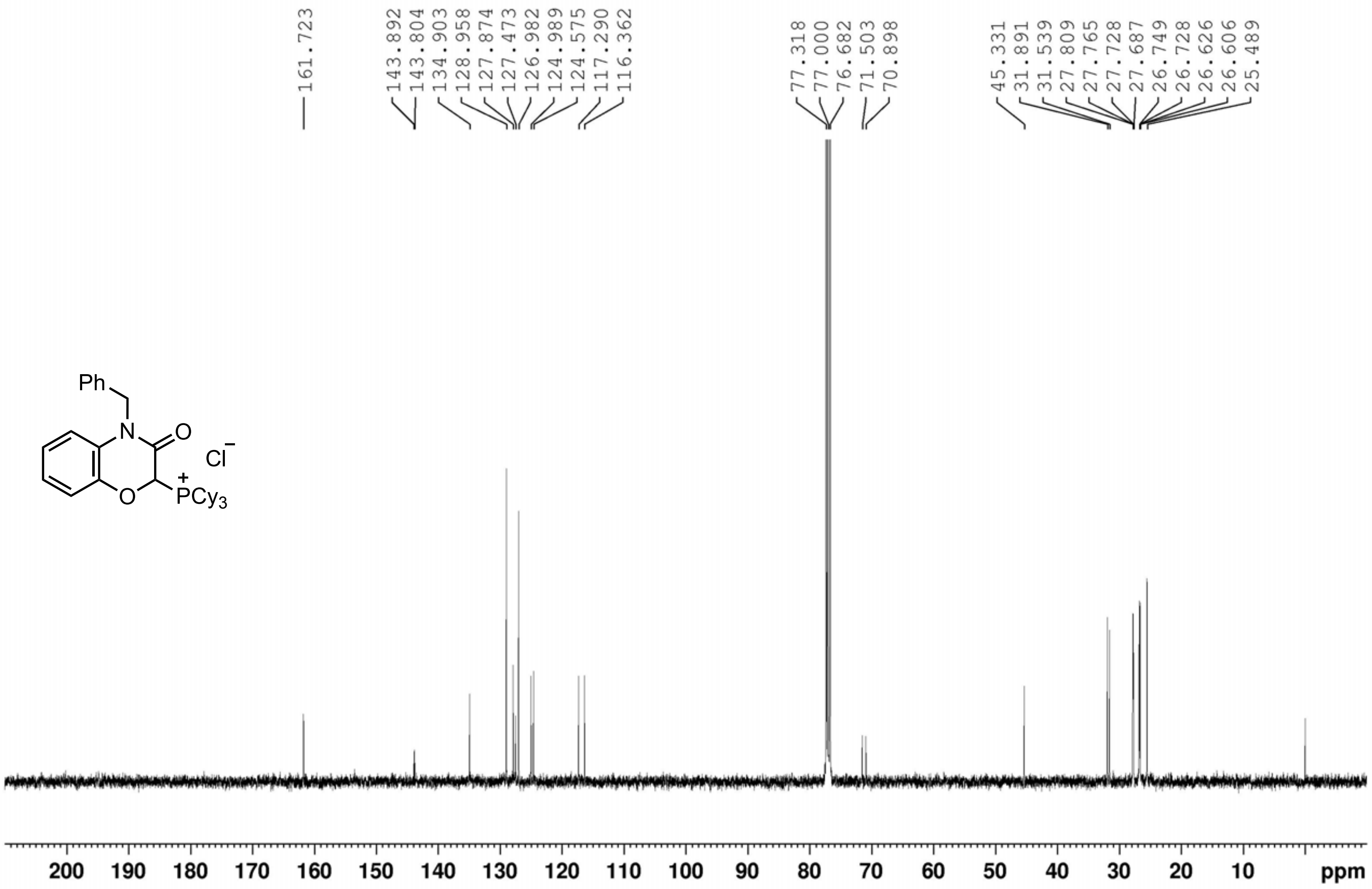

^{13}C NMR (100.6 MHz, $CDCl_3$) spectrum of **2s**

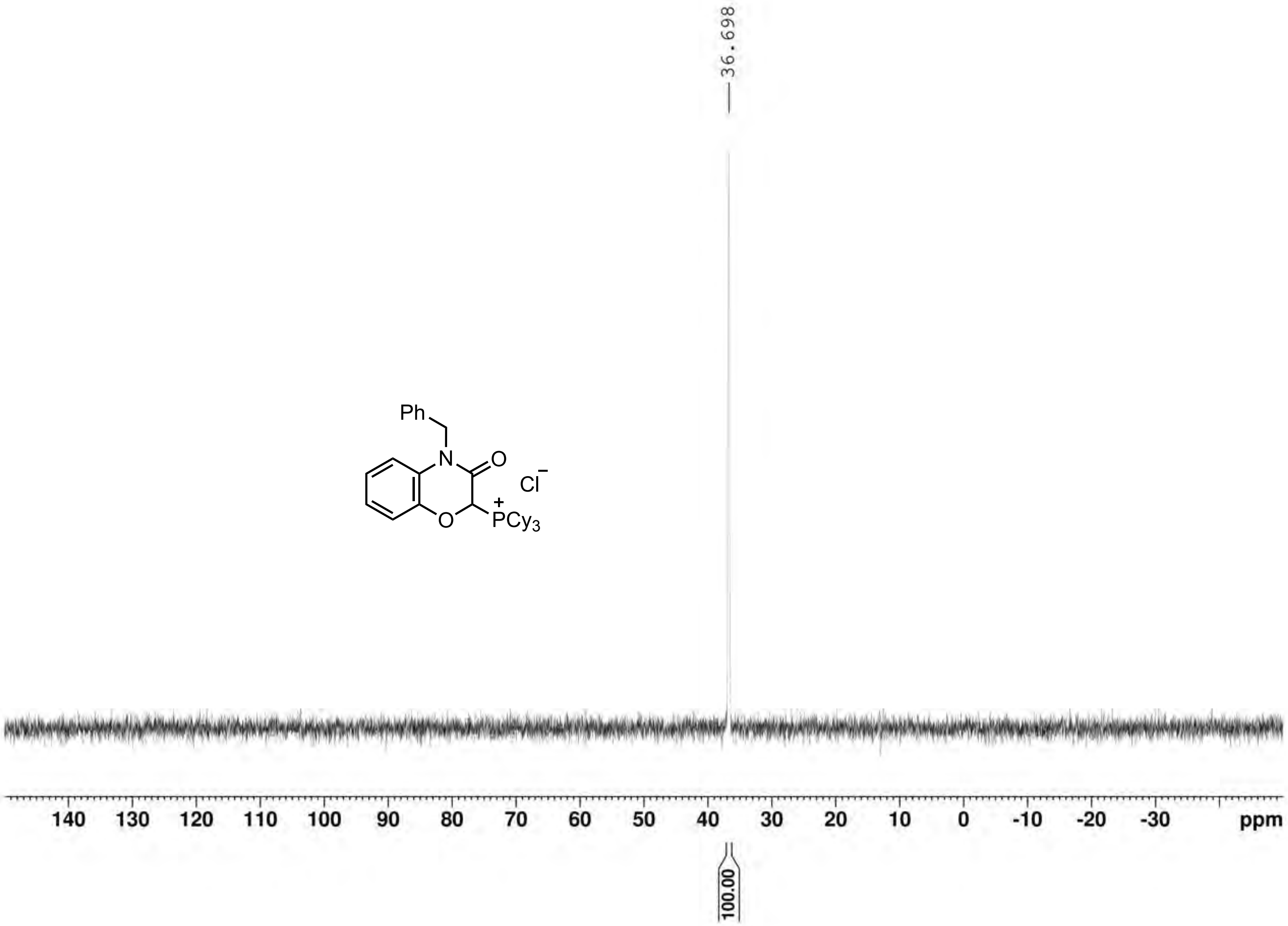

^{31}P NMR (162 MHz, $CDCl_3$) spectrum of **2s**

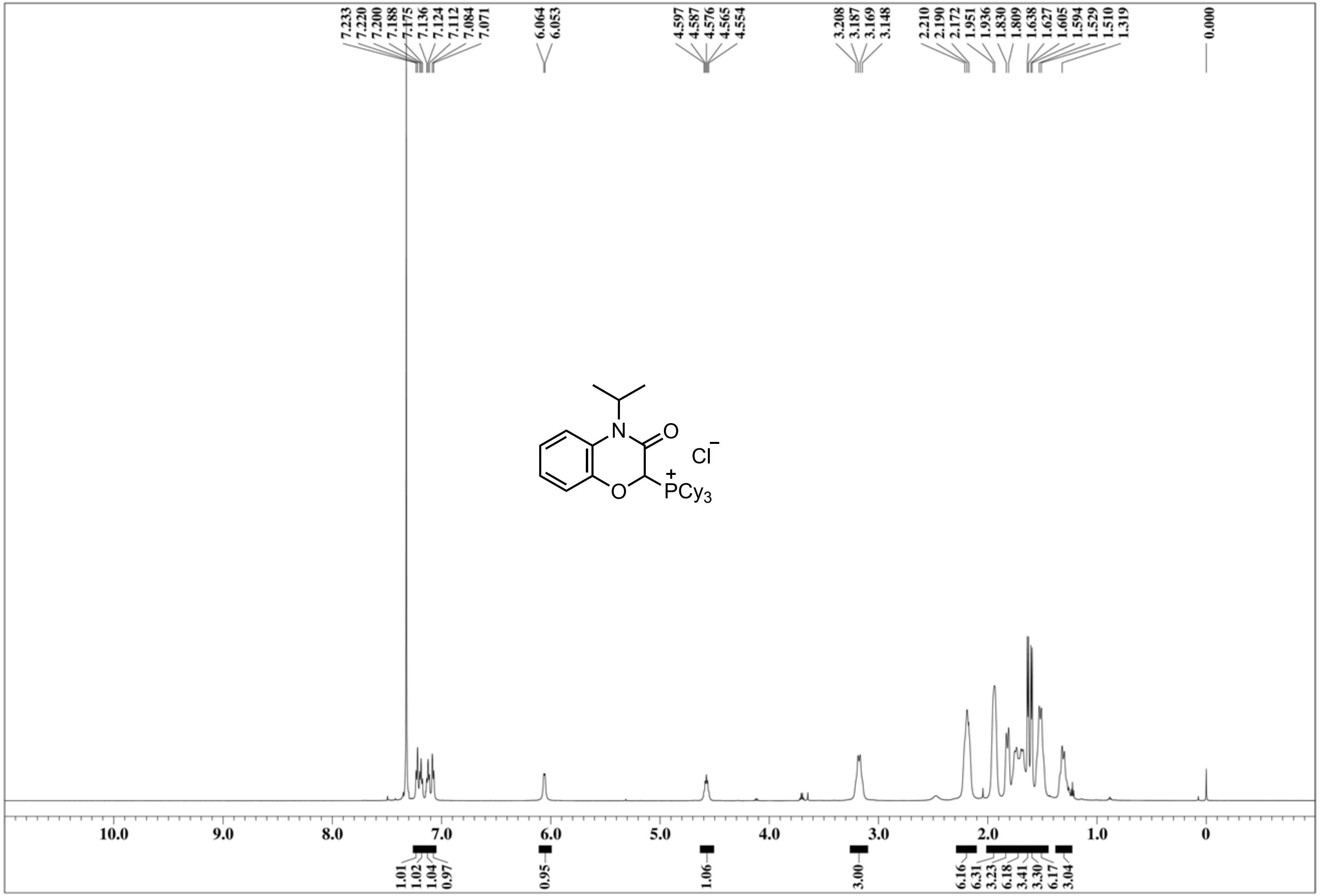

^{1}H NMR (400 MHz, $CDCl_3$) spectrum of **2t**

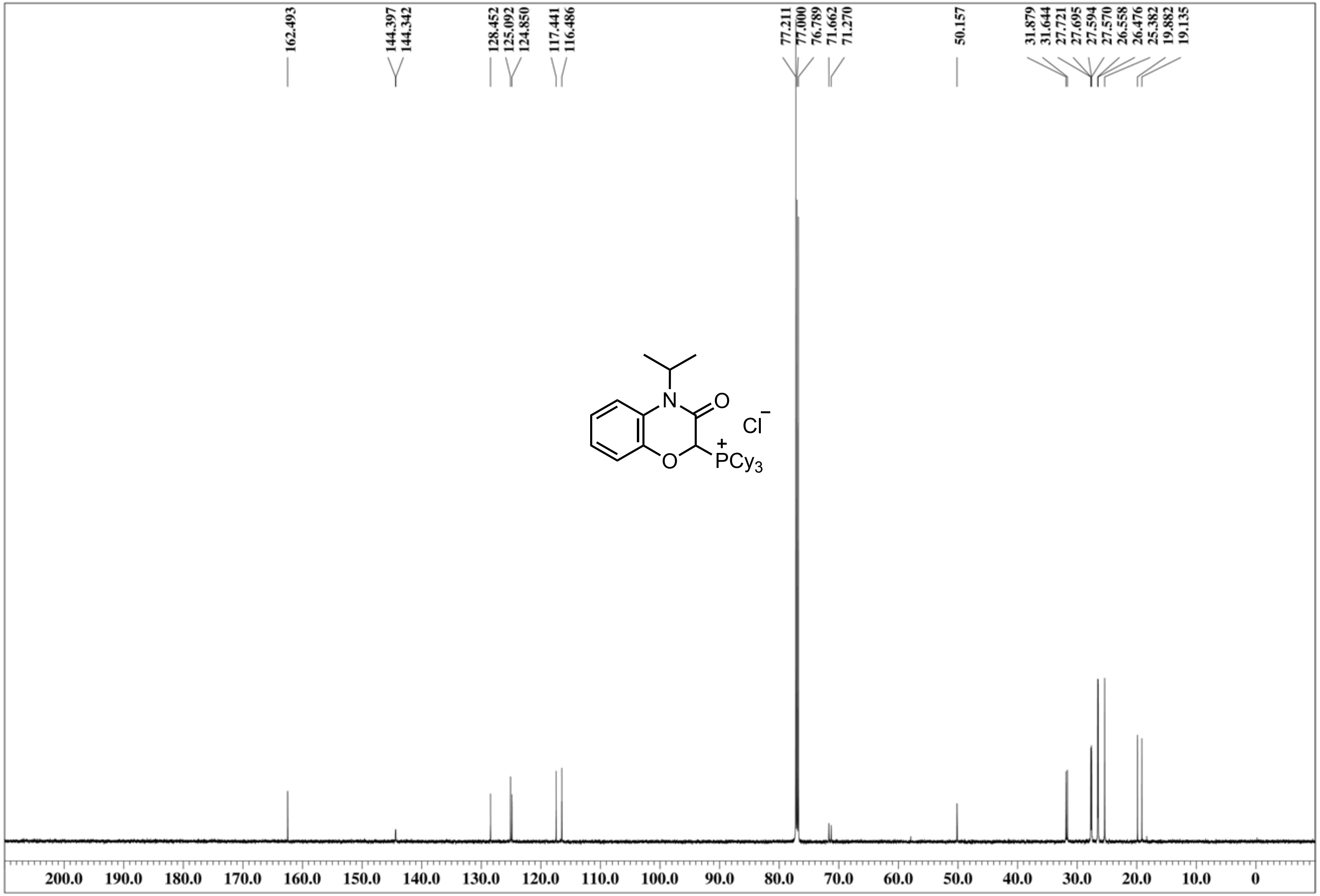

^{13}C NMR (100.6 MHz, $CDCl_3$) spectrum of **2t**

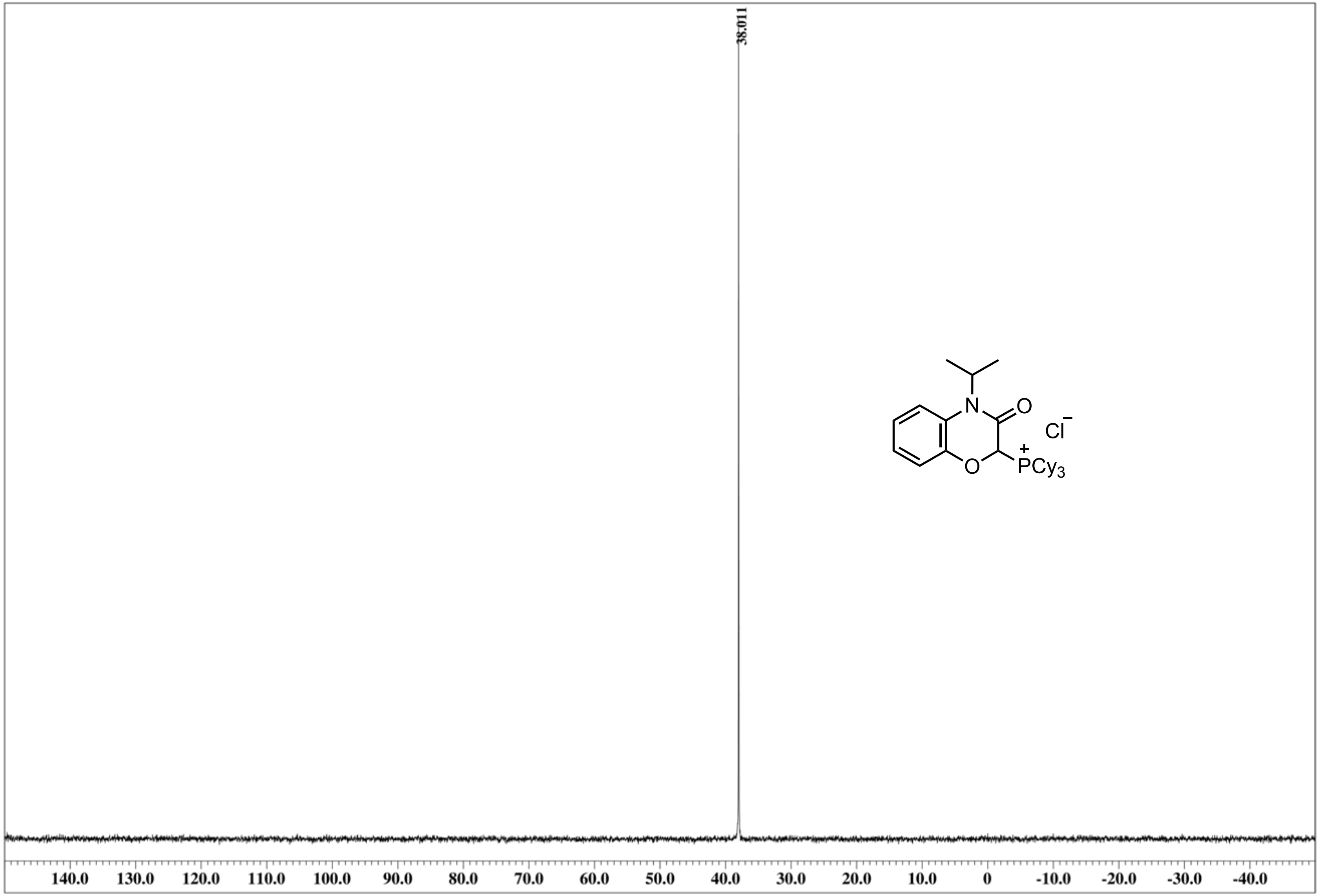

^{31}P NMR (162 MHz, $CDCl_3$) spectrum of **2t**

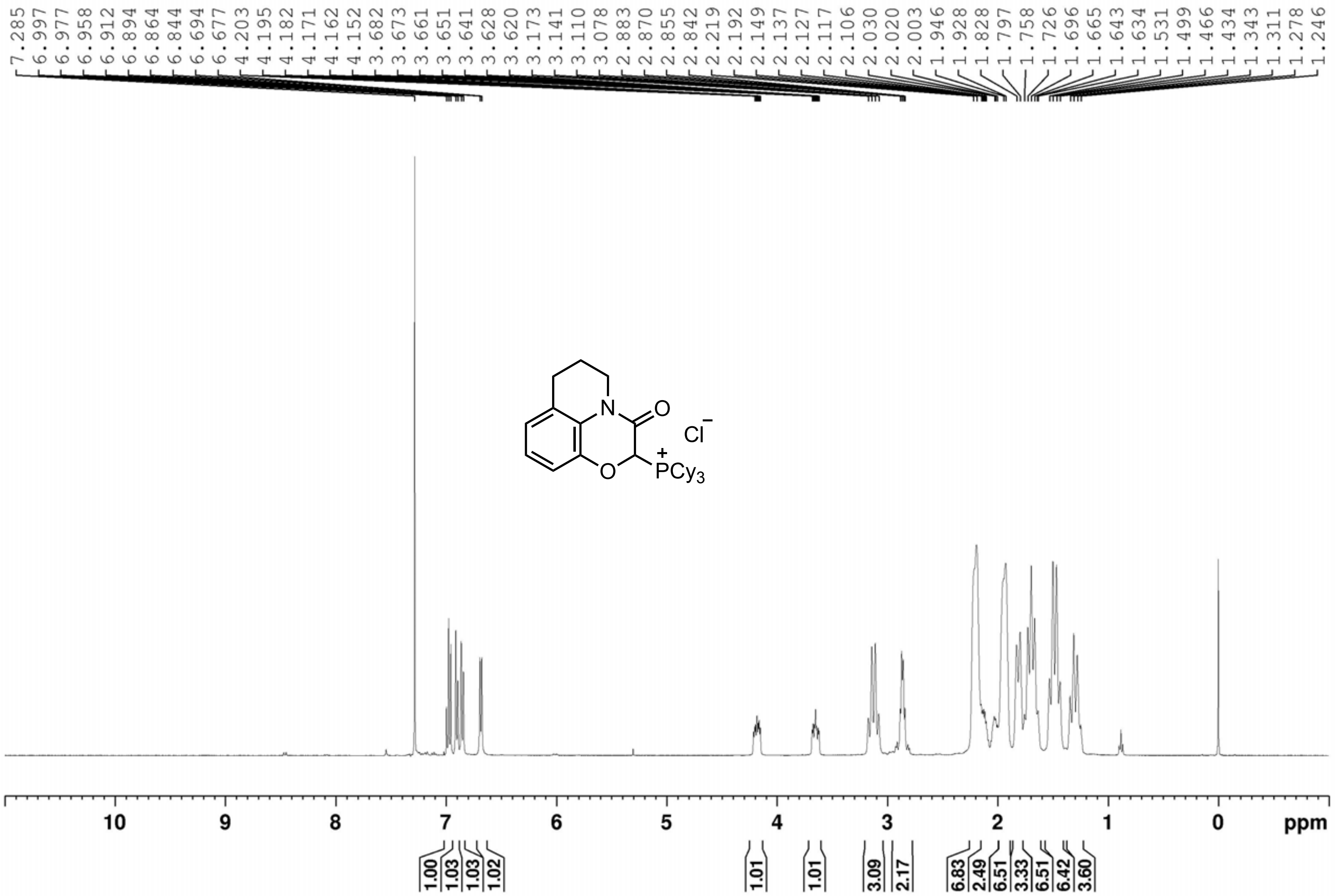

^{1}H NMR (400 MHz, $CDCl_3$) spectrum of **2u**

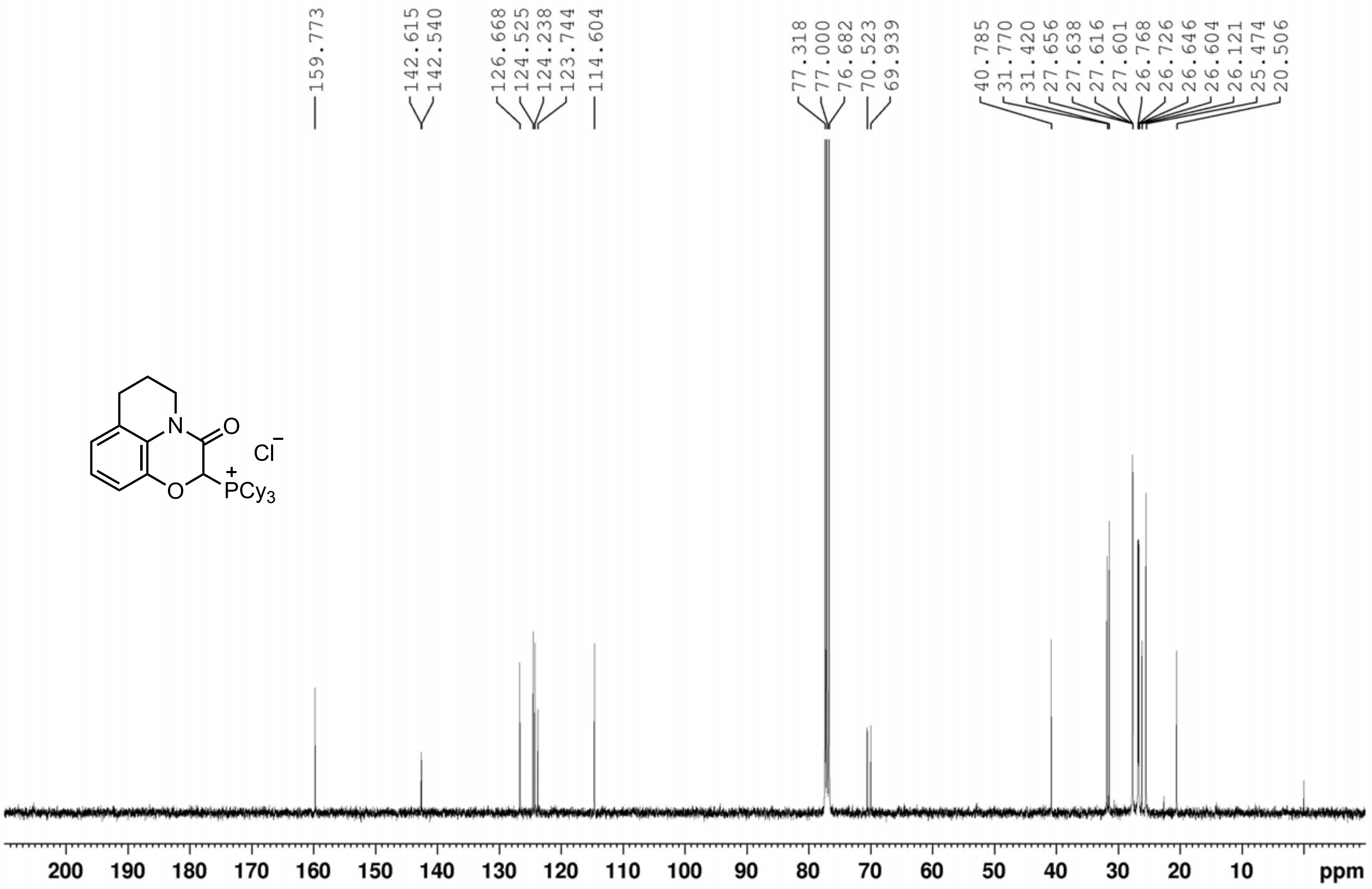

^{13}C NMR (100.6 MHz, $CDCl_3$) spectrum of **2u**

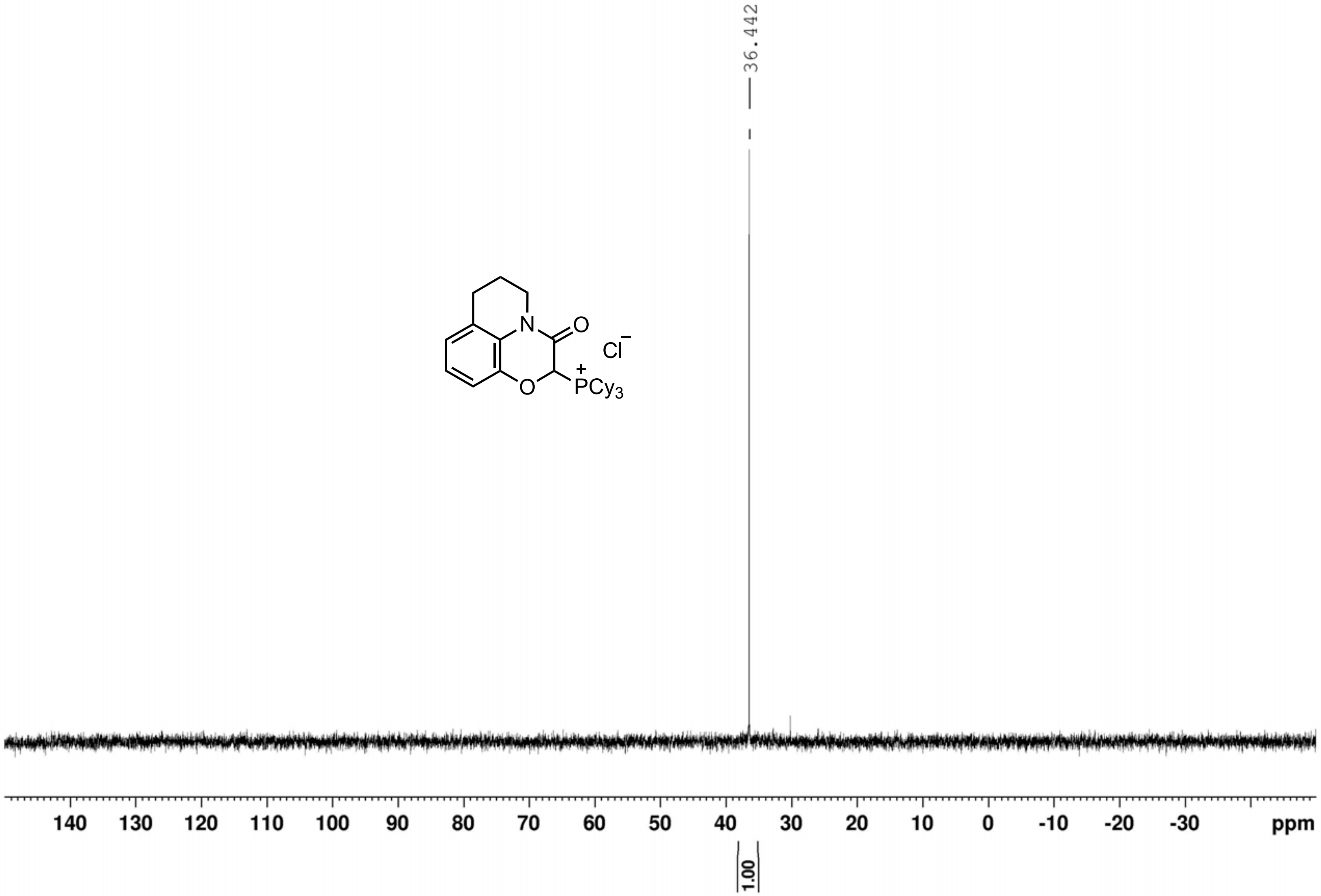

^{31}P NMR (162 MHz, $CDCl_3$) spectrum of **2u**

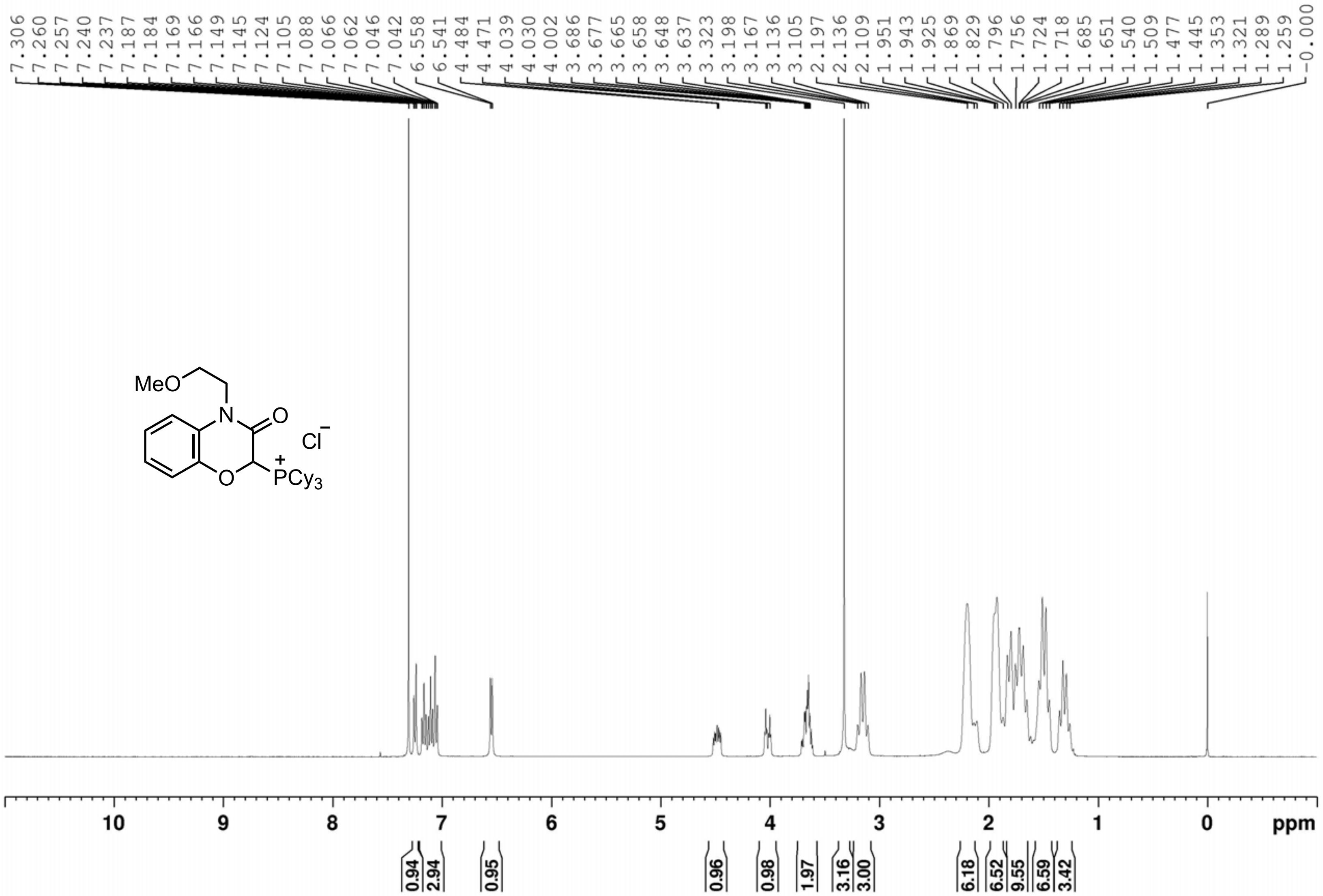

^{1}H NMR (400 MHz, $CDCl_3$) spectrum of **2v**

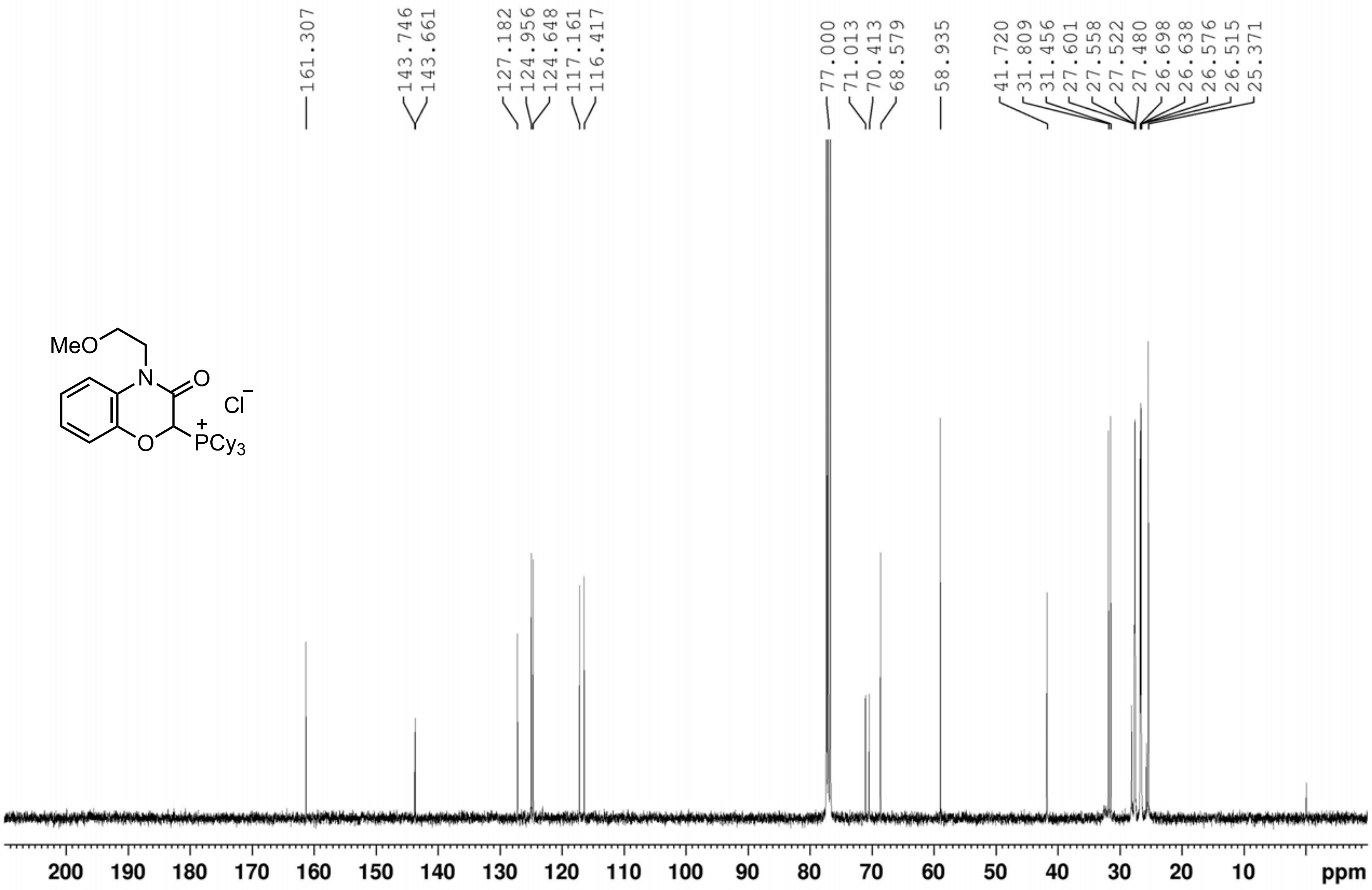

^{13}C NMR (100.6 MHz, $CDCl_3$) spectrum of **2v**

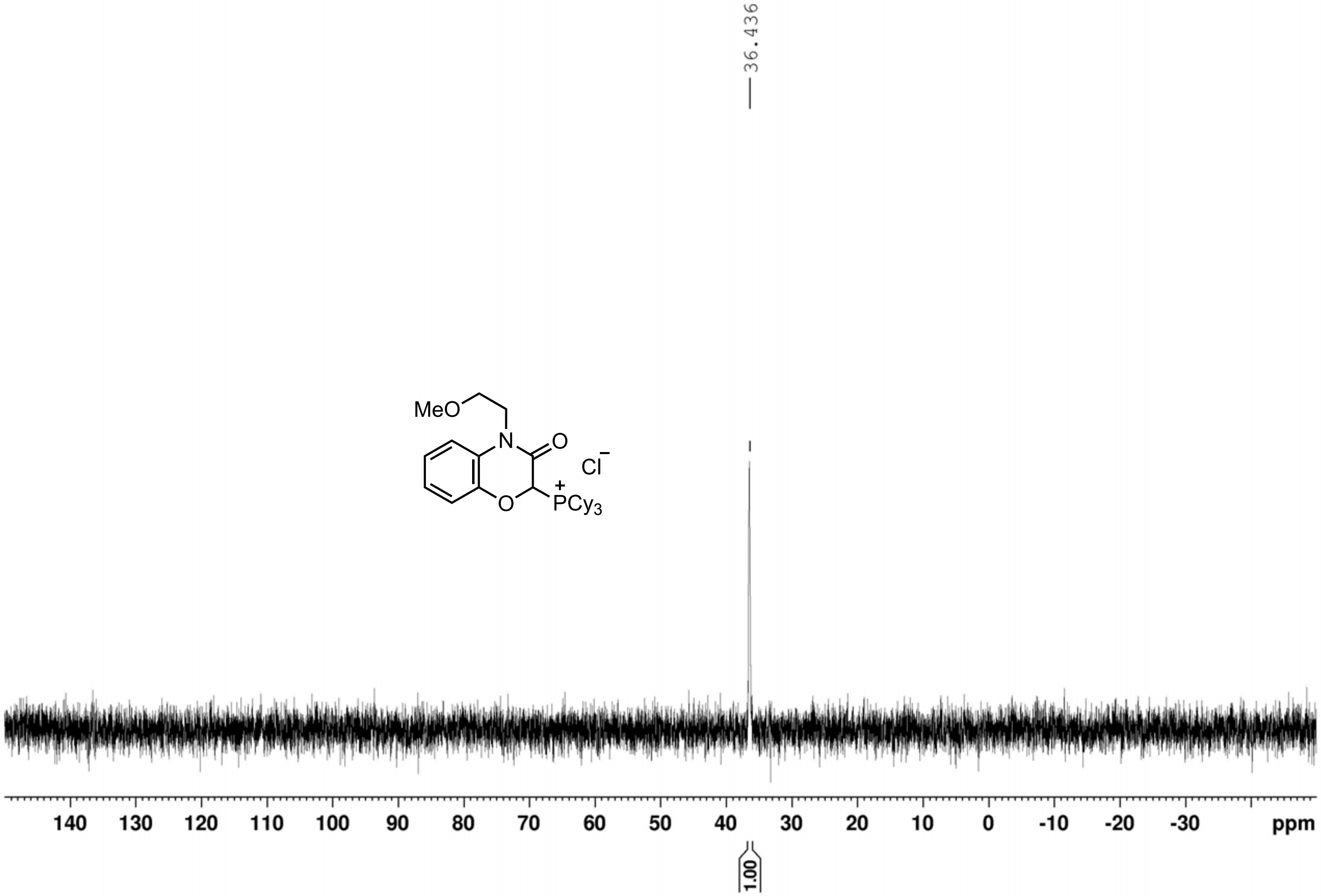

^{31}P NMR (162 MHz, $CDCl_3$) spectrum of **2v**

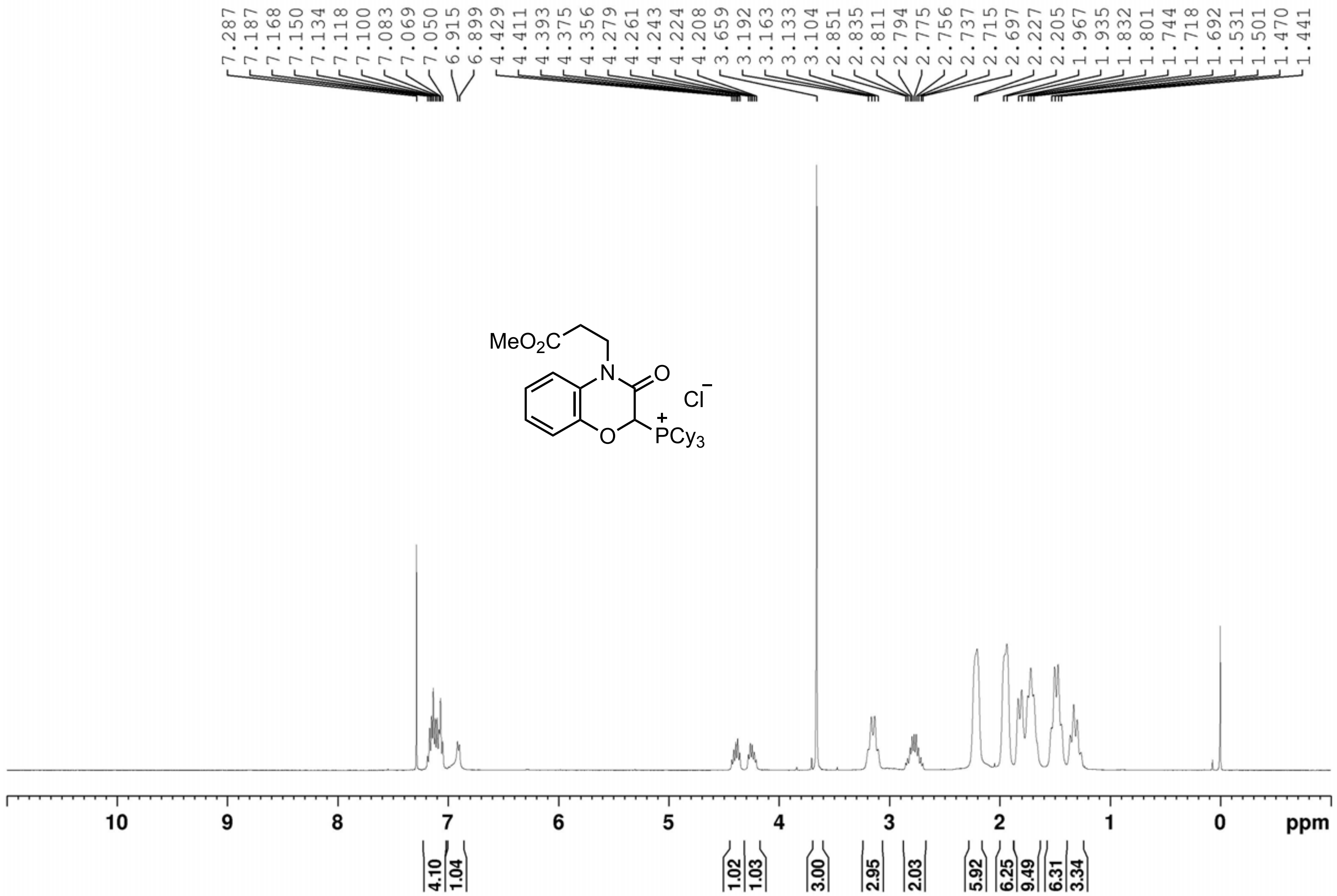

^{1}H NMR (400 MHz, $CDCl_3$) spectrum of **2w**

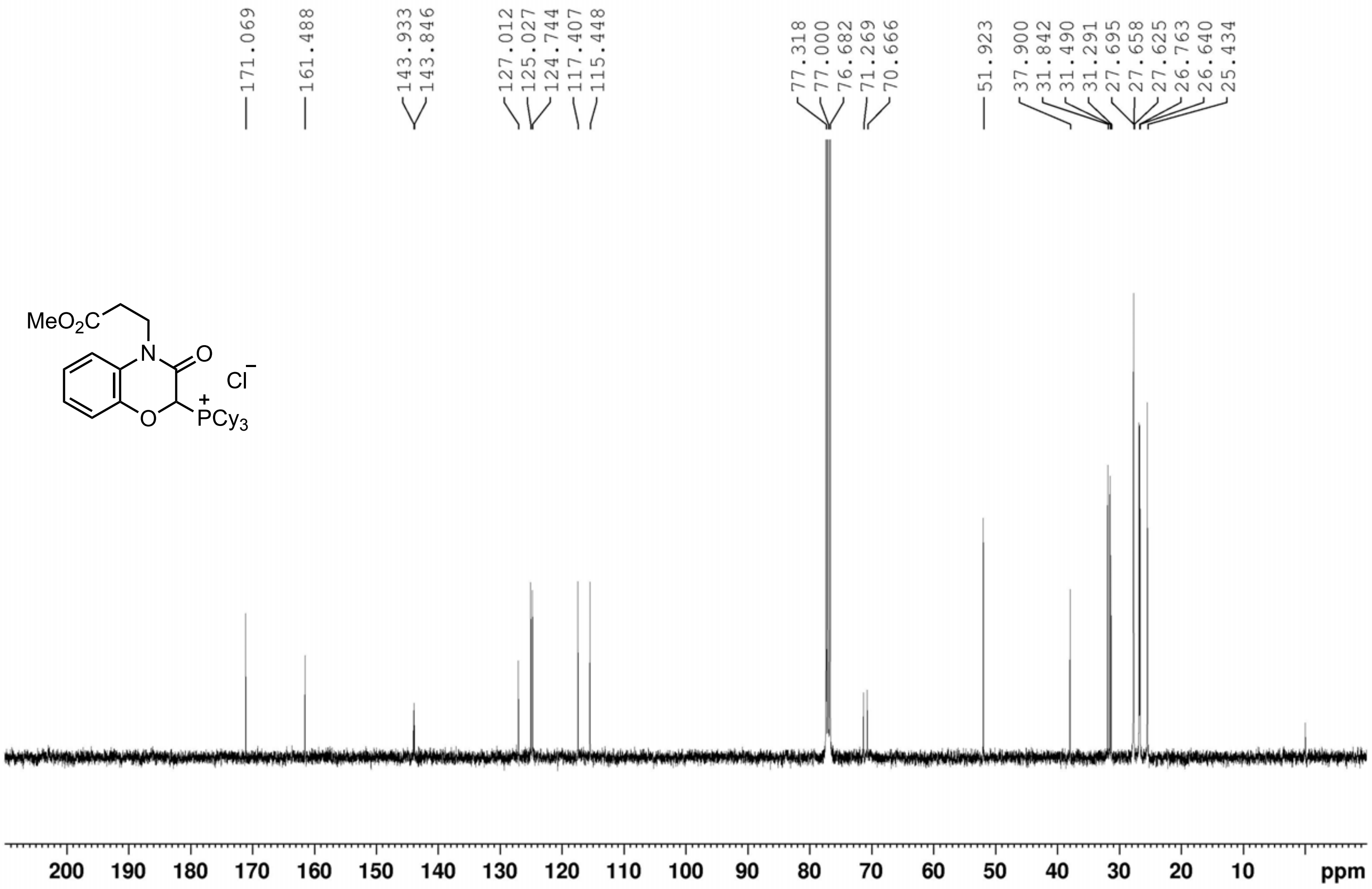

^{13}C NMR (100.6 MHz, $CDCl_3$) spectrum of **2w**

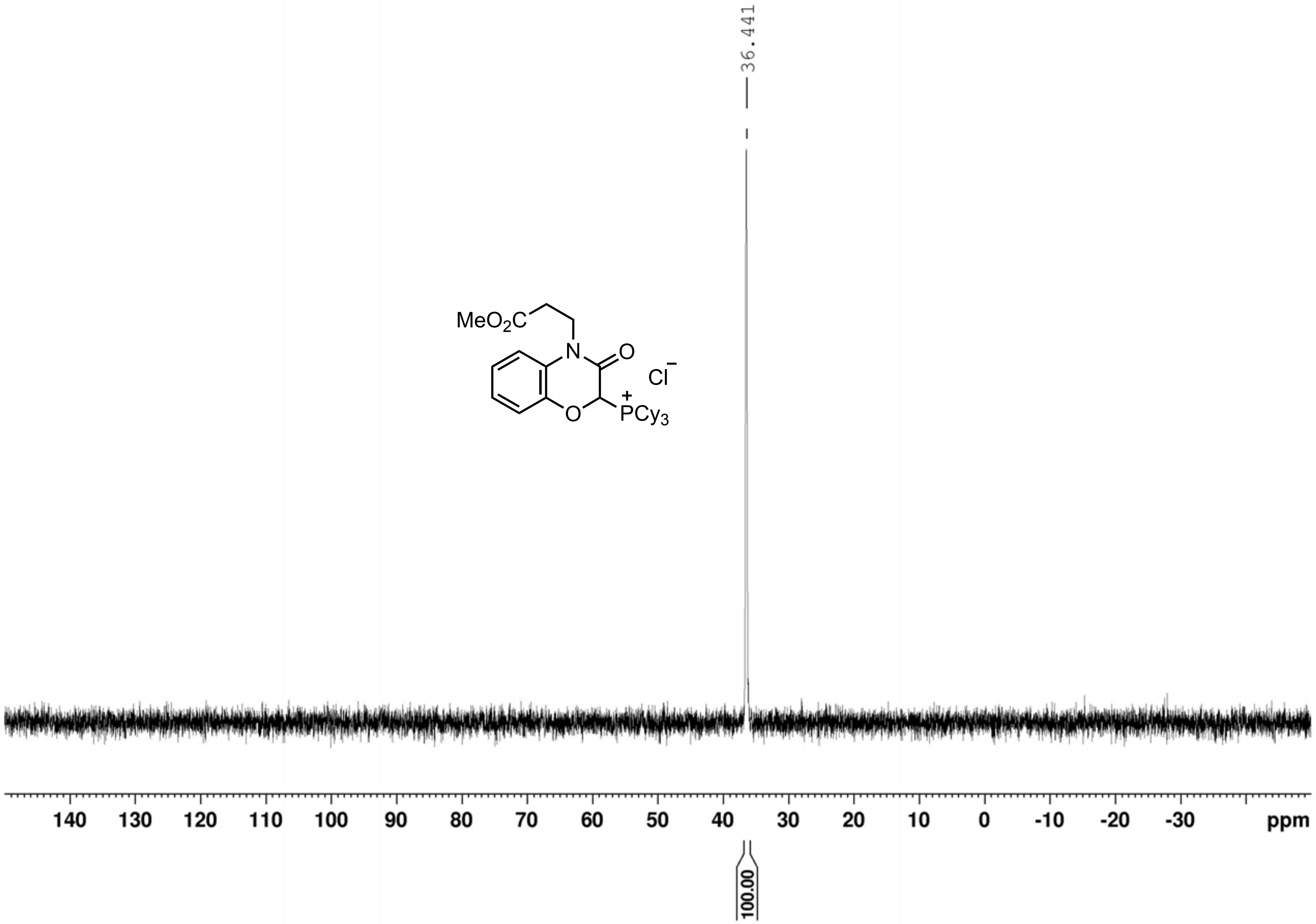

^{31}P NMR (162 MHz, $CDCl_3$) spectrum of **2w**

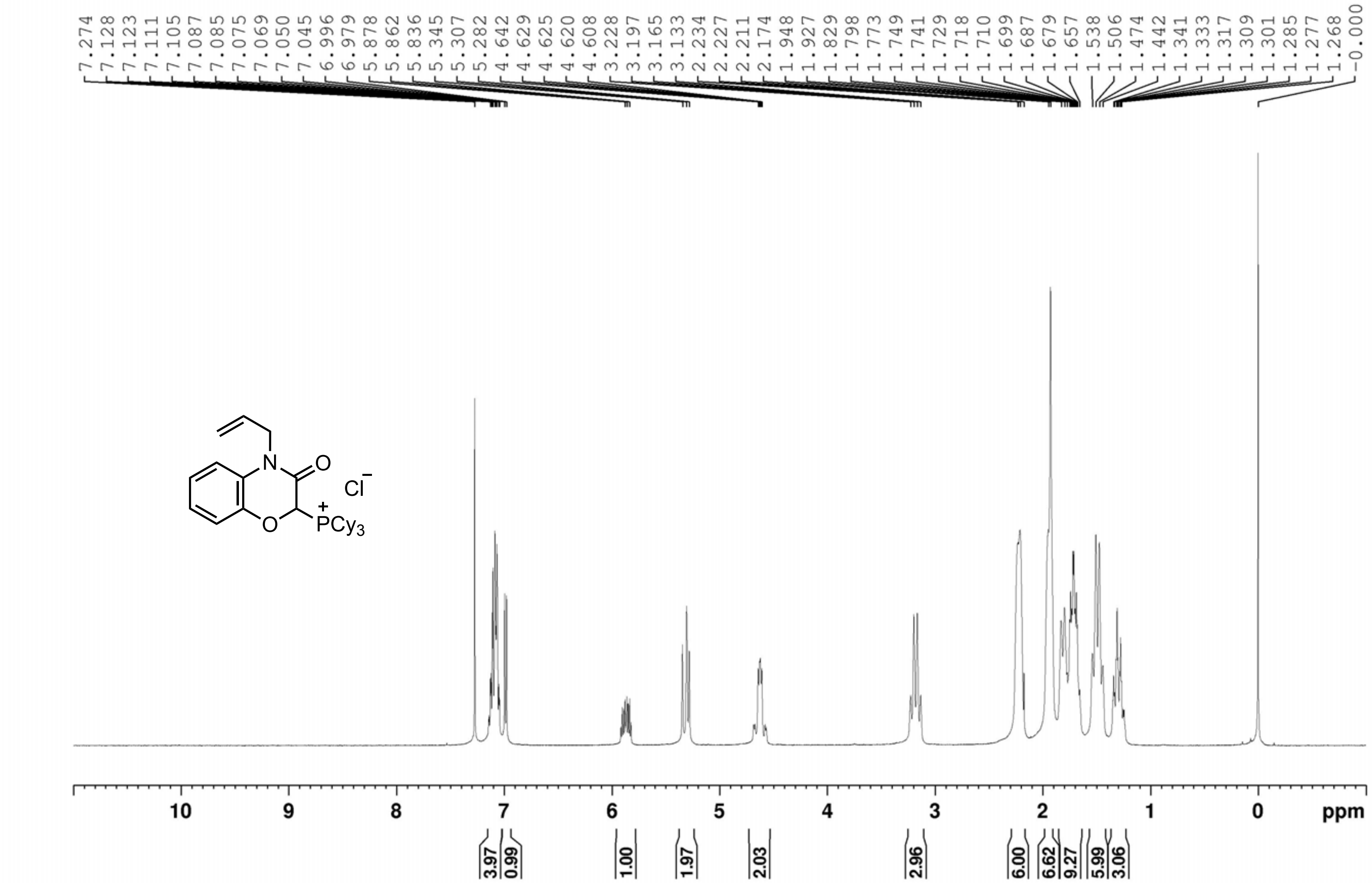

^{1}H NMR (400 MHz, $CDCl_3$) spectrum of **2x**

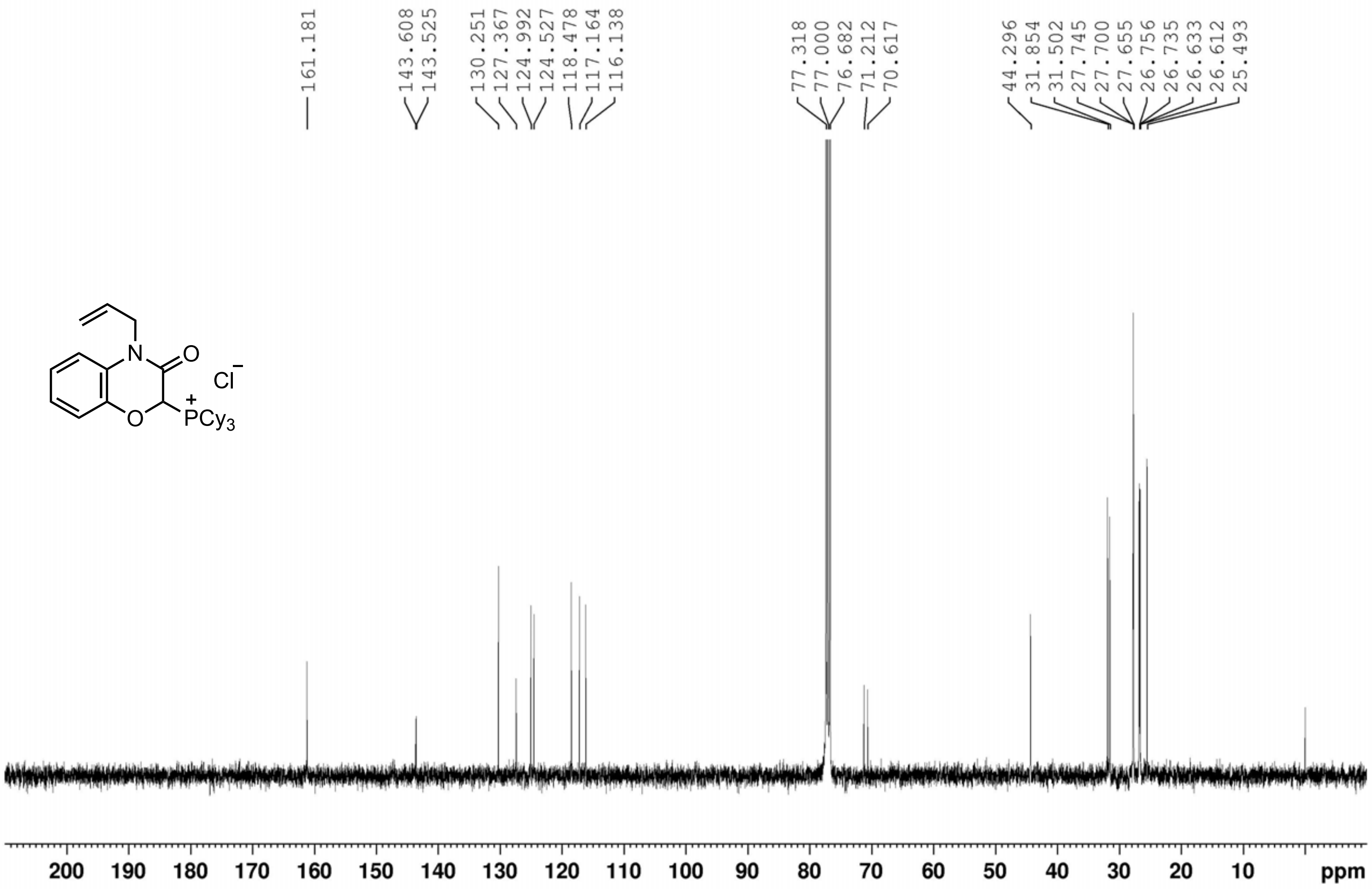

^{13}C NMR (100.6 MHz, $CDCl_3$) spectrum of **2x**

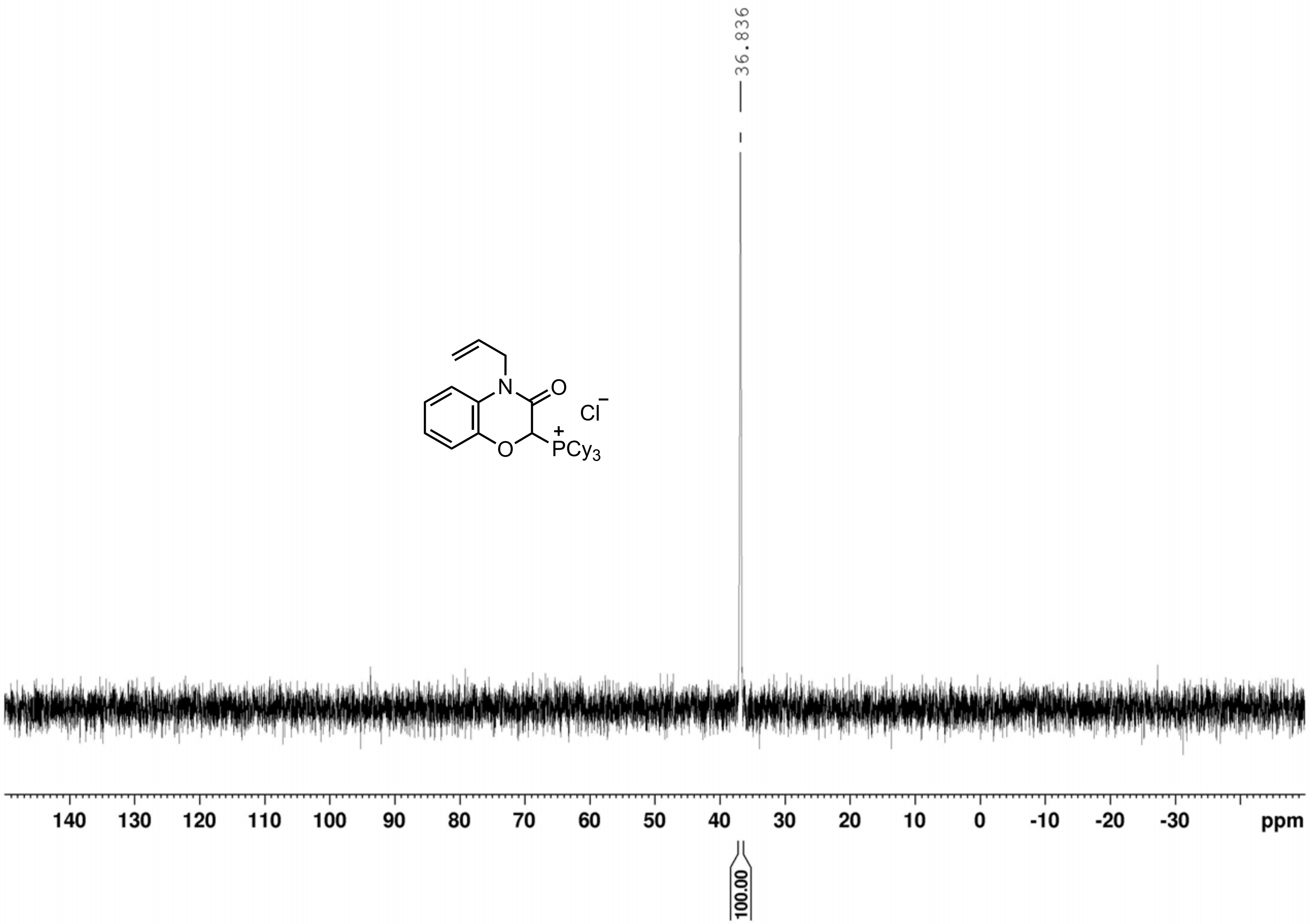

^{31}P NMR (162 MHz, $CDCl_3$) spectrum of **2x**

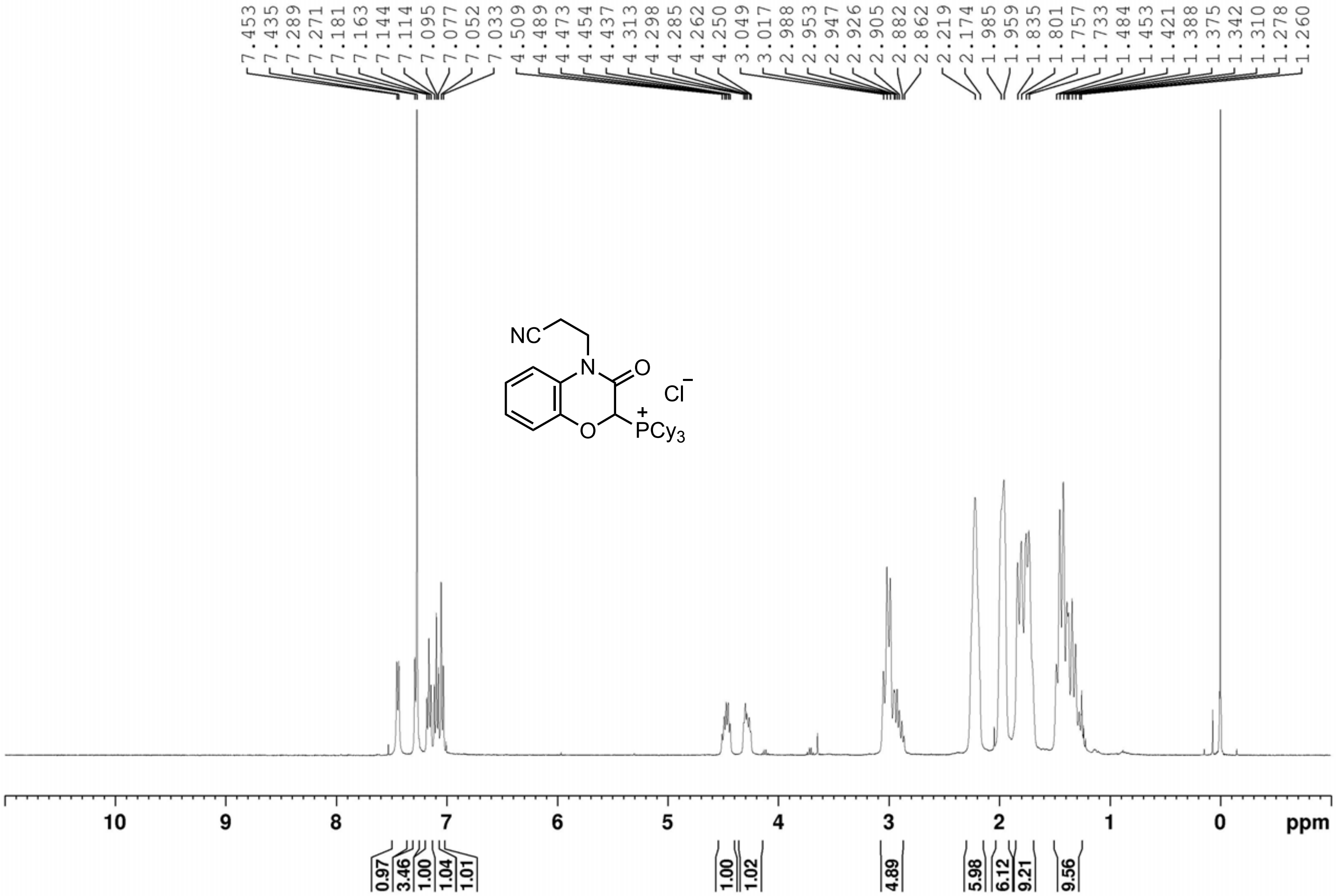

^{1}H NMR (400 MHz, $CDCl_3$) spectrum of **2y**

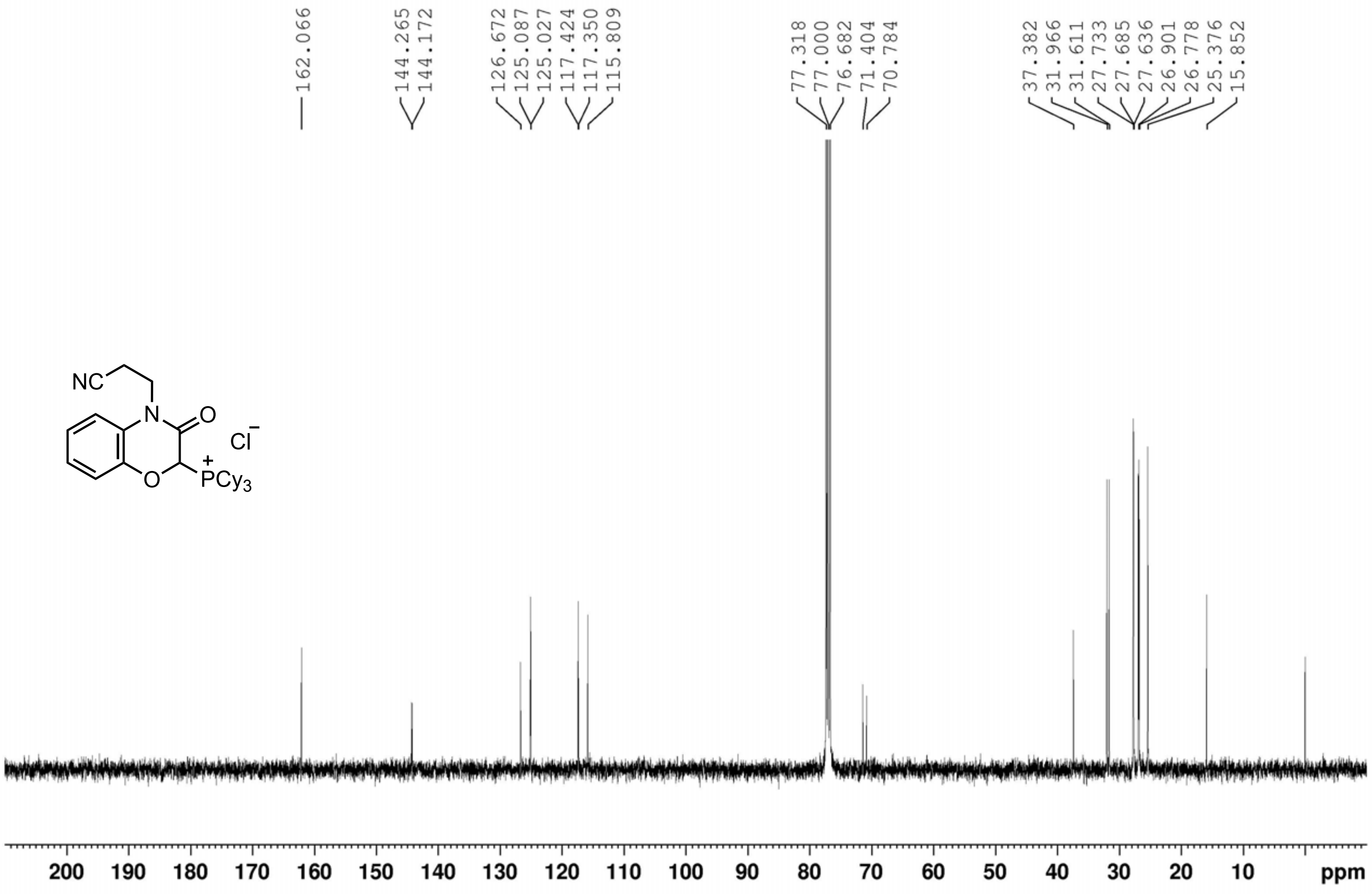

^{13}C NMR (100.6 MHz, $CDCl_3$) spectrum of **2y**

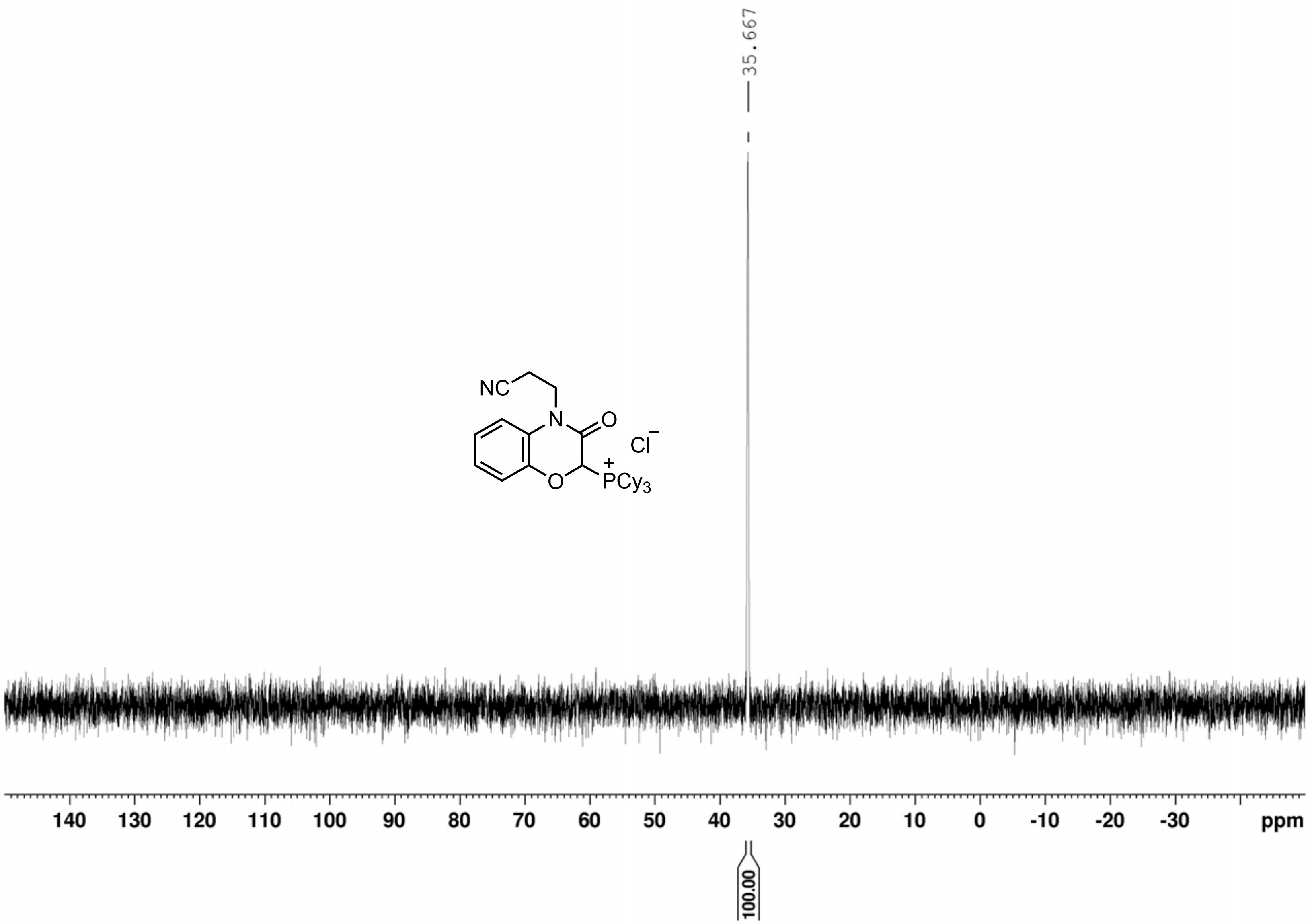

^{31}P NMR (162 MHz, $CDCl_3$) spectrum of **2y**

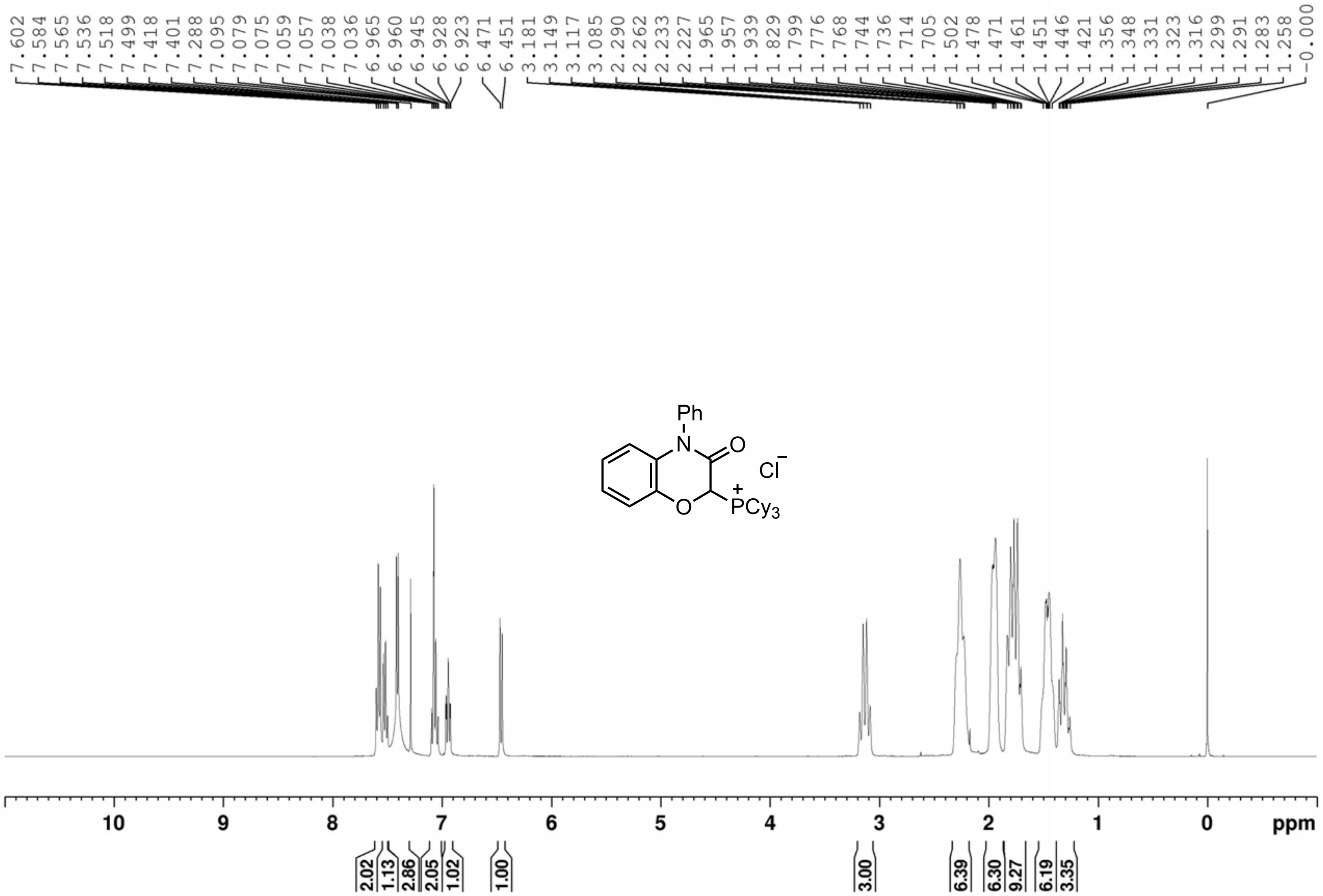

^{1}H NMR (400 MHz, $CDCl_3$) spectrum of **2z**

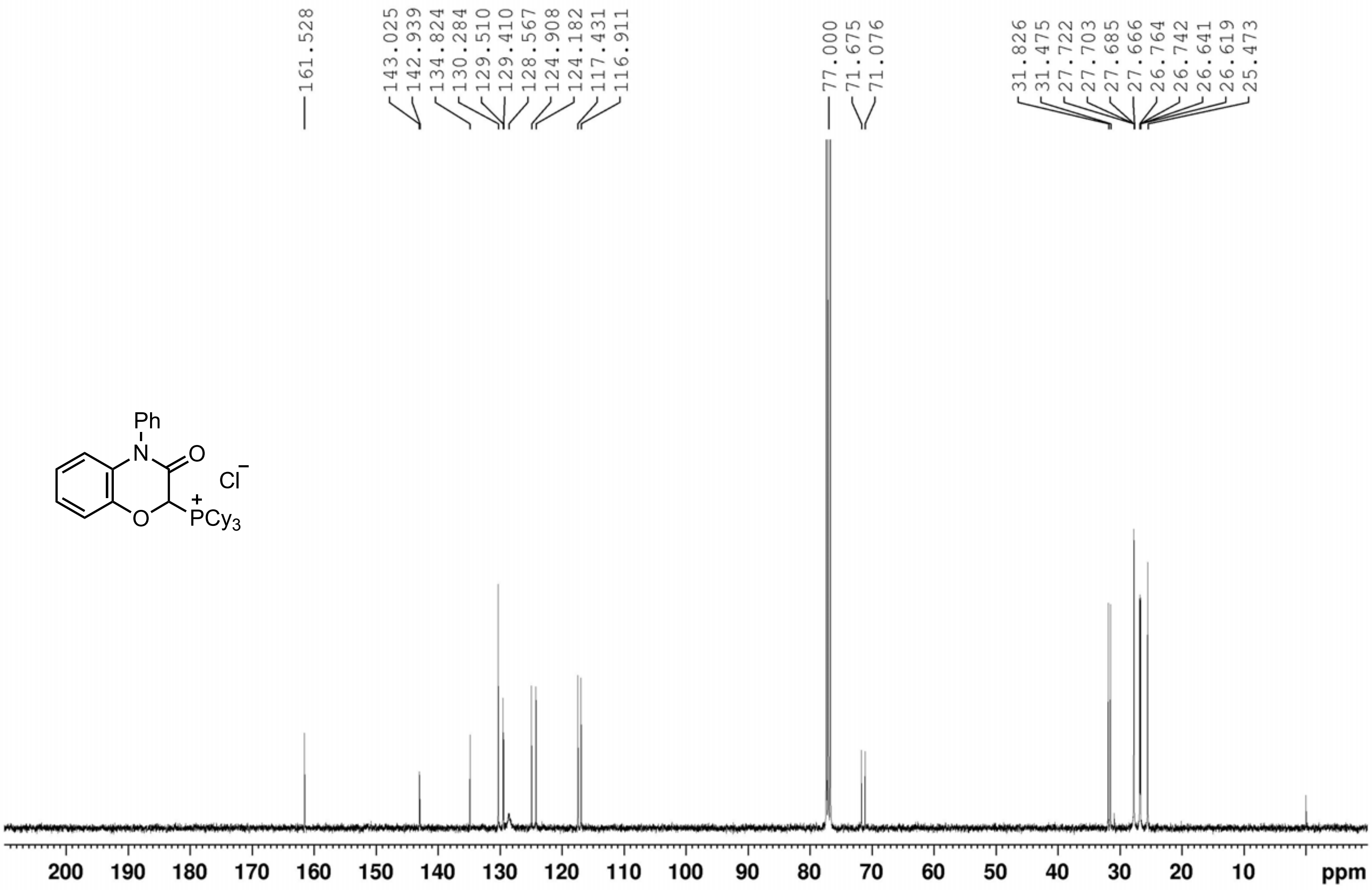

^{13}C NMR (100.6 MHz, $CDCl_3$) spectrum of **2z**

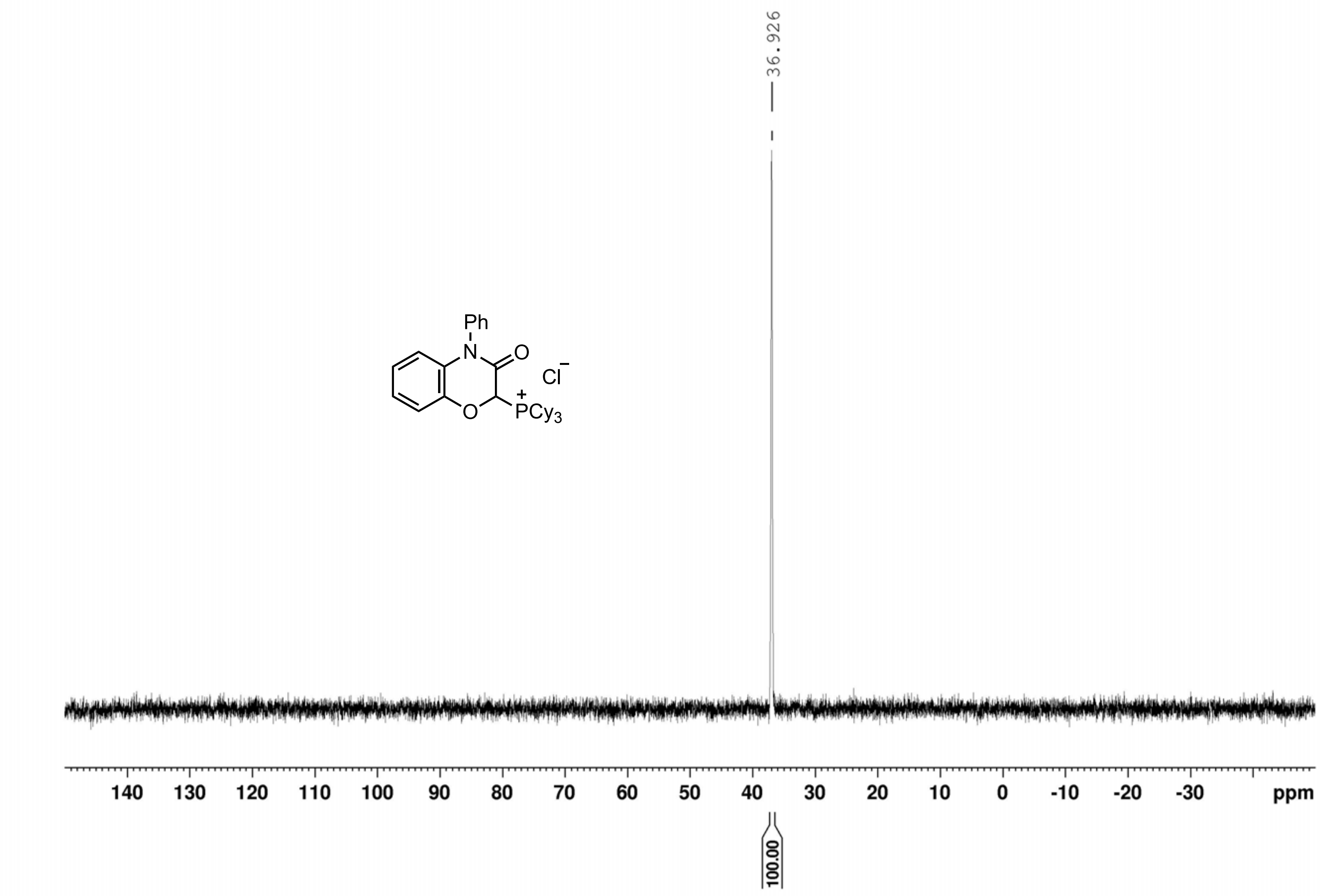

^{31}P NMR (162 MHz, $CDCl_3$) spectrum of **2z**

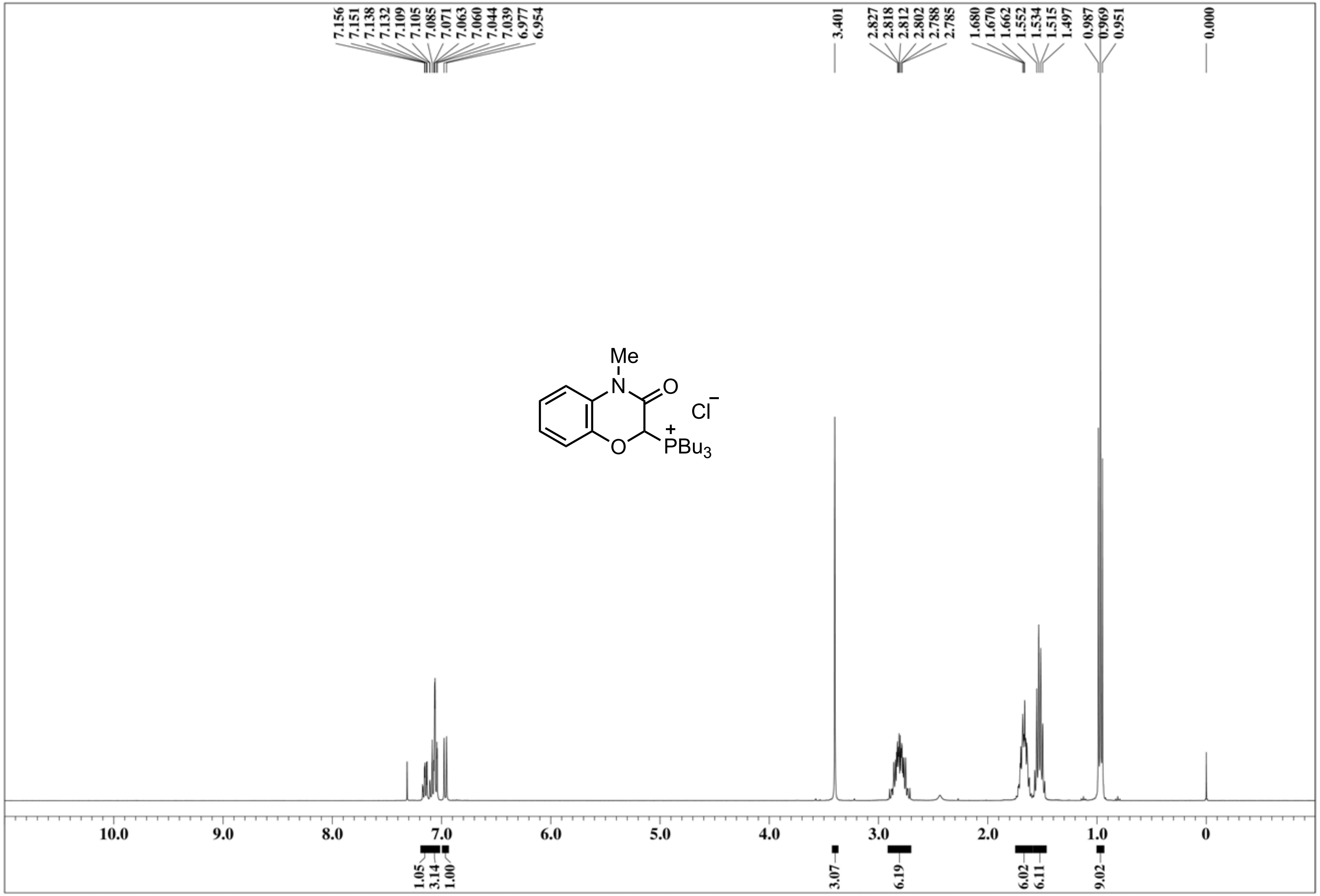

^{1}H NMR (400 MHz, $CDCl_3$) spectrum of **2a’**

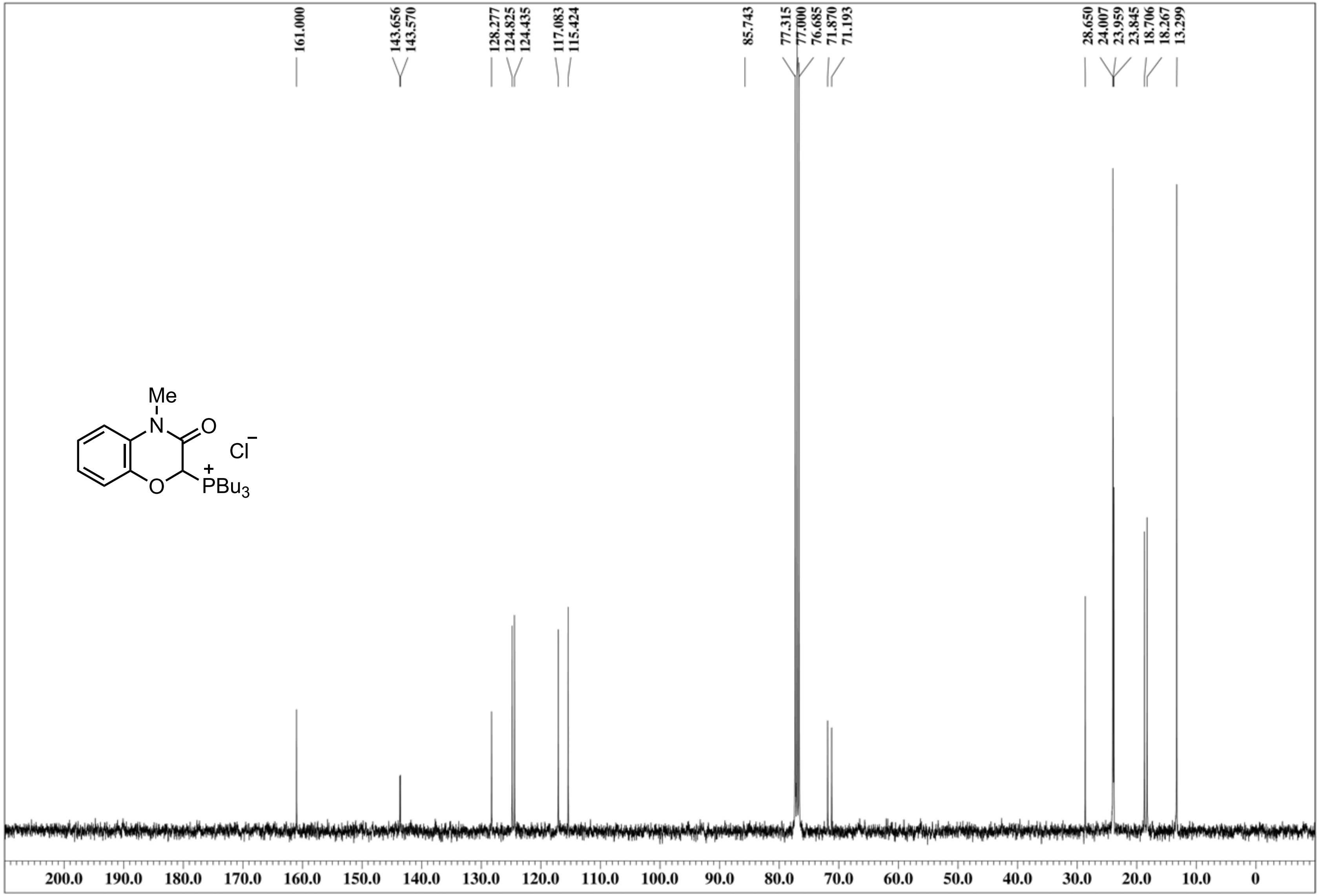

^{13}C NMR (100.6 MHz, $CDCl_3$) spectrum of **2a'**

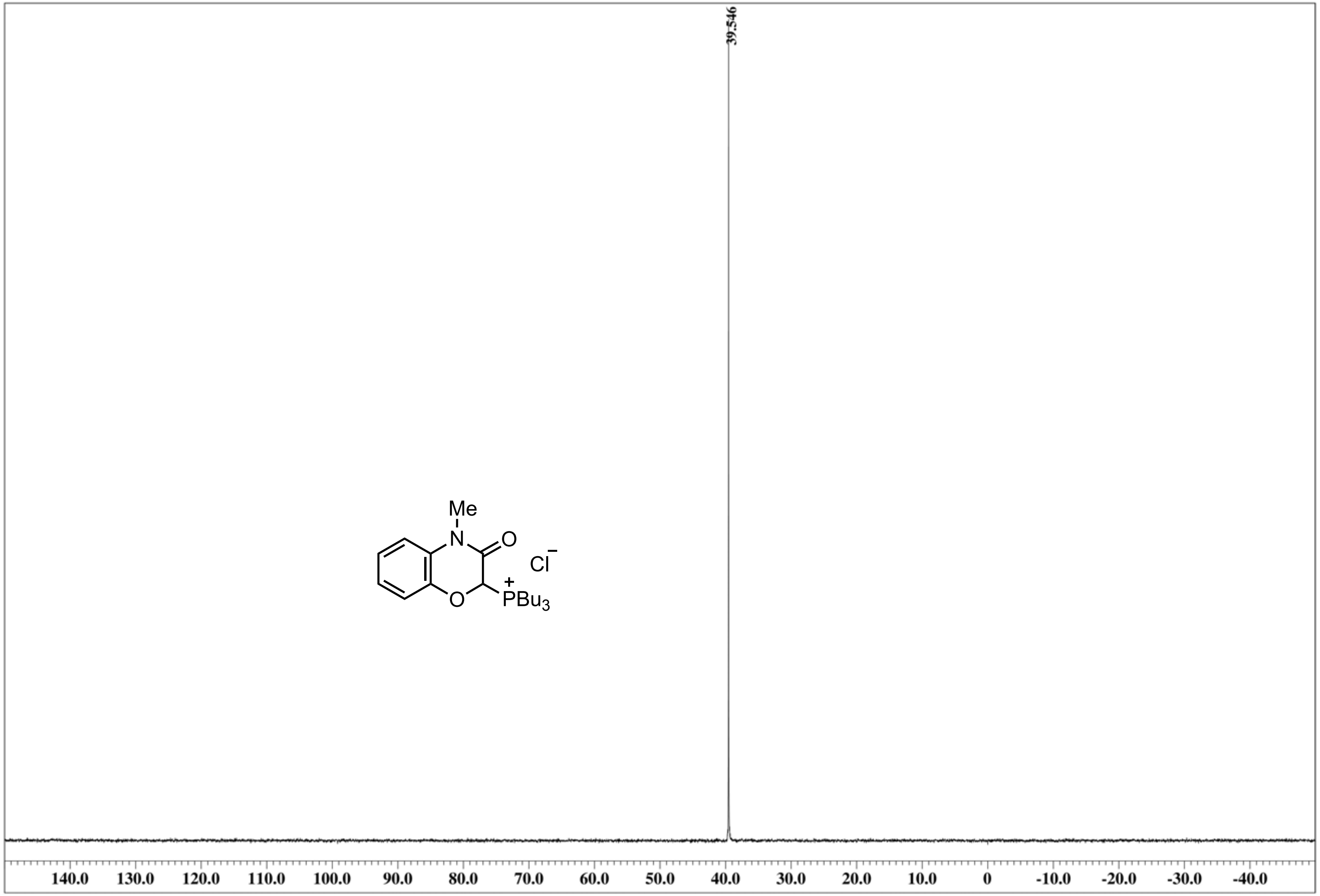

^{31}P NMR (162 MHz, $CDCl_3$) spectrum of **2a'**

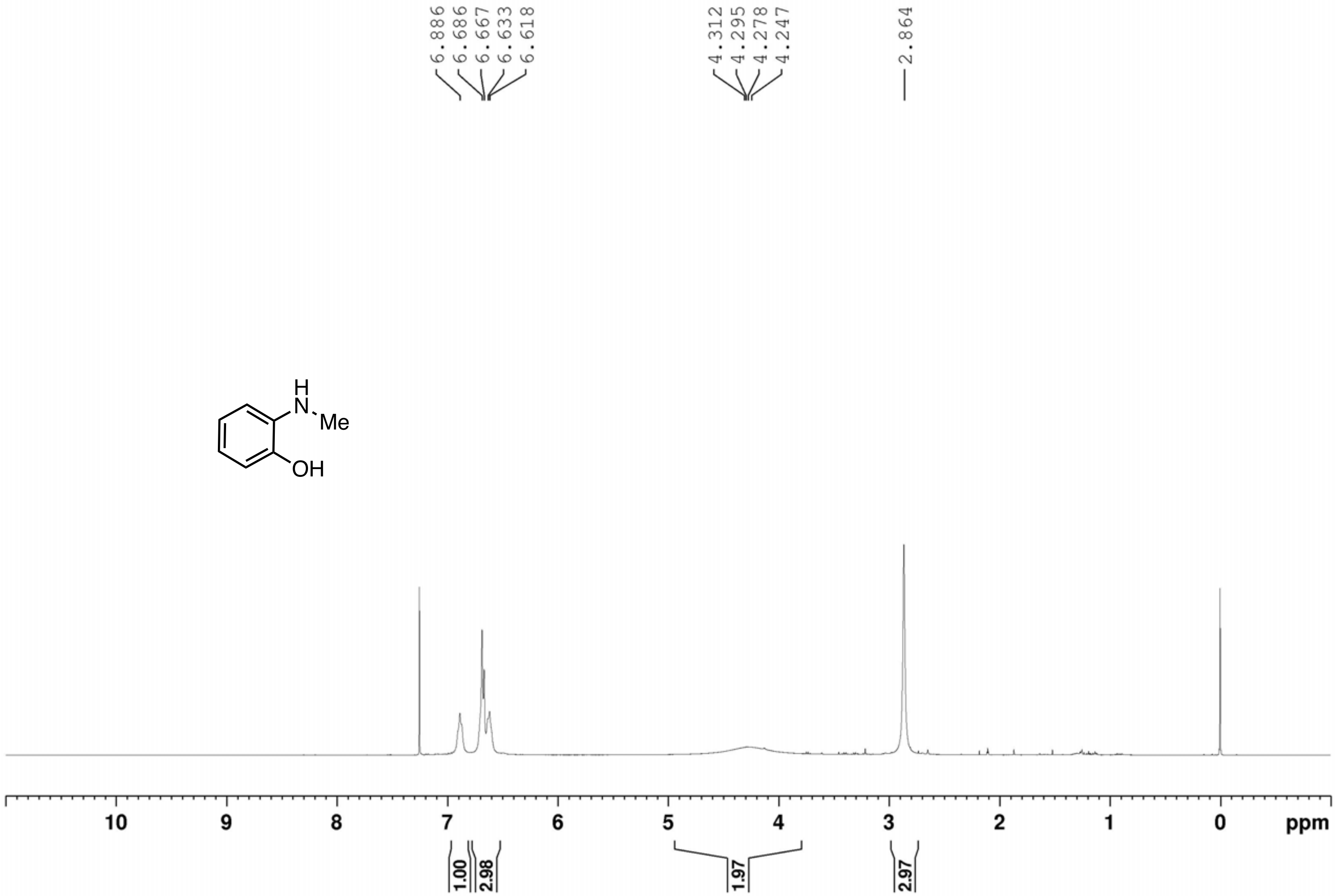

^{1}H NMR (400 MHz, $CDCl_3$) spectrum of **3a**

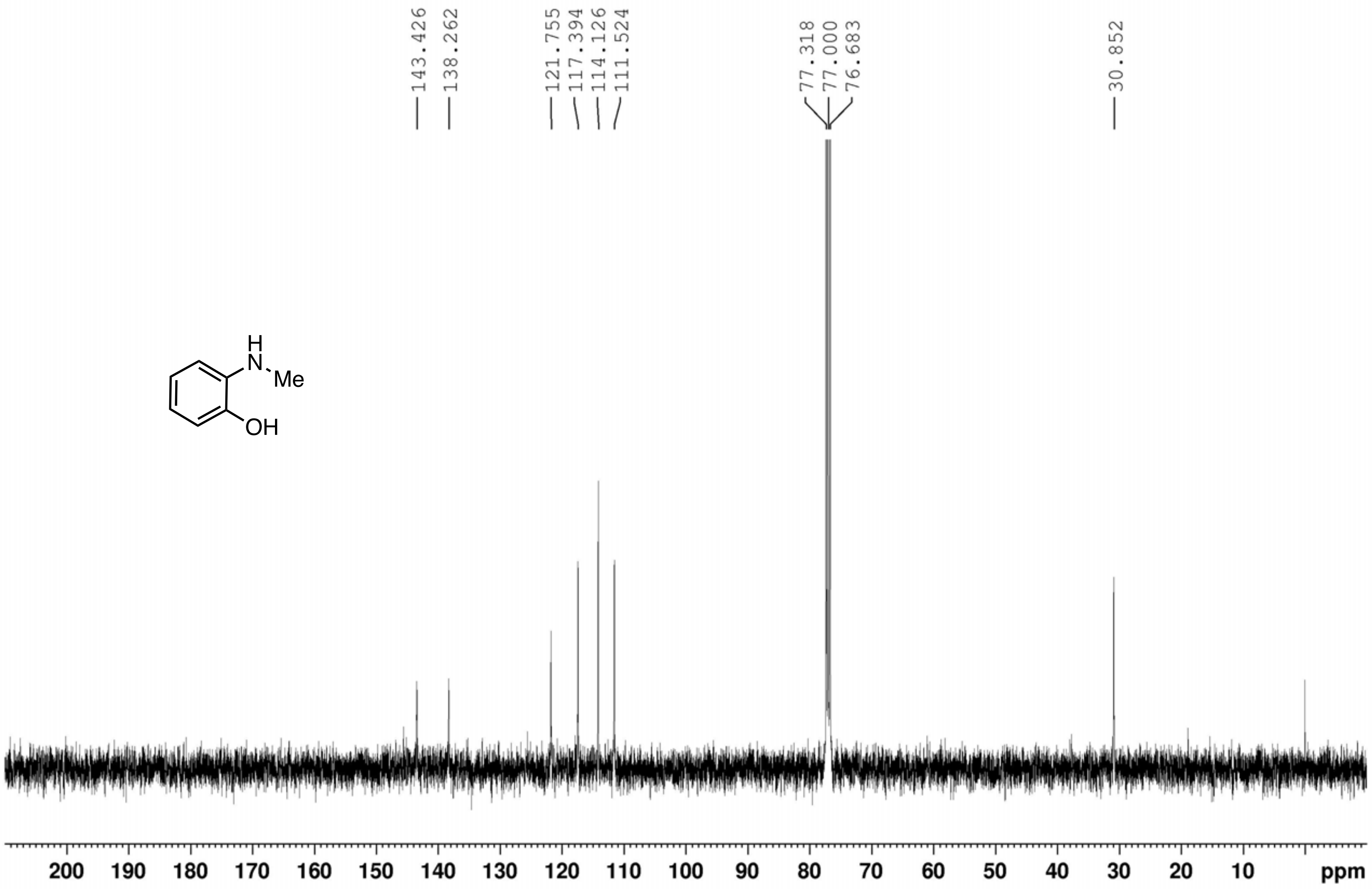

^{13}C NMR (100.6 MHz, $CDCl_3$) spectrum of **3a**

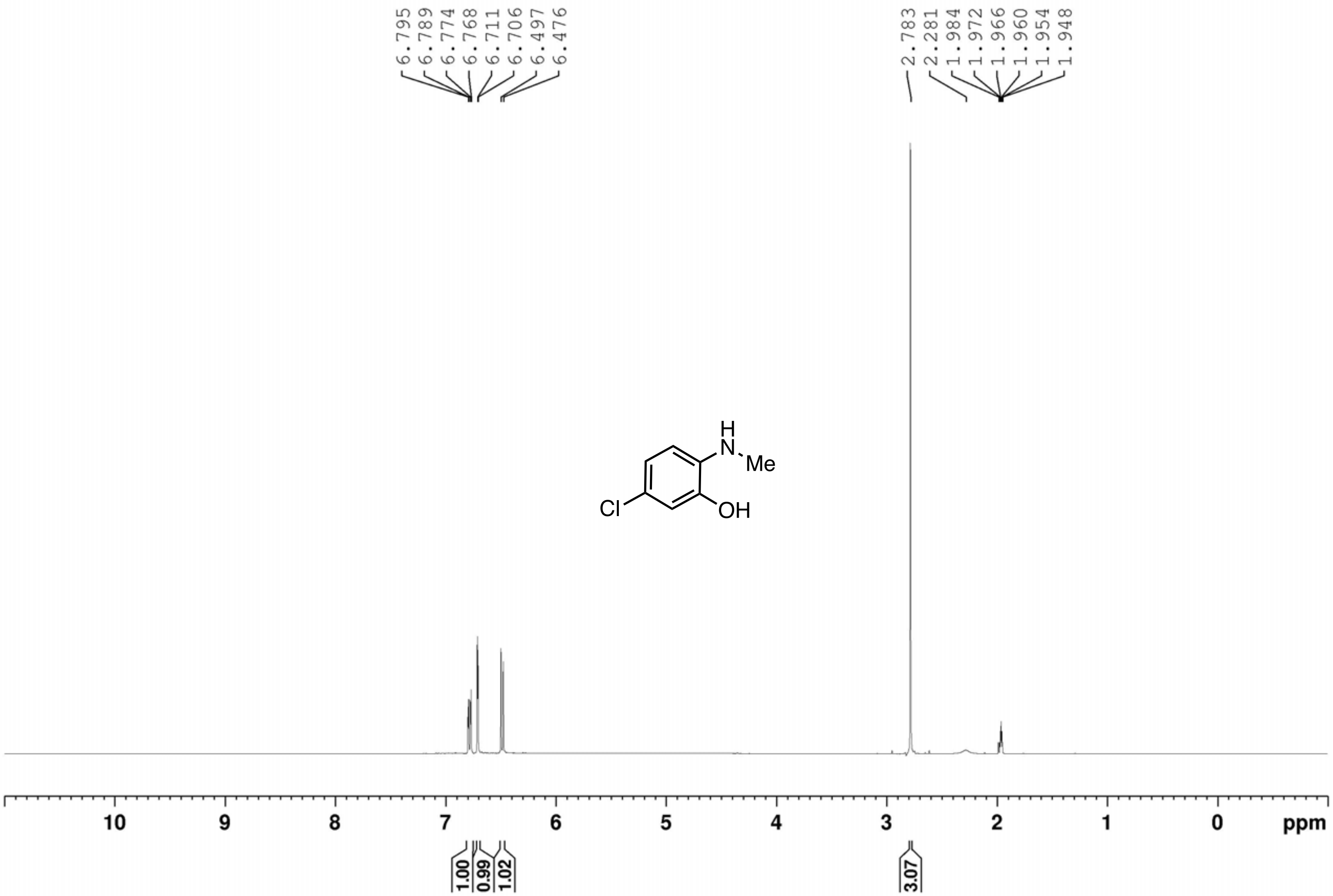

^{1}H NMR (400 MHz, $CDCl_3$) spectrum of **3f**

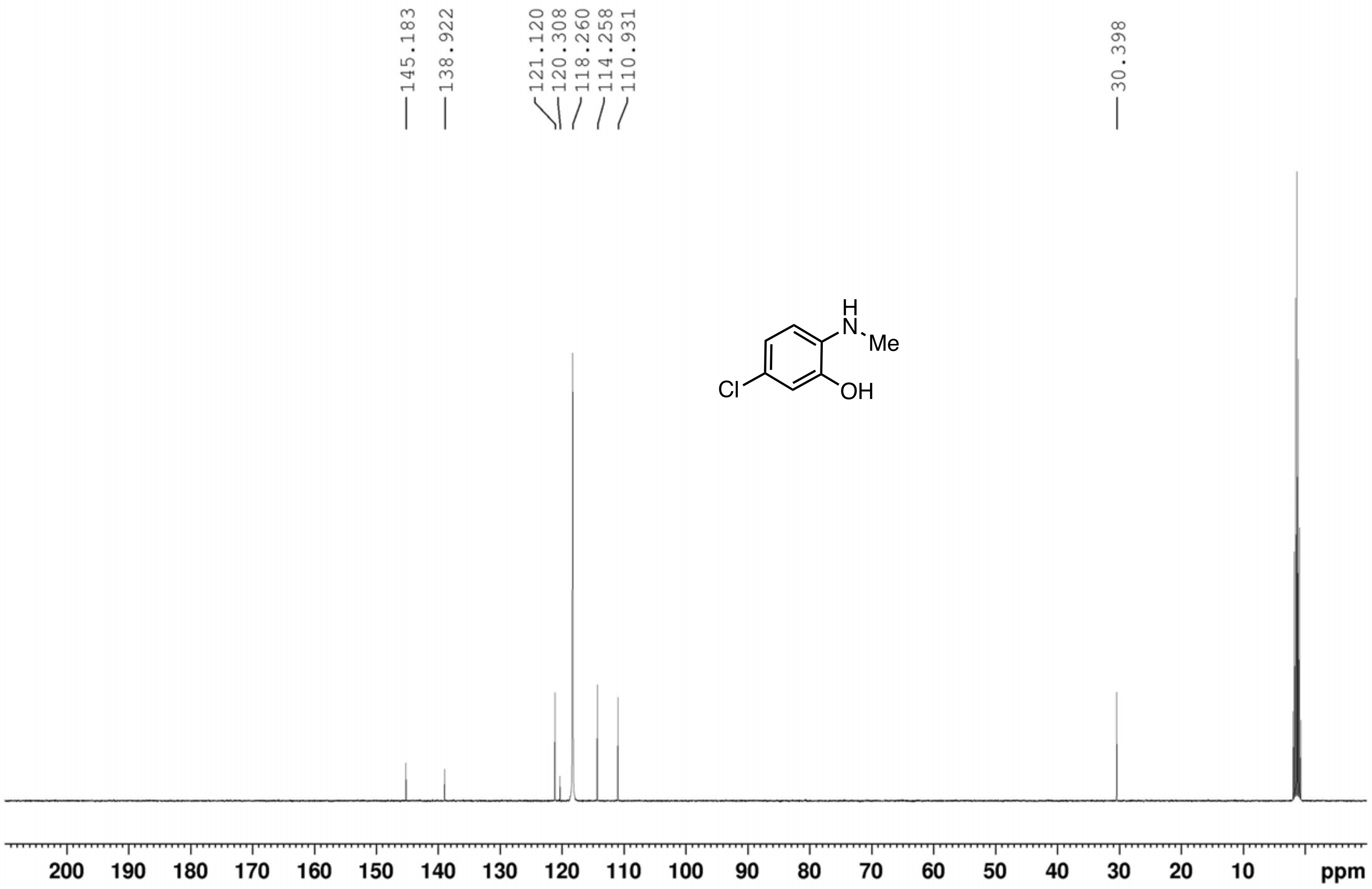

^{13}C NMR (100.6 MHz, $CDCl_3$) spectrum of **3f**

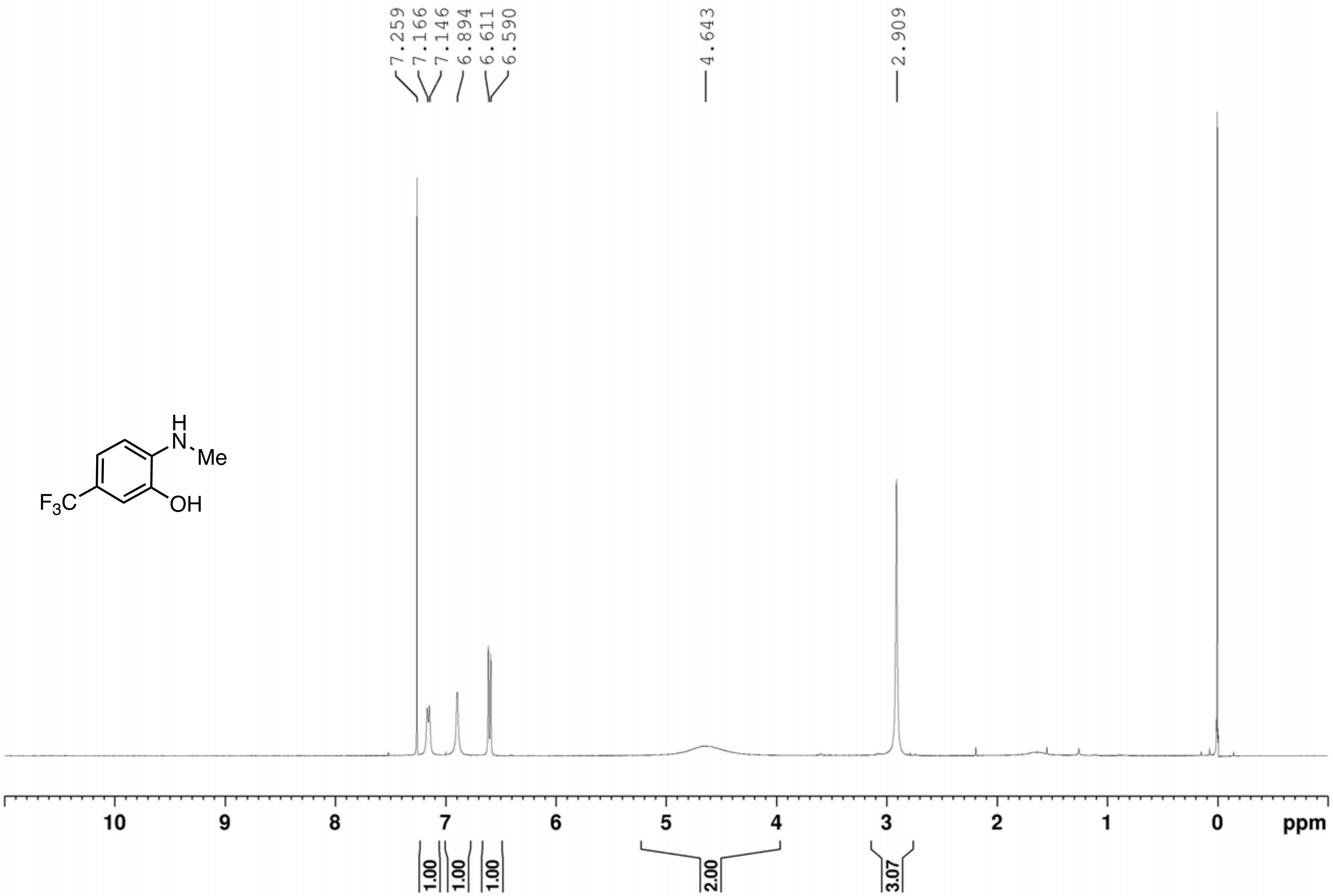

^{1}H NMR (400 MHz, $CDCl_3$) spectrum of **3i**

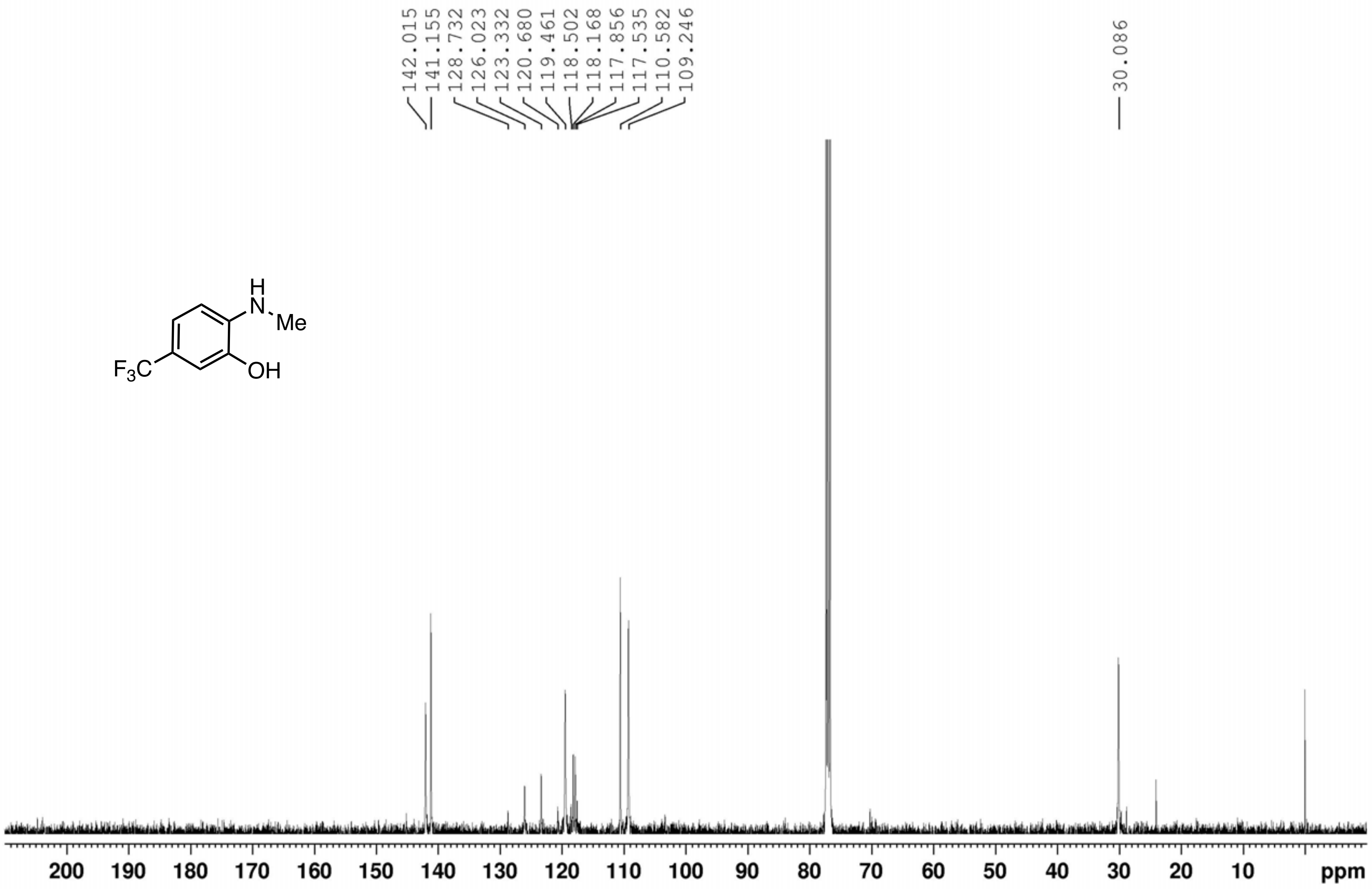

^{13}C NMR (100.6 MHz, CDCl$_3$) spectrum of **3i**

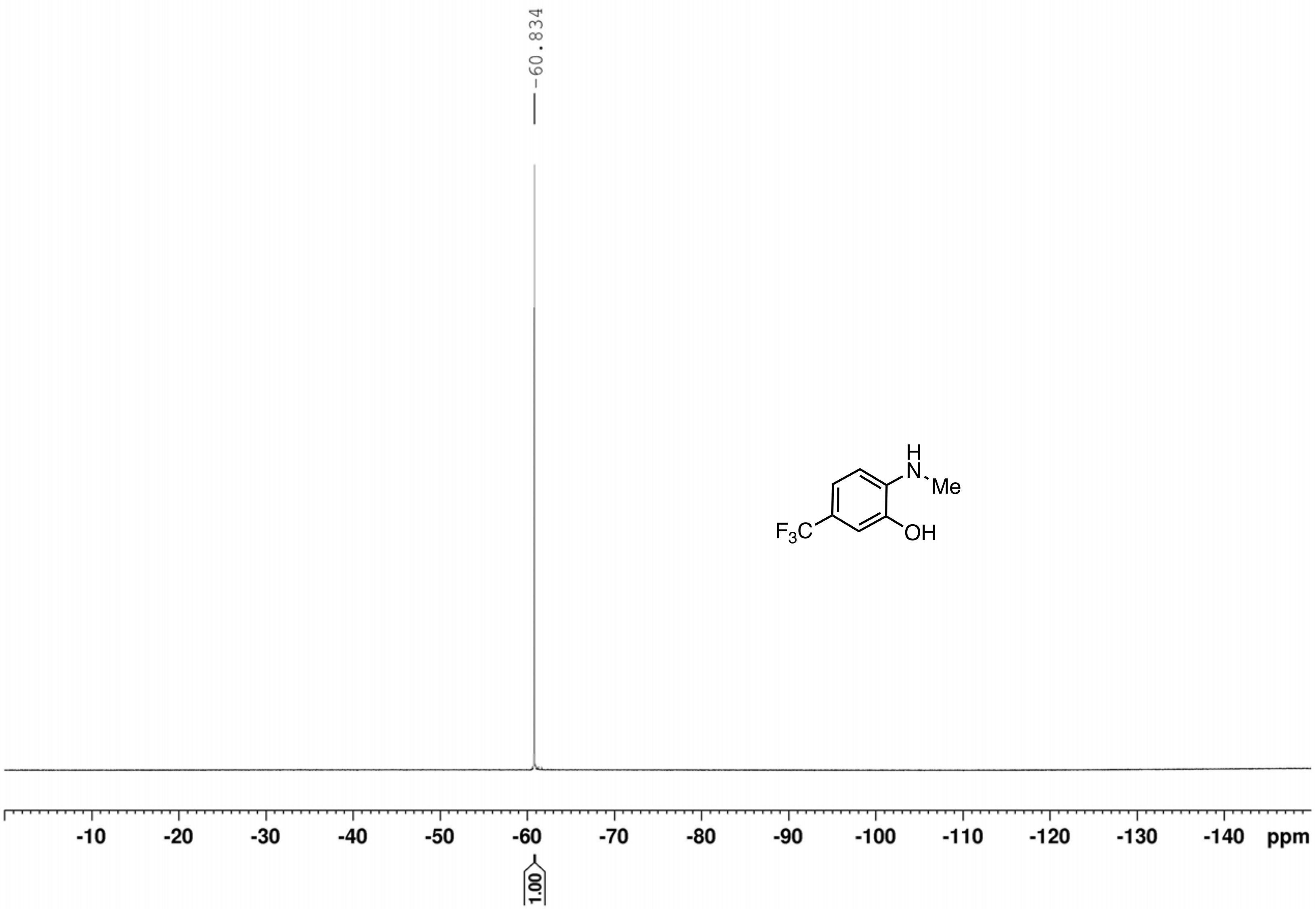

^{19}F NMR (376.5 MHz, $CDCl_3$) spectrum of **3i**

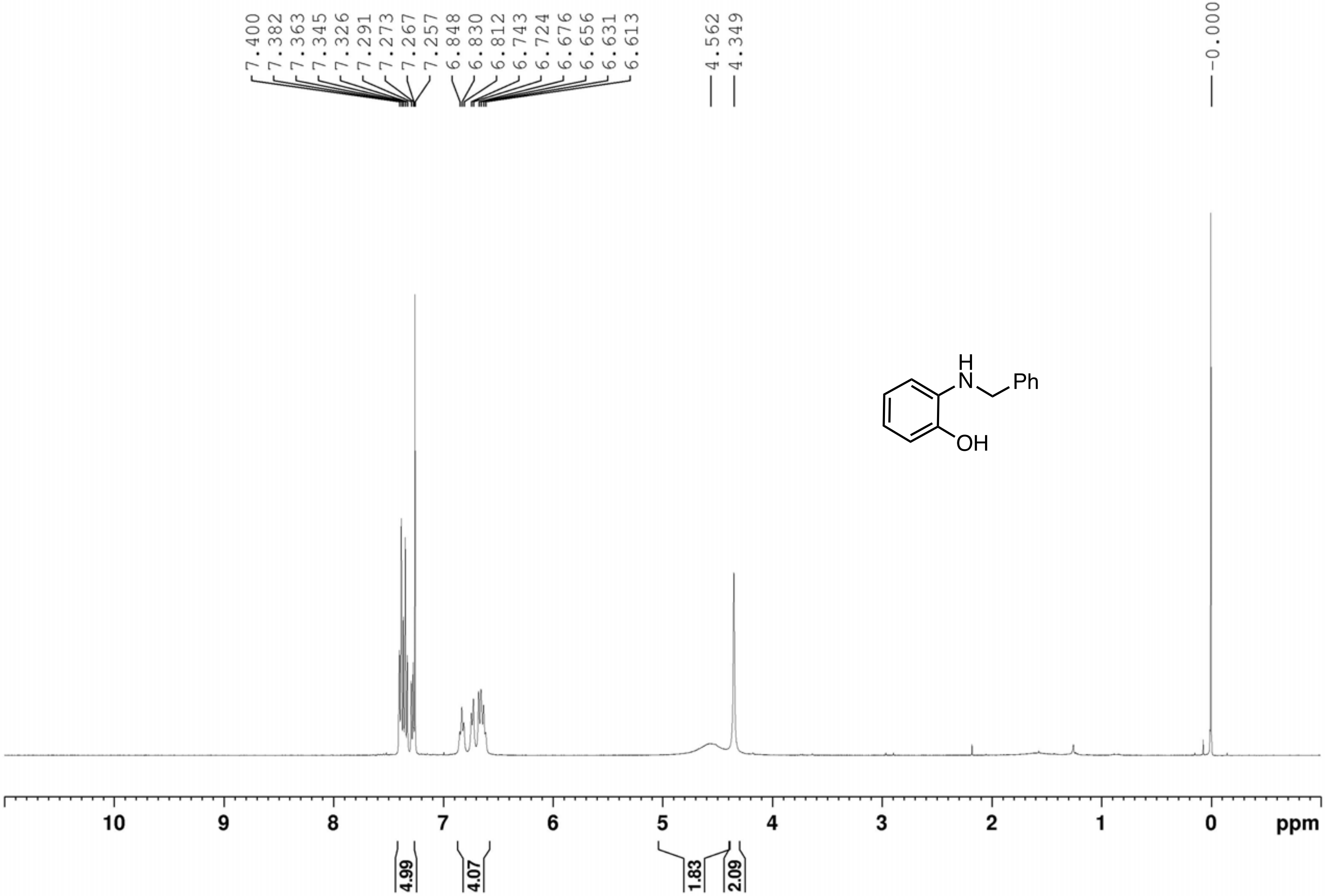

^{1}H NMR (400 MHz, $CDCl_3$) spectrum of **3s**

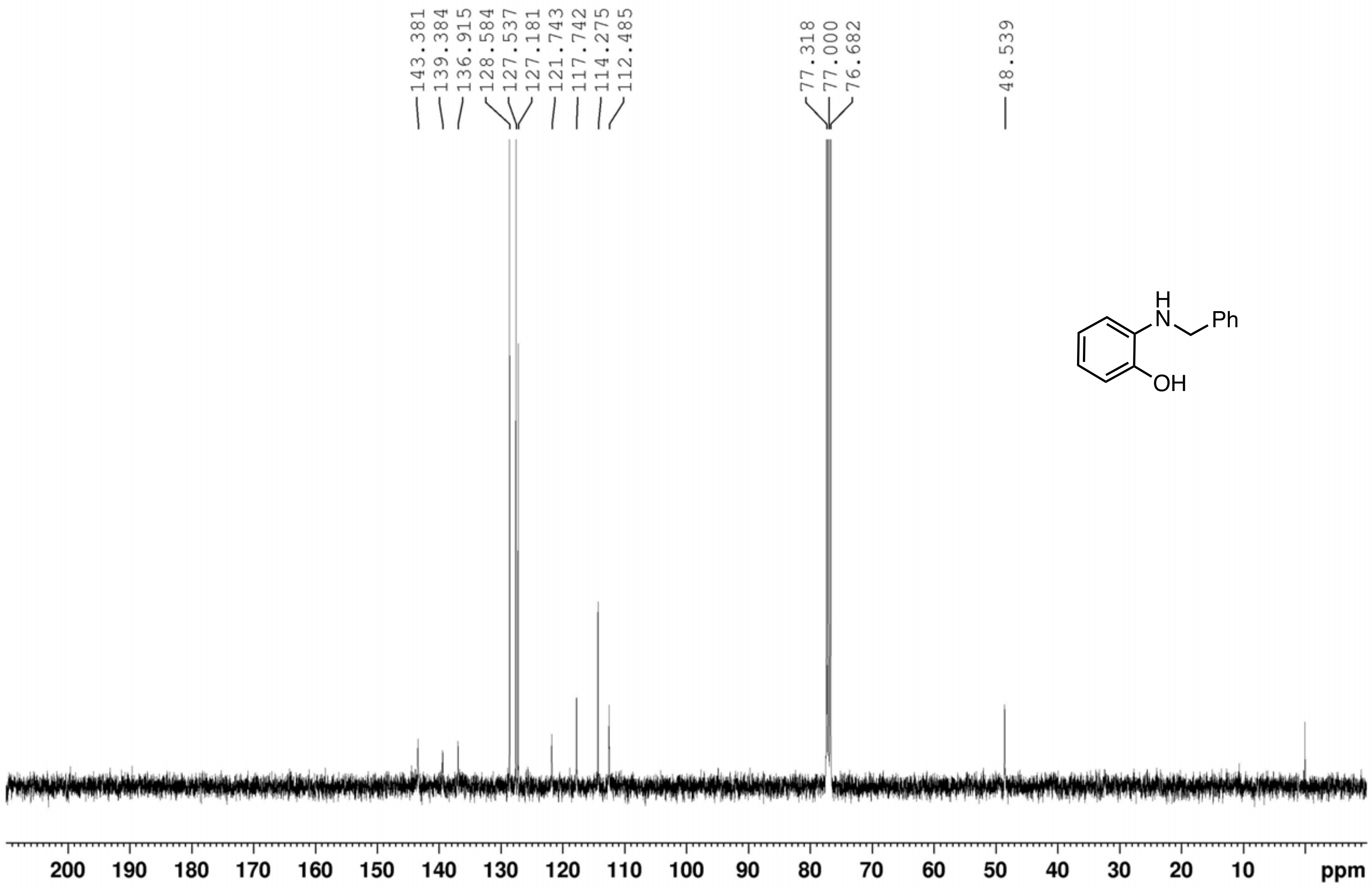

^{13}C NMR (100.6 MHz, $CDCl_3$) spectrum of **3s**

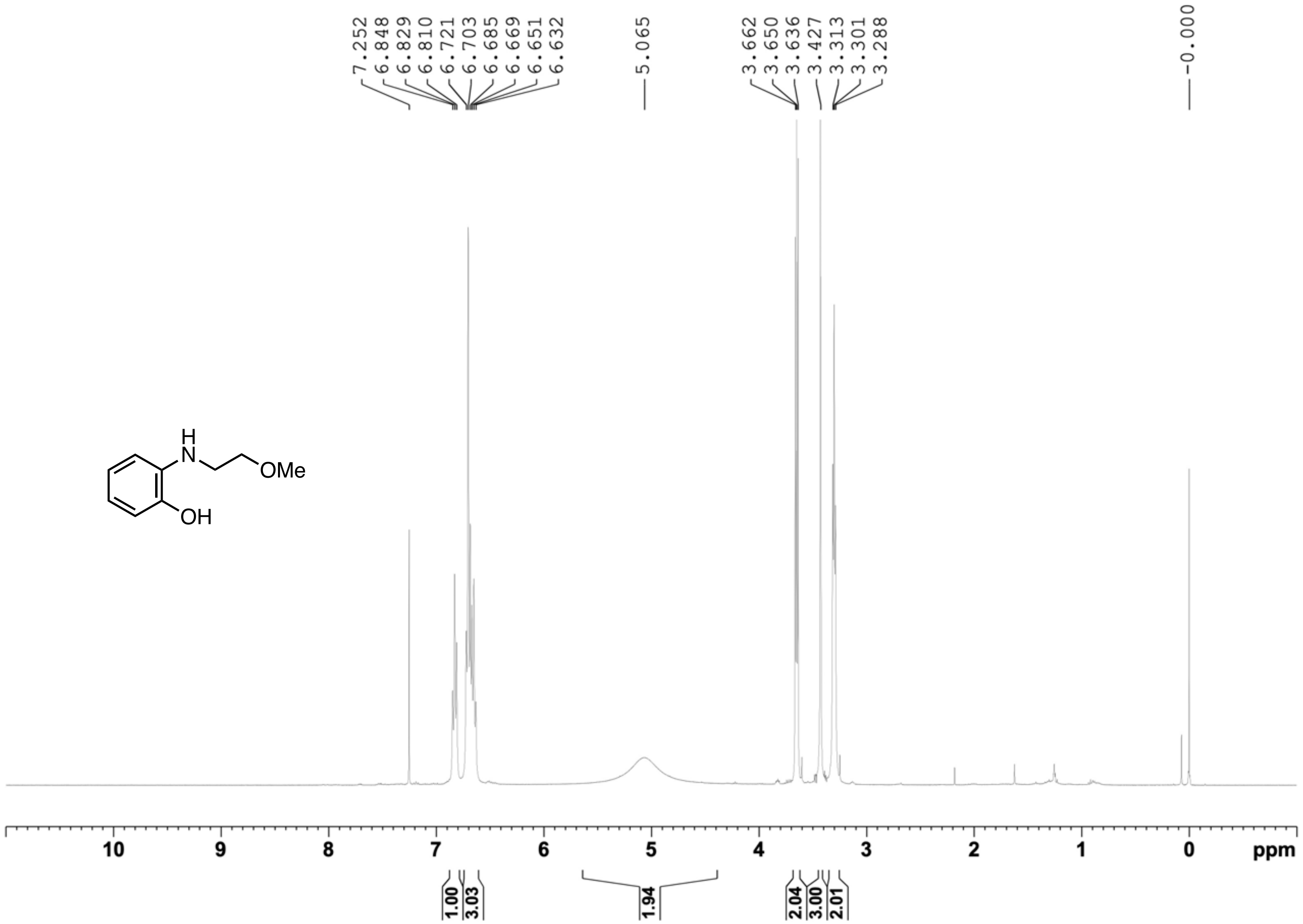

^{1}H NMR (400 MHz, $CDCl_3$) spectrum of **3v**

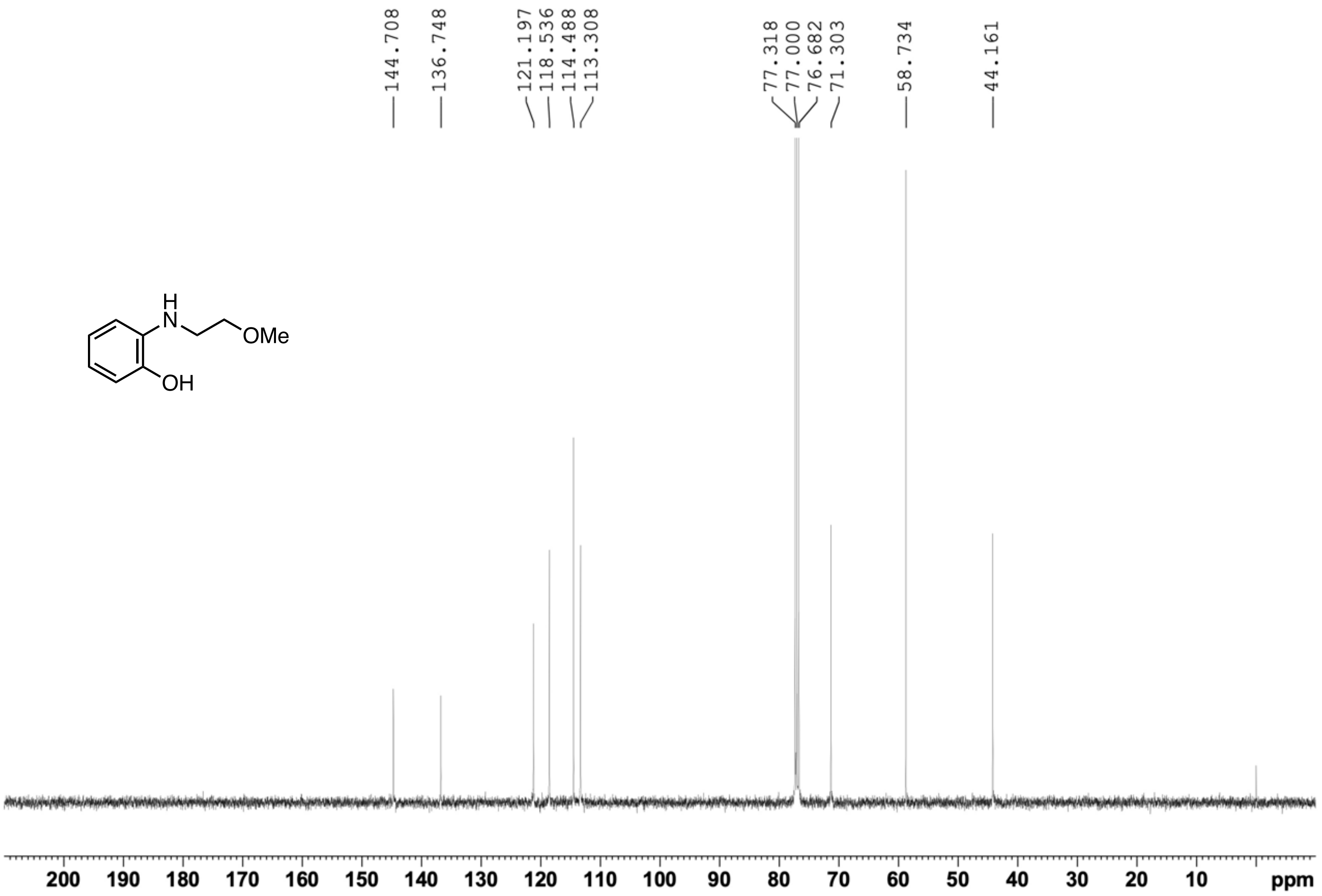

^{13}C NMR (100.6 MHz, $CDCl_3$) spectrum of **3v**

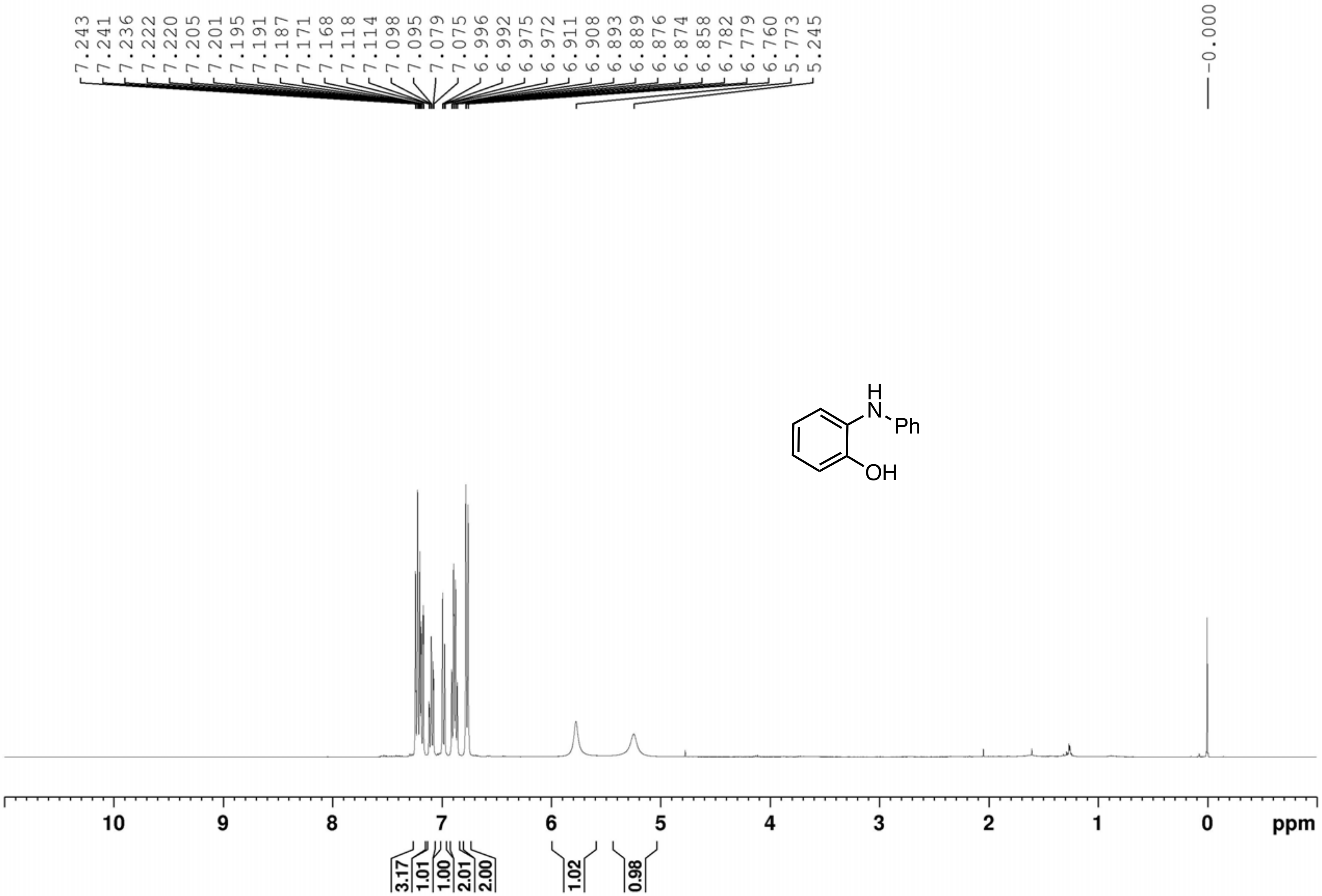

^{1}H NMR (400 MHz, $CDCl_3$) spectrum of **3z**

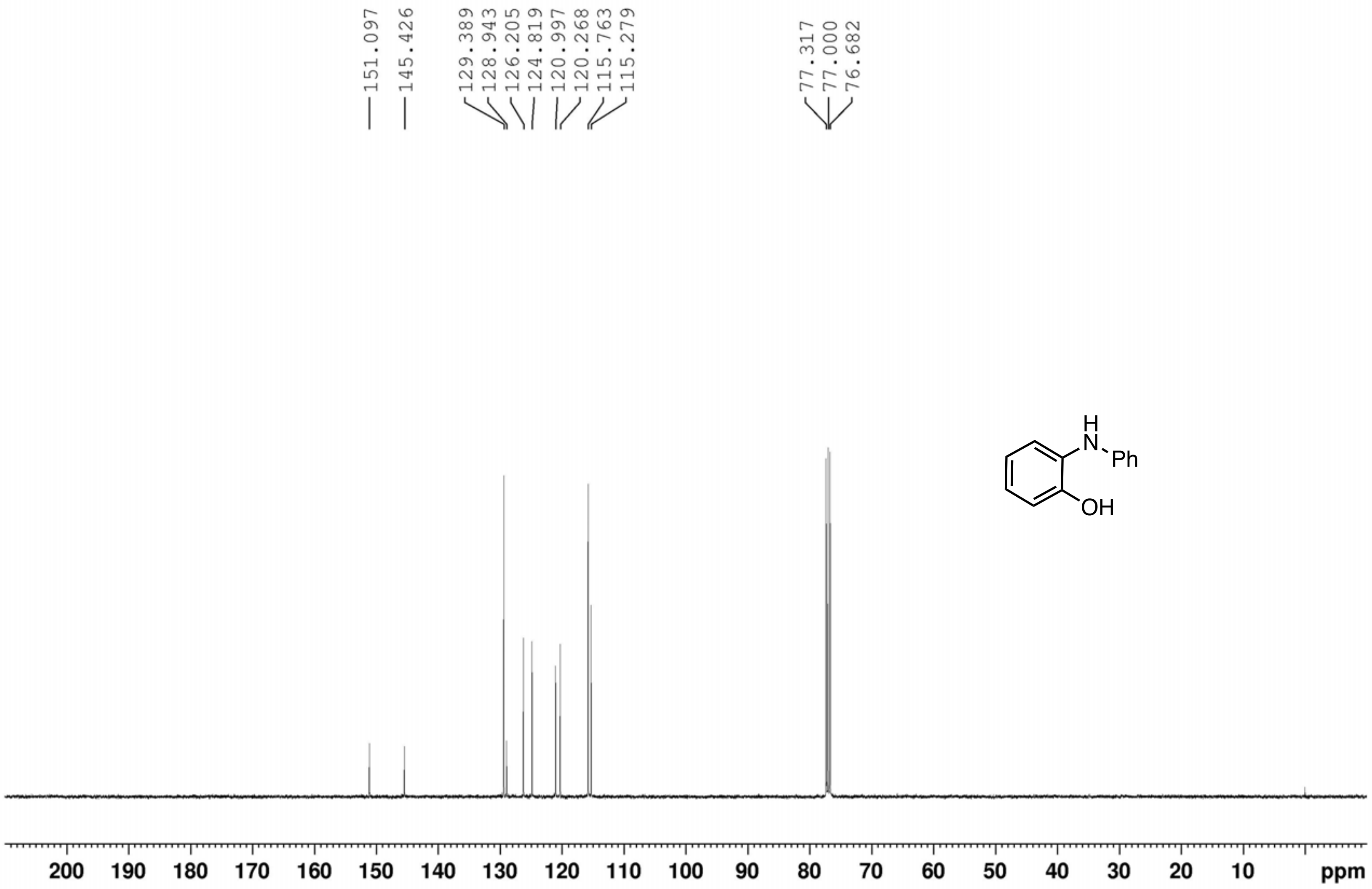

^{13}C NMR (100.6 MHz, $CDCl_3$) spectrum of **3z**

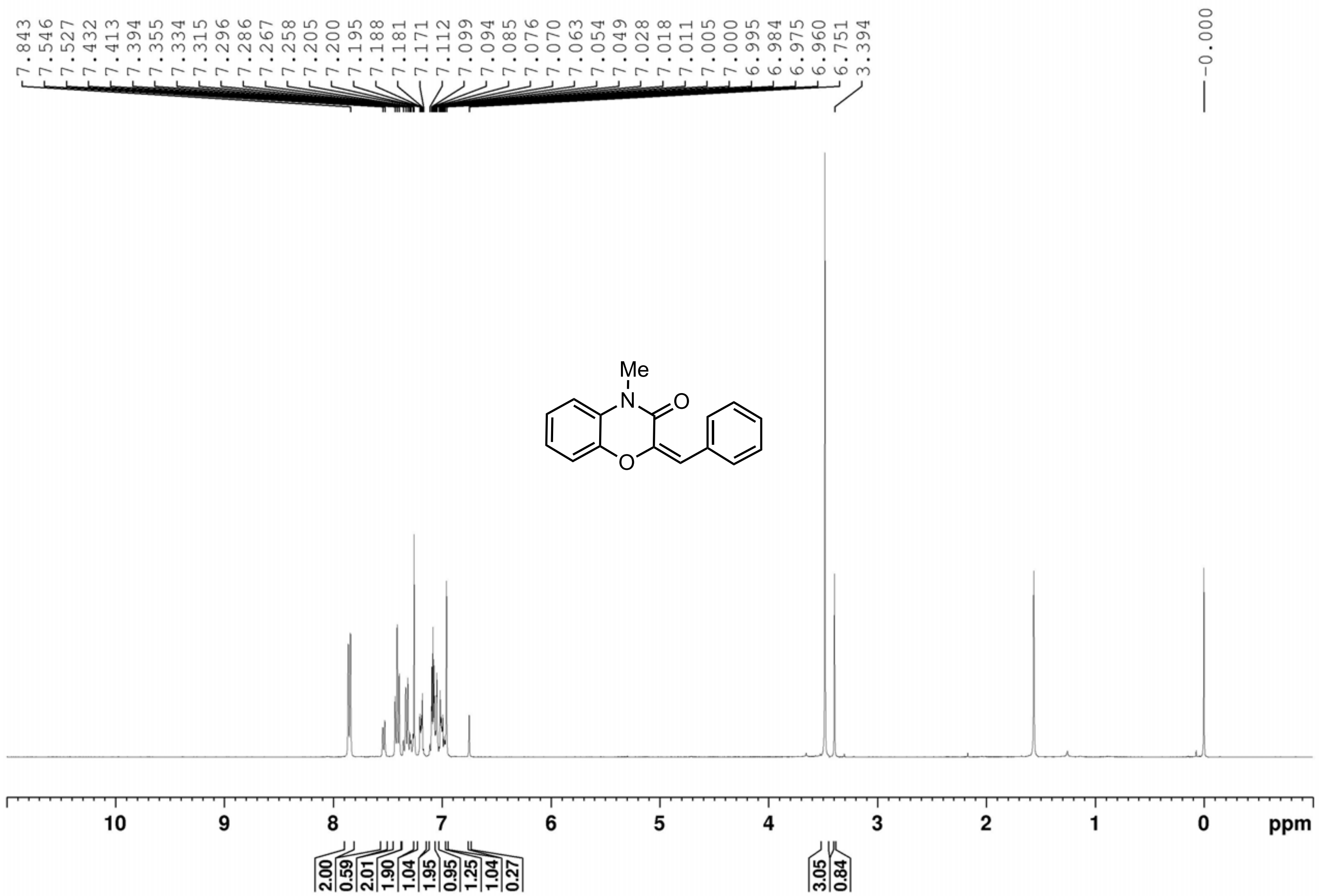

^{1}H NMR (400 MHz, $CDCl_3$) spectrum of **4a**

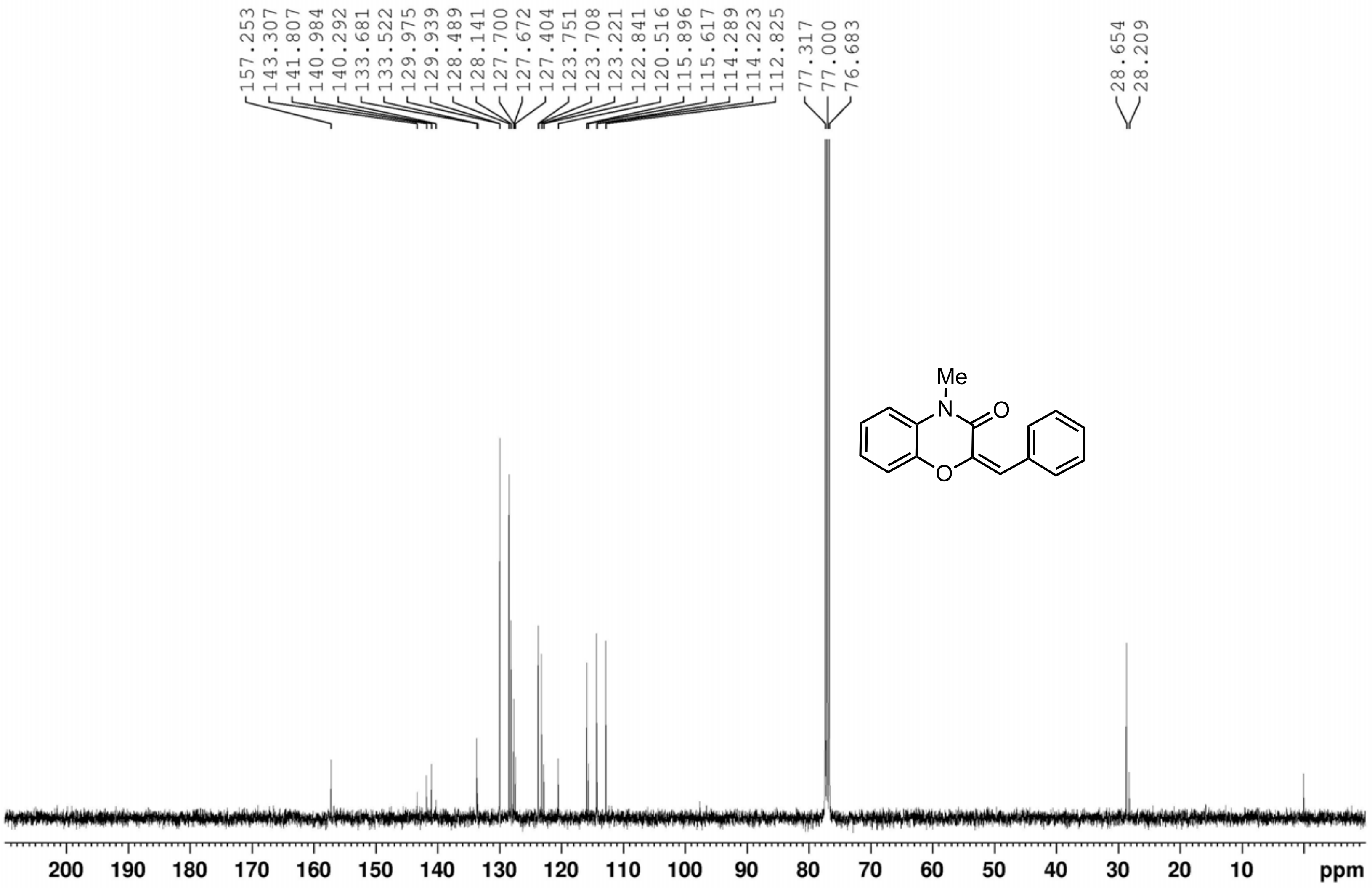

^{13}C NMR (100.6 MHz, $CDCl_3$) spectrum of **4a**

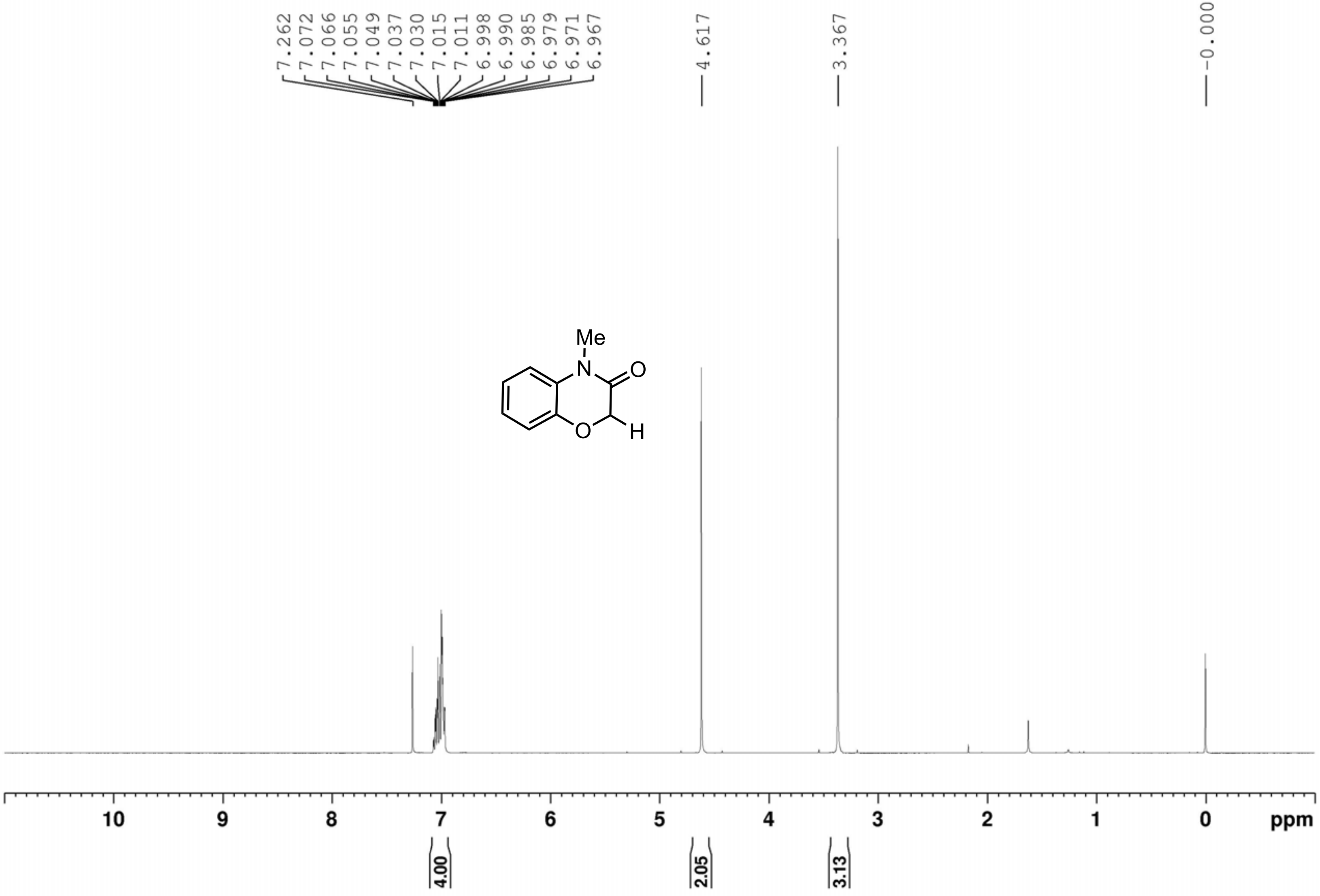

^{1}H NMR (400 MHz, $CDCl_3$) spectrum of **5a**

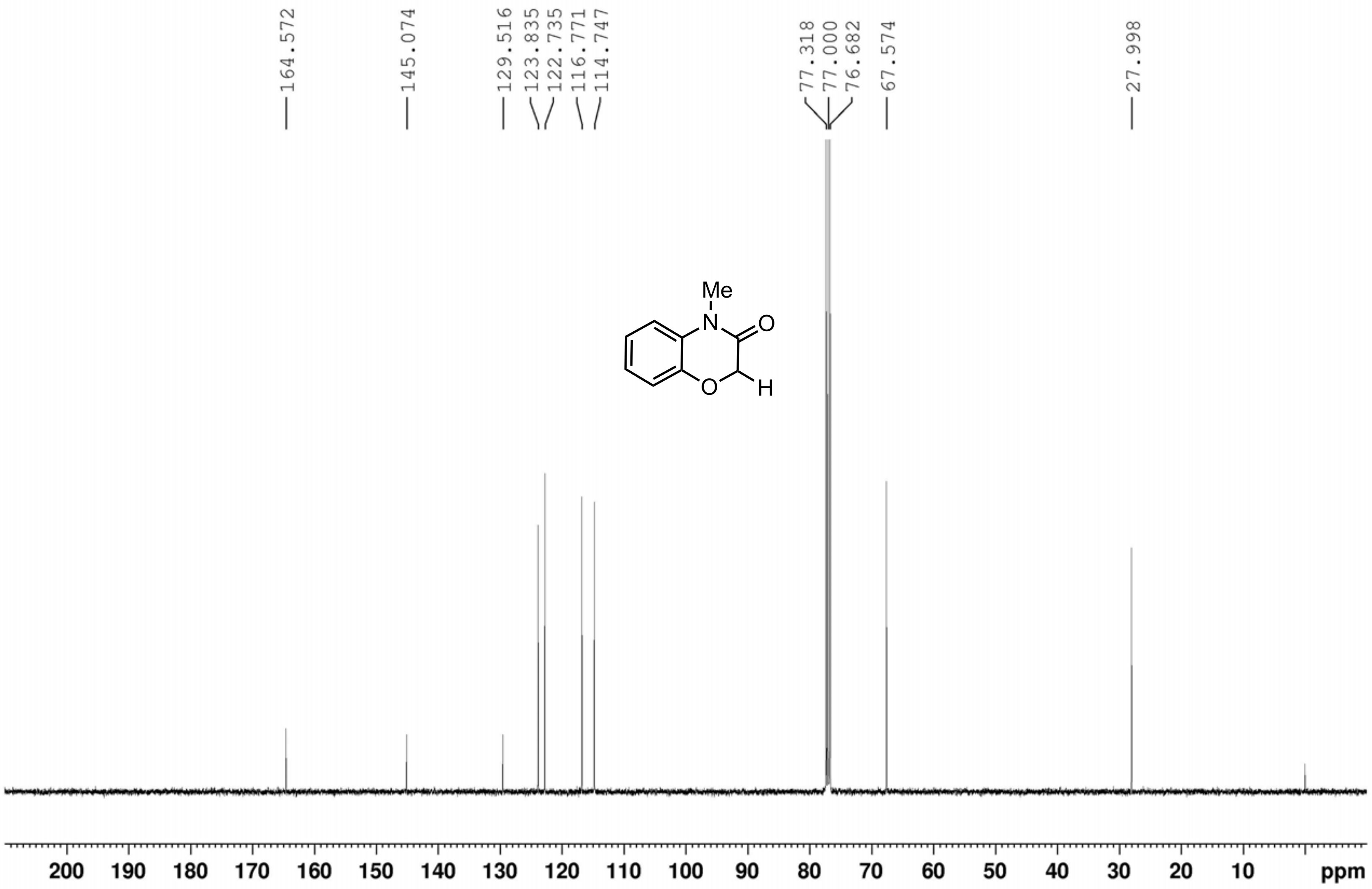

^{13}C NMR (100.6 MHz, $CDCl_3$) spectrum of **5a**

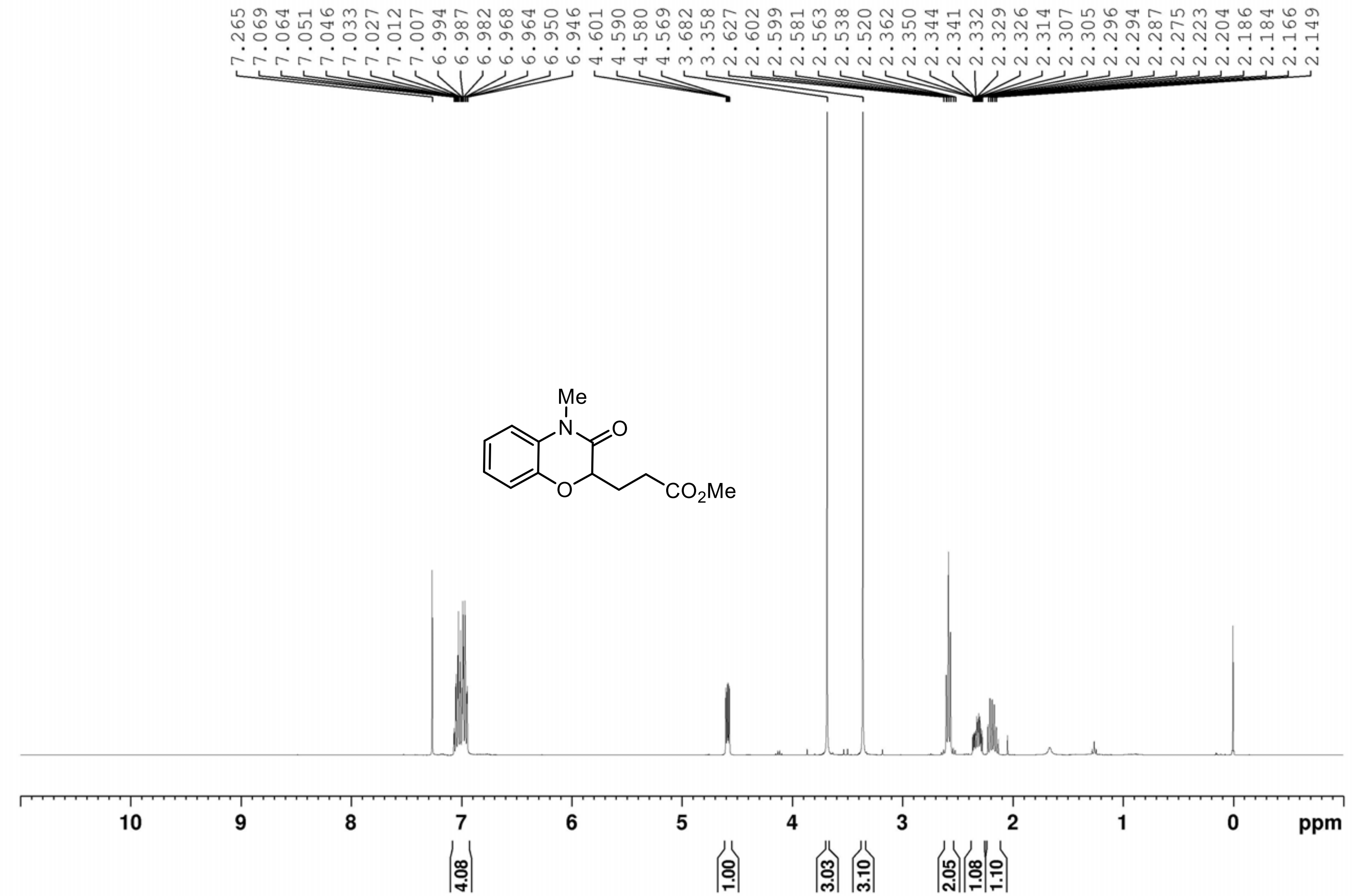

^{1}H NMR (400 MHz, $CDCl_3$) spectrum of **5b**

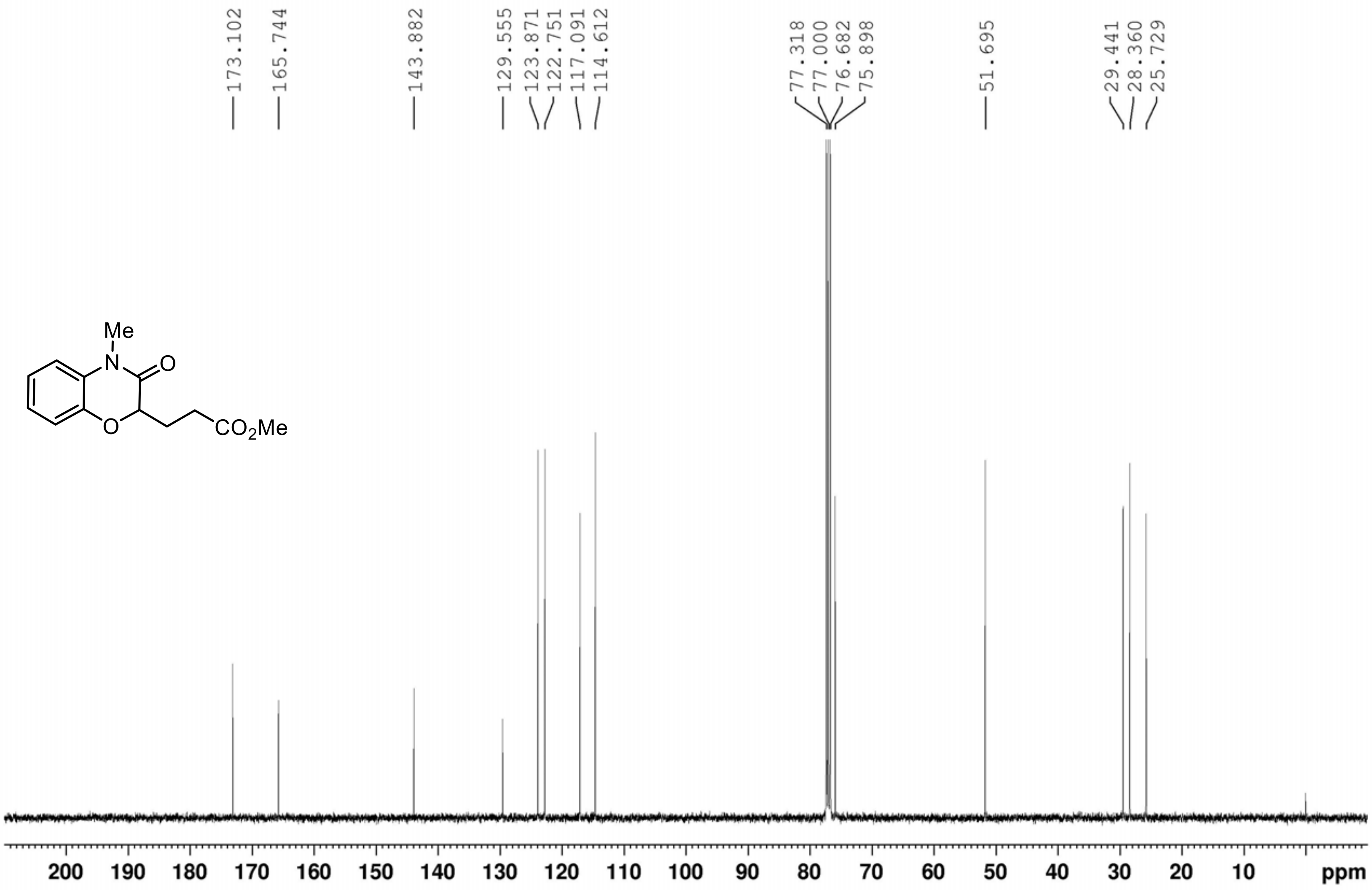

^{13}C NMR (100.6 MHz, $CDCl_3$) spectrum of **5b**

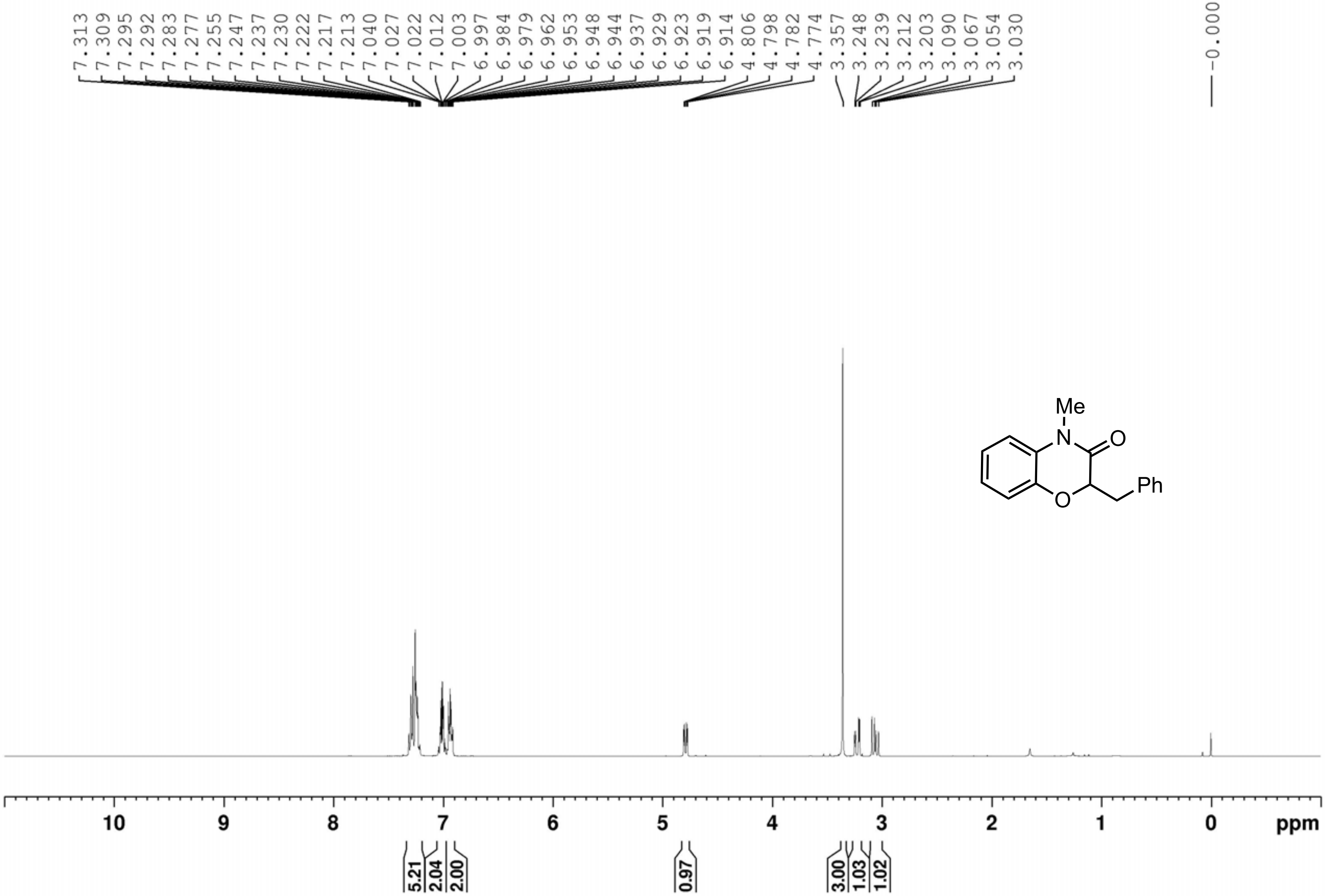

^{1}H NMR (400 MHz, $CDCl_3$) spectrum of **5c**

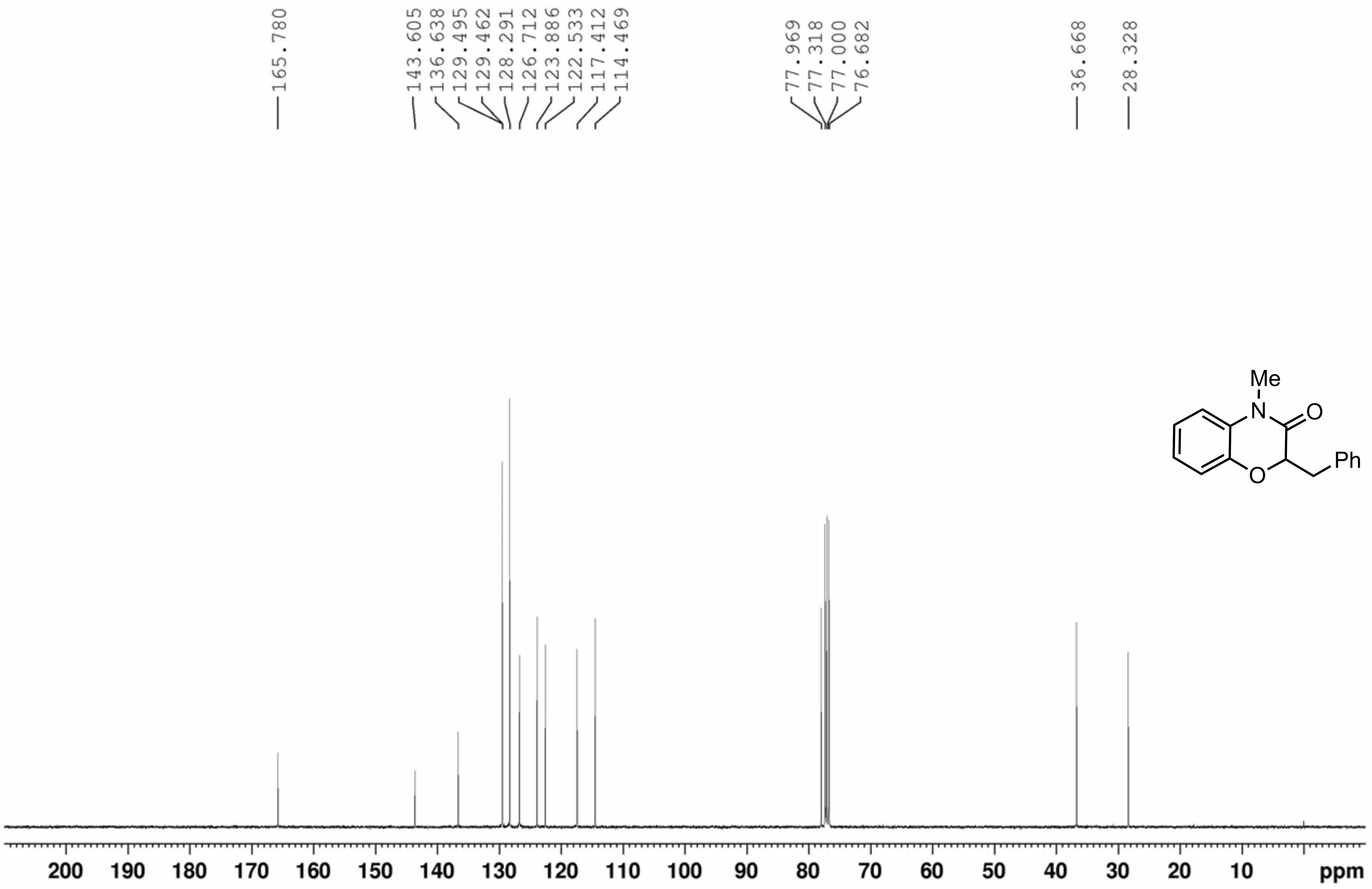

^{13}C NMR (100.6 MHz, $CDCl_3$) spectrum of **5c**

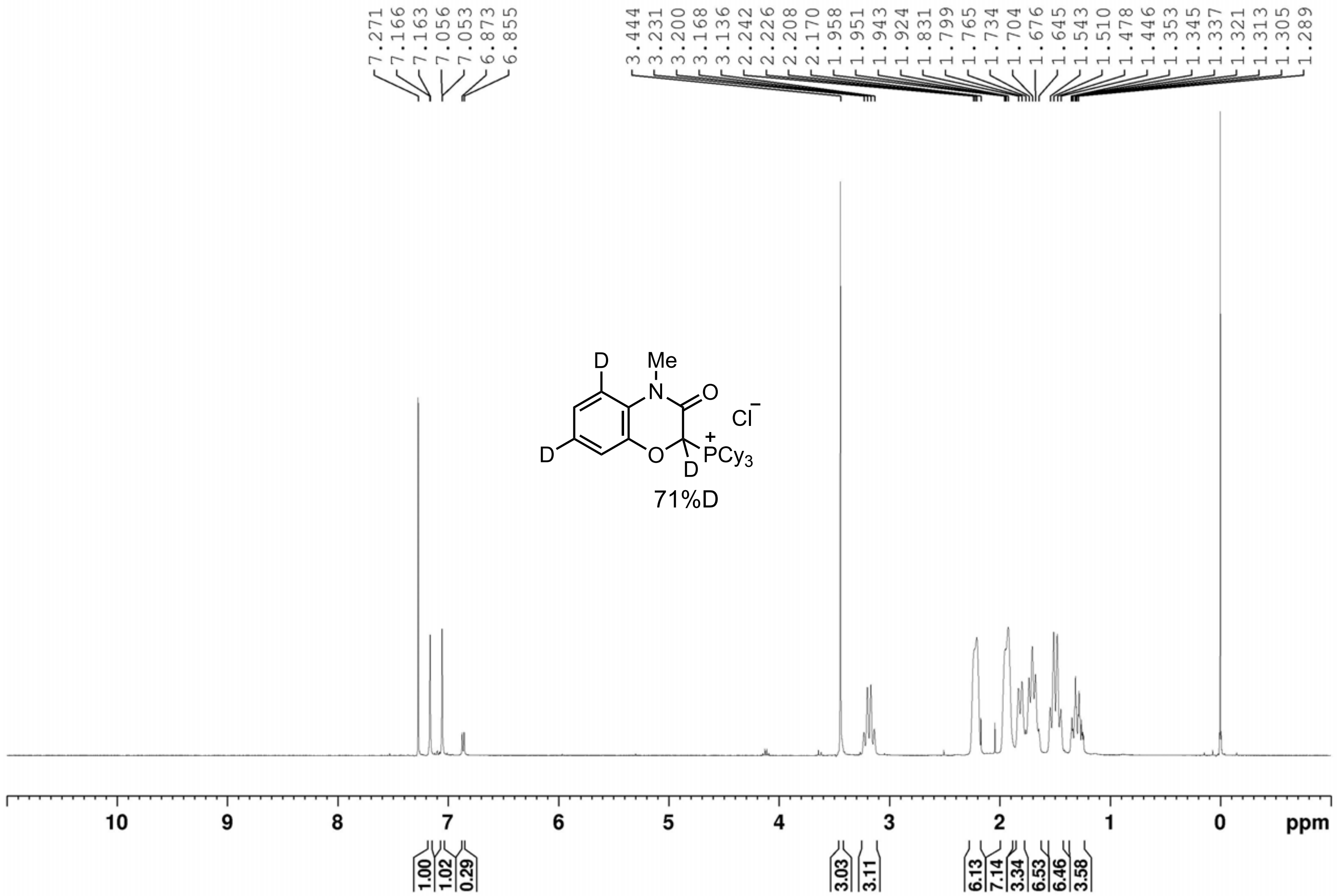

^1H NMR (400 MHz, $CDCl_3$) spectrum of **2a-D**

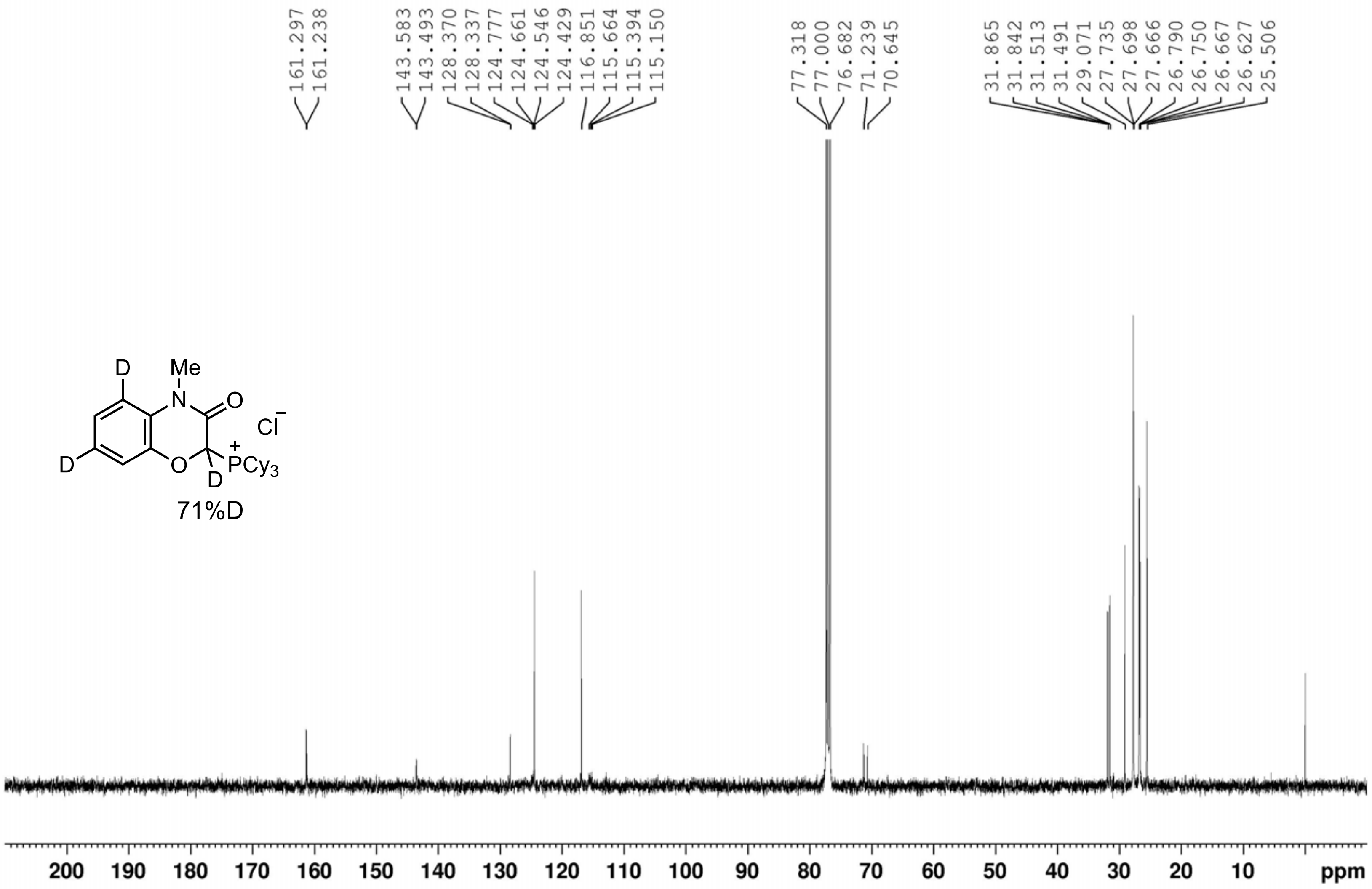

71%D

^{13}C NMR (100.6 MHz, $CDCl_3$) spectrum of **2a-D**

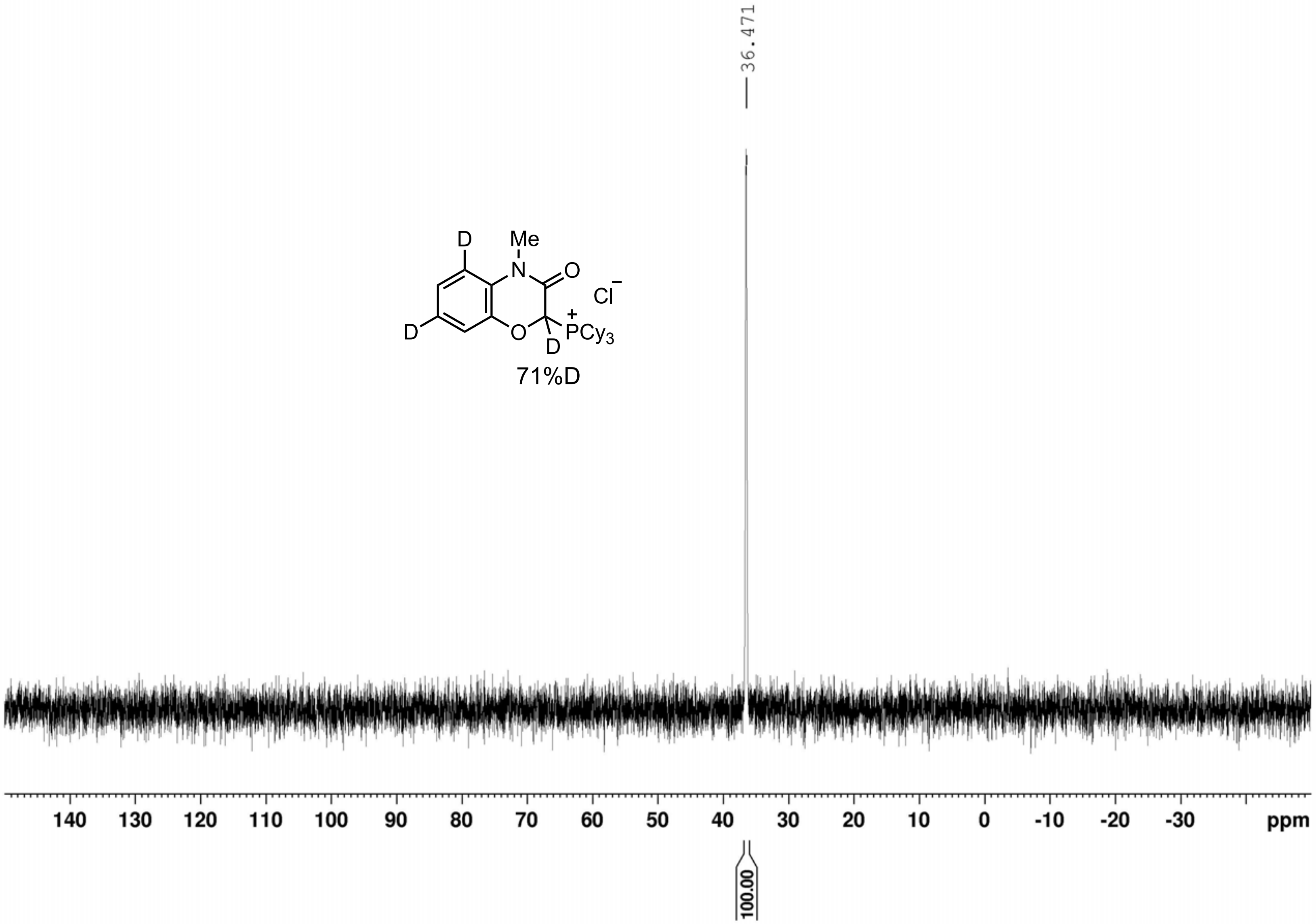

^{31}P NMR (162 MHz, $CDCl_3$) spectrum of **2a-D**

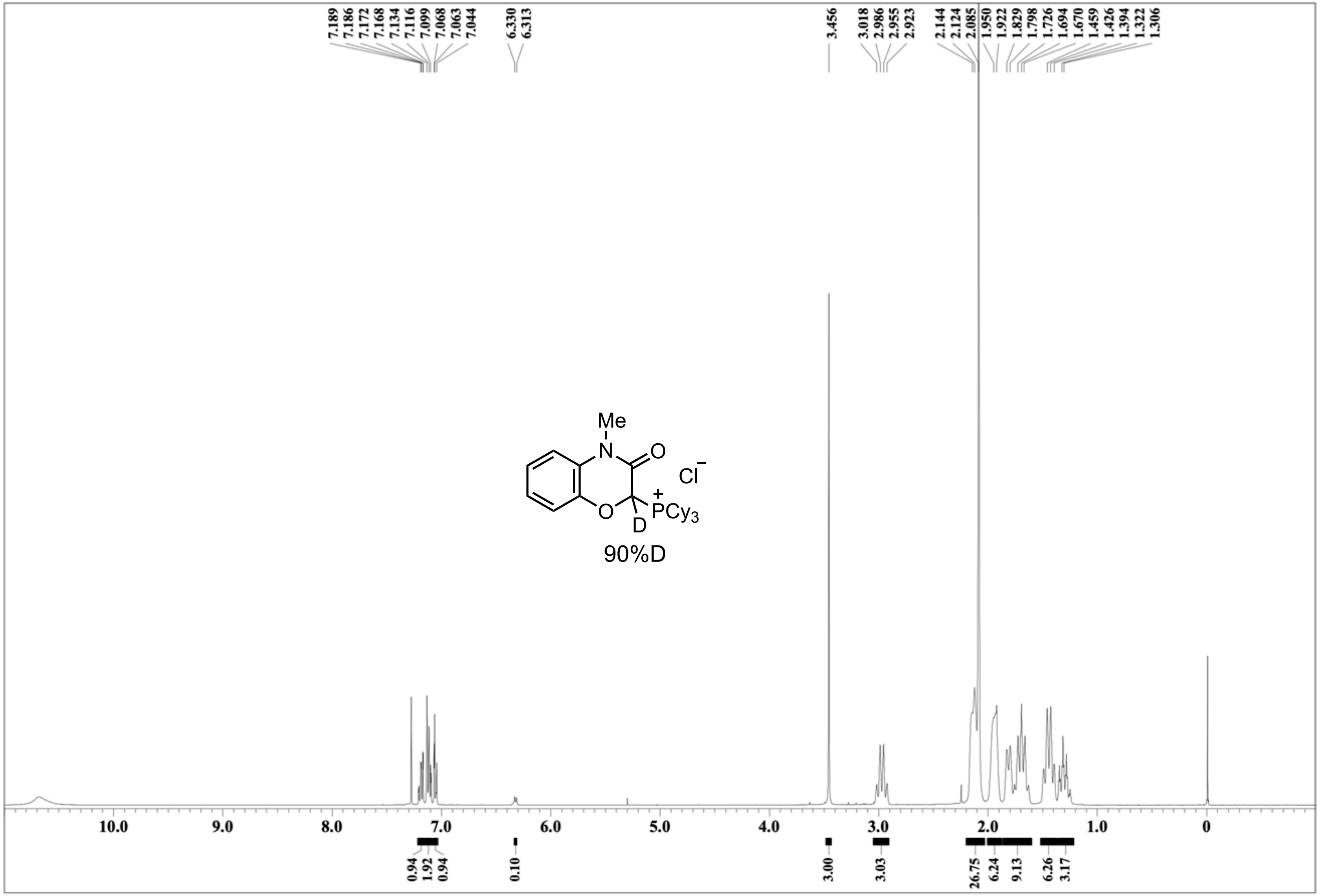

^{1}H NMR (400 MHz, $CDCl_3$) spectrum of **2a-D** (with AcOD)

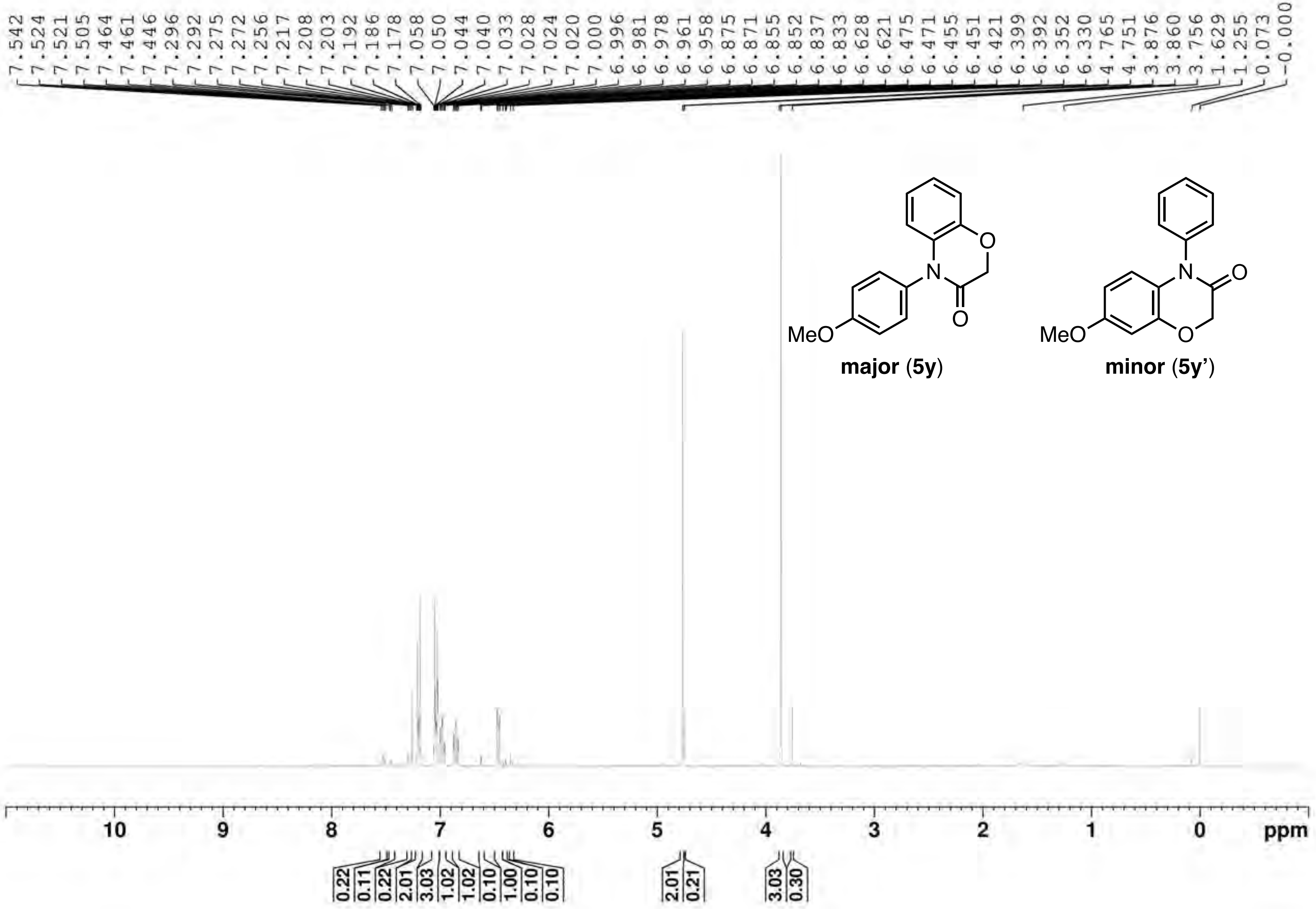

^{1}H NMR (400 MHz, $CDCl_3$) spectrum of **5y** and **5y'**

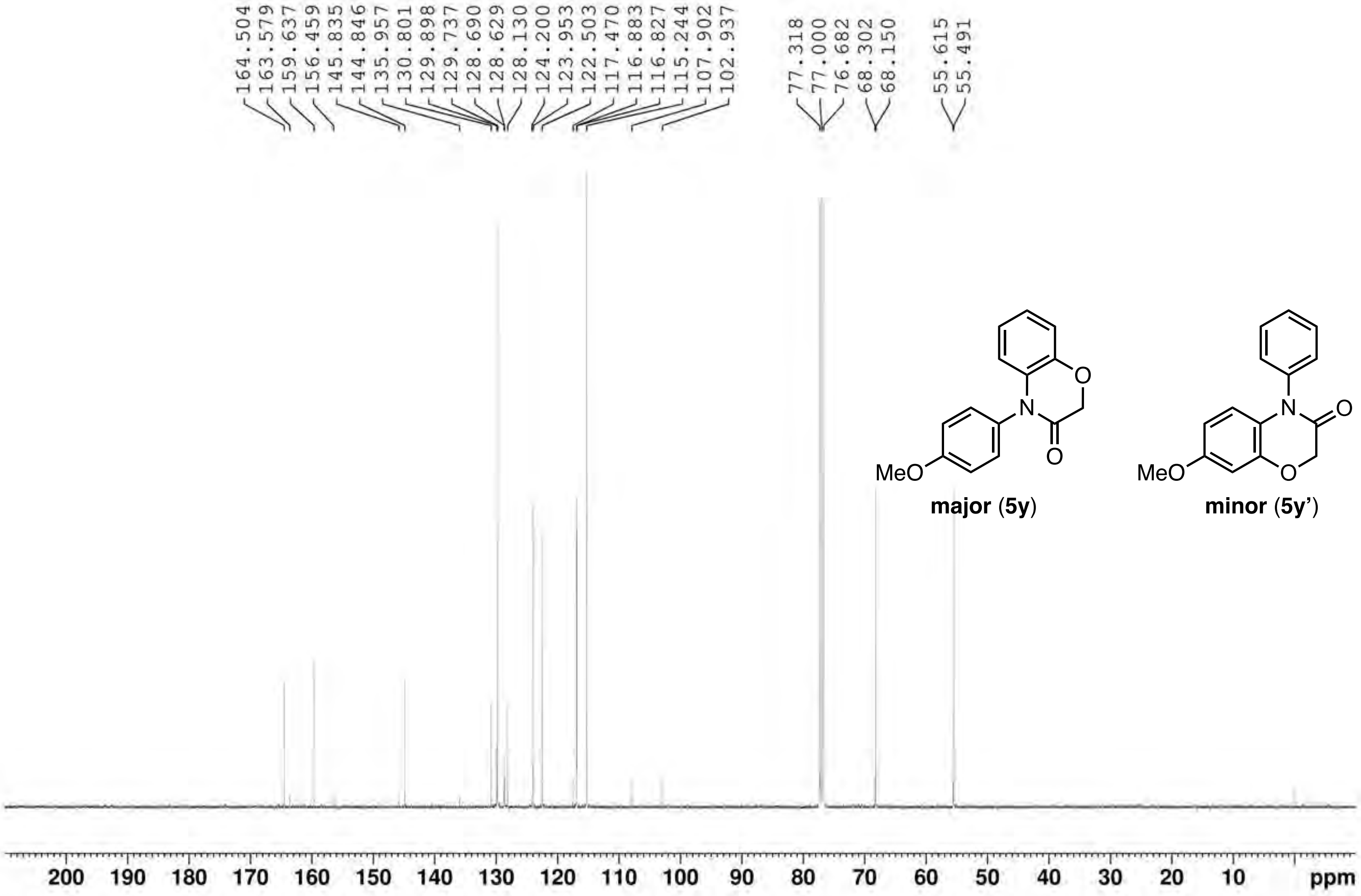

^{13}C NMR (100.6 MHz, $CDCl_3$) spectrum of **5y** and **5y'**

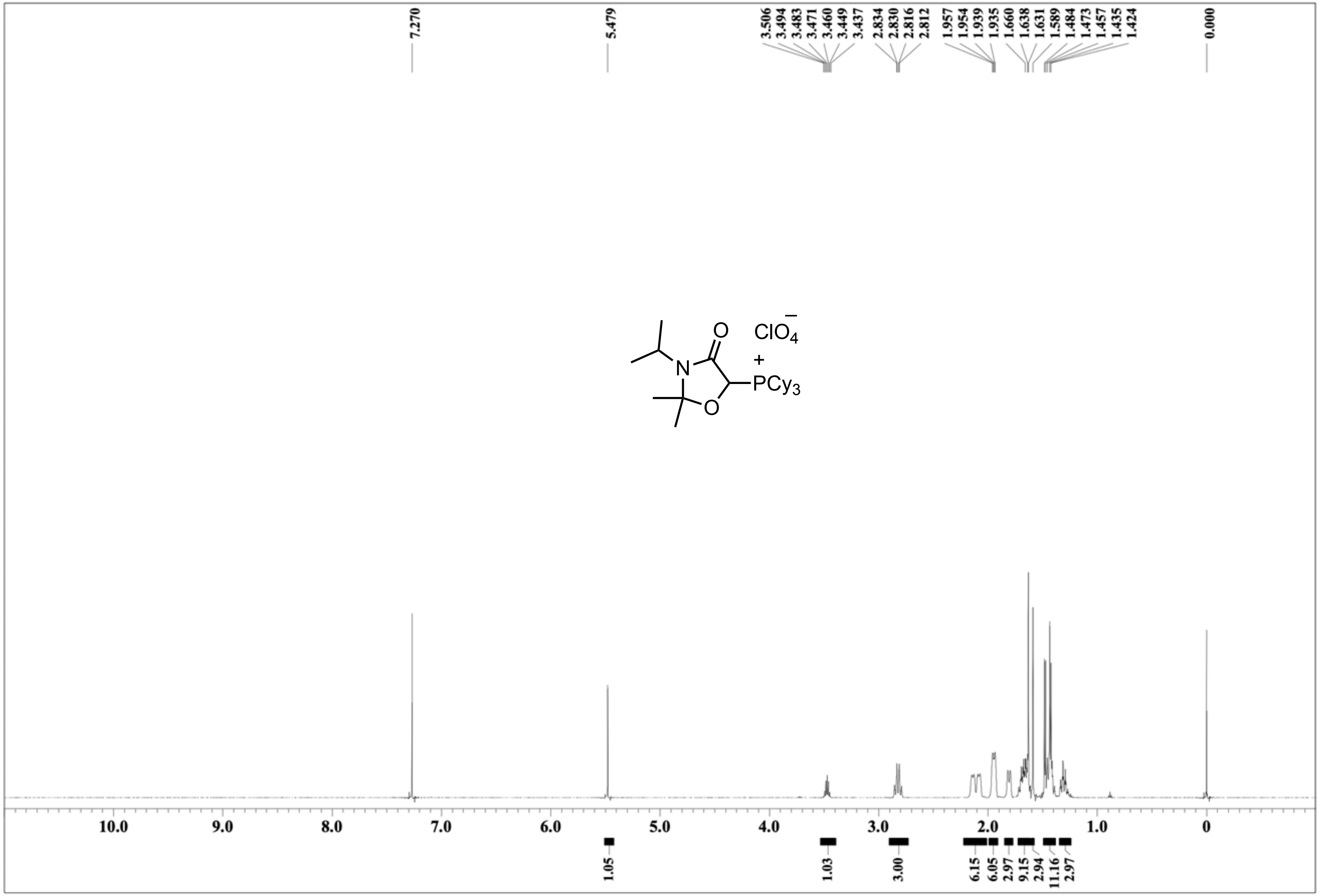

^{1}H NMR (400 MHz, $CDCl_3$) spectrum of **A2**

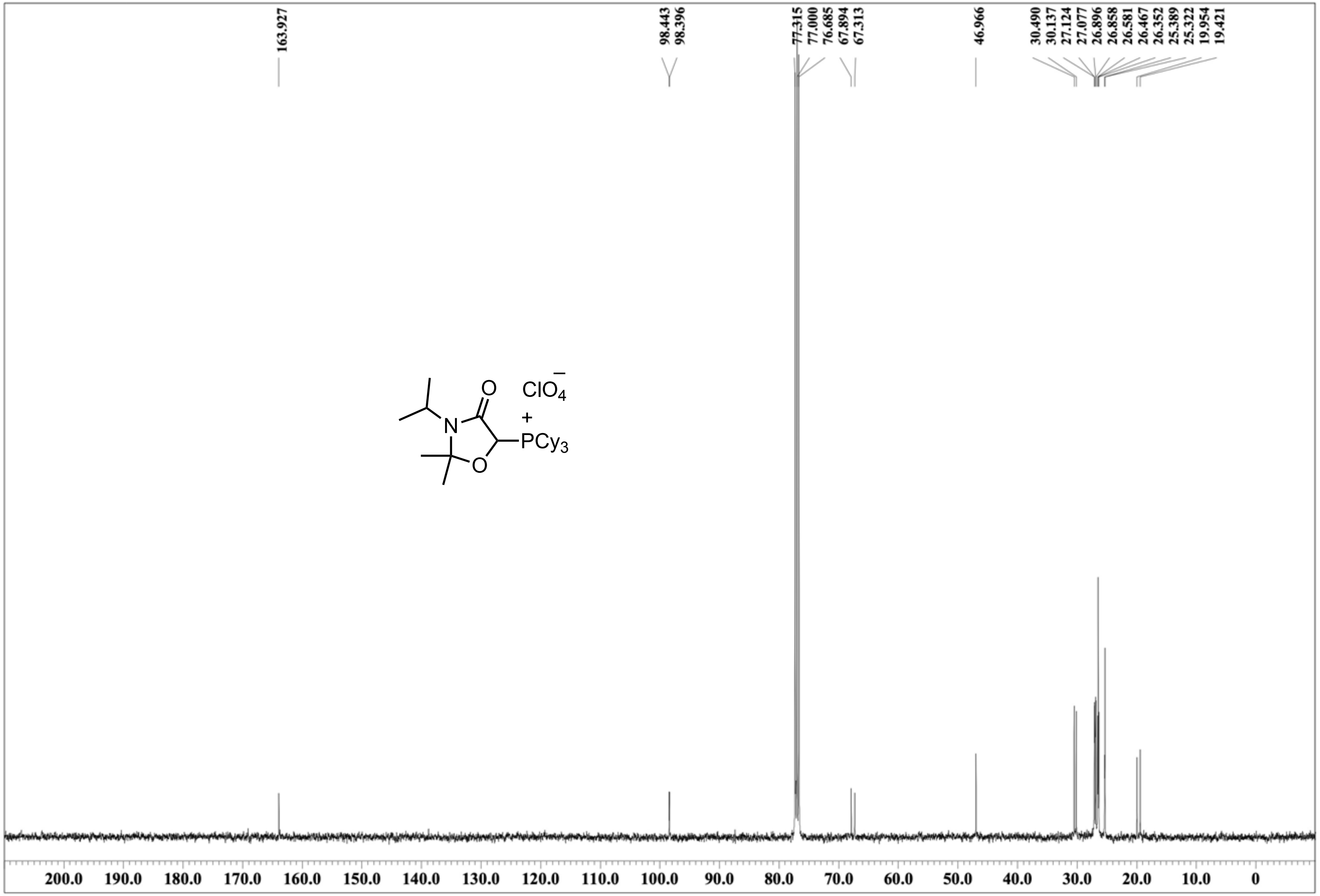

^{13}C NMR (100.6 MHz, $CDCl_3$) spectrum of **A2**

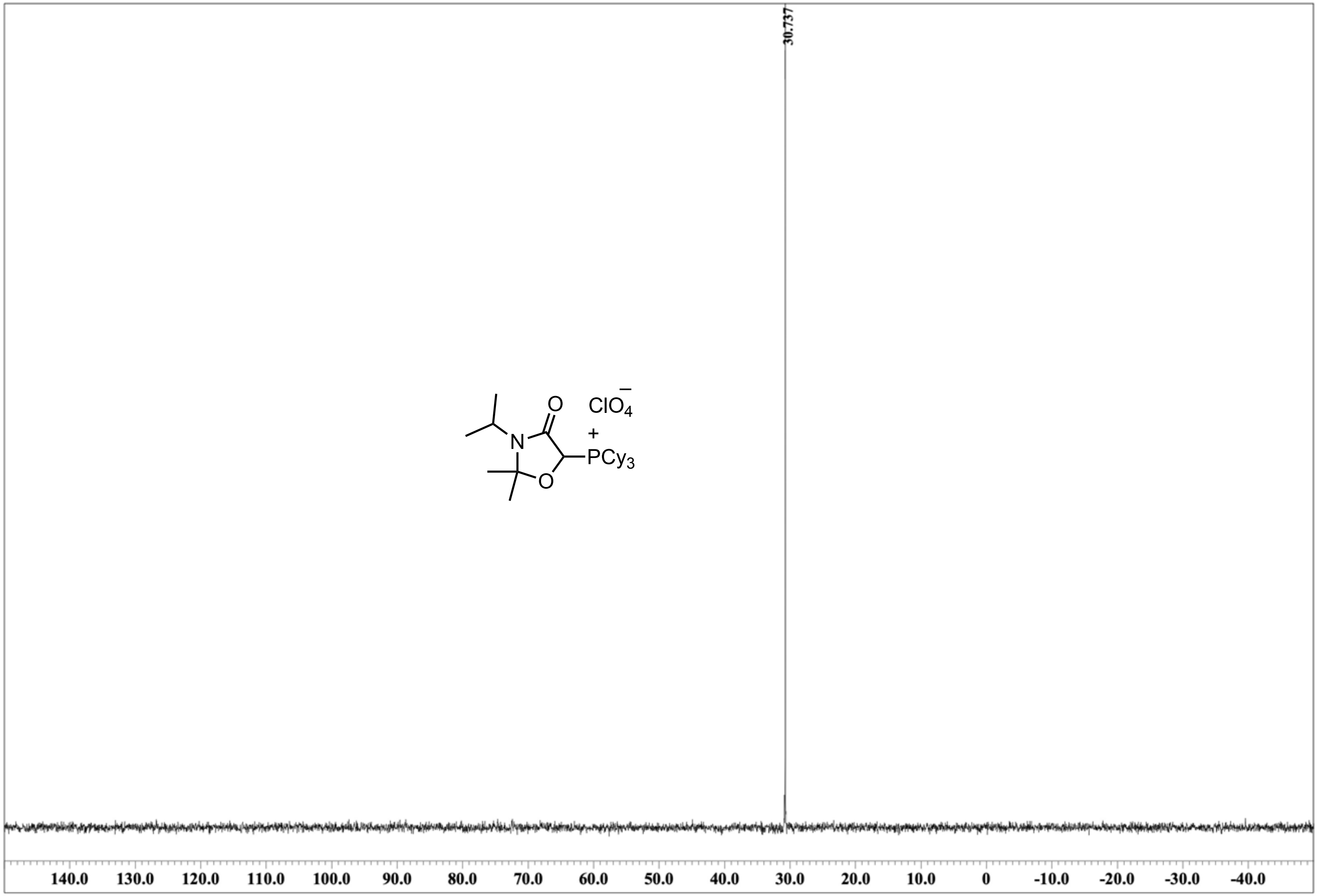

^{31}P NMR (162 MHz, $CDCl_3$) spectrum of **A2**

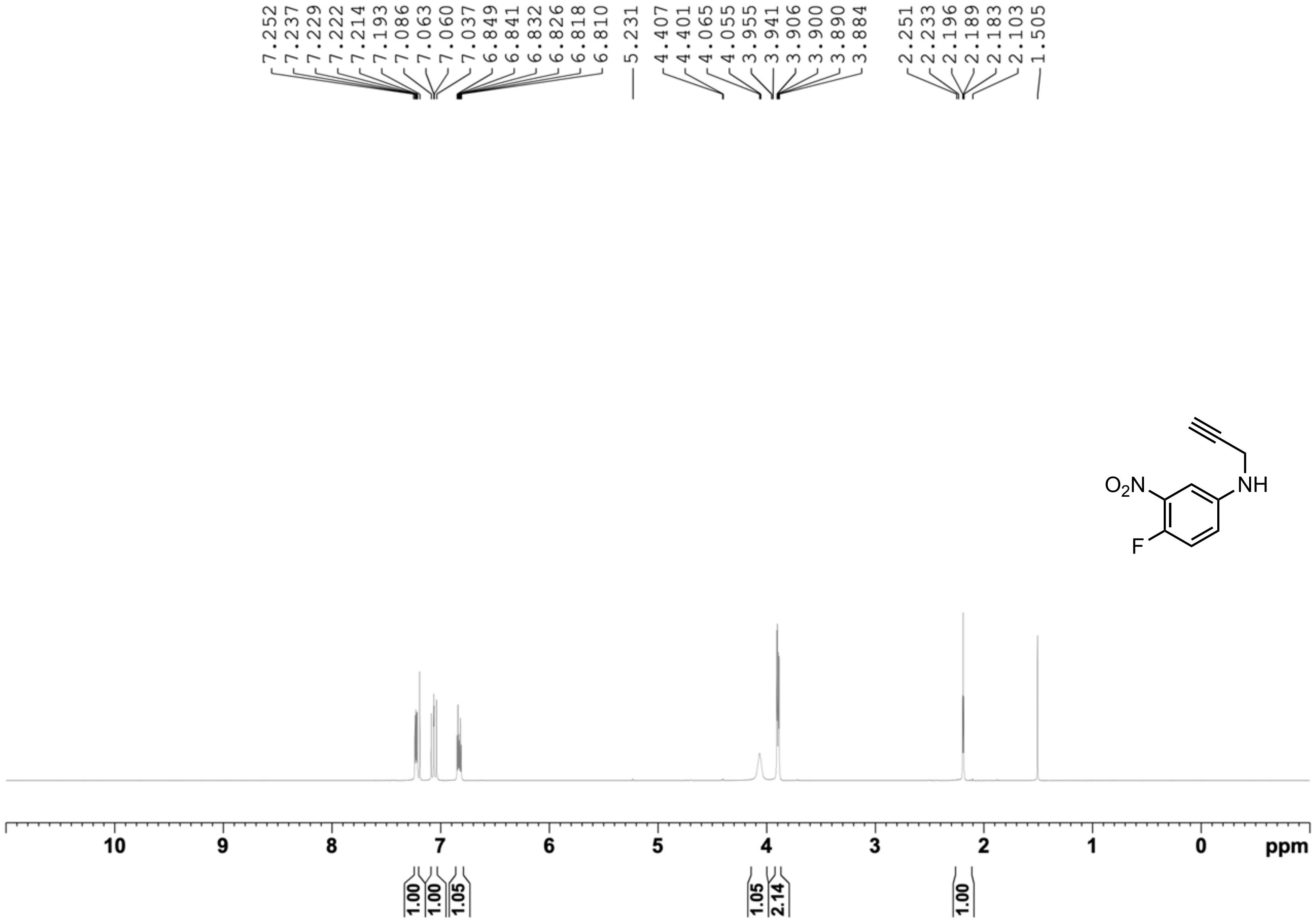

^{1}H NMR (400 MHz, $CDCl_3$) spectrum of **S2**

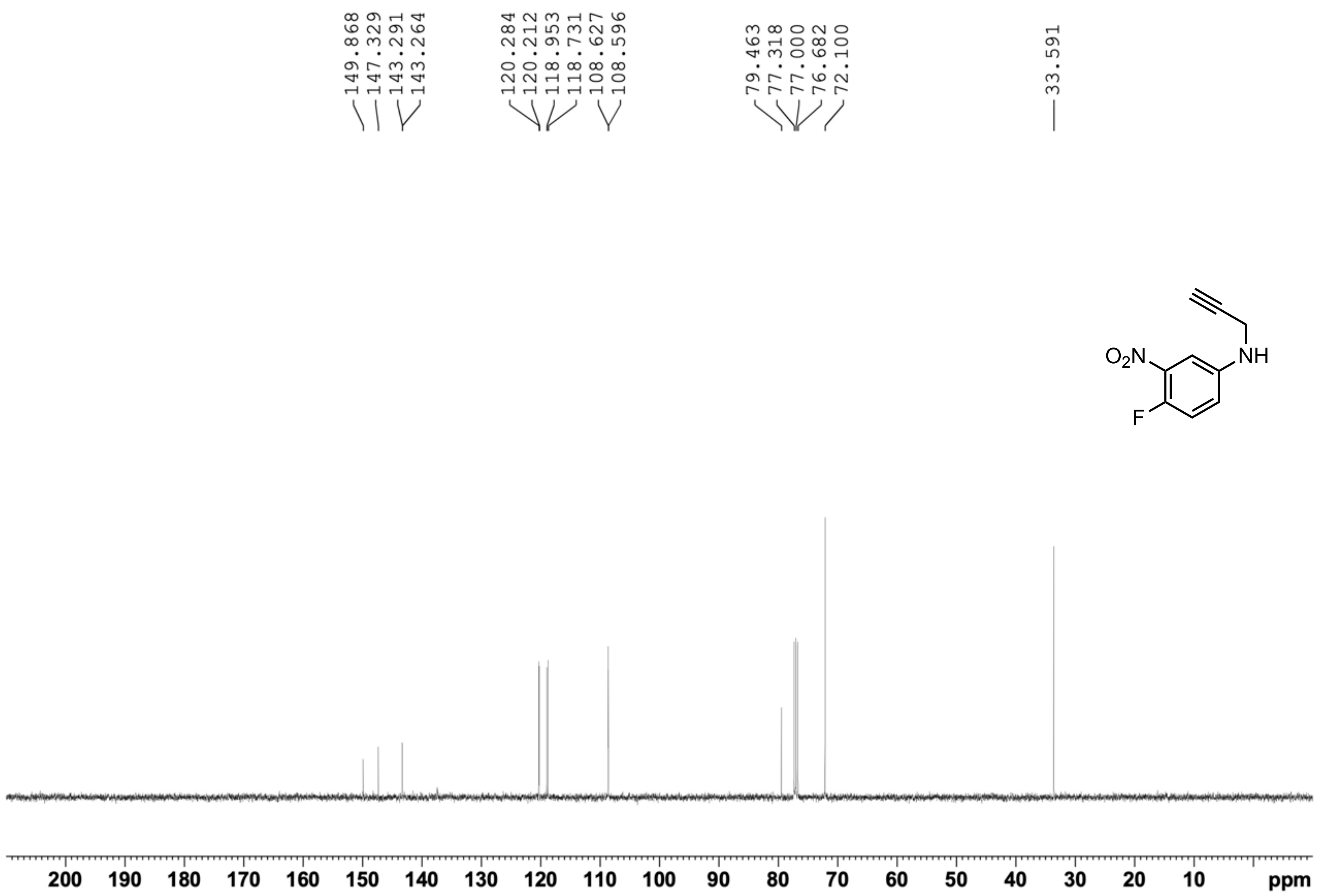

^{13}C NMR (100.6 MHz, $CDCl_3$) spectrum of **S2**

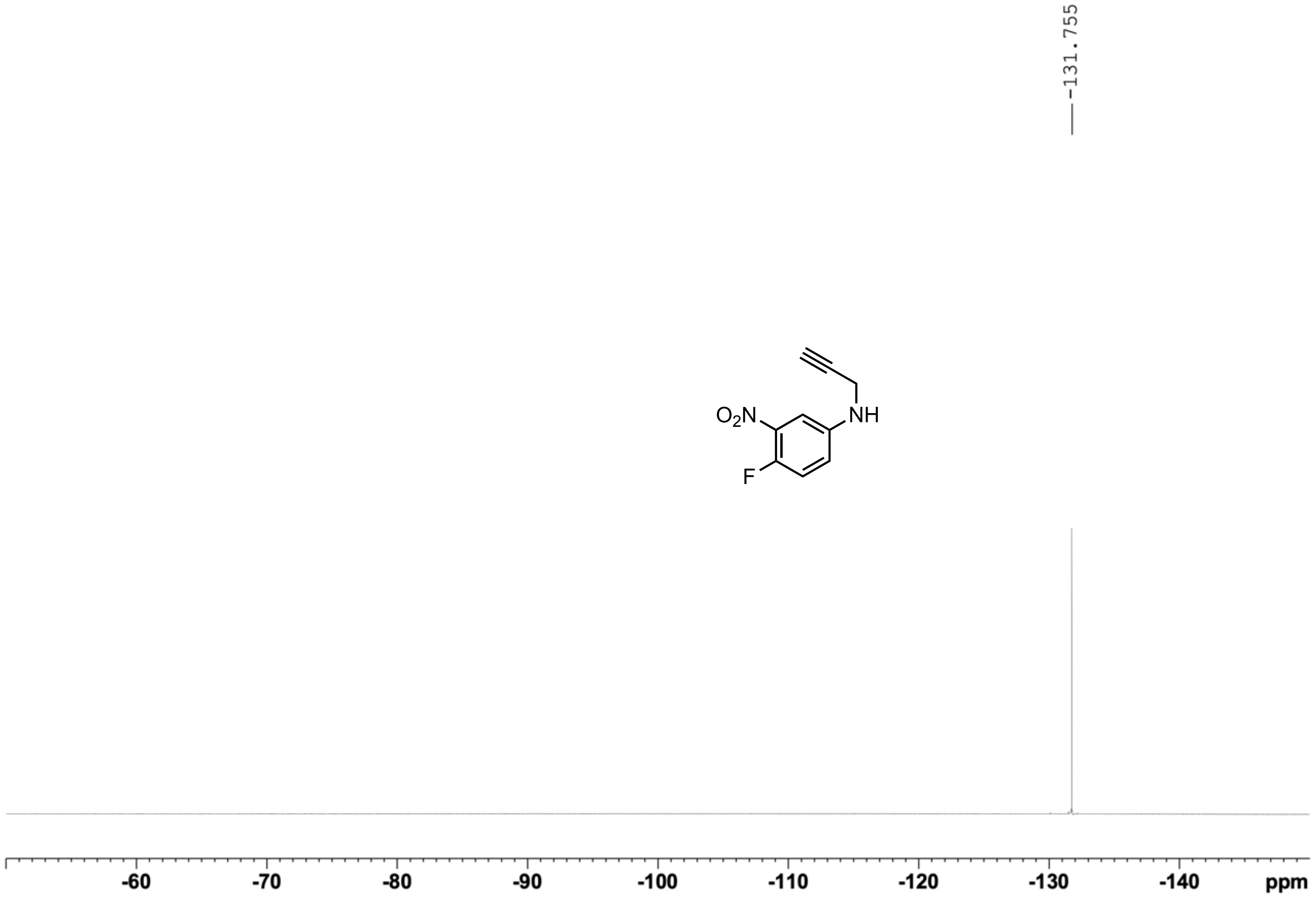

^{19}F NMR (376.5 MHz, $CDCl_3$) spectrum of **S2**

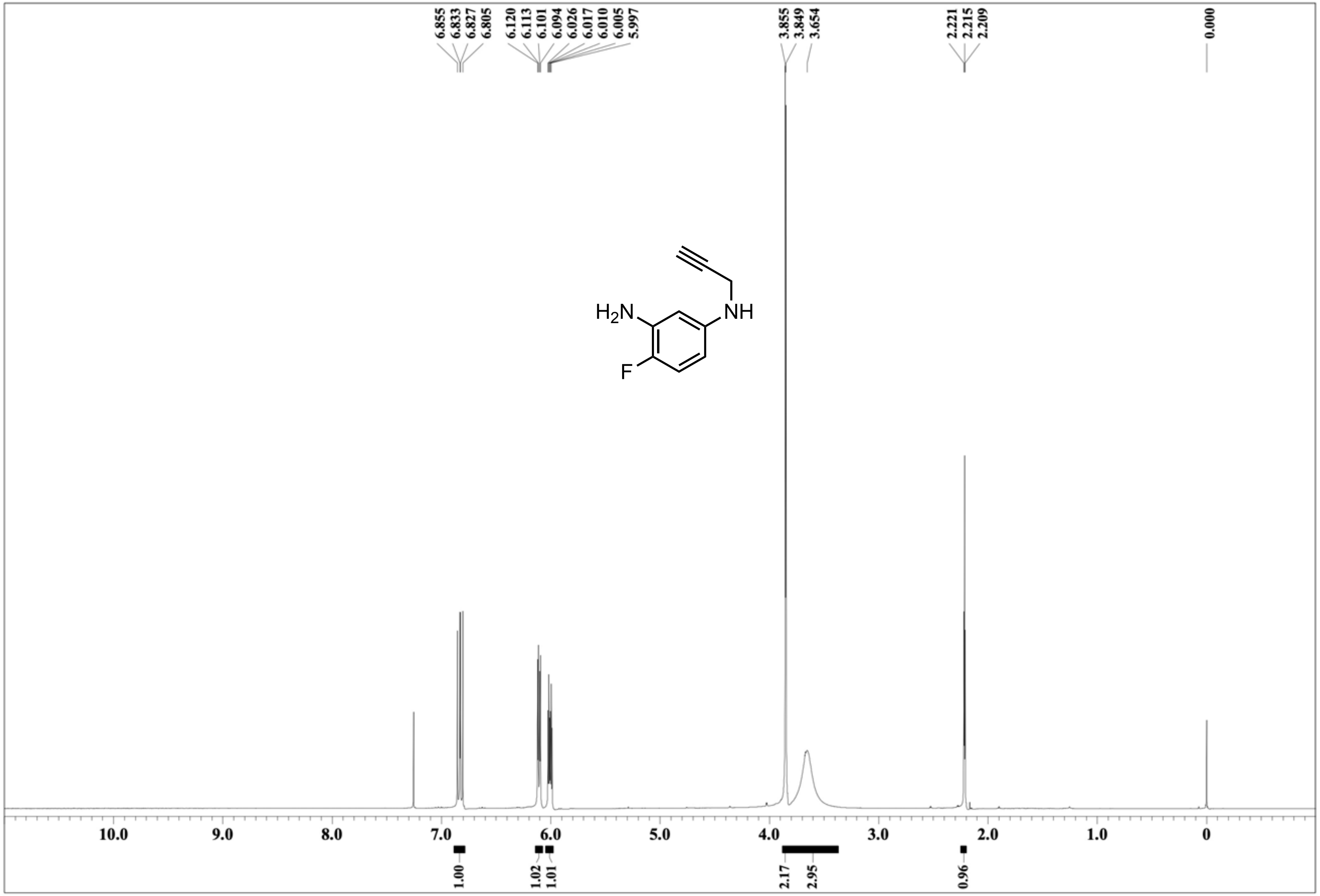

^{1}H NMR (400 MHz, $CDCl_3$) spectrum of **S3**

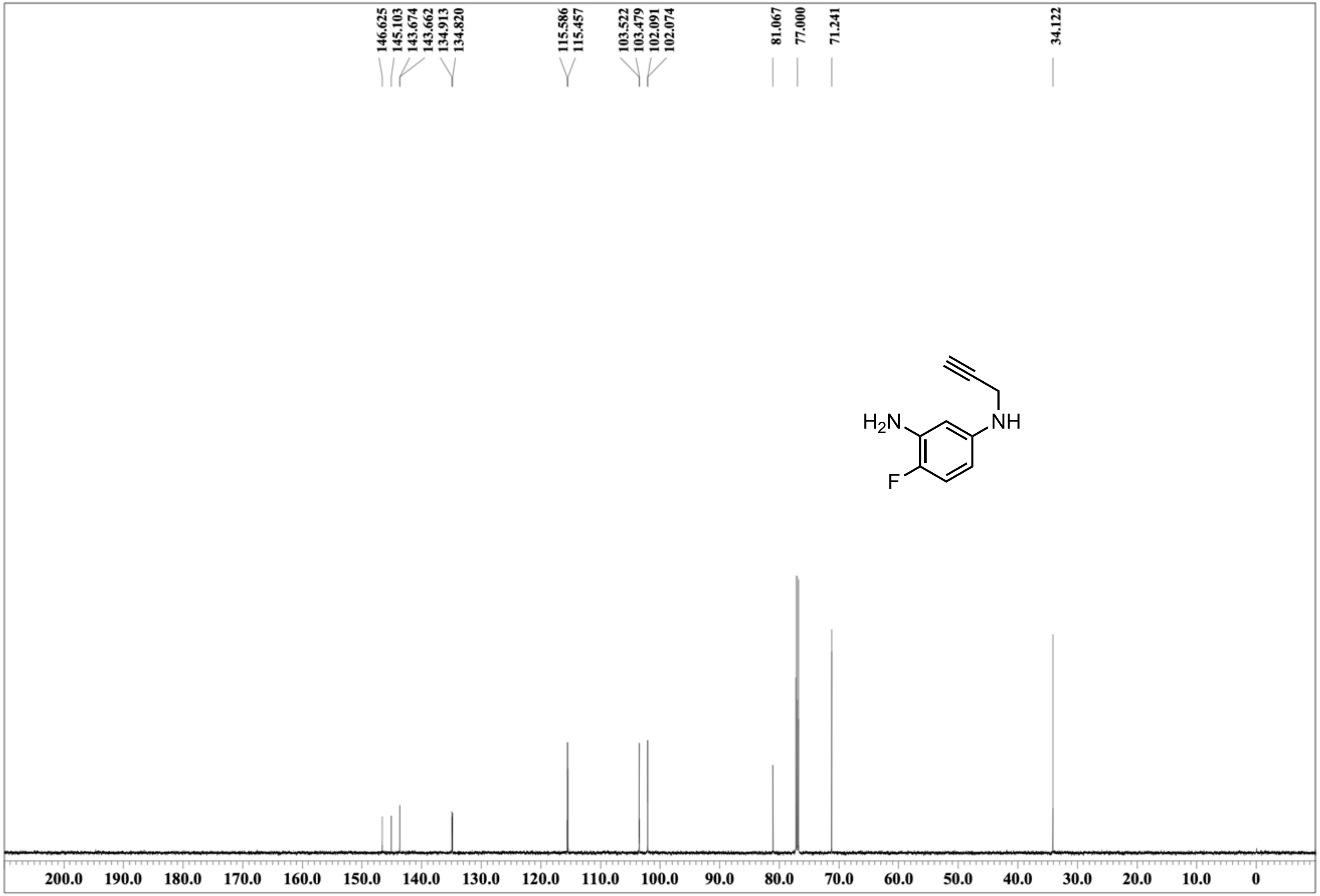

^{13}C NMR (100.6 MHz, $CDCl_3$) spectrum of **S3**

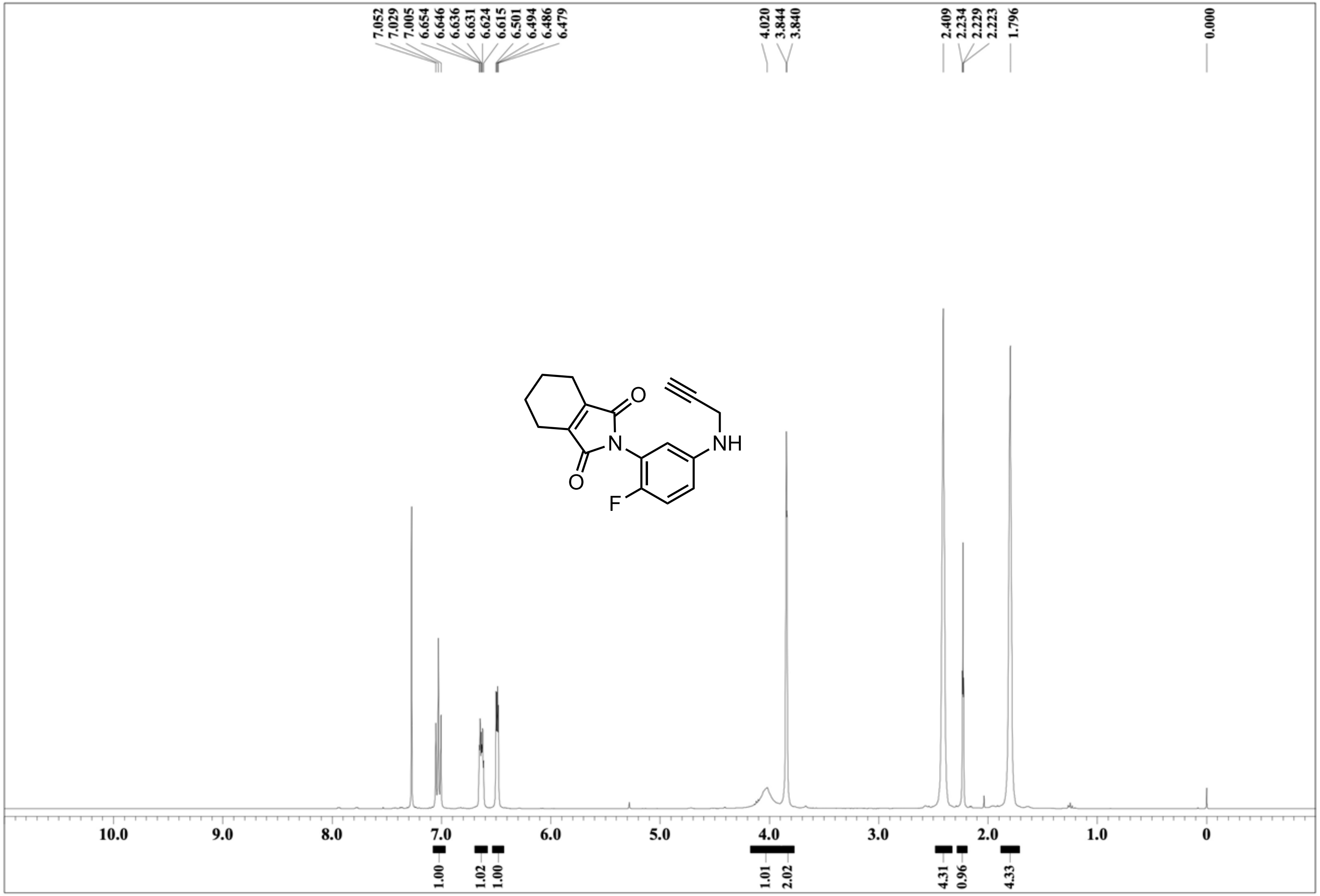

^{1}H NMR (400 MHz, $CDCl_3$) spectrum of **S4**

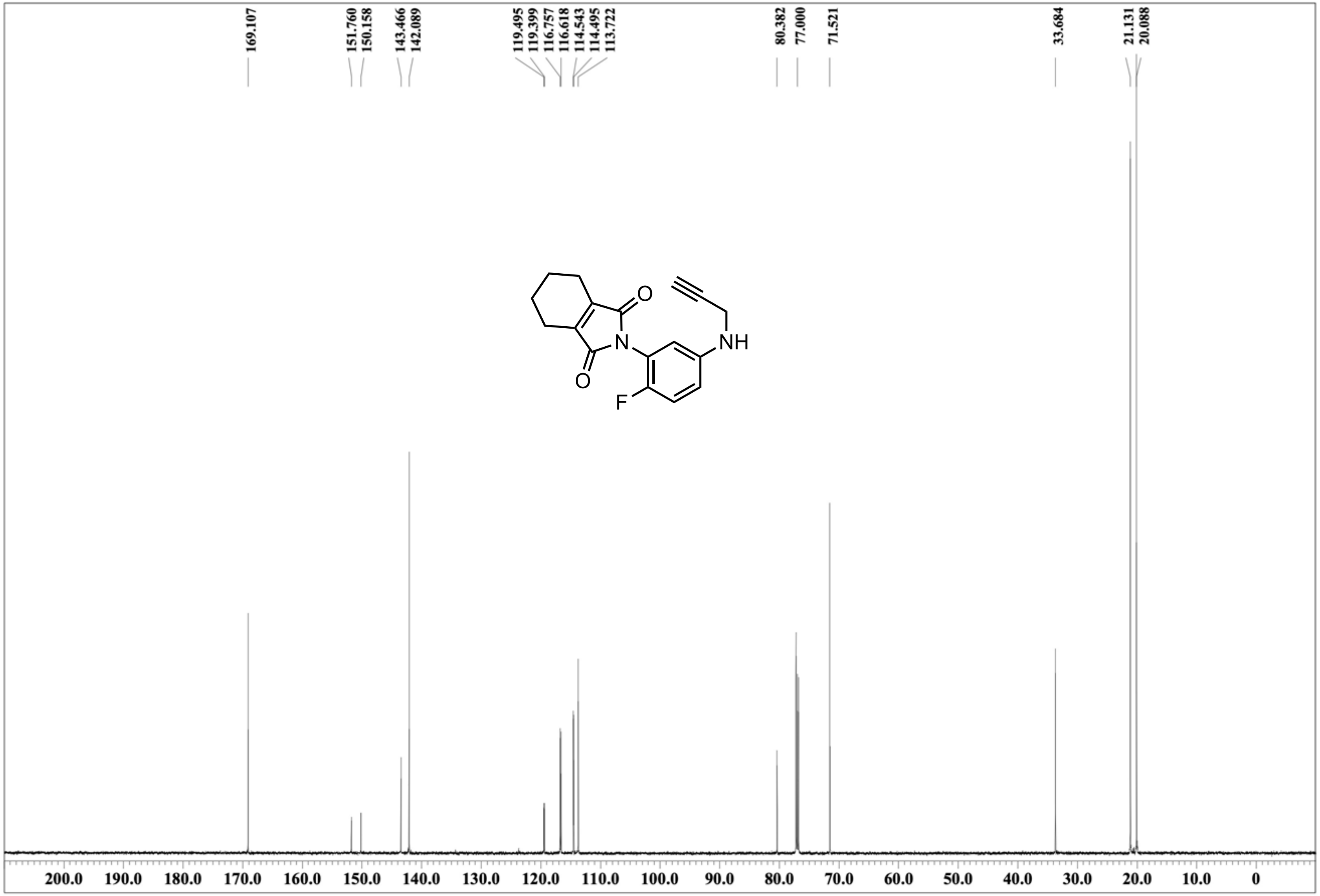

^{13}C NMR (100.6 MHz, $CDCl_3$) spectrum of **S4**

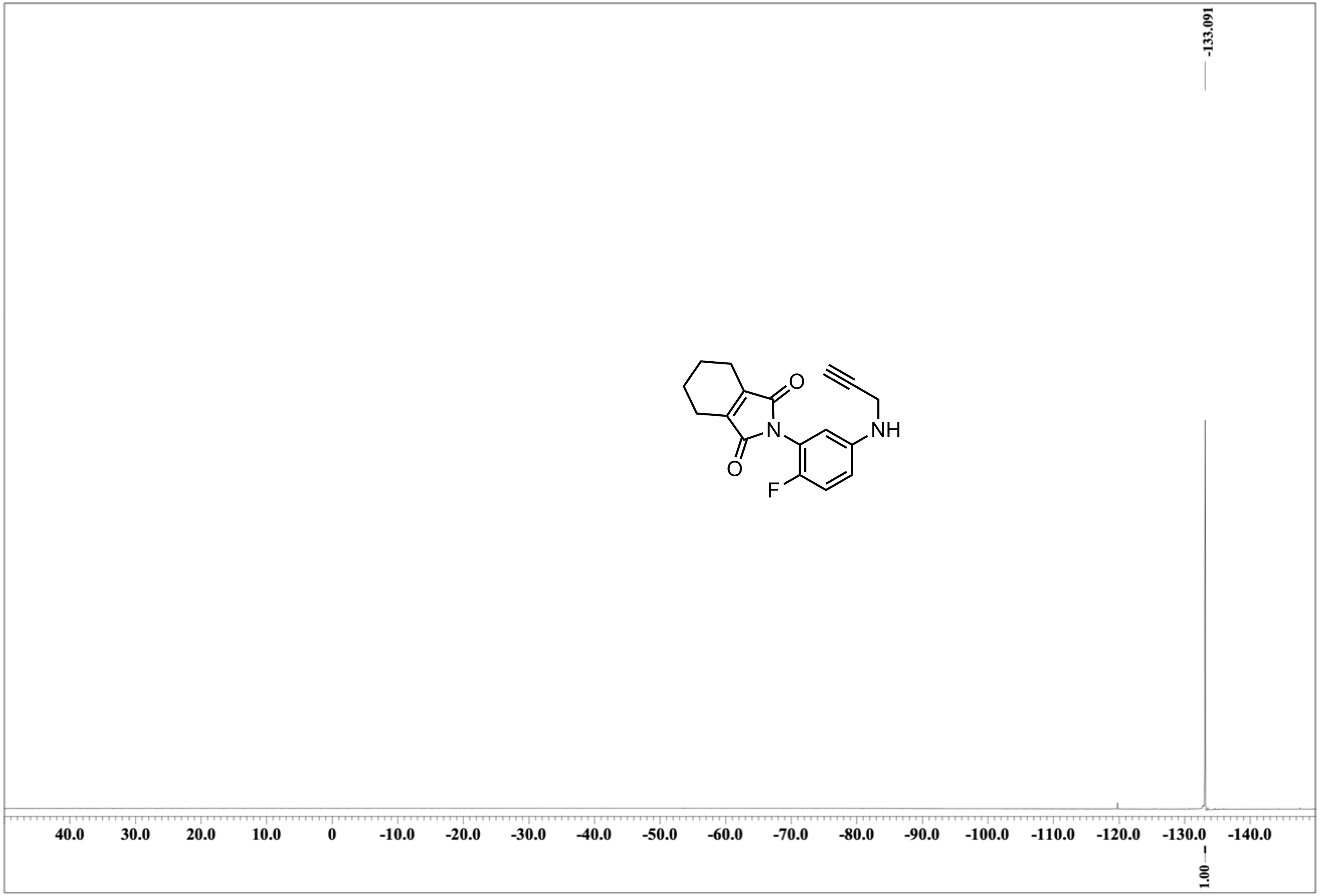

^{19}F NMR (376.5 MHz, $CDCl_3$) spectrum of **S4**

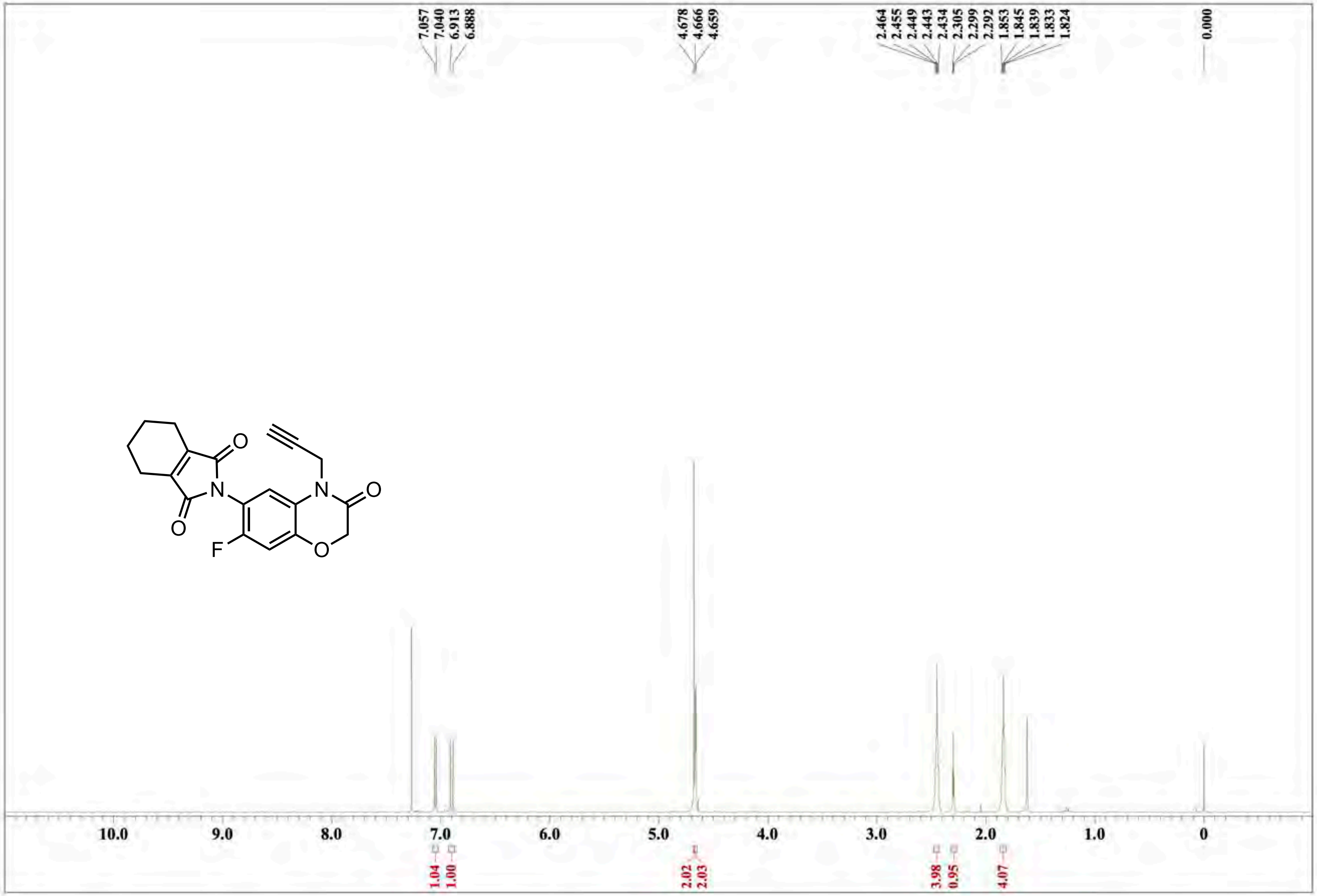

^{1}H NMR (400 MHz, $CDCl_3$) spectrum of **S6**

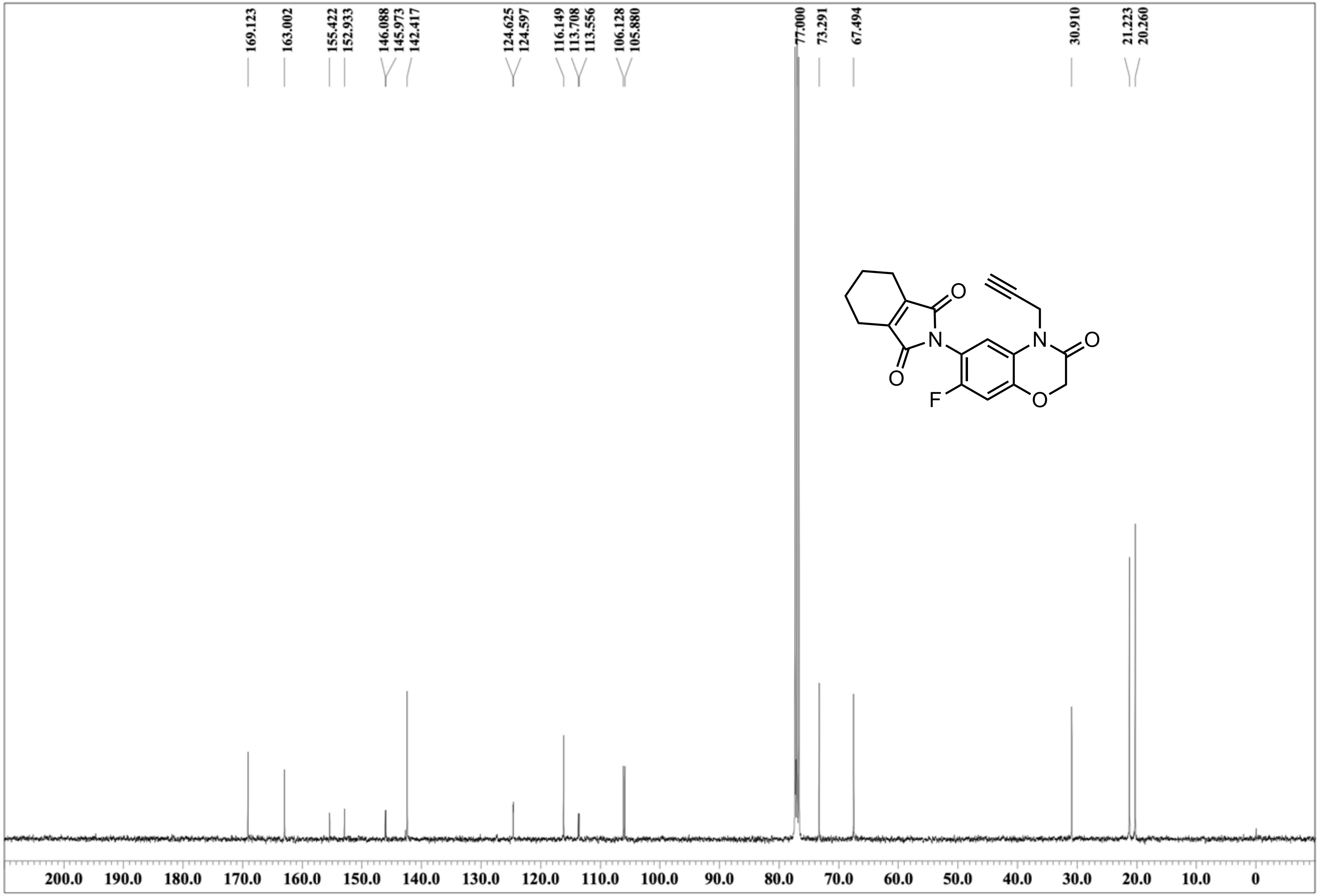

^{13}C NMR (100.6 MHz, $CDCl_3$) spectrum of **S6**

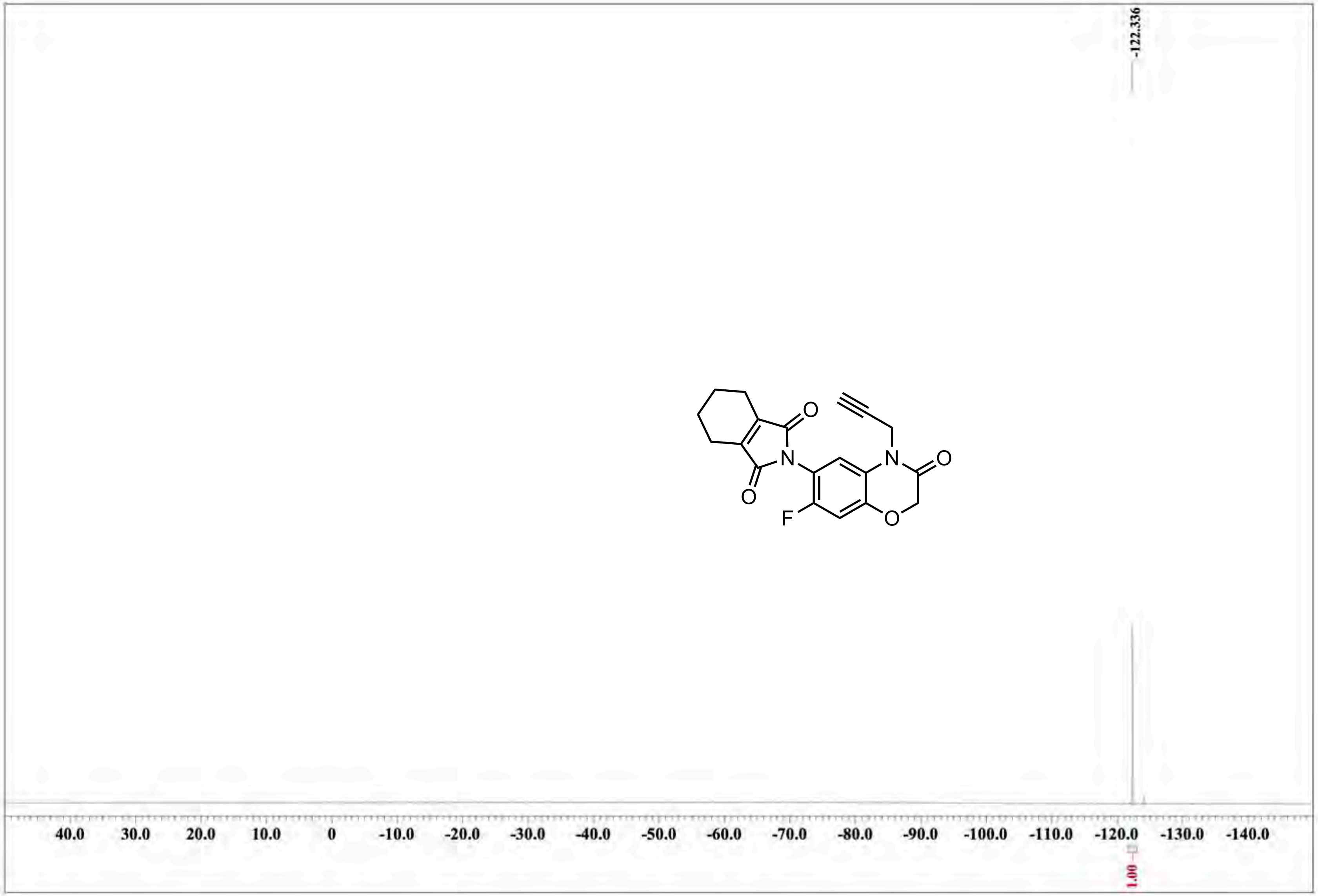

^{19}F NMR (376.5 MHz, $CDCl_3$) spectrum of **S6**

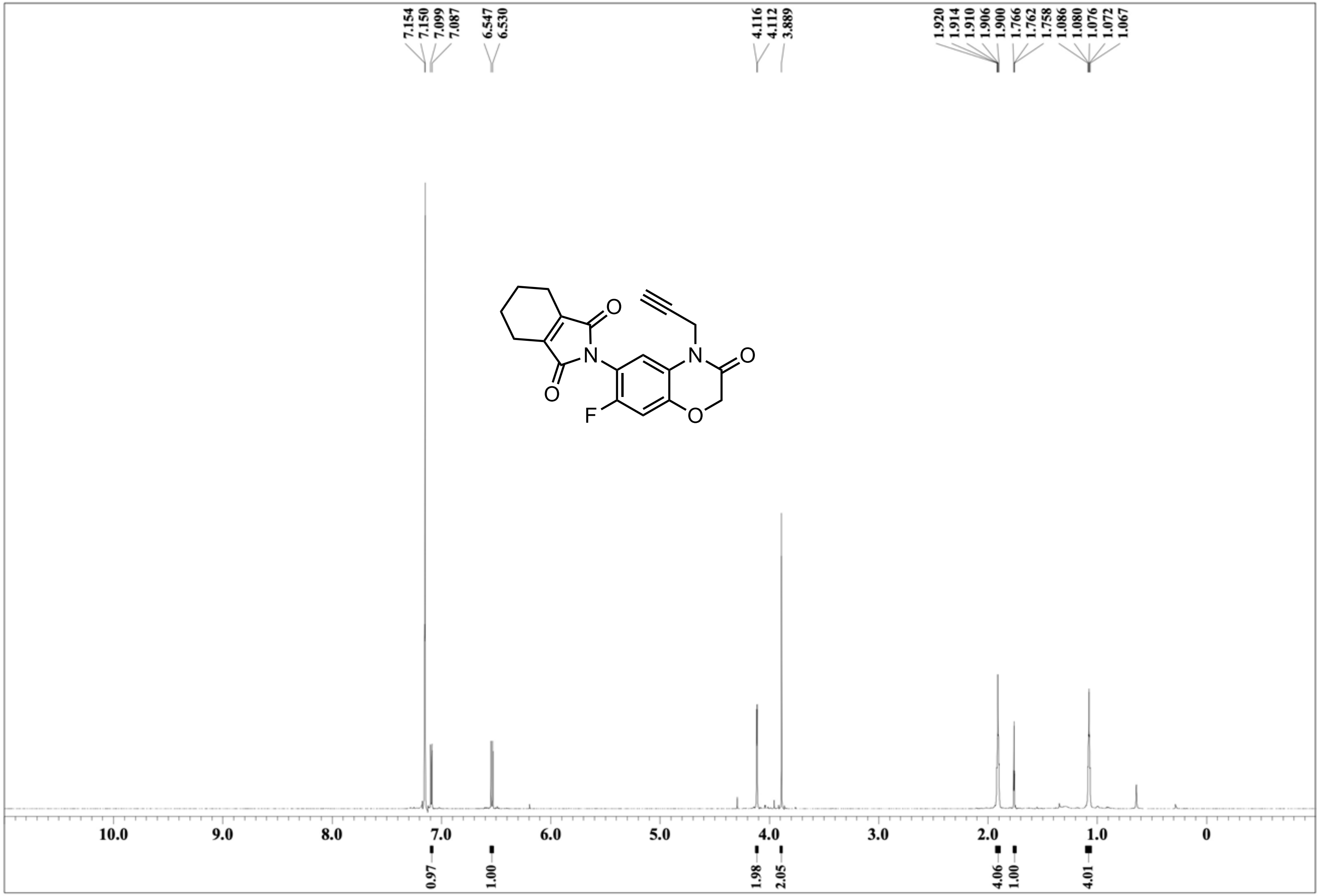

^{1}H NMR (600 MHz, C_6D_6) spectrum of **S6**

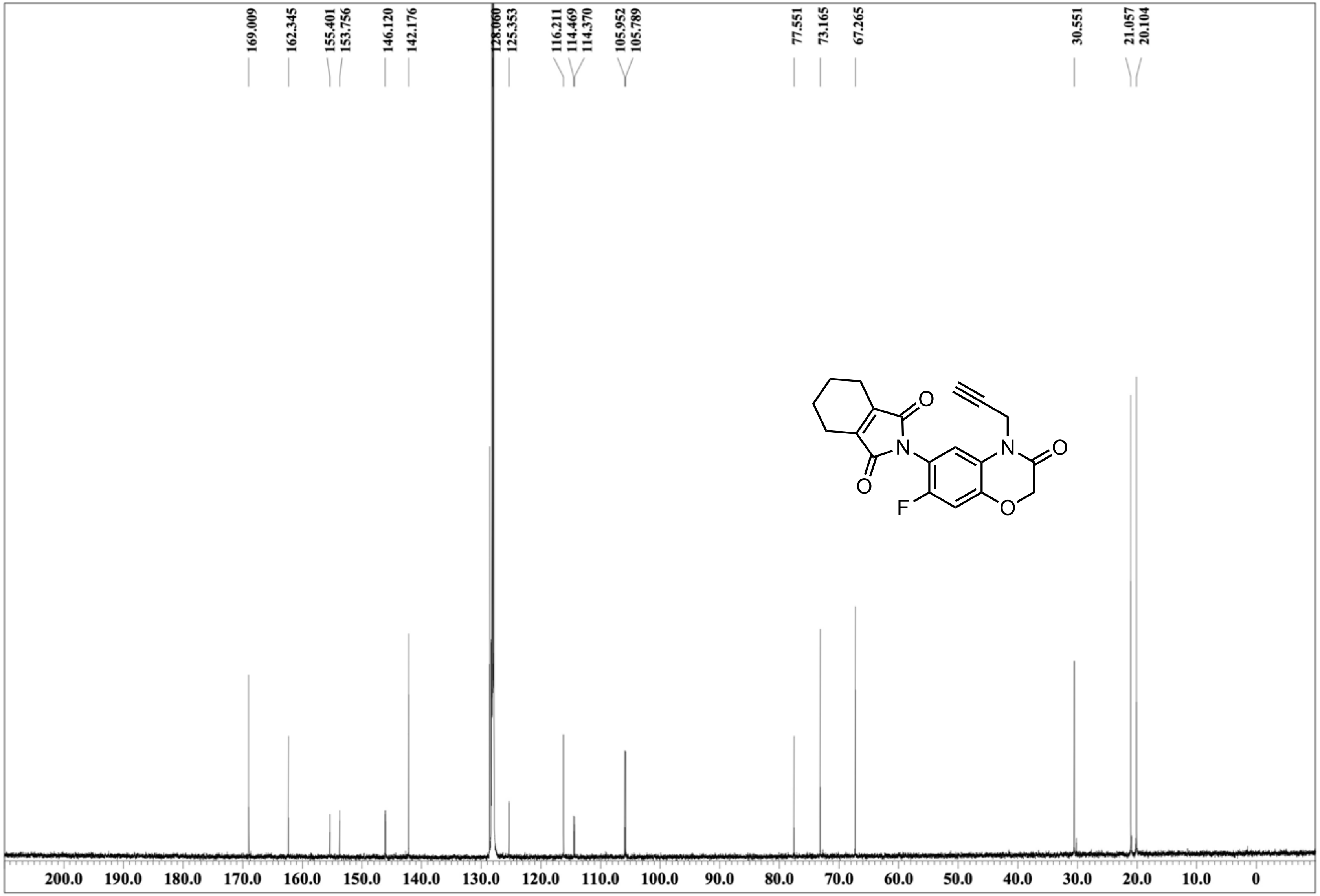

^{13}C NMR (100.6 MHz, C_6D_6) spectrum of **S6**
